# ﻿Preliminary notes on *Justicia* (Acanthaceae) in Peru

**DOI:** 10.3897/phytokeys.258.144435

**Published:** 2025-06-16

**Authors:** Rosa Villanueva-Espinoza, Yunfei Deng, Robert Scotland, John R. I. Wood

**Affiliations:** 1 State Key Laboratory of Plant Diversity and Specialty Crops, South China Botanical Garden, Chinese Academy of Sciences, Guangzhou, 510650, China Chinese Academy of Sciences Guangzhou China; 2 Key Laboratory of National Forestry and Grassland Administration on Plant Conservation and Utilization in Southern China, Guangzhou, 510650, China Key Laboratory of National Forestry and Grassland Administration on Plant Conservation and Utilization in Southern China Guangzhou China; 3 University of Chinese Academy of Sciences, Beijing 100049, China University of Chinese Academy of Sciences Beijing China; 4 División de Ecología Vegetal – CORBIDI, Calle Santa Rita 105 Of. 2, Urb. Huertos de San Antonio Monterrico, Surco, Lima, Peru División de Ecología Vegetal – CORBIDI Lima Peru; 5 Department of Biology, University of Oxford, South Parks Road, Oxford, OX1 3RB, UK University of Oxford Oxford United Kingdom; 6 Honorary Research Associate, Royal Botanic Gardens, Kew, TW9 3AB, UK Royal Botanic Gardens Kew United Kingdom

**Keywords:** Distribution maps, illustrations, new species, pollen, taxonomy, types

## Abstract

*Justicia* L. is the most species-rich genus of Acanthaceae in Peru and is here accepted in its broader traditional circumscription. The present paper marks a preliminary step towards a complete revision of the genus in Peru. Taxonomic notes and full synonymy are provided for 45 species including twenty-one new species, all apparently endemic to Peru: *Justiciaangustituba* J.R.I.Wood & R.Villanueva, *J.baguensis* J.R.I.Wood & R.Villanueva, *J.bambusiformis* J.R.I.Wood & R.Villanueva, *J.chamaecaulis* J.R.I.Wood & R.Villanueva, *J.cajamarcensis* R. Villanueva & J.R.I.Wood, *J.discolor* J.R.I.Wood & R.Villanueva, *J.falcifolia* J.R.I.Wood & R.Villanueva, *J.huallagensis* R.Villanueva & J.R.I.Wood, *J.hyalina* J.R.I.Wood & R. Villanueva, *J.lactiflora* J.R.I.Wood & R.Villanueva, *J.longibracteata* J.R.I.Wood & R.Villanueva, *J.oppositiflora* R.Villanueva & J.R.I.Wood, *J.oxapampensis* R. Villanueva & J.R.I.Wood, *J.rojasiae* R.Villanueva & J,R.I.Wood, *J.saccata* R.Villanueva & J.R.I.Wood, *J.sagasteguii* J.R.I.Wood & R.Villanueva, *J.schunkei* J.R.I.Wood & R. Villanueva, *J.spathuliformis* R.Villanueva & J.R.I.Wood, *J.tumbesiana* R.Villanueva & J.R.I.Wood, *J.valenzuelae* J.R.I.Wood & R.Villanueva and *J.werffii* J.R.I.Wood & R.Villanueva. New subspecies, subsp. machupicchuensis J.R.I.Wood & R.Villanueva of J.alpina Lindau and subsp. filisepala J.R.I.Wood & R.Villanueva of *J.discolor* are described. The genus *Tessmanniacanthus* Mildbr. is united with *Justicia*, the only species in the genus, *T.chlamydocardioides* Mildbr. being treated as *Justiciachlamydocardioides* (Mildbr.) R.Villanueva & J.R.I.Wood. *Justicialoxensis* Wassh., *J.poeppigiana* (Nees) Lindau, *J.sessilifolia* (Lindau) Wassh., *J.soukupii* (Standl. & F.A. Barkley) V.A.W.Graham and *J.tenuistachys* (Rusby) Wassh. & J.R.I.Wood are treated as synonyms respectively of *J.chimboracensis* Wassh., *J.secundiflora* (Ruiz & Pav.) Vahl, *J.alpina* Lindau, *J.radicans* Vahl and *J.tenuiflora* Ruiz & Pav. Fourteen names are lectotypified. The paper is copiously illustrated with line drawings, photographs and pollen images. Distribution maps supplement the information on ecology and distribution provided for all species discussed.

## ﻿﻿Introduction

*Justicia* L. is generally accepted as the largest genus of Acanthaceae with around 1000 species (Manzitto-Tripp et al. 2022) but its circumscription has been in doubt since almost the beginning. Nees (1847b) notably divided it into numerous smaller genera but since then there has been a tendency to recognise a broader *Justicia* culminating in the paper by Graham (1988), which treated the genus in a very broad sense. Graham’s treatment has been followed by most authors since (Scotland and Vollesen 2000; Ezcurra 2002; Wasshausen and Wood 2004; Darbyshire et al. 2010; Wasshausen 2013; Deng 2020) but molecular studies (Kiel et al. 2017, 2018; Manzitto-Tripp et al. 2022) show *Justicia* is paraphyletic although the New World species form a monophyletic clade, if a few smaller genera (*Cephalacanthus* Lindau, *Clistax* Mart., *Harpochilus* Nees, *Megaskepasma* Lindau, and *Poikilacanthus* Lindau) are included (Kiel et al. 2018). Clearly a new classification will be necessary as the *Justicia* name is associated with an Old-World species.

*Justicia* sensu Graham (1988) shows major variation in many aspects of its morphology, including habit and the structure and size of the calyx and corolla. Variation in pollen and seed morphology is very marked and has been widely used both in species delimitation and in attempts to achieve a satisfactory infrageneric classification or to split the genus into separate genera. Graham divided the genus into 16 sections and various subsections based on a range of characters but her infrageneric classification never accounted for all species and her sections were shown by Kiel et al. (2017, 2018) not to be monophyletic. Kiel et al. (2018) in turn used modern molecular methods to identify nine major clades but only a relatively small proportion of species were sequenced and the clades are not always easily recognised from morphology. We have referenced these clades and Graham’s sections where it seemed useful but not in any systematic way. There is still no accepted circumscription of *Justicia*, of its infrageneric elements nor an accepted classification of the Justiciinae Nees as a whole. In the absence of a satisfactory new classification of *Justicia*, we treat the genus in the sense of Graham (1988). As such it is clearly the largest genus of Acanthaceae in Peru as in all the neighbouring countries except Chile, with 72 species recognised in Bolivia (Jørgensen et al. 2015), 157 in Brazil (Chagas and Costa-Lima 2024), 104 in Colombia (Wood et al. 2024) and 45 in Ecuador (Wasshausen 2013).

Despite the problems in circumscribing *Justicia* s.l., it is usually readily recognised by the strongly 2-lipped corolla, the paired superposed bithecous anthers, the lower thecae usually with a basal appendage and by the 4-(rarely 2-) seeded capsule. The pollen is often distinctive although intraspecific pollen variation is very poorly understood. In the descriptions of new species in this paper we have endeavoured to describe these characters in every case but it has not always been possible. The capsules and seeds of a surprisingly large number of species are unknown. In a few cases open corollas are missing from the available collections. It has not always been possible to extract pollen successfully, either because of difficulties with the extraction process or because we wanted to avoid destructive sampling of limited material.

As our studies of Peruvian Acanthaceae are on-going, we are uncertain of the number of Peruvian species but it much exceeds the 49 reported in the Catalogue (Brako and Zarucchi 1993), even though not all of these can be accepted as occurring in Peru. *Justicia* is very diverse in Peru, especially on the eastern slopes of the Andes below 2500 m. However, it is absent from the coastal lowlands and poorly represented on the dry western flanks of the Andes. The Amazonian lowlands are still relatively poorly explored botanically, but *Justicia* diversity appears to fall further east from the Andean foothills. Records of *Justicia* species date back to the end of the 18^th^ century (Ruiz and Pavón 1798) but there have been no systematic studies of the genus in Peru and new species have been described sporadically over the years by diverse authors as part of wider publications (Nees 1847a, 1847b, Wasshausen and Wood 2003) or in scattered short papers (Lindau 1894, 1897, 1904, 1905, 1922; Mildbraed 1926; Wasshausen 1988, 1997, 2006; Wasshausen and Wood 2003).

We have already made significant advances in our studies of *Justicia* (for example Gallego-Jiménez and Wood 2024) and the present paper presents further results, but a number of complex issues remain, which will be treated in a later paper when we have had the opportunity to further research these issues. These include the circumscription of a number of morphologically variable species, including *J.cuzcoensis* Lindau, *J.mendax* (Lindau) Wassh. and *J.ruiziana* Lindau, the relationship of Peruvian species, such as *J.loretensis* Lindau and *J.obovata* Wassh. & J.R.I.Wood, with apparently related species from neighbouring countries and the evaluation of the status of a good number of putative new species currently represented by inadequate material. It is expected that the final paper will include additional new species, an updated checklist, some conservation assessments and a key to all species of *Justicia* occurring in Peru.

## ﻿﻿Materials and methods

This study provides notes on 45 species of *Justicia* occurring in Peru, probably slightly under half the final total number of species found in the country. The species selected are new taxa, new records for Peru, new names or new synonyms or species requiring typification or needed to compare with other species discussed. Our studies are based principally on herbarium studies by Rosa Villanueva and John Wood, informed by fieldwork by one of us (Rosa Villanueva) and an extensive survey of relevant literature, particularly the accounts of *Justicia* in neighbouring countries (Leonard 1958; Wasshausen and Wood 2003, 2004; Wasshausen 2013). Herbarium specimens in BM, CPUN, CUZ, HAG, HOXA, HUT, K, Laboratorio de Dendrología (Cajamarca), MOL, OXF, US and USM (acronyms according to Thiers, 2023) were studied together with specimens loaned from BRIT, F, MO and US to John Wood at Kew. Online images from many herbaria were also accessed via Jstor (https://plants.jstor.org/), Tropicos (https://www.tropicos.org/), Reflora (https://reflora.jbrj.gov.br/reflora/), GBIF (https://www.gbif.org/) and Atrium (https://atrium.andesamazon.org/) and from websites of individual herbaria including B, BRIT, F, G, HBG, K, MEXU, MO, NY, P, US and W. All specimens cited were seen by one or all of us either in a herbarium or as an image unless explicitly indicated otherwise.

Field work was carried out by Rosa Villanueva and colleagues in recent years in diverse regions of Peru. Specimens were collected and deposited mostly in HOXA, MOL and IBSC. Photographs were taken of species seen in the field and observations on colour and habitat were noted at the same time.

Descriptions were prepared based mostly on herbarium specimens but with some reference to fresh material or information in the literature. Full descriptions are provided for new species, but not all information was available in every case, and in particular details of capsules and seeds are not always known, even in cases of some common species, such as *Justiciaalpina*. Shorter descriptions are provided for established species with an emphasis on diagnostic characters or on information not previously available, pollen in particular. We have lectotypified names where there is possible ambiguity, following the International Code of Nomenclature for Algae, Fungi and Plants (Turland et al. 2018). Explanations are provided in many individual cases. In the case of Ruiz and Pavon names, we have chosen specimens from Madrid, selecting the most complete example where there is more than one syntype.

Line drawings have been prepared for all new taxa based on specimens from Peru. Some of these were prepared some twenty years ago by Alice Tangerini and Cathy Pasquale at the Smithsonian Institution (US) at the request of Dieter Wasshausen. Some of their drawings were used in a paper by Wasshausen (2006) but the majority were never used. We are very pleased to have received permission to use these in the current paper. However, the majority of the line drawings were commissioned specially for this paper with the support of the Royal Botanic Gardens, Kew and drawn by Margaret Tebbs. Specimens were examined under a binocular microscope and when necessary dissected for artwork or detailed morphological study after softening either by boiling or with the use of a softening agent such as Libsorb.

Pollen was extracted from both herbarium and field collections, and acetolysed following Erdtman (1960). Pollen was then mounted on Scanning Electron Microscopy stubs, sputter coated with gold palladium and imaged by a JEOL 7610F scanning electron microscope at Oxford and by Hitachi, Regulus-SU8100 in Guangzhou. Descriptions of pollen followed previous research by Graham (1988) as closely as possible. Pollen in this study was distinguished by the presence or absence of a trema area (thinning of the exine associated with area surrounding the aperture) (Figs 48A, 49C), an uninterrupted band of sexine on either side of the aperture (Figs 48F, 51D), presence of discrete islands of sexine (insulae) (Figs 49C, 51B, 52A, 52B), the number of rows of insulae on either side of the aperture (Figs 50B, 52E), invaginations of sexine from the area outside the trema area into the trema area (peninsulae) (Figs 48A, 51F), presence of verrucae (Fig. 50C), aperture number 2 (Fig. 51F) or 3 (Figs 51B, 52B) and P:E (polar to equatorial) ratio.

Information on phenology and habitat is based partly on our field observations but mostly on the data provided by herbarium labels. We have tried to generalise from this information so patterns of flowering and habitat preferences can be discerned. In general species growing in dry country flower in the second half of the rainy season (January to April) while plants from moist forest flower in the dry season (June to September) but there is much individual variation.

The distribution maps were prepared using data gathered from field collections and herbarium labels using ArcGIS Pro 3.0. The elevation data was downloaded from WorldClim 2.1 (Fick and Hijmans 2017).

Conservation assessments are not systematically included. Where assessments have been made previously (León 2006, for example), they were almost certainly premature. We are only in a slightly better position to make assessments because of the larger number of records. However, we do not know whether the records represent extant populations or the size or extent of each population. Even less information is known about the state of the habitat of individual species, although it seems likely that the lowland forest where many species were found has been felled or degraded. We have occasionally made inferences where these are apparent from the records. However the absence of records in the last forty years, in the cases of *Justiciapelianthia*, *J.lactiflora* and *J.schunkei*, for example, may reflect lack of collecting activities rather than extinction. The recent rediscovery of *J.cuspidulata* after more than 150 years underlines the dangers of assuming that the absence of records implies extinction. Recent records of *J.falcifolia* and *J.chamaecaulis* after a prolonged gap in time serve as a similar caution. The work of making reliable conservation assessments lies in the future with those in a position to assess the status of populations in real future time.

## ﻿﻿Taxonomy

In the following notes species are arranged in informal groups based partly on morphology, partly on palynology and/or partial molecular results and ecology. It is hoped that this organisation is more informative to the reader than the use of an alphabetical arrangement. A key would be premature at this stage but the arrangement should help readers to place an unknown species or compare one species with another.

The arrangement is as follows:

Species 1–6 Calyx 4-lobed; corolla relatively large (> 2.5 cm long); inflorescence a terminal spike; lower anther theca usually lacking basal appendage.

Species 7 As for 1–6 but corolla small (< 2.5 cm long).

Species 8 As for 7 but calyx 5-lobed and lower anther theca with basal appendage.

Species 9–16 Corolla long, narrow, tubular; calyx 5-lobed, Inflorescence of terminal and/or axillary spikes, Pollen large. Shrubs from tropical forest.

Species 17–19 Calyx 4-lobed, corolla with slender cylindrical tube, pink or white. Thecae very strongly superposed.

Species 20–23 Bracts setaceous, bristle-like, seeds smooth; calyx 5-lobed. Flowers in dense subcapitate spikes. Mostly Amazonian lowlands.

Species 24–27 Corolla white; calyx 5-lobed; Capsule 2–4-seeded. Seeds smooth. Pollen often verruculose; woody subshrubs of dry NW region.

Species 28–31 Inflorescence a terminal panicle of spikes; calyx 4 or 5-lobed

Species 32–35 Species with spathulate bracts, 5-lobed calyx and small corollas.

Species 36–37 Species with small corollas < 1.5 cm long and oblong or lanceolate bracts.

Species 38–45 Species with corollas > 2.5 cm long and oblong or lanceolate bracts.

Species 1–7. ﻿Most of these species would have been placed in Sect. Dianthera (L.) V.A.W.Graham, subsect. Saglorithys (Rizzini) V.A.W.Graham in Graham’s (1988) classification of Justicia s.l. This subsection is well represented in Peru and is characterised by the 4-lobed calyx, terminal inflorescence, the absence of appendages on the anther thecae and the capsule with a sterile base. Species 1–6 are relatively large-flowered, whereas in sp. 7 (*J.discolor*), the corolla is small. Molecular studies (Kiel et al. 2017) do not support this as a monophyletic group. More sampling is needed.

### ﻿﻿1. Justicia alpina

Taxon classificationPlantaeLamialesAcanthaceae

Lindau, Repert. Spec. Nov. Regni Veg. 1: 159. 1905. (Lindau 1905: 159)

39DF38E3-4AE3-5AB1-AC5B-6743112E799F

Beloperone sessilifolia Lindau, Notizbl. Bot. Gart. Berlin-Dahlem 8: 247. 1922. (Lindau 1922: 247) Type. PERU. Lambayeque, Olmos, 1900–2000 m, *A. Weberbauer* 7105 (presumed holotype B†, isotypes GH 00093741, F-0040524F, US-00478761, US-01268829).
Justicia sessilifolia (Lindau) Wassh., Monogr. Syst. Bot. Missouri Bot. Gard. 45: 1253. 1993 (Wasshausen 1993: 1253), syn. nov.

#### Type.

Peru • Encima de San Pablo, Cajamarca, 2400–2700 m, *A. Weberbauer* 3817 (presumed holotype B†, photo of holotype FOBN008806, isotype MOL-00005624).

#### Description.

Low subshrub scarcely 0.5 cm high, stems often simple. Leaves sessile or shortly petiolate, lamina 2–5 × 1.2–4 cm, ovate, base rounded to cordate, apex acuminate, sparsely hirtellous adaxially, glabrous abaxially, cystoliths conspicuous. Inflorescence of terminal spikes 6–20 cm long, the flowers in opposite pairs; peduncles and rhachis pubescent; bracts at base of inflorescence foliose, floral bracts and bracteoles 6–7 × 0.5–1 mm, lanceolate; calyx subequally 4-lobed, lobes 8–10 × 0.5 mm, lanceolate, white-pilose; corolla 2.2–2.4 cm long, pink or violet, glabrous, tube 10–12 mm long, upper lip 12 × 3 mm, notched, lower lip 11 × 8 mm, 3-lobed, lobes 2–2.5 mm long, thecae 1.5–2.5 × 0.5 mm, oblong, glabrous, parallel, superposed, lower basally acute but without appendage. Capsule 11–14 × 3 mm, weakly clavate, glabrous, 4-seeded; seeds verucullose.

#### Conservation.

This species was assessed as EN, B1ab(iii) following IUCN (2024) guidelines (León 2006). However, this categorisation is likely to have been premature as numerous additional records are now known. The two subspecies recognised below were not then known and no evaluations of the populations or habitats were carried out.

Divisable into two geographical subspecies:

### ﻿﻿1a. Justicia alpina subsp. alpina

Taxon classificationPlantaeLamialesAcanthaceae

074A9E73-6839-5248-A366-0A2BD06A224F

#### Diagnosis.

This subspecies is distinguished by the bracts which are foliose to about the middle of the spike, the lower often entirely leaf-like. The leaves are broadly ovate to suborbicular, up to 4 cm broad and often about as broad as long. They are strictly sessile.

#### Illustration.

Fig. 1A–D.

**Figure 1.**
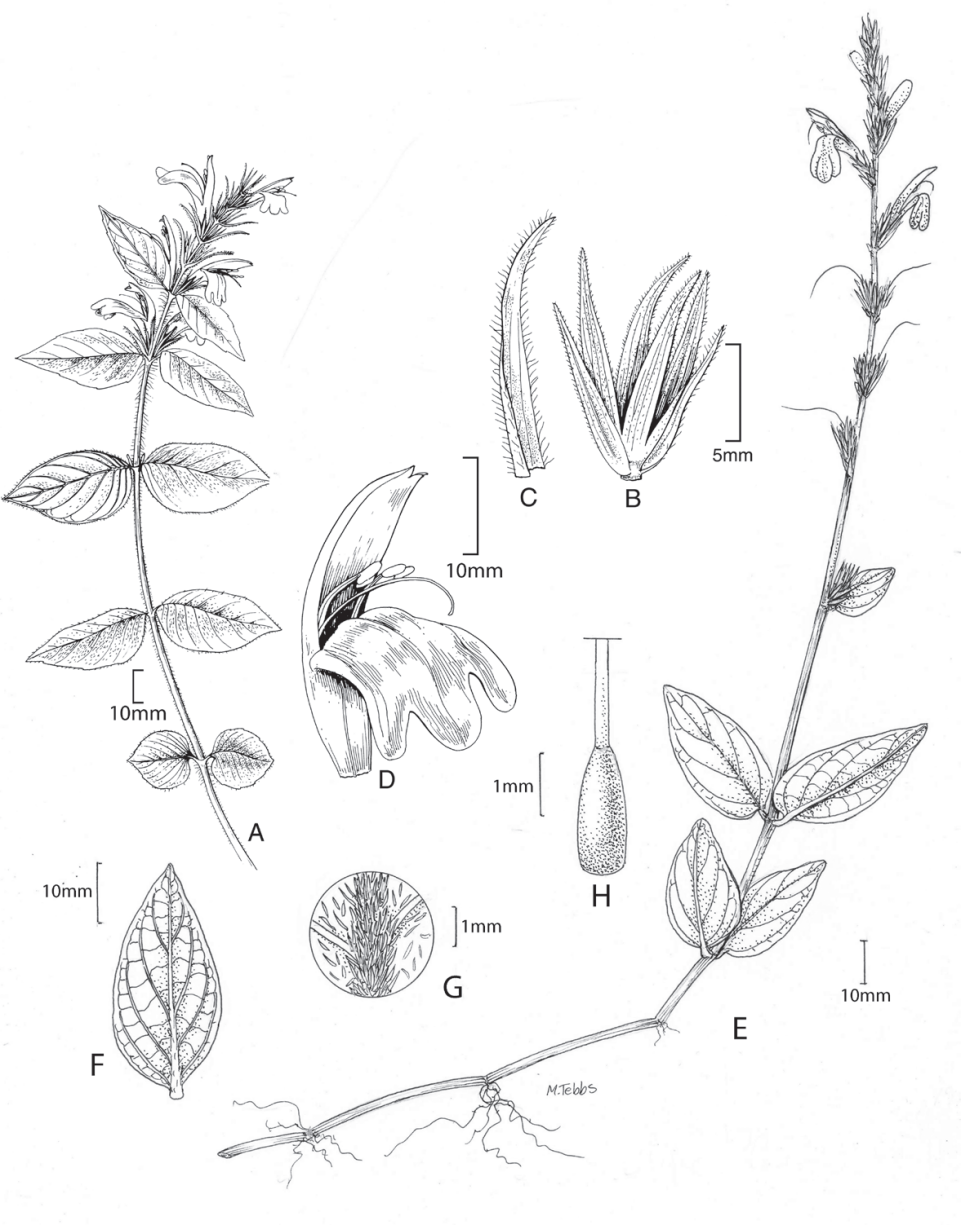
Justiciaalpinasubsp.alpina**A** habit **B** calyx with bracts and bracteoles **C** bract **D** corolla opened out to show stamens. Justiciaalpinasubsp.machupicchuensis**E** habit **F** leaf showing shape and abaxial surface **G** detail of midvein of adaxial surface of leaf **H** ovary. **A**–**D** drawn from *Sagástegui* 7933 by Cathy Pasquale; **E**–**H** drawn from *Ugent* 5331 by Margaret Tebbs.

#### Habitat.

Dry forest between about 1400 and 3000 m.

#### Phenology.

Flowers from March to May.

#### Distribution.

Endemic to three departments of northwestern Peru. Fig. 53.

#### Material examined.

**Peru** • **Cajamarca**: Prov. Cajamarca, arriba de San Pablo, 2470 m, 22 May 1975, *A. Sagástegui* 7933 (HUT, MO, US); • ibid., Dist. Chetilla [7°09'S, 78°40'W], *L. Dávila et al.* 5126 (Laboratorio de dendrologia). Prov. Chota, Dist. Llama [6°33'S, 78°38'W], 2200 m, 6 April 2012¸ *L. García Llatas* 8608 (USM); • ibid., road Llama–Huambos, 2100 m, 22 May 1965, *A. López & A. Sagástegui* 5268 (HUT, US). Prov. Contumazá, La Montana, road to Guzmango [7°22'S, 78°48'W], 2500 m, 18 May 1979, *A. Sagástegui* 9299 (F, MO, US); • ibid., Dist. Contumazá, El Molino (Cascas–Contumazá), 07°25'58"S, 078°47'30"W, 1800 m, 5 April, 1985, *A. Sagástegui* 12554 (MO, US); • ibid., km 13 Chilete–Contumazá, 1400 m, 3 May 1980, *I. Sánchez Vega* 2241 (CPUN, MO); ibid., Bosque Natural de Cachil, *L. Dávila* 166 (Laboratorio de dendrologia, Cajamarca); • ibid., Chilete, Bosque de Huertas, [7°13'S, 78°50'W] 1300–1500 m, *L. Dávila* 822 (Laboratorio de dendrologia, Cajamarca); • ibid., Contumazá, surroundings of Contumazá, 2870–3000 m, 3 May 1999, *M. Binder et al.* 1999/68 (F); • ibid., Contumazá, entre Contumazá y Amanchaloc, 2870–3000 m, 3 May 1999, *E. Rodríguez et al.* 2218 (F, USM); • ibid., desvío a bosque de Cachil, 2600 m, 30 May 1990, *A. Sagástegui* 14283 (F, US); • ibid., El Platanar Hydroelectric Power Plant (road Cascas–Contumazá), 1400 m, 31 March 1994, *A. Sagástegui et al.* 15210 (F, HAO, US); • ibid., Dist. Chilete, Bosque de Huertas, [7°13'S, 78°50'W], 1300–1500 m, 22 April 2006, *L. Dávila* 822 (Laboratorio de dendrología, Cajamarca); • ibid., Shillas, 2725 m, 20 March 2006, 7°19'43"S, 78°48'40"W, *I. Sánchez V*. 227 (CPUN); • ibid., Dist. Guzmango, surroundings of Guzmango, 2500 m, 30 April 1990, *A. Sagástegui* 14260 (F, US, USM); • ibid., La Pampa, 2730 m, 30 May 1959, *A. Sagástegui & R. Samami* 2940 (F, US, HUT); • ibid., C.C.P.P. La Erilla, 2800 m, 2 April 1981, *A. Sagástegui* 9708 (HUT, MEXU, US); • ibid., track from Guzmango to San Benito, 7°23'24"S, 78°54'01.4"W, 2562 m, 25 March 2001, *T. Henning & C. Schneider* 43 (HUT, USM); • ibid., surroundings of Yatón, 2200 m, 1 May 1981, *A. Sagástegui et al.* 9763 (HUT, US); • ibid., Dist. San Benito, Las Chirimoyas (road San Benito–Guzmango), 07°25.5'S, 78°55.5'W, 1592 m, 6 May 2001, *A. Sagástegui et al.* 16421 (F, HAO, US); • ibid., C.C.P.P. Andaloy, 1550 m, 5 May 1965, *A. Sagástegui & M. Fukushima* 5057 (HUT, US); • ibid., surroundings of Yatón, 2200 m, 1 May 1981, *A. Sagástegui et al.* 9763 (HUT, US). Prov. San Pablo, El Civil, Ladera, [7°06'32"S, 78°49'10"W], 2200 m, 12 June 1993, *J. Sánchez Vega* 655 (CPUN); • ibid., entre Conga y Sangal [7°05'S, 78°23'W], 2100 m, 26 March 2004, *I. Sánchez Vega et al.* 12611 (CPUN, F). • **La Libertad**: Prov. Gran Chimú, Corlás (Cascas–Contumazá) [7.29S 78 49W], 1450 m, 26 April 2002, *A. Sagástegui* 16883 (F, US). Prov. Otuzco, Dist. Sinsicap, camino a Paranday, [7°53'S, 78°42'W], 2650 m 1 May 1950, *M. López* 1052 (US); • ibid., 1 May 1954, *A. López* 1081 (US); ibid., 1 May 1954, *A. López et al.* 2289 (US); • ibid., Llacom, 2500 m, 16 March 1954, *M. Vargas* 0158 (US); • ibid., Dist. Huaranchal, 2300 m, 7 June 1958, *A. López et al.* 2688 (HUT, US). Prov. Trujillo, Hac. Campoden [8°07'S, 78°56'W], 27 March 1947, *O. Velarde* 447 (US). • **Piura**: Prov. Huancabamba, Porculla, 2200 m, 10 May 1992, *S. Llatas Queiroz & H. de La Cruz* 3102 (F, MO, US).

### ﻿﻿1b. Justicia alpina subsp. machupicchuensis

Taxon classificationPlantaeLamialesAcanthaceae

J.R.I.Wood & R.Villanueva subsp. nov.

A1D989E5-81B8-5DB2-B82A-0E0FBE61B3BC

urn:lsid:ipni.org:names:77363404-1

#### Type.

Peru • Cusco, Prov. Urubamba, Dist. Machu Picchu, Urubamba Valley, *E.K. Balls* 6801 (holotype K-000544761, isotypes BM, F, US).

#### Diagnosis.

This subspecies is distinguished from alpina by the bracts which are distinct from the leaves and only those subtending the lowermost flower pair sometimes foliose, thus giving the spikes a naked appearance. The leaves are smaller, not exceeding 2.5 cm wide, always clearly ovate and sometimes with very short petioles < 3 mm long. Pollen prolate, 49–54 × 28–31 μm, 2-aperturate, colporate, 1 row of c. 6–8 peninsulae on either side of the aperture (Fig. 48A).

#### Illustration.

Figs 1E–H, 2.

**Figure 2.**
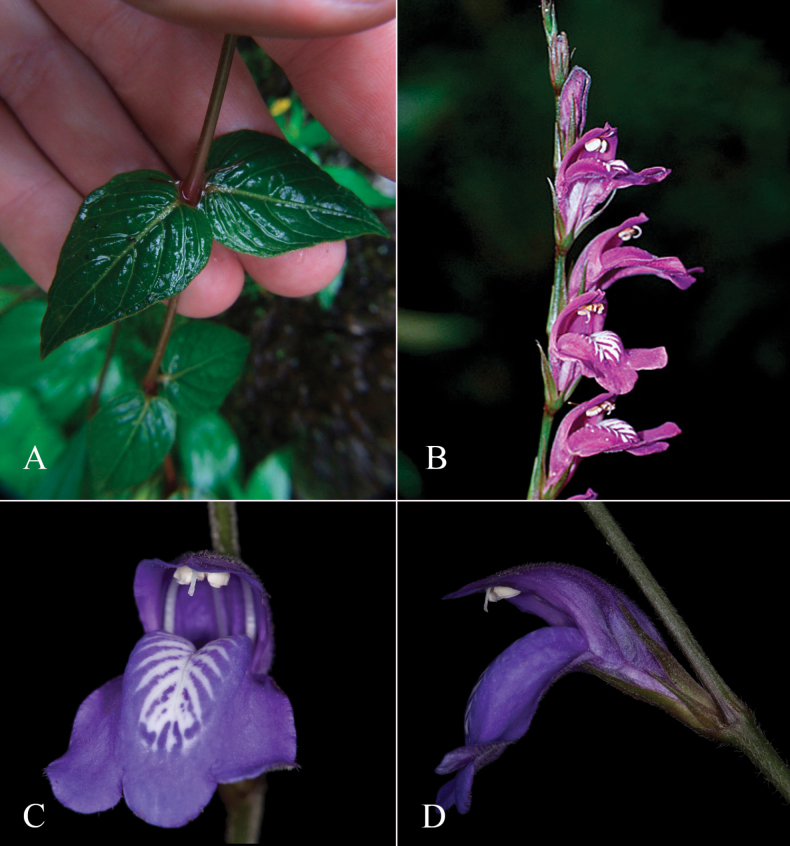
Photographs of Justiciaalpinasubsp.machupicchuensis, showing subsessile leaves, colour variation in corolla and prominent “herring bone” patterning **A** INaturalist **B** R. Foster **C**–**D** J.L. Clark

#### Etymology.

This subspecies is named after the Inca site of Machu Picchu, from the surroundings of which almost all records come.

#### Phenology.

Flowers throughout the year.

#### Habitat.

Open places such as stone walls, disused terraces, rock ledges and grassy slopes in semi shade from 2200 to 3600 m approximately.

#### Distribution.

Endemic to two Andean departments of southern Peru and, with a single exception, restricted to the Machu Picchu area. Fig. 53.

#### Material examined.

**Peru** • **Cusco**: Media Naranja hill, 8750 ft, 27 March 1959, *S.G.E. Saunders* 428 (BM). Prov. La Convención, Dist. Vilcabamba, Lucma Yupancca, 2640–2750 m, 4 June 2022, *W. Galiano et al.* 4327 (CUZ). Prov. Urubamba, on way to Yuncapata, Aug. 1941, *C. Dreyfus* s.n. (USM 12766); • ibid., Dist. Machu Picchu, San Miguel Valley, 2400 m, 20 July 1928, *F. Herrera* 1995 (F); • ibid., Machu Picchu [13°09'S, 72°31'W], 3 Aug. 1930, *C. Vargas* 3170 (F); ibid., 200–300 m, 27 March 1942, *C. Vargas* 2636 (CUZ); • ibid., Huayna Picchu, 2650 m, 21 July 1961, *C. Vargas* 13608 (CUZ); • ibid., 12,000 ft, 9 May 1930 *E.K. Balls* 6801 (BM, K, F, US); • ibid., 2200 m, Oct. 1931, *F. Herrera* 3203 (F); • ibid., 3200 m, 16 May 1936, *Y. Mexia* 8081 (K, MO, US); ibid., 8500 ft, 1 June 1937, *D. Stafford* 794 (BM, F, K); • ibid., 12 Nov. 1937, *D. Stafford* 1222 (BM, F, K); ibid., 2400 m, Feb. 1938, *C. Vargas* 842 (F, CUZ, US); • ibid., 2200 m, 4 Feb. 1939, *H.E. Stork et al.* 10508 (F); • ibid., 7500 ft, 29 March 1939, *W. Balfour Gourlay* 90 (K); • ibid., 3800 m, 9 March 1943, *M. Cardenas* 2297 (US); • ibid., just below ruins of Machu Picchu, 3 March 1958, *D.S. Correll & E.E. Smith* P271 (US); • ibid., 2300 m, 20 May 1958, *Reitz* 5984 (US); • ibid., Valley of Urubamba river, 2400 m, 22 May 1958, *H. Humbert* 30685 (US); ibid, 2500–2600 m, 3 Jan. 1963, *H. Iltis et al.* 1042 (K, US); • ibid., 26 May 1963, *D. & V. Ugent* 5331(K, US); • ibid., Inca ruins of Machu Picchu, 2300 m, 14 March 1967, *L.E. Skog* 1133 (US); • ibid, N of Cuzco, 12 July 1969, *W.J. Colaris* 1327 (U); • ibid., above Machu Picchu, along old Inca trail, 2600 m, 17 April 1977, *Al. Gentry et al.* 19403 (P, US); • ibid., between Machu Picchu and Waina Picchu, May 1980, *W.G. D’Arcy* 13747 (MO); • ibid., Inca Trail Circuit, between Aguas Calientes town and Baños Termales, 13°09'S, 072°32'W, 2400–2700 m, Oct. 1986, *A. Tupayachi* 223 (CUZ, US); • ibid., 13°09'S, 072°31'W, 2300–4150 m, 16–19 March 1988, *P. Núñez & F. Luna* 8877 (CUZ, F, US); • ibid., around Huaynapicchu, 2000–2700 m, 30 March 1989, *P. Nuñez & J. Smith* 10332 (CUZ); • ibid., Pampacachua Cedrobamba, 13°11'S, 72°27'W, 2415 m, 30 Dec. 2000, *A. Tupayachi et al.* 4441 (CUZ); • ibid., Trocha Iran Bingham, 13°09'47"S, 72°13'19"W, 2207 m, 20 May 2003, *I. Huamantupa et al*. 3237 (CUZ, MO); • ibid., Pampacahua, km. 94, 13°06'S, 72°16'W, 2376 m, 21 Jan. 2005, *L. Valenzuela et al*. 4615 (BRIT, CUZ, MO, USM); • ibid., Puente ruinas Machu Picchu, 2000–2400 m, 17 March 1965, *A. Aldave* 4984 (CUZ, HUT); • ibid., Aguas Calientes, near base of mountain to Machu Picchu, 13°09'17"S, 72°31'31"W, 2040 m, 26 May 2010, *J.L. Clark et al.* 11629 (NY, US). • **Puno**: Sandia, surroundings of Sandia [14°14'S, 69°26'W], 2250 m, 31 Jan. 1964, *C. Vargas* 015122 (CUZ).

### ﻿﻿2. Justicia cuspidulata

Taxon classificationPlantaeLamialesAcanthaceae

(Nees) Wassh., Monogr. Syst. Bot. Missouri Bot. Gard. 45: 1253. 1993. (Wasshausen 1993: 1253)

0702DCA2-5370-5A6C-A904-008D447BDC9E

Rhytiglossa cuspidulata Nees, Prodr. [A. P. de Candolle] 11: 348. 1847. (Nees 1847b: 348) Type. PERU. Amazonas, Chachapoyas. *A. Mathews* 3152bis (holotype K-000529252, isotypes BM-000617660, G-00236276, G-00236277, G-00236278, K-000529253, OXF-00194644, US-02878809, fragment).
Rhytiglossa hookeriana Nees, Prodr. [A. P. de Candolle] 11: 348. 1847. (Nees 1847b: 348) Type. PERU. Amazonas, Chacapoyas, Sesuya, *A. Mathews* 3152 (holotype K-000529384, isotypes BM-000617661, G-00236301, G-00236302, GZU-000251216 (fragment), K-000529383, OXF-00194660), syn. nov.
Dianthera hookeriana (Nees) Benth. & Hook.f. ex B.D.Jacks., Index Kew. 1(2): 742. 1893. (Jackson 1893: 742)
Ecbolium hookerianum (Nees) Kuntze, Revis. Gen. Pl. 2: 980. 1891. (Kuntze 1891: 980)
Justicia chachapoyasensis Wassh., Monogr. Syst. Bot. Missouri Bot. Gard. 45: 1253.1993. (Wasshausen 1993: 1253) Type. Based on Rhytiglossahookeriana Nees

#### Type.

Based on *Rhytiglossacuspidulata* Nees,

#### Description.

Stems pubescent. Leaves petiolate, 2–4 × 1–2.5 cm, ovate, obovate or elliptic, apex acuminate to rounded, acute, apiculate or cuspidate, only slightly longer than broad, base cuneate, pubescent; petioles 0.5–1 cm. Inflorescence a terminal spike, 6–10 cm long, usually much exceeding the subtending leaf pair, flowers in opposite pairs, bracts narrowly deltoid-lanceolate, c. 5 mm long, bracteoles 6–8 mm long, narrowly linear-lanceolate, hirtellous, slightly exceeding the 4-lobed calyx; calyx lobes 5–6 mm long, pubescent; corolla 12–18 mm long to apex of upper lip, pink, pubescent, lower lip slightly longer, spreading, lobed to 5 mm, lobes rounded; thecae weakly superposed, base muticous, c. 1 mm long. Capsule and seeds not seen.

#### Illustration.

Fig. 3.

**Figure 3.**
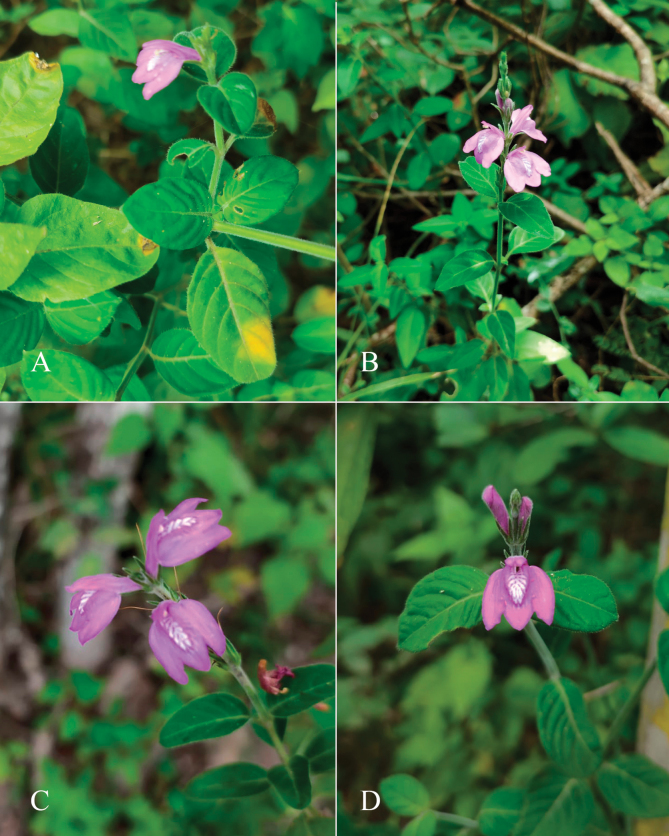
Photographs of *Justiciacuspidulata*. Note the pink flowers with prominent “herring bone” patterning and obtuse leaves with short petioles. Photographs of *Aybar* 13 by David Aybar.

#### Phenology.

Found in flower in March.

#### Habitat.

Disturbed woodland, 697 m.

#### Distribution.

A rare species endemic to Amazonas Province and only known from the type and a single modern collection. Fig. 54.

#### Material examined.

**Peru** • **Amazonas**: Prov. Utcubamba, Dist. Jamalca, between Bagua Grande and Pedro Ruiz, 5°52'41.5"S, 78°12'25.9"W, 697 m, 29 March 2024, *D. Aybar* 001 (MOL); • ibid., 4 May 2024, *D. Aybar* 013 (MOL).

#### Notes.

*Rhytiglossacuspidulata* Nees and *R.hookeriana* Nees were described by Nees in the same publication based on different sheets of the same collection number. Although the cuspidate leaves of the *R.cuspidulata* are very distinct at first glance from the acute to shortly acuminate leaves of *R.hookeriana* there is considerable variation in the various sheets of this collection at BM, K and OXF with varying degrees of intermixture. K-000529252 has lower leaves shortly acuminate and apiculate, but the upper leaves rounded and cuspidate at apex. K-000529384 has some leaves acute and some obtuse and mucronate. No other distinguishing characters are discernible. It seems difficult to maintain *Rhytiglossacuspidulata* Nees and *R.hookeriana* as distinct taxa except possibly as forms so we propose they are treated under the oldest available name in *Justicia*, which is *J.cuspidulata* (Nees) Wassh., *J.hookeriana* being already occupied as *J.hookeriana* (Nees) T. Anderson from Sri Lanka.

Ezcurra (2002) equated *R.cuspidulata* with plants of the Chaco but Wasshausen & Wood showed that the Chaco plants were a distinct species, *Justiciapraetermissa* Wassh. & Wood (Wasshausen & Wood 2003).

Curiously, this plant was not recollected for over 150 years before David Aybar re-found it in March 2024. Photographs of this newly rediscovered species are shown in Fig. 3.

### ﻿﻿3. Justicia chimboracensis

Taxon classificationPlantaeLamialesAcanthaceae

Wassh., Fl. Ecuador 89: 138. 2013. (Wasshausen 2013: 138)

BAC4F123-DD4C-57FA-B71B-C5C65CF8D39F

Justicia loxensis Wassh., Fl. Ecuador 89: 138. 2013. (Wasshausen 2013: 138) syn. nov. Type. ECUADOR. Loja, road from Loja to Zaruma, between Chinches and Sambi, *Harling & Andersson* 14236 (holotype GB-14236, isotype US-01106144).

#### Type.

Ecuador • Chimborazo, Canyon of Río Chanchan, 5 km N of Huigra, *W.H. Camp* E-3285 (holotype US-01106141, isotypes CAS-560569, COL, K-000543842, M-0244152, MO-3085139, NY-2685706, P-04023385, S-12-734, US-02878220).

#### Description.

Perennial herb resembling *J.alpina* and *J.cuspidulata* in habit, shortly petiolate leaves and terminal spicate inflorescence with 4-lobed calyx, large reddish corollas and weakly superposed, basally muticous anther thecae. It is distinguished by the larger, 3.5–9 (–14) × 1.5–5 (–6.5) cm, ovate to oblong-elliptic leaves, which are apically acute to shortly acuminate and basally attenuate and decurrent onto petioles 0.5–2 cm long; also by the generally shorter, laxer spikes 4–10 cm long which usually scarcely exceed the subtending leaf pair, by the calyx lobes filiform apically, ± equalling the bracteoles and by the distinctly larger corolla 2.8–4.5 cm long, which lacks “herring bone” patterning.

#### Illustration.

**Figure 4.**
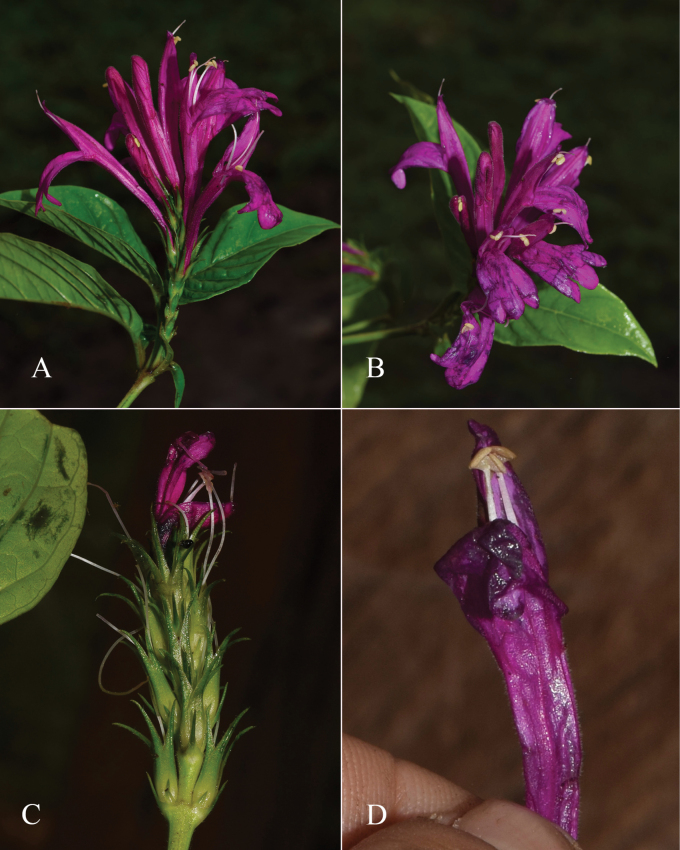
Photographs of *Justiciachimboracensis*. Note the absence of “herring bone” patterning and the muticous thecae held at different angles. Photographs by Rosa Villanueva

Fig. 4; Wasshausen (2013: 127, 139); Sagástegui-Alva et al. (2003: 52).

#### Phenology.

Flowers from February to July.

#### Habitat.

1400–2000(–2500) m, scrubby banks.

#### Distribution.

Southern Ecuador and northwest Peru in the departments of Amazonas, Cajamarca and Lambayeque. Fig. 55.

#### Material examined.

**Peru** • **Amazonas**: Prov. Chachapoyas, 11 km E of Chachapoyas on road to Molinopampa, 1850 m, 23 Feb. 1978, *D.C. Wasshausen & F. Encarnación* 980 (K, US); • ibid., Chachapoyas, outside town on road to Mendoza, 1500 m, 10 April 2001, *H. van der Werff et al.* 16872 (USM); Prov. Bongara, Río Utcubamba, 18–19 km below Caclic, 1500 m, 15 June 1964, *P.C. Hutchison & J.K. Wright* 5857 (USM); • ibid., road from Puente Inferno to Chachapoyas, Río Utcubamba, 1420 m, 25 Feb. 1976, *T. Plowman* 5556 (USM); • ibid., km 19 Pedro Ruiz a Chachapoyas, Oct. 1990, *F. Kahn & F. Moussa* 2745 (USM); • ibid., Dist. Churuha, Catarata Ashpachaca, entre Pedro Ruiz y Chachapoyas, 6°00'48.5"S, 77°55'02.3"W, 512 m, 2 May 2024, *D. Aybar* 009 (MOL); Prov. Luya, Dist. El Tingo, entre Cacle y Pedro Ruiz, 1470 m, 7 July 2001, *I. Sánchez Vega & A. Delgado Salinas* 10813 (CPUN, US). • **Cajamarca**: Prov. Contumazá, 28 km below Contumazá towards Cascas, 7°25'S, 78°25'W, 1835–1900 m, 14 April 1986, *M.O. Dillon et al.*4506 (BM, MO); • ibid., La Montaña (Guzmango-Contumazá), 2500 m, 18 May 1979, *A. Sagástegui et al.* 9310 (HUT, US); • ibid., Dist. San Benito, Andaloy, San Benito–Yeton, 2000 m, 23 March 1988, *A. Sagástegui et al.* 13047 (F, HUT, MO, US). Prov. Jaén, 500 m, 27 March 1960, *F. Woytkowski* 5606 (US). • **La Libertad**: Prov. Otuzco, entre Huaranchal and Chuiquizongo, 2000 m., 8 June 1958, *A. López et al.* 2706 (HUT, US). Prov. Gran Chimú, Corlas (Cascas–Contumazá), 1400 m, 16 Feb. 1995, *A. Sagástegui & S. Leiva* 15512 (F, MO). • **Lambayeque**: road to Jaén, 38.6 km east of Olmos, west side of Abra Porculla [5°51'S, 79°30'W], 1570 m, 14 March 1964 *P.C. Hutchison & J.K. Wright* 4431 (MO, US, USM).

#### Notes.

This plant is somewhat variable. At one extreme, *Sagástegui et al.* 13047 has a corolla 4–4.5 cm long and obscurely puberulent calyx lobes, thus fitting *J.loxensis* rather well. At the other extreme, *Wasshausen & Encarnación* 980 has a shorter corolla c. 3.5 cm long, similar to the type of *J.chimboracensis*, but with a pubescent calyx. Other specimens lie between the two extremes.

Specimens of this plant collected in Peru were generally identified in herbaria as *Justiciachachapoyasensis* (*Rhytiglossahookeriana*), possibly because it grows near Chachapoyas. The species appear to be related but the inflorescence, corolla and calyx lobes are different.

### ﻿﻿4. Justicia rojasiae

Taxon classificationPlantaeLamialesAcanthaceae

R.Villanueva & J.R.I.Wood sp. nov.

807A31C9-F3DD-5D02-B3C9-12EDEF4BDBFF

urn:lsid:ipni.org:names:77363405-1

#### Type.

Peru • Pasco, Prov. Oxapampa, Dist. Oxapampa, Parque Nacional Yanachaga-Chemillén. Quebrada Yanachaga, 10°24'S, 75°28'W, 2250 m, 14 June 2003, *R. Vásquez* 28272 (holotype MO-7066511, isotypes HOXA, US, USM).

#### Diagnosis.

Similar to *Justiciacuspidulata* in the 4-lobed calyx, red corolla and spicate inflorescence with flowers in opposite pairs and reduced bracts resembling the bracteoles, but leaves narrowly oblong-ovate, 2–3 times as long as broad (not ovate-elliptic, scarcely longer than broad), petioles 2–4 mm (not mostly > 10 mm), inflorescence long-pedunculate, lower flower pairs distant, corolla lips relatively short (3–5 mm long, not 10 mm), calyx lobes c. 9 mm long (not 7–8 mm); it also resembles *J.alpina* but the leaves are narrowly oblong-ovate, 2–3 times as long as broad, (not ovate, scarcely longer than broad), distinctly petiolate (not subsessile) and spikes much shorter, 4–5 cm long (not 6–20 cm).

#### Description.

Perennial herb reaching at least 50 cm in height; stem obscurely 4-angled, subglabrous to obscurely bifariously scurfy-puberulent. Leaves shortly petiolate, lamina mostly 4–7 × 1.3–2 cm, narrowly oblong-ovate, apex acuminate to an obtuse apex, base cuneate, slightly oblique, both surfaces glabrous and lacking prominent cystoliths, veins 4–5 pairs; petioles 2–4 mm, glabrous. Inflorescence of lax terminal spikes, 4–5 cm long, the flowers sessile, solitary or paired, opposite along the rhachis, up to 1.5 cm distant; peduncles 1.5–3.5 cm long, bifariously pubescent; rhachis glandular-pubescent; bracts linear 3–4 × 1 mm, glabrous or thinly (glandular-)pubescent; bracteoles 4–5 × 0.5 mm, linear, sparsely pubescent; calyx 4-lobed to base, lobes 9–10 × 0.5 mm at anthesis, linear, acuminate, sparsely pubescent with multicellular, sometimes gland-tipped hairs; corolla pink, pubescent on the exterior, 2.5 cm long; tube gradually widened from 1 mm at base to 5 mm at mouth, 2-lipped, upper lip c. 1.5–3 mm long, notched, lower lip very shallowly lobed, lobes c. 3 mm long, ovate, rounded; filaments glabrous, white, anther thecae broadly oblong, 1.25 × 1 mm, held at same height, glabrous, lacking appendages; pollen prolate, 39–43 × 23–24 μm, 2-aperturate, colporate, one row of c. 9–12 insulae and a second row of peninsulae on either side of aperture (Fig. 48B); ovary and style glabrous. Capsule and seeds not seen.

#### Illustration.

Figs 5, 6A, B.

**Figure 5.**
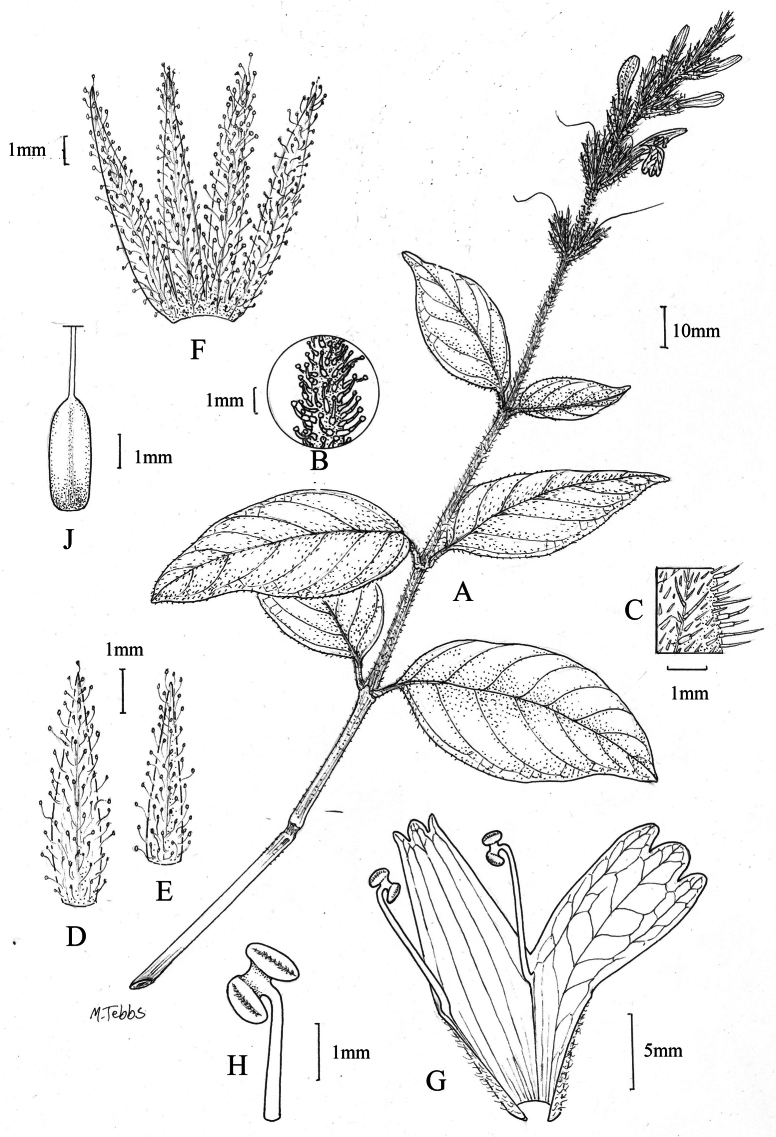
*Justiciarojasiae***A** habit **B** detail of stem **C** detail of adaxial leaf surface **D** bract **E** bracteole **F** calyx **G** corolla opened out **H** anther **J** ovary. Drawn from *Vásquez* 28272 by Margaret Tebbs

**Figure 6.**
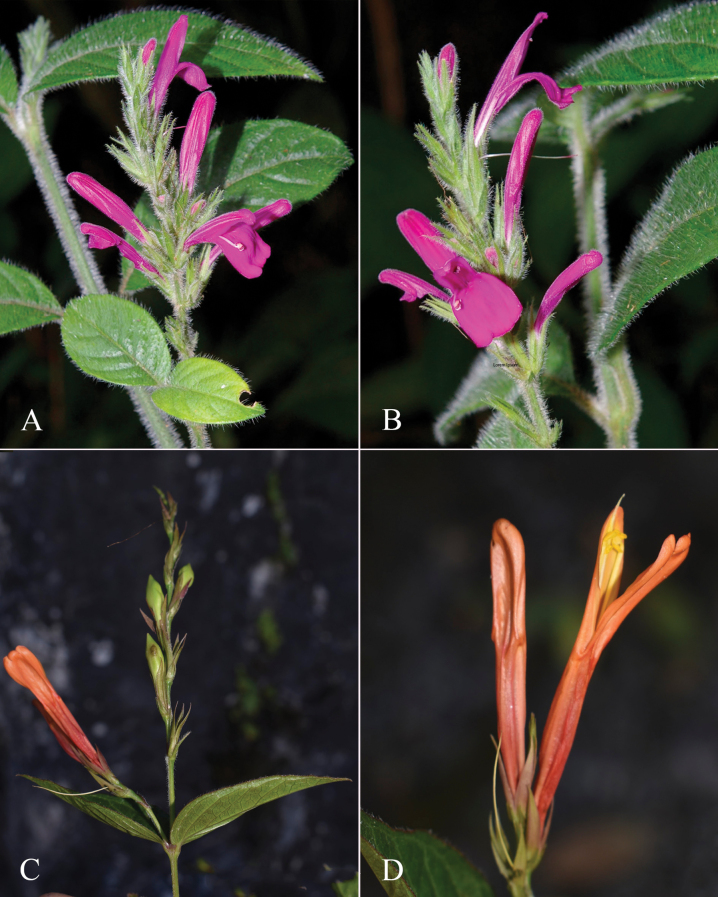
Photographs of **A**, **B***Justiciarojasiae* (*Vásquez* 36460). Note the indumentum and absence of “herring bone” patterning. **C**, **D***Justiciapozuzoensis* (*Villanueva* 925). Note the alternate flowers. **A**, **B** Rodolfo Vásquez, **C**, **D** Rosa Villanueva.

#### Etymology.

This species is named after Rocio Rojas who has contributed to our knowledge of Peru’s flora by numerous records, many cited in this paper.

#### Phenology.

Flowers from April to June.

#### Habitat.

Forest and forest relics, 1900–2250 m.

#### Distribution.

Endemic to the area around the Yanachaga-Chemillén National Park in Oxapampa Province, Pasco Department. Fig. 54.

#### Material examined.

**Peru** • **Pasco**: Prov. Oxapampa, Dist. Chontabamba, Ecolodge Ulcumano 10°38'08"S, 75°25'37"W, 2244 m, 10 May 2021, *R. Vásquez et al.* 45620 (HOXA, USM); • ibid. La Suiza Vieja, 10°38'47"S, 75°30'20"W, 2050 m, 18 June 2004, *R. Rojas et al*. 2936 (HOXA, MO); • ibid., 10°38'34"S, 75°27'31"W, 2000–2200 m, 23 June 2004, *R. Rojas et al*. 3055 (HOXA, MO); • ibid., 10°38'S, 75°40'W, 1900 m, 16 April 2010, *R. Vásquez et al*. 36460 (HOXA, MOL, MO, US, USM); • ibid., Dist. Oxapampa, P. N. Yanachaga-Chemillén, the type.

#### Note.

*R. Rojas et al.* 1174 (MO) from Oxapampa, Dist. Pozuzo, Puesto de Control, Huampal (10°11'S, 75°34'W) at 1100 m appears somewhat similar but has a much denser inflorescence.

### ﻿﻿5. Justicia pozuzoensis

Taxon classificationPlantaeLamialesAcanthaceae

Wassh., Monogr. Syst. Bot. Missouri Bot. Gard. 45: 1253.1993. (Wasshausen 1993: 1253)

23A52EC7-DAED-51B1-B265-E9CF8E494EFB

Jacobinia weberbaueri Lindau, Notizbl. Bot. Gart. Berlin-Dahlem 8: 246. 1922. (Lindau 1922: 246) Type. PERU. Pasco, Prov. Pozuzo, *A. Weberbauer* 6749 (presumed holotype B†, isotypes F-0040507F, GH-00094055, MOL-00005726, MOL-00005727, MOL-00005728, US-02882985).

#### Type.

Based on *Jacobiniaweberbaueri*.

#### Description.

Subshrub to 2 m in height, stems bifariously puberulent. Leaves subsessile or shortly petiolate, lamina 5–10 × 2–3.5 cm, ovate, acuminate, base subcordate, rounded to cuneate, glabrous except ciliolate margin, both surfaces with cystoliths; petioles 0–7 mm long. Inflorescence of solitary or (rarely) branched terminal lax spikes up to 15 cm long, occasionally also arising in the upper leaf axils, the flowers solitary, up to 15 mm apart; bracts 6 × 0.5 mm, puberulent, sterile below; calyx 4-lobed, lobes 12–15 × 1.5 mm, pubescent below, glabrous apically; corolla 4–4.9 cm long, orange, puberulent, tube subcylindrical 3 mm wide at base, widened to 6 mm above, upper lip 21 × 10 mm, bilobed, lower lip 19 × 9 mm, 3-lobed, lobes emarginate; filaments c.22 mm long, anther thecae 2 mm long, superposed, muticous; pollen prolate, 37.5–45 × 25–30 μm, 2-aperturate, colporate, two irregular rows of 6–9 insulae on either side of the aperture, the outer row partially peninsulae (Fig. 48C); ovary 2.5 mm high, glabrous. Capsule 18–20 × 5–6 mm, glabrous, clavate, the base sterile, 4-seeded; seeds c. 3 mm, rounded, verruculose.

#### Illustration.

Figs 6, 7.

**Figure 7.**
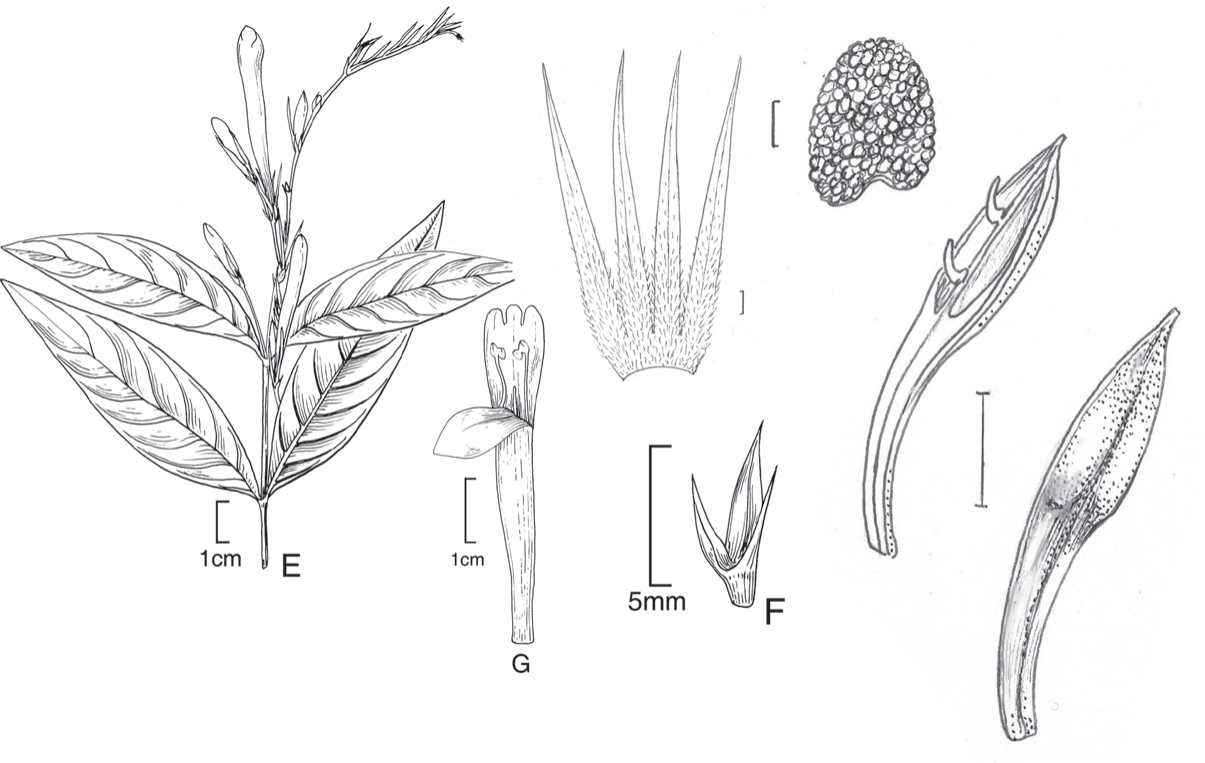
*Justiciapozuzoensis***A** habit **B** bracts and bracteoles **C** calyx **D** corolla opened out showing lips and androecium **E** capsule valves, exterior (right), interior (left) **F** seed. Drawn from *MacBride* 3722, **A, B**, **D** by Cathy Pasquale; **C**, **E**, **F** by Margaret Tebbs

#### Phenology.

Flowers from May to August.

#### Habitat.

Steep forested slopes and roadsides, c. 600 to 1800 m.

#### Distribution.

A rare species endemic to central Peru. Most records are from near Pozuzo. Fig. 55.

#### Material examined.

**Peru** • [**Huánuco**]: Prov. Pachitea, Dist. Chaglla, Yanano [9°50'S, 75°54'W approx.], 6000 ft, 13–16 May 1923, *J.F. MacBride* 3722 (F, US). • **Junín**: Prov. Chanchamayo, San Ramón, Between Lourdes de Oxabamba and Nueva Italia, 11°3"36.5"S, 75°24'18.2"W, 1120 m, 3 Aug. 2023, *R. Villanueva et al.* 925 (HOXA); • ibid., carretera a la Promisoria, pasando Lourdes, 11°3"59.4"S, 75°24'08.7"W, 22 March 2022, *C. Reynel* 22-049 (MOL); • ibid., 28 March 2022, *C. Reynel* 22-112 (MOL). • **Pasco**: Prov. Oxapampa, Dist. Pozuzo, the type; • ibid., Huacabamba–Pozuzo, Cañón de Huacabamba, below Río Tunqui, 10°10'S, 75°35'W, 1000–1500 m, 30 June 1985, *R. Foster et al.* 10367 (F, MOL, USM, US); • ibid., Dist. Pozuzo, 2000 ft, 20–22 June 1923, *J.F. Macbride* 4700 (F, US); • ibid., Dist. Huancabamba, 10°05'42"S, 75°55'8"W. 1777 m, *Xue-Jun Ge et al.* 497 (USM); • ibid., *Xue-Jun Ge et al.* 503 (USM).

#### Conservation.

This species was assessed as EN, B1ab(iii) following IUCN (2024) guidelines (León 2006). However, this categorization is likely to have been premature as fewer records were known at the time and no evaluation of the populations or habitats was carried out.

### ﻿﻿6. Justicia oppositiflora

Taxon classificationPlantaeLamialesAcanthaceae

R.Villanueva & J.R.I.Wood sp. nov.

94171130-DD48-5842-9793-7E088CB17CB9

urn:lsid:ipni.org:names:77363406-1

#### Type.

Peru • Pasco, Prov. Oxapampa, Dist. Huancabamba, Río Yanachaga drainage, Hac. Yanachaga, 10°32'S, 75°32'W, 2240–2260 m, 26 May 1983, *D.N. Smith & G.Pretel* 4189 (holotype MO-3507877, isotypes F-1992314, US-3123730, USM).

#### Diagnosis.

This species is closely related to *Justiciapozuzoensis* but the flowers are arranged in opposite pairs (not solitary), the calyx lobes 6–8 (–11) mm (not 12–15 mm) long and the corolla 3.2–3.9 cm long (not 4–4.9 cm) long. It also bears a close resemblance to *J.novagranatensis* Leonard in having somewhat lax elongate spikes of opposite flowers with similar floral dimensions but differs in the very shallowly lobed lower corolla lip (not deeply lobed to 7 mm).

#### Description.

Subshrub to 1.5 m (–5 m) in height; stems ± glabrous, occasionally obscurely bifariously hirtellous below nodes, sometimes caniculate. Leaves shortly petiolate; lamina 3.5–12 × 0.8–3 cm, lanceolate to narrowly oblong-elliptic, apex acuminate to a fine point, base cuneate, sometimes slightly oblique, margin entire to obscurely undulate, both surfaces glabrous or obscurely hirtellous on the midvein near the base, adaxially with cystoliths, abaxially paler, gland-dotted, venation not prominent, lateral veins c. 6–7; petioles 2–4 mm, deeply channelled, glabrous. Inflorescence of lax spikes, both terminal and from the upper leaf axils; flowers in distant opposite pairs, up to 2 cm apart below, but closer above; spikes 5–9 cm long; rhachis glabrous to obscurely bifariously hirtellous; floral bracts 4–5 × 1–1.5 mm, ovate-deltoid, acuminate, glabrous to shortly puberulent and with prominent cystoliths; bracteoles lanceolate-deltoid 5–6 × 1 mm, glabrous to shortly puberulent; calyx subequally 4-lobed, lobes lanceolate, finely acuminate, 6 × 0.75 mm at anthesis but reaching 11 × 1.25 mm in fruit, pubescent; corolla 3.2–3.9 cm long, red, glabrous, 2-lipped, tube 1.8–2.2 cm long, gradually widened from a narrow base, c. 1.5 mm wide, to 4–5 mm at mouth, strongly 2-lipped, upper lip bifid, 1.5–2 cm long, lower lip slightly shorter, shallowly 3-lobed, lobes subequal, ovate, rounded c. 1 × 1 mm; filaments glabrous, white, c. 2.2 cm long, anther thecae superposed, glabrous, 1.25–1.5 × 0.5 mm, both with a basal appendage; pollen prolate, 47–57 × 25 μm, 2-aperturate, colporate, 1 row of c. 7–10 insulae and 1 row of peninsulae (Fig. 48D); style c. 4 cm long, glabrous; ovary c. 2 mm high, conical, glabrous. Capsule 15.3 mm long, glabrous, clavate, 4-seeded; seeds 2 mm long.

#### Illustration.

Fig. 8.

**Figure 8.**
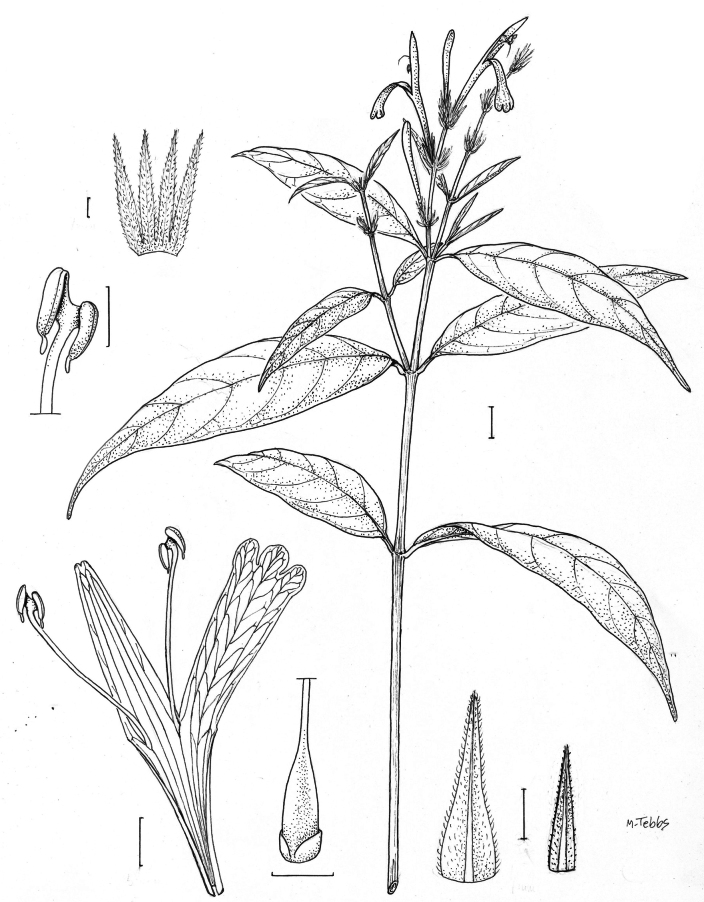
*Justiciaoppositiflora***A** habit **B** bract **C** bracteole **D** calyx **E** corolla opened out to show lips and androecium **F** anther thecae **G** ovary. Drawn from *Smith & Pretel* 4189 by Margaret Tebbs.

#### Etymology.

The epithet “*oppositiflora*” refers to the arrangement of the flowers in the inflorescence, the opposite flowers being an important distinction from *Justiciapozuzoensis*.

#### Phenology.

Found in flower in January and May to August.

#### Habitat.

Primary and secondary forest. 2100–2250 m.

#### Distribution.

Apparently endemic to the Yanachaga-Chemillén National Park in Peru. Fig. 55.

#### Material examined.

**Peru** • Sine loc. *McLean* s.n. (K). **Pasco**: Prov. Oxapampa, the type collection; • ibid., P.N. Yanachaga-Chemillén, Sector Quebrada Yanachaga, 10°24'13"S, 75°29'04"W, 2200 m, 14 Jan. 2005, *R. Vásquez et al*. 30641 (MO); • ibid., Sector Palcazú–Alto Navarra, 10°16’'S, 75°15'W, 2100 m, 24 Aug. 2005, *R. Rojas* 3846 (HOXA, MO, US); • ibid., P.N. Yanachaga-Chemillén, Quebrada Yanachaga, 10°24'44"S, 75°28'56"W, 2250 m, 14 June 2003, *R. Vásquez et al.* 28287 (US, USM).

#### Note.

This species is very close to *Justiciapozuzoensis* differing in little more than the diagnostic characters.

Species 7. Small flowered species with 4-lobed calyx and inflorescence of terminal spikes.

#### ﻿﻿7. Justicia discolor

Taxon classificationPlantaeLamialesAcanthaceae

J.R.I.Wood & R.Villanueva sp. nov.

9D42381A-8DEF-5C96-BDCA-90776934C26E

urn:lsid:ipni.org:names:77363407-1

##### Type.

Peru • Junín, Prov. Satipo, Dist. Río Negro, 800 m, 12 Aug. 1960, *F. Woytkowski* 5790 (holotype US-2960983, isotypes MO-2923588, US-2426094).

##### Diagnosis.

Slender perennial herb with 4-lobed calyx and terminal inflorescence resembling *Justiciaboliviana* Rusby and allies but distinguished by the discolorous, nearly glabrous leaves, the glandular-pilose inflorescence, linear calyx lobes 7.5–9 × 0.25–0.5 mm, small glabrous corolla 10–12 mm long and thinly pubescent capsule.

##### Description.

Perennial herb; stems creeping, rooting at the nodes, eventually ascending to c. 50 cm, striate, glabrous to thinly hirtellous. Leaves equal to slightly unequal in each pair, shortly petiolate; lamina 1.5–9.5 × 0.6–4 cm, very variable in shape even on the same plant, commonly lanceolate to ovate, usually acuminate to an obtuse apex, base broadly to narrowly cuneate, sometimes slightly decurrent onto the petiole, margin undulate to conspicuously crenate, veins prominent, c. 5–6 pairs, discolorous, adaxially green with abundant cystoliths, abaxially usually conspicuously dark violet, both surfaces glabrous; petioles 3–7 mm, subglabrous to hirtellous. Inflorescence a terminal pedunculate spike, often solitary but sometimes with 1–2 secondary spikes from the uppermost leaf axils, spikes 1.5–6 (–14) cm long; peduncles 1–3.5 (–6) cm, thinly to densely glandular-pilose, rhachis glandular pilose, flowers in opposite pairs, c. 8 mm apart below, imbricate above; bracts 4–5 × 0.5 mm, linear-lanceolate, glandular-pilose; bracteoles similar but 2–3 × 0.25 mm; calyx 4-lobed to base, lobes 7.5–9 × 0.25–0.5 mm linear to filiform, acuminate, accrescent in fruit, glandular-pilose; corolla lilac, purple or violet with white “herring bone” patterning, glabrous, 10–12 mm long, 2-lipped, tube c. 6 × 1.5 mm, white or purplish, upper lip 2–4 mm long, entire, lower lip c. 6 mm long, shallowly 3-lobed, lobes c. 1 mm long, ovate, rounded; filaments glabrous, anther thecae shortly oblong, 0.5 × 0.25 mm, superposed, glabrous, lacking basal appendages; pollen prolate, 35 × 21–22 μm, 2-aperturate, colporate, 1 distinct band of sexine on either side of aperture (Fig. 48E); style 9–10 mm long, glabrous. Capsule 6–8 × 1.5–2 mm, narrowly clavate, apiculate, thinly glandular-pubescent, 4-seeded; seeds 1 × 1.25 mm, cordate-ovoid, flattened, rugose, glabrous, reddish-brown.

Divisable into two geographical subspecies:

#### ﻿﻿7a. Justicia discolor subsp. discolor

Taxon classificationPlantaeLamialesAcanthaceae

82B2ECFF-B802-5AF9-AFB9-70A0A1CCEE6B

##### Diagnosis.

Closely related to Justiciadiscolorsubsp.filisepala but more robust, the leaves discolorous, purple abaxially, lanceolate or ovate, acuminate, firm in texture, 1.5–9.5 × 0.6–4 cm, mostly more than 3 times as long as broad (not discolorous or purple abaxially, elliptic, obtuse, thick in texture, 2–5 (–6) × 1–4 cm, c. twice as long as broad), inflorescence imbricate above, the flower pairs c. 8 mm apart below, (not up to 22 mm apart in fruit); calyx lobes linear (not filiform).

##### Illustration.

Figs 9–10.

**Figure 9.**
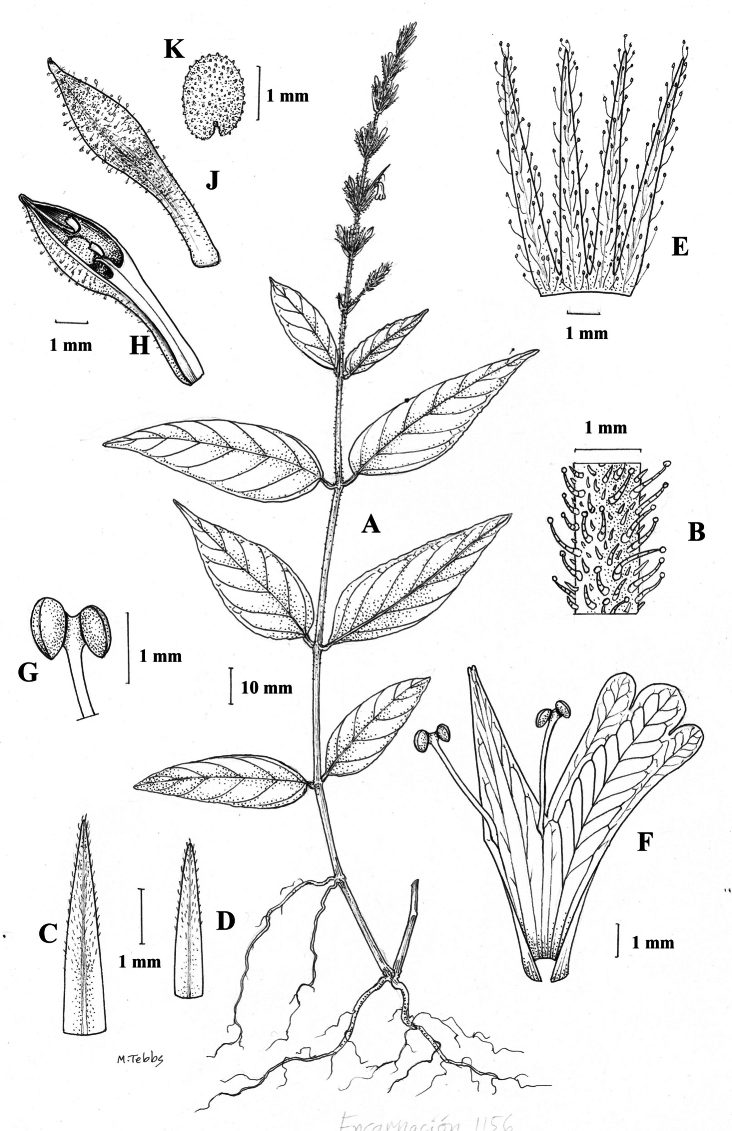
Justiciadiscolorsubspdiscolor**A** habit **B** section of stem showing indumentum **C** bract **D** bracteole **E** calyx **F** corolla opened out showing lips and androecium **G** anther thecae **H** capsule valves, exterior (right), interior (left) **J** seed. Drawn from *Encarnación* 1156 by Margaret Tebbs.

**Figure 10.**
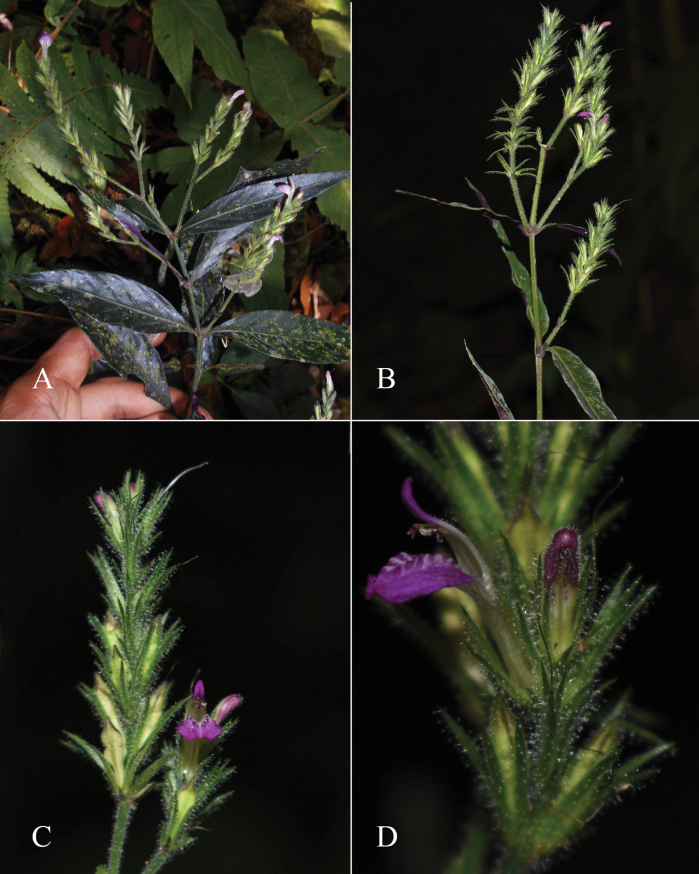
Photographs of *Justiciadiscolor*. Note glandular inflorescence and violet corolla with “herring bone” patterning **A** (*Azevedo* 162) Igor Azevedo **B–D** (*Villanueva* 976) Rosa Villanueva.

##### Etymology.

The name “*discolor*” refers to the leaves which are characteristically green on the upper surface but purple on the lower surface.

##### Phenology.

Flowering from May to August with a single record from December.

##### Habitat.

Rainforest, “bosque alto”, 300–900 m.

##### Distribution.

Endemic to Amazonian Peru, widespread but scattered in occurrence. Fig. 54.

##### Material examined.

**Peru** • **Huánuco**: Prov. Leoncio Prado, E of Las Palmas, 15 km S of Tingo María, 900 m, 20 June 1982, *D.C. Wasshausen & O. Tovar* 1275 (K, US); • ibid., Dist. Luyando, Tulumayo, July 1938, *C.A. Ridoutt* s.n. (USM 10003); • ibid., Dist. Rupa-Rupa, Jacintillo, left margin of Río Monzón, 18 July 1978, *J. Schunke Vigo* 10373 (MO, US); • ibid., Tingo María, 9°18'S, 75°59'W, 700–780 m, 9 Dec. 1981, *T. Plowman et al.* 11251 (F, US); • ibid., Fundo El Encanto, 700 m, 15 Aug. 1943, *C.A. Ridoutt* USM 13149 (USM). Prov. Puerto Inca [Pachitea], Dist. Honoria, carretera Miel de Abejas (1 km arriba de Tornavista) a 3 km del Campamento, 300–400 m, 19 May 1967, *J. Schunke V.* 1969 (F); • ibid., Dist. Codo de Pozuzo, Carretera Pozuzo-Codo del Pozuzo, 28 Aug. 2019, *I. Azevedo & R. Villanueva* 162 (HOXA). • **Junín**: Prov. Satipo, the type; • ibid. 17 Aug. 1960, *F. Woytkowski* 5824 (MO, US). • **Madre de Dios**: Prov. Manu, Manu Park, Cocha Cashu uplands, 11°45'S, 71°0'W, 400 m, 28 July 1986, *P. Nuñez* 5521 (CUZ, MO); • ibid. 11°45'S, 71°00'W, 19 Aug. 1986, *P. Nuñez* 5848 (MO, USM); Prov. Tahuamanu, 8 km del Fundo Noaya, hacia la Quebrada Putirija, 29 May 1978, *F. Encarnación* 1156 (US). • **San Martin**: Prov. Mariscal Cáceres, Dist. [Dept.] Uchiza, Cachiyacu de Lepuna, 450–500 m, 10 July 1974, *J. Schunke V.* 7298 (F, MO, USM). • **Ucayali**: Prov. Coronel Portillo, Ivita, km 59 Carretera Federico Basadre, 17 July 1974, *F. Encarnación* 432 (US). Prov. Padre Abad, Dist. Padre Abad, Catarata Santa Rosa, 9°09'S 75°45'W, 882 m, 8 Aug. 2023, *R. Villanueva et al.* 976 (HOXA).

##### Note.

Usually recognisable even when sterile by the discolorous leaves.

#### ﻿﻿7b. Justicia discolor subsp. filisepala

Taxon classificationPlantaeLamialesAcanthaceae

J.R.I.Wood & R.Villanueva subsp. nov.

B81DEA33-5904-52CA-9446-361CE392E5BE

urn:lsid:ipni.org:names:77363408-1

##### Type.

**Peru** • **Loreto**, Prov. Maynas, Dist. Mazán, 6 km from Fundo Buenas Aires, Río Tamishiyacu, 20 June 1977, *F. Encarnación* 1107 (holotype K-000544764, isotype US-2956915).

##### Diagnosis.

Closely related to Justiciadiscolorsubsp.discolor but a slender herb, the leaves concolorous, elliptic, obtuse, thin in texture, 2–5 (–6) × 1–4 cm, c. twice as long as broad, glabrous or thinly pilose, (not discolorous, purple abaxially, lanceolate or ovate, acuminate, relatively firm in texture, 1.5–9.5 × 0.6–4 cm, mostly more than 3 times as long as broad); inflorescence lax, the flower pairs up to 22 mm apart (not imbricate, not more than 10 mm apart); calyx lobes filiform (not linear).

##### Description.

Slender near isophyllous creeping herb, rooting at nodes; stems to 25 cm, bifariously crisped-pubescent, glabrescent when old, cystoliths prominent and abundant. Leaves petiolate, lamina 2–5 (–6) × 1–4 cm, broadly ovate, oblong-ovate, to elliptic, apex obtuse to rounded, base broadly cuneate, margin undulate to weakly crenate, glabrous or thinly pilose adaxially with multicellular hairs, cystoliths abundant on both surfaces, slightly paler beneath; petioles 3–4 mm. Inflorescence of short, pedunculate terminal spikes, 1–3 cm long, elongating to 8 (–14) cm in fruit, usually solitary but occasionally with a small secondary spike from the uppermost leaf axils; flowers relatively distant, up to 22 mm apart at base of spike; peduncles 1–1.8 cm at anthesis but up to 6 cm in fruit, shortly pilose with gland-tipped whitish hairs; rhachis shortly pilose with gland-tipped whitish hairs, the flowers in opposite pairs, ± imbricate with internodes up to 3 mm long at anthesis but up to 22 mm in fruit, bracts 4–5 × 0.5 mm, linear, mucronulate, shortly pilose with gland tipped hairs, cystoliths prominent; bracteoles similar but only c. 2.5 mm long; calyx subequally 4-lobed to base, lobes linear-filiform, 7.5–8 × 0.25–0.5 mm, accrescent in fruit to 10 mm long, shortly pilose with gland-tipped hairs; corolla pale lilac, glabrous externally, 11–12 mm long, upper lip c. 4 mm long, subentire, lower lip 3-lobed, c. 6 mm long, lobes ovate, rounded; stamens glabrous, anther thecae 0.5 × 0.25 mm, oblong, superposed, glabrous, the lower with an almost imperceptible basal appendage; pollen prolate, 33–36 × 21 μm, 2-aperturate, colporate, a distinct band of sexine on either side of aperture (Fig. 48F); style glabrous, c. 10 mm. Capsule 7–8 × 2 mm, clavate, apiculate, thinly pubescent, valves with prominent venation, strongly recurved after seeds are ejected, 4-seeded; seeds 1.25 × 1.5 mm, tuberculate.

##### Illustration.

Fig. 11A–J.

**Figure 11.**
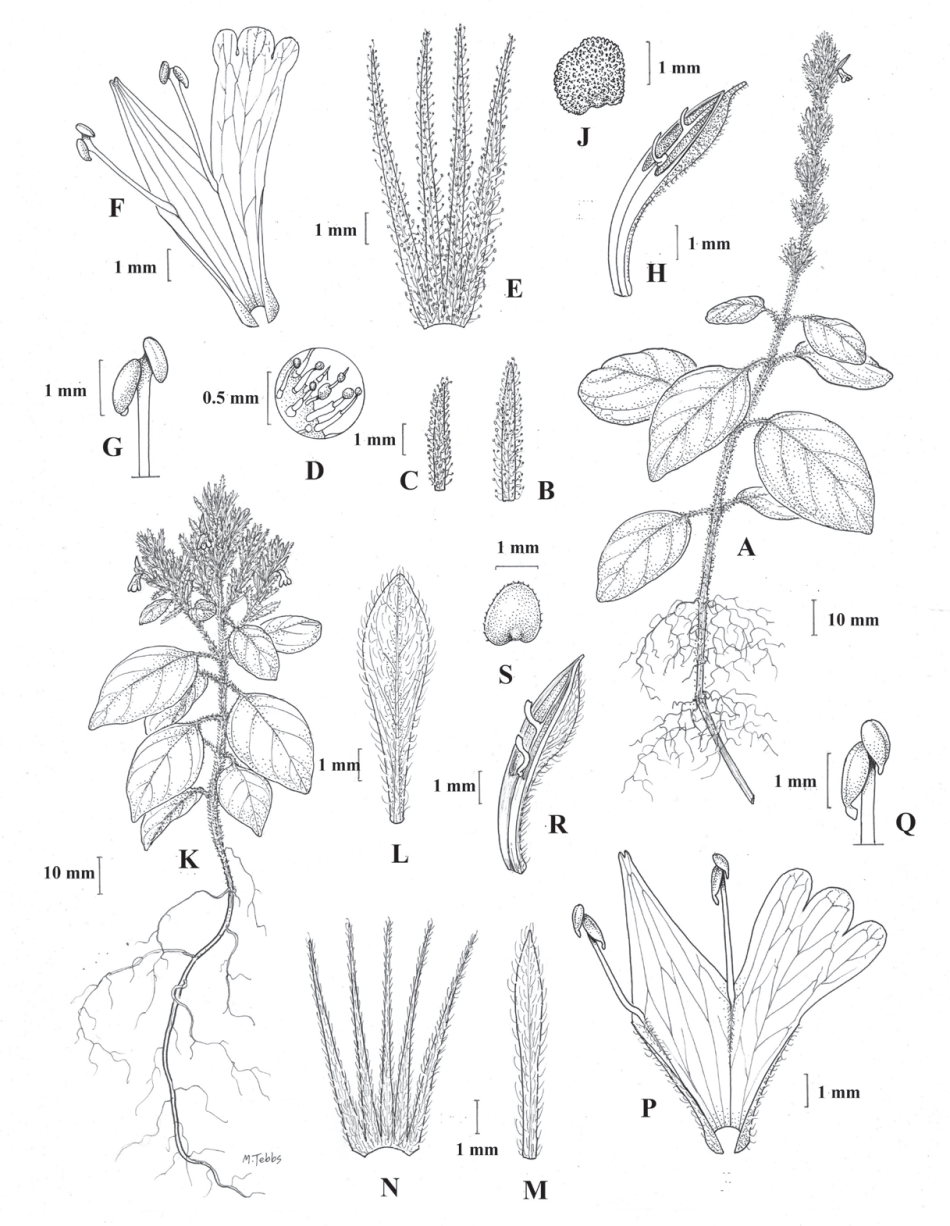
Justiciadiscolorsubsp.filisepala**A** habit **B** bract **C** bracteole **D** detail of indumentum **E** calyx **F** corolla **G** anther **H** capsule valve **J** seed. *Justiciachamaecaulis***K** habit **L** bract **M** bracteole N calyx **P** corolla **Q** anther **R** capsule valve **S** seed. **A–J** drawn from *Encarnación* 1107 **K–S** from *Encarnación* 927 by Margaret Tebbs.

##### Etymology.

The name “*filisepala*” refers to the calyx lobes which are characteristically thread-like in this subspecies.

##### Phenology.

Found in flower in February, March and June.

##### Habitat.

Humid lowland rainforest, perhaps liable to flooding, 95–190 m.

##### Distribution.

Endemic to Amazonian Peru in Loreto. Fig. 54.

##### Material examined.

**Peru** • **Loreto**: Prov. Maynas, Gamitanacocha, Río Mazán, 100–125 m, 8 Feb. 1935, *J.M. Schunke* 192 (F, US, USM); • ibid., 6 km from Fundo Buenas Aires, Río Tamishiyacu, 20 June 1977, *F. Encarnación* 1107 (K, US). Prov. Mariscal Ramón Castilla, Río Yavari, 20 km río abajo de Angamos, Quebrada Curacinha, 5°03'05"S, 72°43'52"W, 95–190 m, 28 March 2003, *H. Beltrán et al.* 5429 (F, USM). 4

The following are more robust with concolorous leaves and might be seen as intermediate with subsp. discolor.

##### Additional material examined.

**Peru** • **Ayacucho**: Prov. La Mar, Río Catute, between Santa Rosa and Sanabamba, 700 m, 16 Sept. 1976, *D.C. Wasshausen & F. Encarnación* 1155 (US). **Loreto**, Región Amazonas, Caballococha, 3°55'S, 70°30'W, 106 m, 15 Aug. 1989, *R. Vásquez & N. Jaramillo* 12755 (MO, US).

﻿Species 8. This species is probably unrelated to the previous seven. It is similar to *Justiciadiscolor* in its low stature, terminal inflorescence and small corolla but the calyx is 5-lobed and the pollen 3- or possibly 4-aperturate.

#### ﻿﻿8. Justicia chamaecaulis

Taxon classificationPlantaeLamialesAcanthaceae

J.R.I.Wood & R.Villanueva sp. nov.

A1B8041D-BFE4-5482-8481-E1F94D1D7361

urn:lsid:ipni.org:names:77363409-1

##### Type.

Peru • Loreto, Maynas, Dist. San Juan Bautista, Peñanegra, 8 km de Iquitos, 19 Aug. 1976, *F. Encarnación* 927 (holotype K-000544765, isotypes US-2956902, USM)

##### Diagnosis.

Low herb, < 10 cm high, the short dense inflorescence usually with several spikes and calyx 5-lobed; somewhat similar to some forms of *Justiciapotarensis* (Bremek.) Wassh. in leaf shape, indumentum and pilose inflorescence but always < 10 cm high (not up 40 cm), the spikes 1–4 cm long (not 5–15 cm long), flowers densely imbricate (not clearly separated) and the bracts equalling or longer than the calyx (not shorter).

##### Description.

Small leafy herb < 10 cm high from a slender taproot with fibrous lateral branches; stem usually simple, villous with stiff white hairs. Leaves crowded below inflorescence, the internodes up to 12 mm, but usually much less, petiolate, lamina 1.5–7 × 1–2 cm, oblong-elliptic, apex obtuse, adaxially thinly pilose to glabrescent except pubescence along the mid vein, abaxially slightly paler, glabrous except for pubescence on the mid vein above petiole, punctate; petioles 3–13 mm, villous. Inflorescence of solitary terminal bracteate spikes, sometimes with a secondary spike from uppermost leaf axil; spikes 1–4 cm long, sessile, the flowers imbricate, bracts 9 × 2.5 mm, oblanceolate, obtuse, green with prominent veins, thinly pilose; bracteoles 8.3–13 × 1.25 mm, thinly pilose, oblanceolate; calyx 5-lobed to near base, lobes linear-filiform, attenuate, slightly unequal in width, 6–7 × 0.25–0.5 mm, thinly pilose; corolla c. 8 mm long, 2-lipped, tube c. 4 × 1.5 mm, white; lips purplish, upper lip lanceolate, notched; lower lip 4–5 × 5 mm, 3-lobed, the lobes rounded, thecae oblong, 1 × 0.25 mm, glabrous, strongly superposed, the lower with a basal appendage; pollen prolate, 38–41 × 23–26 μm, 3-aperturate (possibly 4-aperturate), colporate, a row of unbroken band of sexine on either side of aperture (Fig. 49A); style pilose. Capsule 7–8 × 2 mm, clavate, pubescent, 4-seeded; seeds 1.5 mm diam., rugose.

##### Illustration.

Fig. 11K–S.

##### Etymology.

The name “*chamaecaulis*” meaning ‘low stem’ refers to the low growing habit so characteristic of this plant.

##### Phenology.

Found in flower in February, July, August, and September.

##### Habitat.

Forest clearings and tracksides on white sand at low altitudes, 140–150 m.

##### Distribution.

Endemic to Peru and only known from the Iquitos area of Maynas in Loreto. Fig. 65.

##### Material examined.

**Peru** • **Loreto**: Prov. Maynas, Río Nanay, halfway between Santa María de Manay and Iquitos, 3°30'S, 73°30'W, 140 m, 23 Feb. 1981, *Al. Gentry et al*. 31643 (MO, US); • ibid., Dist. Iquitos, trail from Picuru (lower Río Maynas) to Río Mazan, *S. McDaniel* 21463 (US); Dist. San Juan Bautista, carretera Pena Negra a 7 km de Quisto Cocha, Río Itaya, 150 m, 18 Sept. 1981, *M. Rimachi* 5713 (US); • ibid., 8 km de Iquitos, 19 Aug. 1976, *F. Encarnación* 927 (K, US, USM); • ibid., Quistococha, vic. Iquitos, 140 m, 16 Nov. 1977, *Al. Gentry* 20723 (MO, US, USM); • ibid., Mishana, trail from village to Camp. 1, 3°50'S, 73°30'W, 140 m, 22 July 1980, *Al. Gentry et al.* 28936 (MO); • ibid., Dist. Alto Nanay, Santa María de Nanay, Mishana (Río Nanay), 5 Aug. 1990, *R. Vásquez et al.* 14167 (MO).

Species 9–16. The Appendiculata clade. *Justiciaaphelandroides*, *J.appendiculata* and *J.sanchezioides* certainly belong to the Appendiculata clade sensu Kiel et al. (2018). This clade comprises taxa from wet tropical forests that tend to be shrubs (1–3 m in height), with large (5.5–8.0 cm long), slender and conspicuous flowers, anther thecae that are nearly parallel with appendages small or absent, and seeds that are discoid with rugose testa.

#### ﻿﻿9. Justicia appendiculata

Taxon classificationPlantaeLamialesAcanthaceae

(Ruiz & Pav.) Vahl, Enum. Pl. 1:159. 1804. (Vahl 1804: 159)

E668B997-1696-5421-AB5B-37F6D9A75885

Dianthera appendiculata Ruiz & Pav., Fl. Peruv. Prodr.1: 12. 1798. (Ruiz and Pavón 1798: 12) Type. PERU. *Pavon* s.n. (lectotype MA-817205 designated here, isolectotypes BM-000992613, MA-815488, OXF-00194632).
Beloperone appendiculata (Ruiz & Pav.) Nees, Prodr. 11: 423. 1847. (Nees 1847b: 423)
Beloperone denudata Nees, Prodr. [A. P. de Candolle] 11: 423. 1847. (Nees 1847b: 423) 1847. Type. PERU. Loreto, Maynas, *Poeppig* 2017B (lectotype W, designated by Wasshausen and Wood (2004: 50)
Beloperone mathewsiana Nees, Prodr. [A. P. de Candolle] 11: 731. 1847. (Nees 1847b: 731) Type. PERU. Moyabamba, *A. Mathews* 1535 (holotype K-000529267, isotype OXF-00194398).
Beloperone pubinervia Lindau, Bull. Herb. Boissier 5(8): 675. 1897. (Lindau 1897: 675) Type. PERU. *Pavon* s.n. (presumed holotype B†, isotypes G00236307, US-2883208).

##### Type.

Based on *Diantheraappendiculata* Ruiz & Pav.

##### Description.

Shrub 1–3 m in height. Leaves very large and often overtopping the inflorescence, lamina 11–30 × 6–13 cm, elliptic, acuminate at both ends, long petiolate, glabrous, abaxially characteristically yellow-green with brown venation, lateral veins 14–17 pairs. Inflorescence of axillary and terminal spikes, individually short, < 5 cm long, forming a much branched compound inflorescence, the lateral spikes verticillate: inflorescence bracts large, foliose, yellow-green, ovate, 2.5–7 × 1–3.5 cm; floral bracts 1.4 × 0.7 mm, deltoid, yellow-green, pubescent; calyx 5-lobed, 5–6 mm long; corolla salmon-pink, very slender, 40–50 mm long, puberulent; anther thecae ellipsoid, 2 × 0.75 mm with a small white basal appendage, parallel, weakly superposed; pollen prolate 57 × 21 μm, 2-aperturate, colporate, 1 row of c. 8 insulae and a few poorly developed peninsulae on either side of the aperture (Fig. 49B, Kiel et al. 2018: 462). Capsule 15–17 × 4 mm, clavate, minutely glandular; seeds 4, rugose.

##### Illustration.

Fig. 12A, B.

**Figure 12.**
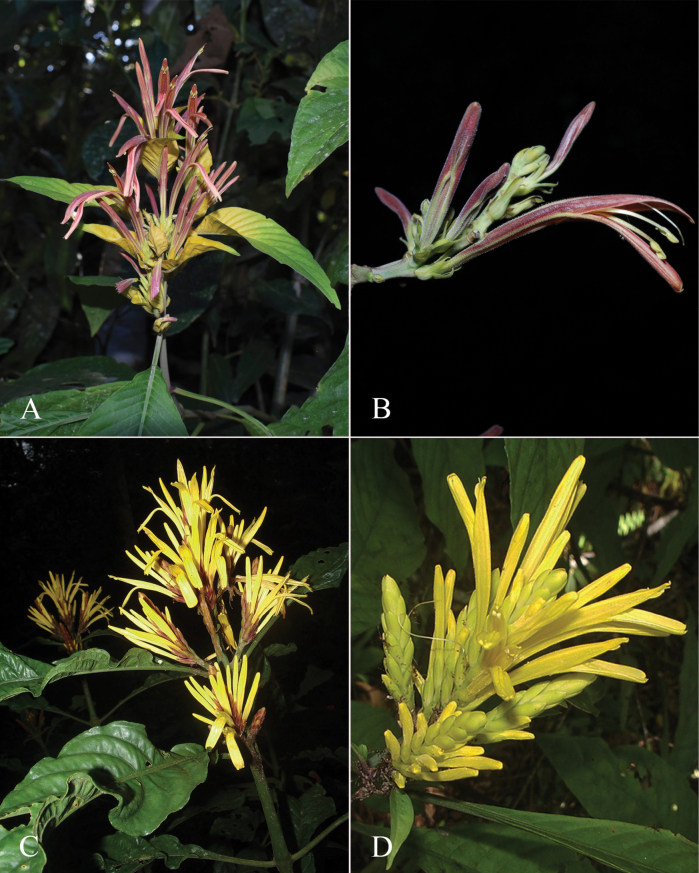
Photographs of **A, B***Justiciaappendiculata*. Note the distinctive whitish-green bracts. **C**–**D***Justiciarauhii*. Note distinctive yellow corollas. **A, B** Rosa Villanueva, **C, D** Isau Huamantupa.

##### Habitat.

Frequent in lowland rainforest up to c. 800 (–1500) m.

##### Phenology.

Flowers principally from May to September; reports of flowering outside this period are rare.

##### Distribution.

Eastern Andean slopes of Bolivia, Ecuador and Peru extending into the Amazonian lowlands and into Amazonian Brazil, but apparently absent from Colombia and northern Ecuador. Fig. 56.

##### Material examined.

**Peru** • **Ayacucho**: Río Apurimac Valley, near Kimpitiriki, 400 m, 10 May 1929, *E. P. Killip* 22944 (F, US); • ibid., 10–11 May 1929, *E.P. Killip* 23015 (F, US); Prov. La Mar, Cordillera, Vilcabamba, *T.R. Dudley* 9095 (NA); • ibid., Río Marantari, below Santa Rosa bridge, 580 m, 28 May 1975, *D.C. Wasshausen & F. Encarnación* 480 (K, US, USM). • **Cusco**: Prov. La Convención, *T.R, Dudley* 10256 (NA); • ibid., Dist. Pichari, Quempiri, caserío Campa, margen derecha del Río Ene, 460–480 m, 24 July 1965, *R. Ferreyra* 16374 (US, USM); • ibid., Dist. Echarate, plataforma de perforación de gas, Cashiriari 3, 8 July 2005, *S. Matías et al*. 6296 (USM); • ibid., Dist. Quiteni, 12°38'28"S, 73°04'18"W, 600 m, 19 July 2004, *W. Galiano et al.* 6713 (CUZ, FHO). Prov. Urubamba, alto Río Urubamba–Hac. Pigliato, Aug. 1925, *A. Diehl* 2426 (F); • ibid., Alto Manguriari, 700 m, *G. Ortiz* 33 (CUZ27869); *G. Ortiz* 17 (CUZ27853). • **Huánuco**: La Merced de Cachiyaquillo, 400–500 m, 14 Aug. 1948, *R. Ferreyra* 4480 (US, USM); • ibid., along road to Fundo San Juan at junction of Río Chinchao and Río Huallaga, 18 July 1962, *M. Mathias & D. Taylor* 5914 (F, USM). Prov. Pachitea, c.20 km to 24 km SE of Puerto Inca, 700 m, 31 July 1988, *B. Wallnofer* 117-31788 (US); • ibid., Dist. Honoria. Quebrada de Shahuinto, Bosque Nacional de Iparia, 200–400 m, 12 July 1978, *J. Schunke V.* 2114 (F, US). Prov. Leoncio Prado, Dist. Luyando, Tulumayo, entre Tingo María y Divisoría, 700–800 m, 5 Aug. 1947, *R. Ferreyra* 2137 (US, USM); • ibid., Hac. Shapajilla, cerca de Tingo María, 800 m, 10 Aug. 1946, *R. Ferreyra* 0894 (US, USM); • ibid., km 35 between Tingo María & Pucallpa, 1500 m, 3 June 1981, *G. Sullivan* 1168 (US); • ibid., Puente Tulumayo, cerca de Tingo María.700–750 m, 24 July 1948, *R. Ferreyra* 4345 (K, US, USM); • ibid., Dist. Mariano Damaso, Cayumba, entre Huánuco y Tingo María, 800–900 m, 15 July 1948, *R. Ferreyra* 4193 (MO, MOL, US, USM); • ibid., Dist. Rupa-Rupa, al oeste de Tingo María, 700–750 m, 21 March 1978, *J. Schunke V*. 10070 (US); • ibid., hills above airport, Tingo María, left bank of Río Huallaga, 700–800 m, 5 April 1976, *T.C. Plowman* 5819 (US, USM); • ibid., Calpar Bella; cueva de los Hauriños. 700–900 m, 2 July 1976, *J. Schunke V.* 9488 (USM); • ibid., *J. Schunke V.* 9489 (F, US); • ibid., Tingo María, 600–650 m, 10 July 1958, *R. Ferreyra* 13136 (US, USM). • **Junín**: Prov. Chanchamayo, de Chontabamba a Marinioc, July 1878, *A. Raimondi* 10637, 10648 (USM); • ibid., Dist. Chanchamayo, La Merced, 700 m, 29 May–4 June 1929, *E.P. Killip & A.C. Smith* 23477 (F, US); • ibid., 2000 ft, 10–24 Aug. 1923, *J.F. Macbride* 5266 (F, US); • ibid., Dist. San Ramón, Hac. Huacara, 800–900 m, 11 July 1959, *K. Lothar Diers* 1299 (US); ibid, Lourdes de Oxabamba, 11°04'S, 75°23'W; 1246 m, 3 August 2023, *R. Villanueva et al.* 916 (MOL); • ibid., Dist. Perene, Colonia Perene, 680 m, 14–22 June 1929, *E.P. Killip & A.C. Smith* 24914 (F, US). Prov. Huancayo, Oserato/Tambo, 4 Aug. 1964, *G. Weiss* 240 (F). Prov. Satipo, Dist. Río Tambo, Com. Nativa Oviri, 11°15'S, 73°47'W, 4 July 2018, *M. Kujawska* 429 (USM); • ibid., Dist. Satipo, San Francisco de Satipo, 700 m, 23 June 1977, *J. Solomon* 3225 (F); • ibid., Satipo, 800 m, Aug. 1940, *C. Ridoutt* s.n. (USM 11823 & USM11430); • ibid., July 1940, *C. Ridoutt* s.n. (USM 11875). • **Loreto**: without exact data, 480 m, 25 July 1964, *P.C. Hutchison et al.* 6045 (K). Prov. Alto Amazonas, Yurimaguas, Río Huallaga, May 1855, *R. Spruce* 3892 (K); • ibid., Santa Rosa, lower Río Huallaga below Yurimaguas, 135 m, Sept. 1929, *E.P. Killip & A.C. Smith* 28893 (F, US); • ibid., Washintsa and vicinity, Río Huasaga, 3°20'S, 76°20'W, 185 m, 16–26 July 1986, *W. Lewis et al.* 11245 (USM) • ibid., Puranchim, Río Sinchiyacu, 2°50'S, 76°55'W, 200 m, 21–27 Nov. 1986, *W. Lewis et al*. 11881 (USM). Prov. Coronel Portillo, Boqueron pass, Tingo María to Pucallpa, 480 m, 19 June 1982, *D.C. Wasshausen & O. Tovar* 1272 (K, US). Prov. Datém del Marañón, Dist. Manseriche, Soledad, on Río Itaya, 110 m, 20–22 Sept. 1929, *E.P. Killip & A.C. Smith* 29645 (F). Prov. Ucayali, Pucallpa–Lima Highway, km 85, 200 m, 20 July 1970, *S. McDaniel* 13940 (F, US). • **Madre de Dios**: Prov. Manu, Cocha Cashu Biological Station. Manu National Park, 11°52'S, 71°22'W, 400 m, 1 Aug. 1983, *Al. Gentry* 43281 (F, MO, US); • ibid., 400 m, 14 Sept. 1985, *P. Nuñez* 1862 (F); • ibid., 400 m, July 1984, *P. Nuñez* 12 (CUZ024596); • ibid., 350 m, July 1978, *R. Foster & J. Terborgh* 6551 (F); • ibid., 27 Nov. 1980, *R.B. Foster* 5955 (F); • ibid., 11°53'S, 71°23'W, 350 m, 5 Sept. 1986, *R.B. Foster* 11272 (F, USM); • ibid., Cocha Cashu, between Panagua and Tayakome, 11°22'S, 71°22'W, 400 m, 17–24 Aug. 1974, *R.B. Foster et al.* 3311 (K, U, US, USM); • ibid., Cocha Cashu uplands, 11°45'S, 71°0'W, 400 m, 28 July 1986, *P. Nuñez* 5517-1862 (BRIT, CUZ, USM); • ibid., Quebrada Fierro, 11°22'S, 71°58'W, 400 m, July 1988, *G. Shepard* 2199 (F); Camp. Botánico, 320 m, 5 Sept. 2003, *A. Maceda* 843 (BRIT). Prov. Tahuamanu, Quebrada del Km 24 de carretera Iberia–Iñapari, 1 June 1978, *F. Encarnación* 1168 (US). Prov. Tambopata, Dist. Puerto Maldonado, Com. Nativa de Infierno. Hermosa Chica. Centro Ñape, 12°50'S, 69°17'W, 260 m, 25 June 1991, *V. Baca* 181 (US, USM); • ibid., 13 Aug. 1990, M. Alexiades 1019 (NY, US, USM); • ibid., Las Piedras, 12°29'S, 69°03'W, 200 m, 28 July 1991, *M.E. Timaná* 1954 (US). • **Pasco**: Prov. Oxapampa, 8 km W of Puente Paucartambo, 1200 m, 27 May 1979, *D.C. Wasshausen & F. Encarnación* 1130 (K, US, USM); • ibid., Dist. Huancabamba, P.N. Yanachanga, El Huampal, 10°11'S, 75°34'W, 1200 m, 29 June 2003, *H. Van der Werff* 17870 (MO); • ibid., 10°10'58"S, 75°34'25"W, 1100 m, 23 July 2006, *A. Montenegro et al.* 12523 (HOXA); • ibid., 10°11'S, 75°34'W, 1200 m, 1 July 2003, *H. van der Werff et al.* 17923 (HOXA, US); • ibid., Camino a Pozuzo, 10°04'02"S, 75°32'59"W, 1200–1480 m, 2 June 2004, *R. Rojas et al*. 2558 (HOXA, USM); • ibid., Dist. Oxapampa, Carretera Oxapampa y Paucartambo, 10°55'51"S, 75°17'08"W, 730 m, 11 June 2003, *R. Rojas et al.* 1145 (HOXA); • ibid., Dist. Palcazú, Com. Nativa Loma Linda, 334 m, 19 July 2007, *E. Becerra* 1575 (AMAZ, HOXA, HUT, MO, MOL, USM); • ibid. Dist. Pozuzo, 2000 ft, 20–22 June 1923, *J.F. Macbride* 4643 (F, US); • ibid., Dist. Villa Rica, along road Chatarra–Cacazu, 10°32'S, 75°04'W, 890 m, 13 July 2003, *H. van der Werff et al.* 18433 (HOXA, US, USM). • **San Martin**: Entre Cinchono y Boquerón, 15 Aug. 1946, *R. Ferreyra* 1115 (US, USM). Prov. Lamas, Vicinity of Shanusi, 38 km SW of Yurimaguas, 250 m, 12 May 1979, *D.C. Wasshausen & F. Encarnación* 1048 (K, US); Prov. Moyobamba, 1835, *A. Mathews* 1535 (K). Prov. Tocache, Dist. Uchiza, Tingo María–Tocache Nuevo road, valley of Río Huallaga, 10°04'02"S, 75°32'59"W, 500 m, 6 April 1984, *T.B. Croat* 57968 (US, USM); • ibid., Dist. Tocache, Tocache Nuevo, 350–400 m, 22 June 1974, *J. Schunke V.* 6985 (F, US, USM); • ibid., Fundo “Cucareland”, (Río Cañuto), 500–520 m, 12 May 1979, *J. Schunke V.* 10962 (US); • ibid., Río Cañuto, cerca de Tananta, 500–520 m, 2 June 1980, *J. Schunke V.* 11763 (US, USM); • ibid., Quebrada de Huaquisha, 400–500 m, 3 Dec. 1980, *J. Schunke V.* 12436 (F, JRBJ, K, NA, U, US, USM). • **Ucayali**: Cerro de Canchyuaya, Río Ucayali, 135 m, 30 July 19300, *S. McDaniel* 2583 (US); • ibid., 30 July 1970, *S. McDaniel* 14146 (F, US, USM). Prov. Atalaya, Com. Nativa Yaminahua–Raya, saliendo de Atalaya. 10°19'51"S, 72°58'08"W, 250 m, 25 June 2000, *H. Beltrán & R. Retejo* 3528 (USM). Prov. Coronel Portillo, Cordillera Azul, km 64, Tingo María–Pucallpa road, 1 km E of Puente Cholon, 600 m, 4 June 1981, *K. Young & G. Sullivan* 690 (US); • ibid., Lower Boquerón del Padre Abad, 480 m, 25 July 1964, *P.C. Hutchison et al.* 6045 (F, K, P, US, USM); • ibid., near Perú–Brasil border, quebrada Sapallal, tributary of Quebrada Shesha, base of Cerro Las Cachoeiras, 08°02'S, 73°55'W, 260 m, 19 June 1987, *Al. Gentry & C. Diaz* 58458 (BRIT, USM); • ibid., Dist: Iparia. falda del Cerro Ariapo, cuencas de los Ríos Iparia y Ariapom, Reserva Comunal el Sira, 9°27'S, 74°33'W, 1550–1600 m, 20 Sept. 2010, *J.G. Graham* 5972 (US), 5993 (MOL, US). Prov. Padre Abad, Dist. Padre Abad, Cuenca del Río Aguaytia, Quebrada Chesman cerca del Boquerón del Padre Abad, margen izquierda del Río Yurac, 350–400 m, 7 Feb. 2004, *J. Schunke & J. Graham* 15824 (F, MOL, USM); Vecindad de Aguaytia, 1 July 1960, *M. Mathias & D. Taylor* 5073 (F, USM); Prov. Purús, Dist. Purús, al lado del Río Purús, cerca Comunidad Nativa de Miguel Grau, 220 m, 1 July 2002, *J.G. Graham* 1596 (US); • ibid., Río Curanja, cerca la comunidad nativa colombiana, 10°4'S, 71°6'W, 300–350 m, 3 July 2002, *J.G. Graham & J. Schunke Vigo* 1602 (US); ibid, Río Curanja, cerca Com. Nativa de Colombiana, 10°4'S, 71°6'W, 300–350 m, 14 July 1998, *J. Graham* 584 (F, US).

##### Lectotypification.

MA-817205 is somewhat arbitrarily selected as the lectotype of *Diantheraappendiculata* as it has the original annotation of *Diantheraappendiculata* and is marginally the better specimen.

#### ﻿﻿10. Justicia tumbesiana

Taxon classificationPlantaeLamialesAcanthaceae

R.Villanueva & J.R.I.Wood sp. nov.

3DABF4A8-B082-5F97-BE1F-9DD1BB9FC1C3

urn:lsid:ipni.org:names:77363410-1

##### Type.

Peru • Tumbes, Prov. Zarumilla, Dist. Matapalo, entre P.C. “El Caucho” y P.C. “Campoverde”. Bosque Nacional de Tumbes. Reserva de Biósfera del Noroeste, 3°50'29"S, 080°15'33"W, 720 m, 24 July 1992, *Camilo Díaz S., H. Horna & A. Peña Cruz* 5081 (holotype MO-04651117, isotypes MEXU, MOL, US, USM – 3 sheets).

##### Diagnosis.

Bears an obvious superficial resemblance to *Justiciaappendiculata* in the large, dark, sometimes reddish inflorescence bracts and the tubular red, pubescent corolla but leaves broadly oblong-elliptic (not narrowly oblong-elliptic), the inflorescence shorter, to 10 cm long (not up to 15 cm), the lateral branches subsessile (not clearly pedunculate), the floral bracts prominent, oblanceolate, up to 10 mm long (not inconspicuous, ovate c. 2–3 mm long).

##### Description.

Shrub 3.5 m high; stem woody, bark pale brown, peeling, glabrous. Leaves subequal in each pair, petiolate, lamina 8–18 × 3.5–8 cm, broadly oblong-elliptic, apex very shortly acuminate, base cuneate and shortly decurrent, margin crenulate, both surfaces glabrous with abundant small cystoliths, abaxially paler, slightly glaucous, the venation highlighted-white, lateral veins 8 pairs; petioles 0.7–5 cm. Inflorescence of short axillary spikes up to 10 cm long and 3 cm wide (excluding expanded corolla), peduncles c. 1.5–2.5 cm, scurfy; rhachis bifariously scurfy; inflorescence bracts 4–5 × 1–2 cm, narrowly obovate, dark coloured, glabrous; flowers arising in short opposite spikes of indeterminate form borne on a puberulent lateral branch up to 1 cm long, superficially appearing verticillate; floral bracts 1 × 2–4 mm, oblong-oblanceolate, puberulent, often purplish; bracteoles 7 × 1 mm, oblong, puberulent; pedicels c. 1 mm, puberulent; calyx 5-lobed, lobes 5 × 1 mm, lanceolate, acuminate, puberulent; corolla 3 cm long, orange, pubescent with gland-tipped hairs, subcylindrical, tube gradually widened from 1 mm at base to 5 mm after c. 18 mm, upper lip 12 mm long, entire, lower lip c. 12–13 mm long, 3-lobed, lobes oblong, 4 × 2 mm, obtuse; filaments c. 22 mm long, pilose below, anther thecae oblong, c. 2.5 × 0.5 mm, glabrous, lower with a basal appendage, parallel, slightly superposed; pollen prolate, 40–55 × 25 μm, 2-aperturate, colporate, 1 row of 6–7 insulae on either side of aperture with a second row sometimes grading into peninsulae (Fig. 49D); style thinly pilose below, glabrous above; ovary narrowly ovoid, c. 2.5 mm high, black, glabrous. Capsule and seeds not seen.

##### Illustration.

Fig. 13.

**Figure 13.**
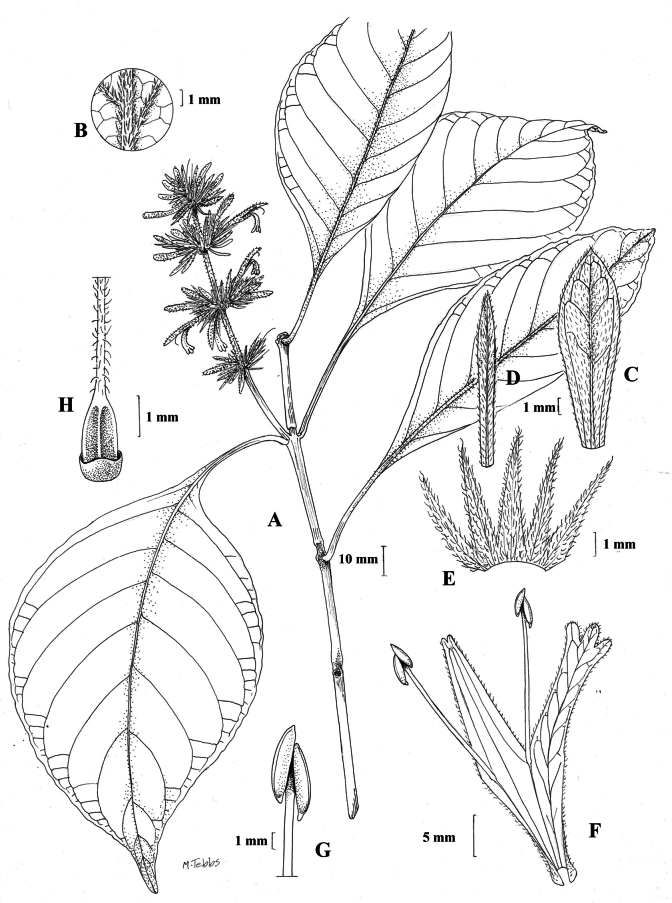
*Justiciatumbesiana***A** habit **B** detail of abaxial surface of leaf **C** bract **D** bracteole **E** calyx **F** corolla opened out to show stamens **G** anther **H** ovary and style base. Drawn from *Diaz et al.* 5081 by Margaret Tebbs.

##### Etymology.

This species is named *Justiciatumbesiana* after Tumbes region, where it is the only recorded species in the genus. The Bosque Nacional de Tumbes in the Reserva de Biósfera del Noroeste is an isolated area of woodland near the Pacific Ocean in Peru’s otherwise arid coastal region.

##### Phenology.

Found in flower in July.

##### Habitat.

Woodland at 720 m.

##### Distribution.

Endemic to Tumbes in Peru and only known from the type collection. Fig. 56.

##### Material examined.

**Peru** • **Tumbes**: Only known from the type collection.

#### ﻿﻿11. Justicia pelianthia

Taxon classificationPlantaeLamialesAcanthaceae

Leonard, Contr. U.S. Natl. Herb. 31: 591. 1958. (Leonard 1958: 591)

0B537C50-19B6-5DBE-90A1-3D7AAE498F22

##### Type.

Colombia • Putumayo, between Quebrada de la Hormiga and San Antonio de Güamués, 330 m, *J. Cuatrecasas* 11157 (holotype US-00137141, isotypes COL-00004518, F-0047446F).

##### Description.

Subshrub, stems bifariously strigose. Leaves petiolate, lamina 8–21 × 4–9 cm, oblong-elliptic, shortly acuminate, base attenuate and decurrent onto a petiole up to 3.5 cm long, both surfaces glabrous except for the strigose veins; petioles 1–6 cm, strigose. Inflorescence a compact terminal panicle up to 16 cm long and wide, over-topped by the leaves, composed of short lax spikes, these strigose with golden hairs; bracts 7–10 × 1.25 mm, lanceolate; bracteoles similar; calyx 12–15 mm long, deeply 5-lobed, puberulent; corolla up to 6.5 cm long, red to purple, glandular-pubescent, upper lip 3.3 cm long, emarginate, lower lip 3.5 cm long, shallowly 3-lobed, lobes 3–4 mm long; anther thecae broadly oblong, 2 × 1.25 mm, strongly superposed, lower with minute basal appendage; pollen perprolate, 69–77 × 33 μm, 2-aperturate, colporate, 1 row of c. 9–12 insulae and a second ill-defined row of peninsulae on either side of aperture (Fig. 49C); ovary glabrous. Capsule and seeds not seen.

##### Illustration.

Fig. 14A–D.

**Figure 14.**
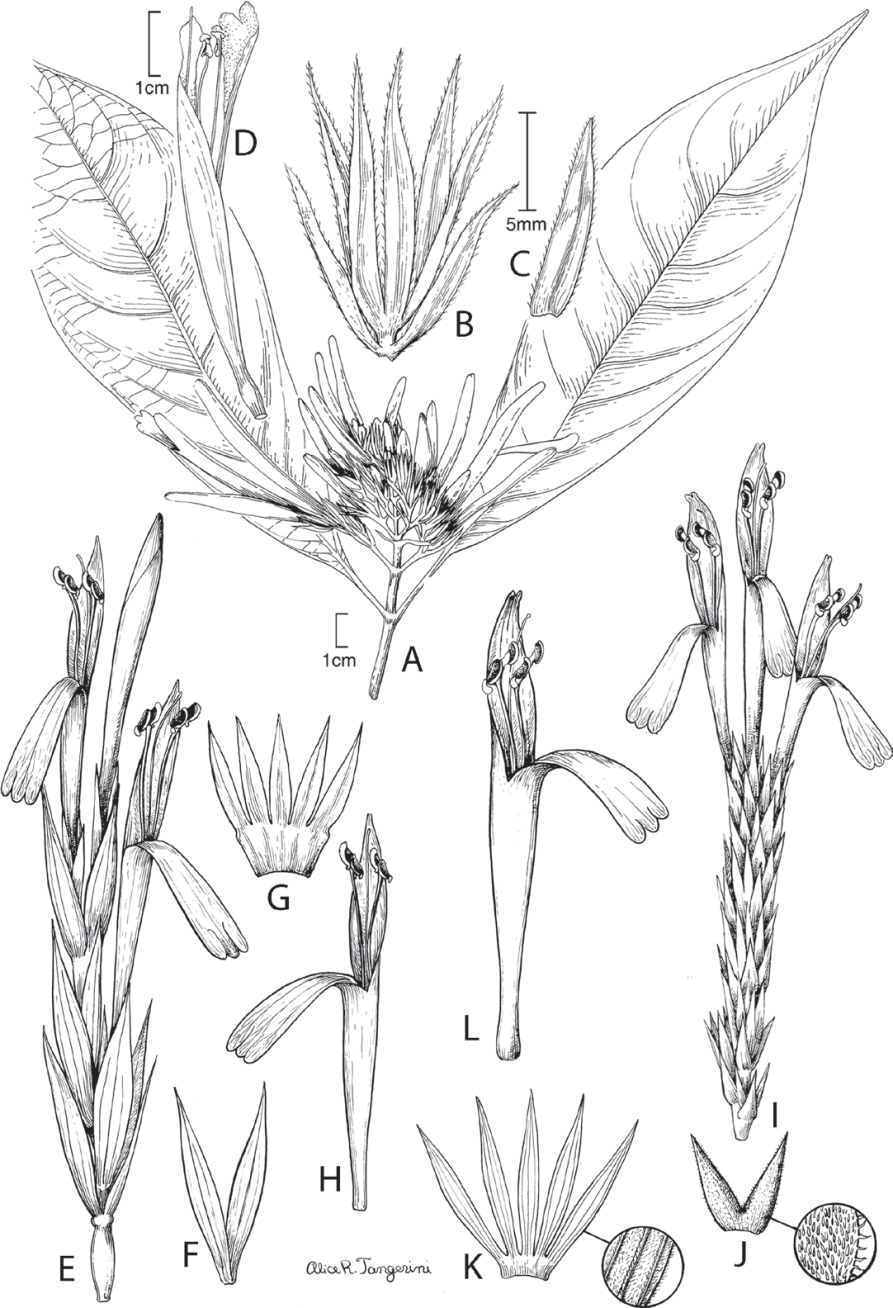
*Justiciapelianthia***A** habit **B** bracts, bracteoles and calyx **C** bract **D** corolla. *Justiciasanchezioides***E** inflorescence **F** bracts with detail of cystoliths on surface and indumentum **G** bract with detail of venation **H** corolla and stamens. *Justiciasiraensis***I** inflorescence **J** bracts **K** calyx **L** corolla. **A, D** drawn from *Klug* 1429 by Cathy Pasquale **E, H** drawn from *Schunke* 4809 by Alice Tangerini **I, L** drawn from *Wasshausen & F. Encarnación* 529 by Alice Tangerini.

##### Habitat.

Lowland, primary rainforest on clay/lateritic soils, 100–150 m.

##### Phenology.

Mainly June to August but this may reflect seasons with accessibility to collectors.

##### Distribution.

Loreto region of Amazonian Peru and neighbouring Putumayo in Colombia. A new record for Peru. Fig. 57.

##### Material examined.

**Peru** • **Loreto** (all from the Iquitos region) : Timbuchi, Río Nanay, June–July 1929, *Ll. Williams* 861a (F). Prov. Maynas, Quebrada Sucusari, Camp. Llachapa, N side of Río Napo below Mazán, 140 m, 6 Nov. 1979, *Al. Gentry et al.* 27592 (MO, US); • ibid., Dist. Iquitos, Mishuyacu, near Iquitos, 100 m, Oct.–Nov. 1929, *G. Klug* 73 (F, US); • ibid., May–June 1930, *G. Klug* 1429 (F, US); • ibid., Iquitos, 100 m, 3–11 Aug. 1929, *E.P. Killip & A.C. Smith* 27116 (US); • ibid., Iquitos, trail from Picura (lower Río Nanay) to Río Mazán, 19 May 1978, *S. McDaniel et al.* 21499 (US); • ibid., Río Amazonas, c. 10 km below mouth of Río Nanay, Trocha de Santa María de Ojeal, 14 June 1976, *S. McDaniel et al.* 20709 (MO, US); • ibid., Río Amazonas, ca 10 km below mouth of Río Nanay, trail from Santa María de Ojeal to Interior, 21 June 1976, *M. Rimachi* 2351 (US); • ibid., Dist. Alto Nanay, Pinto-Cocha on the Río Nanay, July 1929, *Llewellyn Williams* 799 (F).

##### Notes.

*Justiciapelianthia* and *J.palaciosii* Wassh. differ principally in the dimensions of the bracts and calyx but may prove to be conspecific. The older name, *J.pelianthia* is adopted here. *Justiciapelianthia* is also similar in facies to *J.sanchezioides* but the bracts are inconspicuous, only 5–10 mm long and the calyx shorter. Curiously there have been no records from Peru since 1979, suggesting either that there has been forest clearance where the plant grew or that there has been no recent field work in the area.

#### ﻿﻿12. Justicia sanchezioides

Taxon classificationPlantaeLamialesAcanthaceae

Leonard, Contr. U.S. Natl. Herb. 31: 572. 1958. (Leonard 1958: 572)

2E1A20B2-091F-5C1C-B662-93C5F21294AF

##### Type.

Colombia • Dept. Putumayo, Puerto Ospina, *J. Cuatrecasas* 10581 (holotype US-00137162, isotype COL-00004522, F-0047448F).

##### Description.

Subshrub up to 3 m high; stems glabrous or nearly so. Leaves large, lamina 9–28 × 2–8 cm, oblong-elliptic, acuminate to an obtuse apex, base attenuate and decurrent on the petiole, glabrous, lateral veins prominent, 10–15 pairs; petioles 0.5–3 cm. Inflorescence a compact terminal panicle of 1-several branched, hirsute spikes up to 12 cm long, hairs often golden; peduncles up to 2 cm; bracts 18–21 × 3–4 mm, oblong or lanceolate, thinly puberulent, bracteoles similar but only 1–2 mm wide; calyx 5-lobed, lobes 11–15 × 2 mm, lanceolate, acute, thinly pubescent; corolla 4.5–5.5 cm long, reddish, glandular-puberulent, tube 2–3 cm long, upper lip emarginate, lower lip shallowly 3-lobed, the lobes 4 × 3 mm; thecae superposed, oblong 2.5 × 1.25 mm, with a distinct basal appendage; pollen prolate, 58–68 × 34–35 μm, 2-aperturate, colporate, 2 rows of c. 7–9 insulae on either side of aperture but second row grading into peninsulae ovary glabrous (Fig. 49F, Kiel et al. 2018: 462). Capsule 20 × 4 mm, clavate, 4-seeded, minutely glandular; seeds rugose, lenticular.

##### Illustration.

Fig. 14E–H.

##### Habitat.

Lowland rainforest, often in gullies and by streams up to about 800 m.

##### Phenology.

Found in Flower from April to August.

##### Distribution.

Colombia, Ecuador and Peru, somewhat scattered and disjunct in several parts of its distribution. Fig. 57.

##### Material examined.

**Peru** • **Loreto**: Prov. Maynas, southern side of Río Putumayo, near Puerto Leguizamo 0°11'38”–0°12'09"S, 74°47'16”–74°49'34"W, *Suarez et al.* 1314 (COAH). • **San Martin**: Prov. Tocache [Mariscal Cáceres], Tocache Nuevo–Juanjuí road, 89 km de Tocache Nuevo, 7°43'S, 76°40'W, 810 m, 23 July 1982, *D.N. Smith* 2148 (US); • ibid., Dist. Pólvora, Puerto Pizana (margen derecha del Río Huallaga) [8°01'S, 76°39'W], 10 April 1971, *J. Schunke Vigo* 4809 (COL, F, K, MO, USM); • ibid., Puerto Pizana, Río Huallaga [8°01'S, 76°39'W], 350 m, 4 June 1974, *J. Schunke Vigo* 6909 (F, MO, US, USM); • ibid., Chauyauyacu, cerca de Canuto, 10 Aug. 1979, 600 m, *R. Ferreyra* 19291 (USM); • ibid., Dist. Tocache [Nuevo], carretera al Río Tocache, 400 m, 2 Aug. 1969, *J. Schunke Vigo* 3278 (F, K, US, USM); • ibid., 14 April 1970, *J. Schunke Vigo* 3901 (F, K, US, USM); • ibid., Cerro de Palo Blanco, 15 km from Tocache Nuevo, 700–800 m, 15 July 1982, *A. Meerow et al.* 1009 (FLAS); • ibid., Quebrada Cachuyacu de Huaquisha, margen derecha del Río Huallaga, 17 May 1970, *J. Schunke Vigo* 3989 (F, K, US, USM); • ibid., camino a Santa Rosa, margen derecha del Río Mishollo, 350–370 m, 5 Aug. 1973, *J. Schunke Vigo* 6734 (F, MO, US, USM); • ibid., Quebrada de Yacu Sisa (camino a Shunté), 800–850 m, 18 July 1974, *J. Schunke Vigo* 7557 (US, USM); • ibid., Almendras camino a Pueblo Viejo, 400 m, 21 April 1975, *J. Schunke Vigo* 8194 (US); • ibid., Cerro de Palo Blanco, 450–600 m, 14 June 1978, *J. Schunke Vigo* 10236 (MO); • ibid., Palo Blanco, above Río Tocache, 500–550 m, 29 June 1978, *T. Plowman & J. Schunke Vigo* 7457 (COL, F, K, U, US, USM); • ibid., Trocha a Cañutillo, cerca Cerro Palo Blanco, 800–850 m, 6 May 1980, *J. Schunke Vigo* 11559 (MO, US); • ibid., Cachuyacu de Huaquisha, 500–600 m, 9 Dec. 1980, *J. Schunke Vigo* 12458 (BR, F, K, L, MO, NA, US, USM).

##### Note.

Leonard (1958) failed to note the similarity between *Justiciasanchezioides* and *J.pelianthia*. The former resembles a more robust bracteate version of the latter with larger flower parts, particularly the longer calyx.

The following plants from Huánuco may represent a distinct species. They differ by their large bracts 31–40 (–60) × 7–10 (–12) mm and anthers with a very short basal appendage.

##### Additional material examined.

**Peru** • **Huánuco**: Leoncio Prado, Dist. Hermilio Valdizan [9°56'59"S, 76°15'04"W], cerca de la Divisoria, 1500–1600 m, 21 June 1976, *J. Schunke Vigo* 9331 (F, MO, US); • ibid., La Divisoria, 9°56'59"S, 76°15'04"W, 1550 m, 17 April 1976, *T. Plowman* 5919 (US).

#### ﻿﻿13. Justicia aphelandroides

Taxon classificationPlantaeLamialesAcanthaceae

(Mildbr.) Wassh., Monogr. Syst. Bot. Missouri Bot. Gard 45: 1253. 1993. (Wasshausen 1993: 1253)

1E2EECFE-5D2B-5E90-9D6A-E6CE32090839

Jacobinia aphelandroides Mildbr., Notizbl. Bot. Gart. Berlin-Dahlem 9: 989. 1926. (Mildbraed 1926: 989) Type. PERU. Upper Marañón, *Tessmann* 4222 (holotype B†, photo of holotype F0BN008903, isotype MO-3345889, not seen).

##### Type.

Based on *Jacobiniaaphelandroides* Mildbr.

##### Description.

Herb 2 m high, stems stout, glabrous. Leaves petiolate, lamina 20–25 × 5–7 cm, narrowly obovate, apex shortly acuminate, gradually tapered to a cuneate base, margin entire; petioles 1–1.5 cm. Inflorescence of simple terminal spikes c. 7 cm long; peduncles 5 cm; bracts 7–8 × 4 mm (c. twice as long as broad), ovate, finely acuminate; bracteoles similar but smaller, 6 × 2.5 mm; calyx subequally 5-lobed to 2 mm above base, lobes 8.5–9.5 × 1.5 mm, minutely puberulent; corolla pink, papillate, tube 3.5–4 cm long, lips subequal, 2.2–2.5 cm long, upper lip lanceolate, emarginate, lower lip 3-lobed, lobes c. 2 mm long, lanceolate; pollen prolate-perprolate, 63–74 × 29–39 μm, 2-aperturate, colporate, ≈ 3 rows of solid insulae on either side of aperture (Fig. 49E). Capsule and seeds not seen.

##### Phenology.

Found in flower October.

##### Habitat.

Lowland, rainforests, 160–275 m.

##### Distribution.

Endemic to Amazonas region. Fig. 57.

##### Material examined.

**Peru** • **Amazonas**: Prov. Bagua, Río Marañón, mouth of Río Santiago, 250–275 m, 14–15 Oct. 1962, *J.J. Wurdack* 2245 (F, K, USM).

##### Notes.

Similar to *Justiciaelegantissima* (Lindau) Wassh. but the calyx lobes subequal and much shorter.

Only known from two collections, the most recent in 1962. Further exploration in the Río Santiago zone is needed to confirm the continued presence of this species as the surroundings of the Santiago river have been degraded by illegal mining.

#### ﻿﻿14. Justicia rauhii

Taxon classificationPlantaeLamialesAcanthaceae

Wassh. Beitr. Biol. Pflanzen 63: 428. 1988. (Wasshausen 1988: 428)

E0B50ED9-D6EE-5BD0-AA65-2A8B6B13250B

##### Type.

Peru • Cusco, [Prov.] Paucartambo, Dist. Kosñipata, along roadside 3 km E of Atalaya, 9 km N of Pilcopa, *D.C. Wasshausen & F. Encarnación* 579 (holotype US-00074193).

##### Description.

Subshrub 1 m high. Leaves petiolate, lamina 11–23 × 4–8 cm, oblong-elliptic, tapered and distinctly acuminate at both ends; petiole 2.5–5 cm. Inflorescence of 1–3 dense, reddish, terminal spikes 4.5–7.5 cm long; rhachis pubescent with glandular hairs; bracts 7–10 × 3 mm, indurate, oblong, acute, c. 3 times as long as broad; bracteoles similar but narrower; calyx 5-lobed, the lobes 9–10 × 1–1.2 mm, glandular-pubescent; corolla 3.5–4.5 cm long, yellow, papillate, tube c. 20 mm long, ± equalling lips, lower lip shallowly 3-lobed, the lobes ovate, c. 2 mm long; anther thecae oblong, 3 × 0.75 mm, nearly parallel, strongly superposed, lower with white basal appendage; pollen prolate, 47 × 36 μm, 3-aperturate, colporate with 1 row and a partially developed second row of insulae on either side of the aperture (Wasshausen 1988: 427). Capsule and seeds not seen.

##### Illustration.

Fig. 12; Wasshausen 1988: 422, I–K.

##### Phenology.

Although found in flower in February and October, flowering is principally in the June to August period.

##### Habitat.

Lowland rainforest, 400–760 m.

##### Distribution.

Endemic to southern Peru in Cusco and Madre de Dios. Fig. 58.

##### Material examined.

**Peru** • **Cusco**: Prov. Paucartambo, Dist. Kosñipata, lumber Trail north of Pilcopata, 580 m, 27 June 1975, *D.C. Wasshausen & F. Encarnación* 586 (K, US); • ibid., Kosñipata [Pilcopata], Fundo Santa Alicia, 13°05'S, 71°10'W, 700 m, 3 Feb. 1985, *A. Tupayachi* 2 (MO, US), • ibid., Pilcopata-Atalaya, 450–550 m, 5 Aug. 1956, *C. Vargas* 11291 (CUZ, US); • ibid., 760 m, 4 June 1964, *C. Vargas* 15516 (US); • ibid., along roadside 3 km E of Atalaya, 9 km N of Pilcopata, 600 m, 26 June 1975, *D.C. Wasshausen & F. Encarnación* 608 (US); • ibid., along lumber Trail N of Pilcopata, 580 m, 27 June 1975, *D.C. Wasshausen & F. Encarnación* 609 (US). • **Madre de Dios**: Prov. Manu, P.N. Manu, Quebrada Fierro, first significant tributary of Río Manu upriver from Tayakome, 11°22'S, 71°58'W, 400 m, July 1988, *G. Shepard* 2043 (F); • ibid., Río Sotileja, 11°40'S, 71°55'W, 400 m, 8 Oct. 1986, *R. Foster & B. d´Achille* 11692 (F); • ibid., Dist. Fitzacarrald, Com. Nat. Yomibato, across river from Oscar´s new garden, 13 July 1996, *G. Shepard* 995 (USM).

#### ﻿﻿15. Justicia siraensis

Taxon classificationPlantaeLamialesAcanthaceae

Wassh., Ann. Naturhist. Mus. Wien, B 108B: 171. 2006[May 2007]. (Wasshausen 2007: 171)

B4BE24EE-231F-5B37-9791-20CF202777D1

##### Type.

Peru • Huánuco, Prov. Pachitea, Pucallapa region, Sira Mountains, 9°29'S, 74°50'W, 300–360 m, 15 July 1988, *B. Wallnöfer* 14-15788 (holotype W (not seen), isotype US-00902118).

##### Description.

Subshrub 2–2.5 m high; stems glabrous below, bifariously scurfy-puberulent above. Leaves shortly petiolate, lamina 10–20 × 3.5–7 cm, broadly oblong-elliptic to narrowly ovate, apex acute to shortly acuminate, base attenuate, ± glabrous; petioles 0.5–1 cm. Inflorescence of 1–3 dense, terminal spikes, 3–10 cm long, these simple or forked; peduncles 1–2 cm, rhachis puberulent; bracts 6.5–9 × 2 mm, often indurate, narrowly linear-lanceolate, ciliolate; bracteoles 6 × 1–1.5 mm; calyx subequally 5-lobed, lobes 9–10 × 1 mm, narrowly lanceolate, ciliolate; corolla 4.5–5.5 cm long, red, papillate, tube 2.5–3 cm, upper lip 1.8–2 cm, minutely notched, lower lip 3-lobed, lobes c. 2 mm long, rounded, cuculate; anther thecae oblong, 1.2 mm long, parallel, weakly superposed, both with a basal appendage; pollen prolate, 50 × 29 μm, 2-aperturate, colporate, 1 row of c. 6–7 insulae on either side of aperture. Capsule 14–15 × 2.9–3.6 mm, clavate, 4-seeded.

##### Illustration.

**Figure 15.**
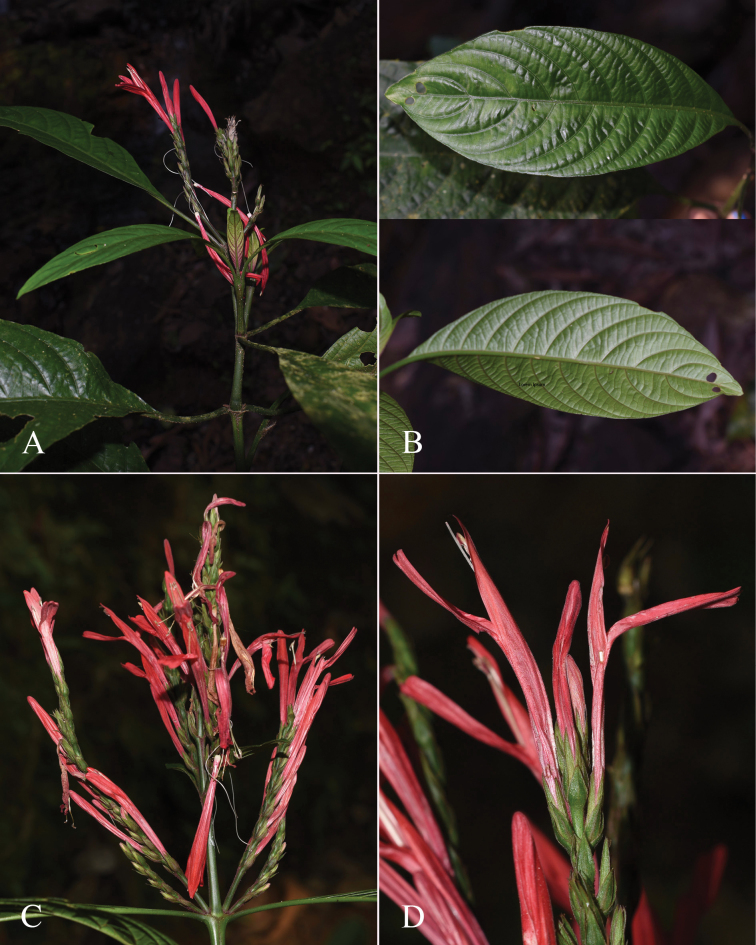
Photographs of *Justiciasiraensis* by Rosa Villanueva.

Figs 14I–L, 15; Wasshausen 2007: 172.

##### Phenology.

Found in flower in January and February and from June to August.

##### Habitat.

Rainforest in the Andean foothills 300–1500 m.

##### Distribution.

Endemic to Peru, occurring in scattered locations from Cusco north to San Martin. Fig. 58.

##### Material examined.

**Peru** • **Ayacucho**: Prov. La Mar, Trail between Santa Rosa and Sanabamba, 700 m, 9 June 1975, *D.C. Wasshausen & F. Encarnación* 529 (US). **Cusco**: Prov. La Convención, Dist. Echarate, Río Manguriari, Alto Urubamba, 12°47'S, 72°40'W, 750 m, 2 Feb. 1991, *P. Nuñez et al.* 12947 (MO); • ibid., Alto Manguriari, 700 m, 4 Aug. 1990, *G. Ortiz* 4 (CUZ027839). • **Huánuco**: Prov. Puerto Inca [Pachitea], Dist. Honoria, Miel de Abejas, Río Pachitea, 300–400 m, 19 July 1967, *J. Schunke Vigo* 2123 (F, HOXA, K, US, USM). • **Junín**: Prov. Satipo, Dist. Río Tambo, sector Shimabenzo, Feb. 2012, *J.L. Marcelo Pena* 6350 (MOL); • ibid., Com. Nat. Pichiquia, Parque Nacional Otishi, 11°22'12"S, 74°22'19"W, 1348 m, 13 July 2013, *L. Valenzuela et al.* 25086 (HOXA, USM). • **Loreto**: Prov. Ucayali, Dist. Pampa Hermosa, Parque Nacional Cordillera Azul. Sector Shanshuico, 7°20'54"S, 76°00'33"W, 421 m, *R. Vásquez et al.* 41728 (USM, HOXA). • **Pasco**: Prov. Oxapampa, Dist. Pozuzo, Alto Lagarto a Pozuzo Alto Victoria, 10°07'09"S, 75°29'25"W, 1500 m, 29 June 2008, *R. Rojas & G. Ortiz* 5846 (HOXA, MO, USM); • ibid., Dist. Palcazú, Com. Nat. Alto Lagarto-Convento (Reserva Comunal Yanesha), 10°08'04"S, 75°22'06"W, 500 m, 30 July 2014, *R. Rojas & G. Ortiz* 9355 (HOXA, HUT). • **San Martin**: Prov. Mariscal Cáceres, Dist. Campanilla, Cachihuañusca, 21 Aug. 1970, *J. Schunke Vigo* 4285 (F, US); • ibid. Dist. Juan Jui, Alto Río Huallaga, 400–800 m, Jan. 1936, *G. Klug* 4202 (BM, F, K. U, US, USM).

##### Note.

Differs somewhat unsatisfactorily from *Justiciarauhii* principally by the red corolla 4.5–5.5 cm long.

#### ﻿﻿16. Justicia beckii

Taxon classificationPlantaeLamialesAcanthaceae

Wassh & J.R.I.Wood, Kew Bull. 58(4): 820. 2003. (Wasshausen and Wood 2003: 820)

CF949DF0-1A79-52F0-BBE1-EBCDAFC06F43

##### Type.

**Bolivia** • Caranavi, *D.C. Wasshausen & J.R.I. Wood* 2152 (lectotype US-00811134, designated here, isolectotypes K, LPB, US-00731151).

##### Description.

Shrub 1.5–2 m high; stems erect, glabrescent. Leaves with lamina 6.5–28 × 2.5–11 cm, oblong-elliptic to obovate, attenuate at both ends, entire, glabrous except for the minutely scurfy-puberulent veins beneath. Inflorescence a terminal panicle formed of shortly pedunculate spikes arising from the axils of the uppermost leaves; spikes 3–18 cm long; flowers imbricate; bracts 5–6 × 4–6 mm, elliptic, obtuse or shortly acute, rufous-scurfy-pubescent and coarsely ciliate; calyx 5-lobed to just above the base, lobes 7–9 mm × 0.75–1 mm, one slightly larger than the others, linear-lanceolate, scurfy-pubescent; corolla 2.6–4 cm long, salmon-red, covered in numerous sessile glands, the lips 8–12 mm long, the upper lip entire, slightly hooded, lower lip 3-lobed, the lobes 1.5 × 1 mm, oblong-ovate, rounded; anther thecae 1.75 × 0.5 mm, oblong, glabrous with a white basal appendage c. 0.5 mm long, superposed, parallel; pollen prolate, 2-aperturate, colporate, 2–3 rows of insulae on either side of the aperture, sexine reticulate (Wasshausen and Wood 2003: 811).

##### Illustration.

Wasshausen and Wood 2003: 822.

##### Distribution.

Andes of northern Bolivia and extreme south of Peru. Fig. 58.

##### Material examined.

**Peru** • **Puno**: Prov. Sandia, along Río Tambopata between San Juan del Oro and San Ignacio, 1100 m, 8 June 1982, *D.C. Wasshausen & A. Salas* 1228 (US).

##### Lectotypification.

There are two sheets of *Wasshausen & Wood* 2152 at US annotated as holotype, so to avoid uncertainty we are designating US-00811134 as lectotype as this was the sheet used in the preparation of the original description.

##### Note.

Closely related to *J.aphelandroides*, *J.siraensis* and *J.rauhii*. All four species share a very similar inflorescence with a long tubular corolla covered in very small, subsessile glands. Curiously, the fruit are not known from any of them except *J.siraensisJusticiabeckii* has a dark red corolla, which is shorter than in the other species, being only 2.6 to 4 cm in length. Additionally, the bracts are distinctive in the four species. *J.beckii* has elliptic bracts, c. 6 mm long, which are scarcely longer than broad and with an obtuse or shortly acute apex. In *J.rauhii* and *J.siraensis* the bracts are oblong or lanceolate, acute, up to 10 mm long and at least three times as long as broad. In *J.aphelandroides* the bracts are ovate, about twice as long as broad and terminate in a long fine point. *J.rauhii* has yellow flowers.

Species 17-19. The Chaetothylax clade. Species in this clade are characterised by 2-aperturate pollen, 4-lobed calyx and very strongly superposed anther thecae. The corolla tube is relatively slender, cylindrical and clearly longer than the corolla lips.

#### 17. Justicia radicans

Taxon classificationPlantaeLamialesAcanthaceae

Vahl, Enum. Pl. 1: 137. 1804. (Vahl 1804: 137)

02405F58-E945-552F-9FE7-8C5D342EF5B1

Dianthera ciliata Ruiz & Pav., Fl. Peruv. Prodr.1: 12. 1798. (Ruiz and Pavón 1798: 12) non Justiciaciliata Jacq. (1772). Type. PERU. *Ruiz & Pavon*, s.n. (lectotype MA-815493, designated here, isolectotypes BM-000992617, OXF-00194390).
Beloperone soukupii Standl. & F.A. Barkley, Madroño 9: 152. 1950 (Standley and Barkley 1950: 152). Type. PERU. Huánuco, 10 km downstream from Tingo María, 630 m, 28 Oct. 1938, *H.E. Stork & O.B. Horton* 9532 (holotype F-0040523F, isotypes G-00236204, K-000529475, NA-0026193, UC-647116).
Justicia soukupii (Standl. & F.A.Barkley) V.A.W. Graham, Kew Bull. 43(4): 604. 1988. (Graham 1988: 604), syn. nov.

##### Type.

Based on *Diantheraciliata* Ruiz & Pav.

##### Description.

Perennial herb usually 0.5–1 m high; stems thinly pubescent. Leaves petiolate, lamina 5–12 (–16) × 2–3.5 (–6) cm, oblong-elliptic, apex strongly mucronate, base narrowly cuneate, decurrent, glabrous, petioles mostly 1–1.5 cm. Inflorescence of short axillary and terminal foliose spikes, mostly c. 2 cm long but the terminal sometimes reaching 5 cm; peduncles 0–2 cm; floral bracts oblong to oblong-elliptic, conspicuously apiculate, 12–15 × 1.5–2.5 mm; bracteoles subulate c. 5 mm long; calyx subequally 4-lobed to base, the linear subulate, ciliolate, c. 10 mm long; corolla 1.8–2.5 mm long, white, lilac or pink, pubescent, lower lip deflexed, “herring bone” patterning present, shallowly 3-lobed, the lobes ovate, 1–2 mm long; thecae strongly superposed, the upper oblong-elliptic c. 1 × 0.5 mm, the lower separated by 1–1.5 mm, poorly developed; pollen perprolate, 37–40 × 23–25 μm, 2-aperturate, colporate, reticulum with large circular elements forming 1 row of 7–8 insulae on each side of aperture (Fig. 50A). Capsule 7–8 mm, clavate, pubescent, 4-seeded; seeds rugose.

##### Illustration.

Figs 16, 17.

**Figure 16.**
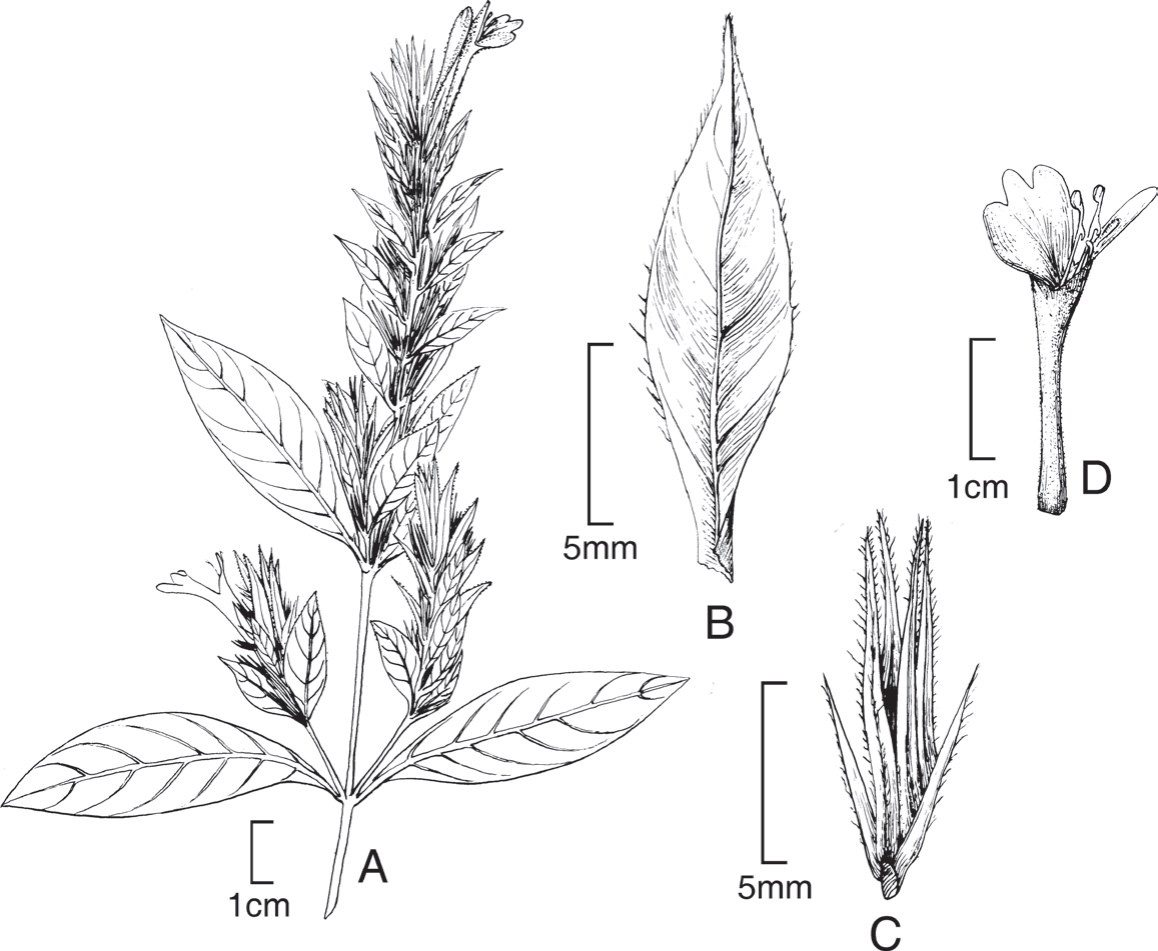
*Justiciaradicans***A** habit **B** bract **C** calyx and bracteoles **D** corolla with stamens and separated thecae. Drawn from *Wasshausen* 476 by Cathy Pasquale.

**Figure 17.**
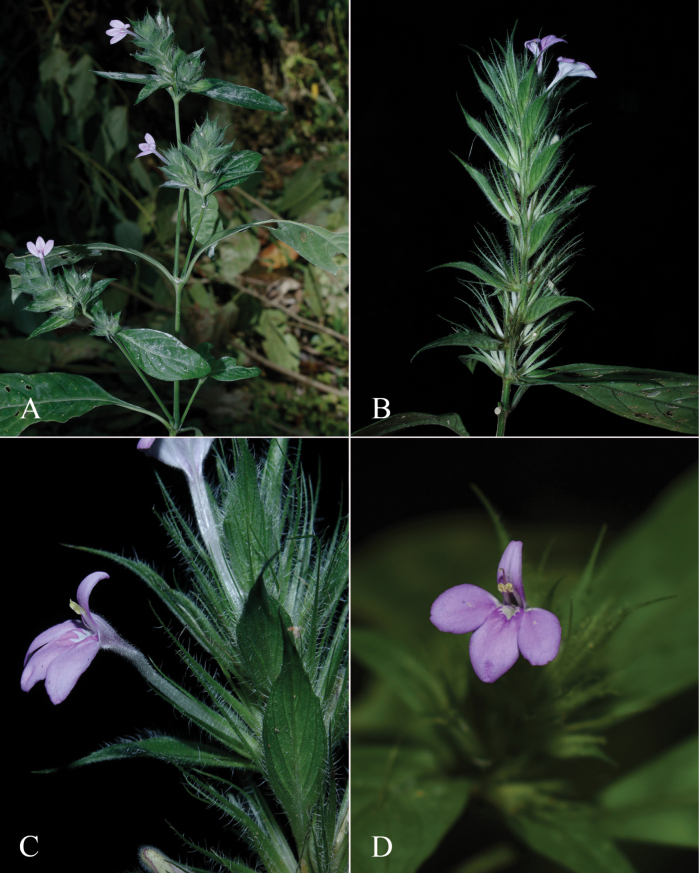
Photographs of *Justiciaradicans* (*Villanueva* 928) by Rosa Villanueva.

##### Phenology.

Found in flower through much of the year but principally between May and September.

##### Habitat.

Open, often disturbed Andean woodland, especially forest margins, from about 300 to around 1800 m.

##### Distribution.

Endemic to Peru and frequent on the eastern Andean slopes from San Martin south to Cusco. Fig. 59.

##### Material examined.

**Peru** • **Ayacucho**: Prov. La Mar, Río Marantari, below Santa Rosa Bridge, 580 m, 28 May 1975, D.C. *Wasshausen & F. Encarnación* 476 (K, MO, US, USM). **Cusco**: Prov. La Convención, entre Potrero and Idma [Itma], 1050–1500 m, 19 April 1953, *C. Vargas* 10596 (CUZ); • ibid., Río Mapituriani, opposite Hac. Luisiana, Cuenca Río Apurimac, 1000 m, 14 Sept. 1976, *D.C. Wasshausen & F. Encarnación* 647 (K, MO, US, USM); • ibid., Dist. Santa Ana, subcuenca Chuyapi, Poromate, 12°55'43"S 72°47'30"W, 1800–2300 m, 17 Sept. 2002, *L. Valenzuela et al.* 461 (CUZ, HOXA, HUT, MO). • **Huánuco**: Fundo Naranjillo, cerca de Tingo Maria, 700–750 m, 6 Aug. 1947, *R. Ferreyra* 2203 (US, USM). • **Junín**: Prov. Chanchamayo [Tarma], Dist. San Ramón, 3000 ft, Aug. 1945, *C. Sandeman* 4964 (K, OXF); • ibid., saliendo de Lourdes de Oxabamba, 11°03'47"S, 75°24'22.1"W, 1096 m, 3 Aug. 2023, *R. Villanueva et al.* 928 (HOXA); • ibid., Satipo–La Merced, 6 km W of Pichanaki, 69 km E of La Merced, 850 m, 26 May 1979, *D.C. Wasshausen & F. Encarnación* 1122 (K, MO, US); • ibid., 6 km S of Vitoc, along road from San Ramón to Pucara, 1100 m, 28 May 1979, *D.C. Wasshausen & F. Encarnación* 1139 (K, MO, US, USM); • ibid., Satipo–La Merced, 4.3 km W of Río Pichinaki, 10°58'S, 74°58'W, 8 June 1998, *T. Croat & M. Sizemore* 81965 (MO); • ibid., Santuario Nacional de Pampa Hermosa, 10°59'S, 75°25'W, 1400–1900 m, 14 March 2017, *S. Riva et al.* 122 (MSM). • **Pasco**: Prov. Oxapampa, 2 km W of Puente Paucartambo, 800 m, 27 May 1979, *D.C. Wasshausen & F. Encarnación* 1129 (K, MO, US, USM); • ibid., Pozuzo, P.N. Yanachaga-Chemillén, Humpal, 10°10'58"S, 75°34'25"W, 1100 m, *A. Monteagudo et al.* 12501 (MOL); • ibid., Dist. Huancabamba-Pozuzo, Cañón de Huancabamba, 10°10'S, 75°35'W, 30 June 1985, *R. Foster et al.* 10363 (MOL). Prov. Oxapampa, Dist. Pozuzo, Yanachaga-Chemillén, sendero Robin Foster, 27 Aug. 2019, *Azevedo et al.* 147 (HOXA). • **San Martin**: Prov. Bellavista, Dist. Alto Biavo, Sector las Palmas, P. N. Cordillera Azul, puesto de Control 20 Mojarra, 07°25'26"S, 76°11'50.8"W, 772 m, 13 Sept. 2019, *L. Valenzuela et al.* 36844 (HOXA, USM). Prov. Huallaga, Mishquiyacu, cerca de Saposoa, 200–300 m, 29 Aug. 1948, *R. Ferreyra* 4639 (MO, US); • ibid., Saposoa, Centro Poblado de Shima, 450 m, Apr 2017, *M. Quispe* 29 (USM). Prov. Tocache [Mariscal Caceres], Dist. Tocache [Nuevo], Quebrada de Huaquisha, right bank of Río Huallaga, 16 May 1970, *J. Schunke Vigo* 3977 (K, US, USM); • ibid., 25 May 1970, *J. Schunke Vigo* 4014 (US, USM); • ibid., Fundo Porvenir, Río Huallaga, 3 Sept. 1970, *J. Schunke Vigo* 4316 (F, K, US, USM); • ibid., 400 m, 25 June 1974, *J. Schunke Vigo* 6998 (US); • ibid., 600–700 m, 27 March 1975, *J. Schunke Vigo* 8166 (US); • ibid., 500–600 m, 5 May 1975, *J. Schunke Vigo* 8385 (F, US); • ibid., Quebrada de Challauayacu, 480–500 m, 4 Feb. 1979, *J. Schunke Vigo* 10788 (MO); • ibid., Río Cañuto, cerca de Tanta, 500–520 m, 2 June 1980, *J. Schunke Vigo* 11760 (BR, JBRJ, US, USM); • ibid., Río de la Plata, fundo del Sr Manuel Gatica, 550–700 m, 12 Aug. 1980, *J. Schunke Vigo* 12155 (US); • ibid., Quebrada Cachiyacu de Huaquisha, 500–650 m, 5 Dec. 1980, *J. Schunke Vigo* 12446 (F, K, JBRJ, US, USM). **Ucayali**: Prov. Coronel Portillo, Dist. Calleria, Cuenca del Río Utiquinia, quebrada Espjoyacu, afluente de la quebrada Manuela, 7°56.67'S, 73°53.61'W, 300 m., 8 Sept. 2003, *J.G. Graham* 2637 (F, US, USM).

##### Notes.

The corolla resembles that of *Justiciagoudotii* V.A.W. Graham, but is typically longer, > 2 cm in length, the bracts oblong or elliptic, strongly apiculate, 8–15(–20) × 2–5(–8) mm (not lanceolate 4–6 mm long) conspicuously exceeding the 5-lobed calyx; leaves narrowly oblong-elliptic, 3–4 times as long as broad (not ovate-elliptic, 1.5–3 times as long as broad).

When describing *Beloperonesoukupii*, Standley & Barkley compared it with *B.cochabambensis* Rusby (=*Justiciaramulosa*), presumably being unaware of the existence of *J.radicans* and listed several characters which distinguish the two species, including the longer corolla and the broader more pilose bracts of *J.ramulosa. J.radicans*, in fact, largely replaces *J.ramulosa* in Peru.

*J.G. Graham* 2637 was collected in eastern Ucayali outside the main distributional range of *Justiciaradicans* (Fig. 59). It appears to be correctly identified but confirmation of its presence in this area is desirable.

Two specimens resemble *J.radicans* Vahl but are immediately distinguished from both by the long 5–7 mm acumen of the bracts (not 1–3 mm); additionally, the inflorescence is strictly terminal, not terminal and axillary as is usual in *J.radicans* and *J.rusbyi*.

##### Additional material examined.

**Peru** • **Pasco**: Prov. Oxapampa, Dist. Pozuzo, Puesto de Vigilancia Huampal, 10°11'S, 75°34'W. 1100 m, 11 Aug. 2003, *R. Rojas et al*. 1178 (MO); • ibid., P.N, Yanachaga-Chemillen, Puesto de Control. Huampal, 10°11'09"S, 75°34'12"W, 1300 m, 21 July 2006, *A. Monteagudo et al.* 12491 (MO).

#### ﻿﻿18. Justicia ramulosa

Taxon classificationPlantaeLamialesAcanthaceae

(Morong) C. Ezcurra, Bol. Soc. Argent. Bot. 25: 350. 1988. (Ezcurra 1988: 350)

14CD6FD1-5E61-5D87-87B5-FF8A10A5D7F0

Beloperone ramulosa Morong, Ann. New York Acad. Sci. 7: 194. 1893. (Morong and Britton 1893: 194) Type. PARAGUAY. Asunción, *T. Morong* 706 (holotype NY-00049760, isotypes BM-000549630, E-00104467, G-00102437, G-00102438, GH-00093738, K-000529297, MICH-1104016, MO-716370, NY-00311808, NY-00049760, PH-00102437, US-00478559).
Beloperone tetramerioides Lindau, Bull. Herb. Boissier 3: 488. 1895. (Lindau 1895: 488) Type. BOLIVIA. Santa Cruz, Río Yapacani, *Kuntze* s.n. (presumed holotype B†, photo of holotype F0BN008948, isotypes NY-00311811, NY-00311812 US-00137238).
Justicia tetramerioides (Lindau) V.A.W.Graham, Kew Bull. 43: 604. 1988. (Graham 1988: 604)
Beloperone velascana Lindau, Bull. Herb. Boissier 3: 489. 1895. (Lindau 1895: 489) Type. BOLIVIA. Santa Cruz, Velasco, *Kuntze* s.n. (presumed holotype B†, isotype NY-00311813).
Justicia magentea V.A.W.Graham, Kew Bull. 43: 603. 1988. (Graham 1988: 603) Type. Based on Beloperonevelascana Lindau.
Beloperone cochabambensis Rusby, Mem. Torrey Bot. Club 6(1): 103. 1896. (Rusby 1896: 103) Type. BOLIVIA, Chapare, *M. Bang* 1215 (holotype NY-00038821, isotypes BM-000992618, E-00104448, F-0077564F, G-00236359, GH-00093731, K-000529355, M-0186151, MICH-1104013, MO-716325, MIN-1000417, NY-00278982, NY-00278983, PH-00007757, S-03-2309, US-00137222, US-02880383, W-1892-000905, WIS-v0256364WIS).
Justicia cochabambensis (Rusby) V.A.W.Graham, Kew Bull. 43: 603. 1988. (Graham 1988: 603)
Beloperone pseudociliata Mildbr., Notizbl. Bot. Gart. Berlin-Dahlem 9: 1159. 1927. (Mildbraed 1927: 1159) Type. BOLIVIA. Santa Cruz, Buenavista, *Jose Steinbach* 7137bis (holotype B†, photo of holotype F0BN008938, isotypes BM-000992622, CAS-0005294, E-00346891, F-0077569F, G-00236206, G-00236361, K-000529353, MO-1403186, NY-00311807, PH-00007731, S-03-2306, U-0143634, UC-306463, US-01948831).
Justicia pseudociliata (Mildbr.) V.A.W.Graham, Kew Bull. 43: 603. 1988. (Graham 1988: 603)

##### Type.

Based on *Beloperoneramulosa* Morong

##### Diagnosis.

Very similar to *Justiciaradicans*, most obviously differing in the larger, dark reddish or magenta corolla 2.5–3.5 cm in length. Additional differences lie in the leaves, which are commonly rather abruptly narrowed at the base, in the bracts which are broader (to c. 8 mm), more conspicuously ciliate with white hairs and with apex merely apiculate. The corolla is larger in all parts, the lobes of the lower lip c. 3 × 3 mm. Also distinctive are the thecae, the lower not being separated by a gap from the upper and appearing to be fully developed.

##### Illustration.

Fig. 18.

**Figure 18.**
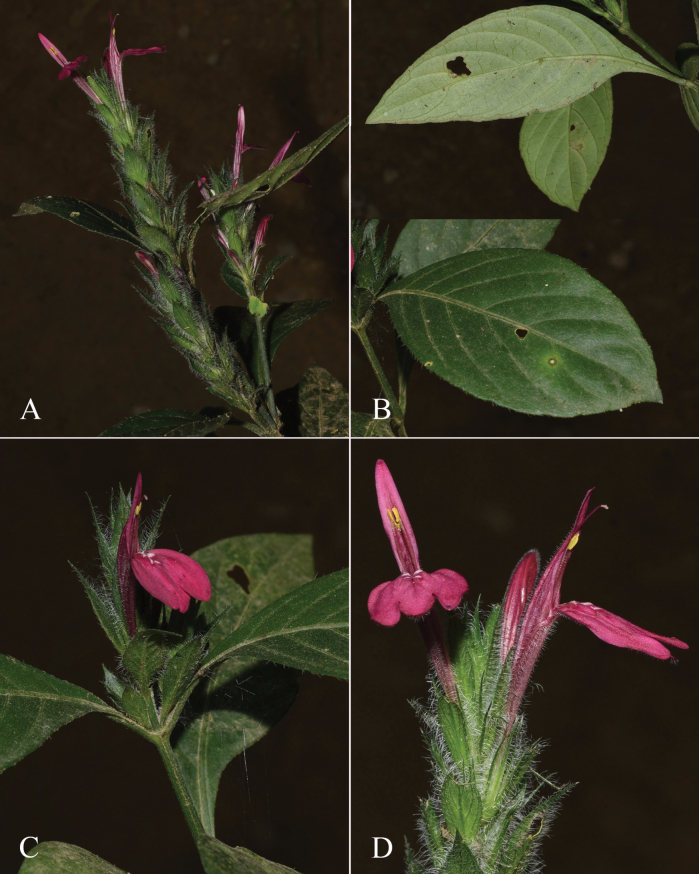
Photographs of *Justiciaramulosa* by Rosa Villanueva. Note characteristic dark red colour of the corolla.

##### Phenology.

Found in flower in May and June.

##### Habitat.

(in Peru). Humid forest 350–1900 m.

##### Distribution.

Frequent in parts of northern Argentina, Paraguay, southern Brazil and Bolivia, just entering the southernmost departments of Peru. Fig. 59.

##### Material examined.

**Peru** • **Madre de Dios**: Prov. Tahuamanu, km 32 Ibreria–Iñapari, 30 May 1978, *F. Encarnación* 1165 (US). Prov. Manu, Parque Nacional de Manu, 350 m, 23 July 1979, *R. Foster et al.* 6823 (F). Prov. Tambopata, Dist. Tambopata, km 7 Otilia, 12°31'43.6"S, 75°12'41.4"W, 240 m, 17 Aug. 2002, *J. Nina* 6 (HAG). • **Puno**: Prov. Sandia, between Putina and San Ignacio, 1100 m, 19 June 1986, *P. Nuñez & C. Muñoz* 5150 (CUZ); • ibid., 1900 m, 9 June 1982, *D.C. Wasshausen & A. Salas* 1237 (K, US); • ibid., Dist. San Juan del Oro, 1350 m, 6 June 1982, *D.C. Wasshausen & A. Salas* 1201 (K, US); • ibid., Dist. Limbani, Oconeque, 2200 m, 9 June 1974, *C. Vargas* 22567 (CUZ).

#### ﻿﻿19. Justicia angustituba

Taxon classificationPlantaeLamialesAcanthaceae

J.R.I.Wood & R.Villanueva sp. nov.

19C53C44-4C74-518E-BB52-803887C96206

urn:lsid:ipni.org:names:77363411-1

##### Type.

Peru • Cajamarca, Prov. San Ignacio Dist. Chirinos, Las Juntas, margen derecha del Río Tabaconas, 5°21'S, 78°46'W, 550–650 m, 3 Feb. 1996, *J. Campos & O. Diaz* 2380 (holotype F-2235748, isotypes MO-5297086, US-3387861, USM).

##### Diagnosis.

A new species resembling *Justiciagoudotii* but the leaves consistently smaller, 2–3 × 0.8–2.4 cm (not 4–12 × 1.5–6 cm), inflorescence terminal (not axillary and terminal), calyx shorter, 6–7 mm (not 8 mm long) and the capsule pubescent (not glabrescent).

##### Description.

Herb to c. 40 cm in height, stems at first decumbent and rooting, then erect, somewhat wiry below, crisped-pubescent above, glabrescent below. Leaves petiolate, small, lamina (1–) 2–3 × 0.8–2.4 cm, ovate, apex acute, base rounded and very shortly decurrent onto the petiole, cystoliths abundant adaxially, both surfaces pubescent but abaxially more densely so, paler, lateral veins 4–5 pairs; petioles 0.2–0.8 cm, pubescent. Inflorescence of subsessile, few-flowered clusters terminal on the branches; peduncles 0–2 mm, hirsute; pedicels 1–2 mm; bracts 10 × 3 mm, narrowly oblong-elliptic, narrowed at both ends, apex acuminate and acute, ciliate on margins and veins; bracteoles linear 3 × 0.5 mm; calyx 4-lobed to base, lobes 8–10 × 1 mm, linear-lanceolate, acuminate, slightly narrowed to base, hirsute; corolla c.1.5 cm long, pink, pubescent, tube c. 10 × 1 mm, slender, cylindrical, upper lip short, deltoid, hooded, subentire, c. 4 mm long, lower lip 3-lobed, the palate with white “herring bone” patterning, the lobes c. 6 × 2.5 mm, oblong, rounded; filaments 2 mm long, glabrous, anther thecae 1.25 × 0.5 mm, oblong, glabrous, lower held at right angles; pollen prolate, 50–54 × 29–30 μm, 2-aperturate, colporate, 2 rows of c. 8–12 of insulae on either side of aperture (Fig. 50B); ovary, glabrous. Capsule 10 × 3 mm, clavate, thinly pubescent, 4-seeded; seeds rounded, c. 2 mm diam., smooth, glabrous.

##### Illustration.

Fig. 19.

**Figure 19.**
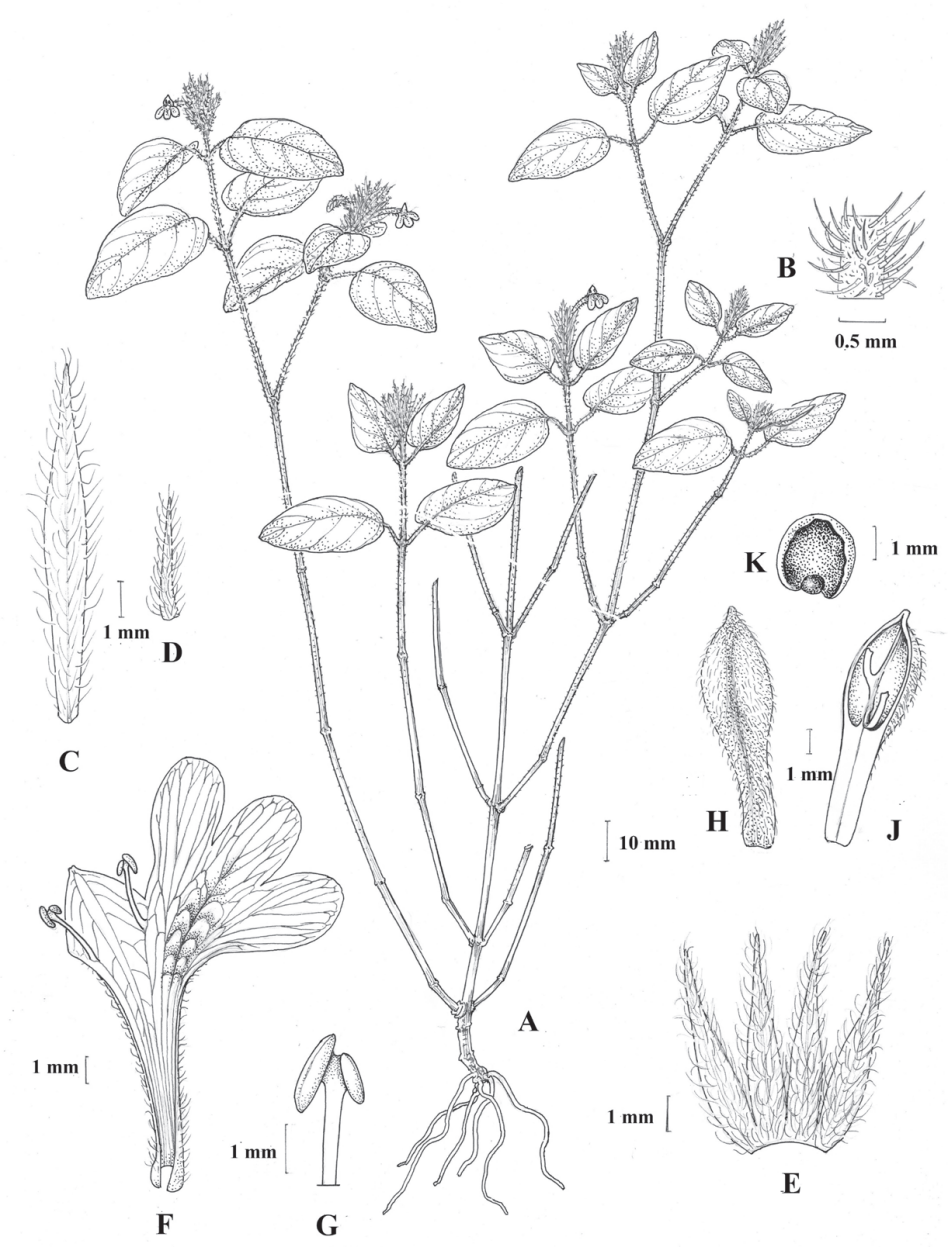
*Justiciaangustituba***A** habit **B** detail of stem **C** bract **D** bracteole **E** calyx **F** corolla **G** anther **H** exterior of capsule valve **J** interior of capsule valve; K seed. Drawn from *Campos & Diaz* 2380 by Margaret Tebbs.

##### Etymology.

This species is given the epithet “*angustituba*” because of its distinctively slender corolla tube.

##### Phenology.

Found in flower in February and June.

##### Habitat.

Secondary woodland, 450–650 m.

##### Distribution.

Endemic to San Ignacio Province in Cajamarca Department, Peru. Fig. 59.

##### Material examined.

**Peru** • **Cajamarca**: Prov. San Ignacio, Dist. Chirinos, Las Juntas, the type collection; • ibid., Las Juntas, 5°22'48"S, 78°46'58"W, 450 m, 1 June 2000, *R. Rojas et al.* 0892 (MO).

Species 20–23. The Simonisia clade. Species with a 5-lobed calyx, smooth seeds, a pubescent capsule and long, slender bracts and bracteoles.

#### 20. Justicia riedeliana

Taxon classificationPlantaeLamialesAcanthaceae

(Nees) V.A.W. Graham, Kew Bull. 43(4): 605. 1988. (Graham 1988: 605)

F53B8CC1-4A83-51E8-9165-67B764D1E152

Simonisia riedeliana Nees, Flora Bras. 9: 145. 1847. (Nees 1847a: 145) Type. BRAZIL. Río Madeira, *Riedel* 1332 (lectotype GZU000250368, designated here, isolectotype LE?, n.v., NY-00278265, US-2880507, fragment).
Chaetochlamys macrosiphon Lindau, Bull. Herb. Boissier 3: 490. 1895. (Lindau 1895: 490) Type. BOLIVIA. Between Cochabamba and Chimore, *Kuntze* s.n. (presumed holotype B†, photo of holotype FOBN008899, lectotype US-00137244 (Cat. No.701835), designated by Wasshausen and Wood 2004: 74, isolectotypes NY-00311862, NY-00311863).
Justicia macrosiphon (Nees) V.A.W. Graham, Kew Bull. 43(4): 605. 1988. (Graham 1988: 605)
Beloperone bangii Rusby, Mem. Torrey Bot Club 6: 104. 1896. (Rusby 1896: 104) Type. BOLIVIA. Cochabamba, *M. Bang* 1224 (holotype NY-00278981, isotypes BM-000617754, BR-0000008424488, CAS-0000978, E-00346889, F-0077563F, GH-00093730, K-000529352, M-0186155, MO-716326, NY-00431317, PH-00007733, US-00478553, WIS-v0256365WIS).

##### Type.

Based on *Simonisiariedeliana* Nees

##### Description.

Subshrub 40–60 cm high, stems glabrous. Leaves petiolate, lamina mostly 7–15 × 3–6.5 cm, elliptic, apex shortly acuminate, base, abruptly narrowed then attenuate and decurrent on the petiole, lateral veins c. 7 pairs, glabrous; petioles up to 4 cm. Inflorescence of short dense axillary and terminal spikes, c. 3–5 cm long; bracts and bracteoles linear-setaceous 2.5–4 × 0.1 cm, puberulent; calyx subequally 5-lobed to base, lobes 14–17 × 1.5–2 mm, lanceolate, finely acuminate, puberulent; corolla pubescent, 2-lipped, basal cylindrical tube 30–40 × 2 mm, white, widened slightly in upper 5 mm, lips pink, upper lip lanceolate, commonly recurved, 10–15 mm long, lower lip with “herring bone” patterning, 3-lobed, 12–20 mm long, deeply 3-lobed, lobes obovate, rounded, 13–17 × 7–12 mm; anther thecae strongly superposed, 2 × 0.75 mm, separated by c. 1 mm, parallel, the lower with a short basal appendage. Capsule 20 × 6 mm, woody, puberulent, clavate, 4-seeded; seeds smooth.

##### Illustration.

Fig. 20C, D.

**Figure 20.**
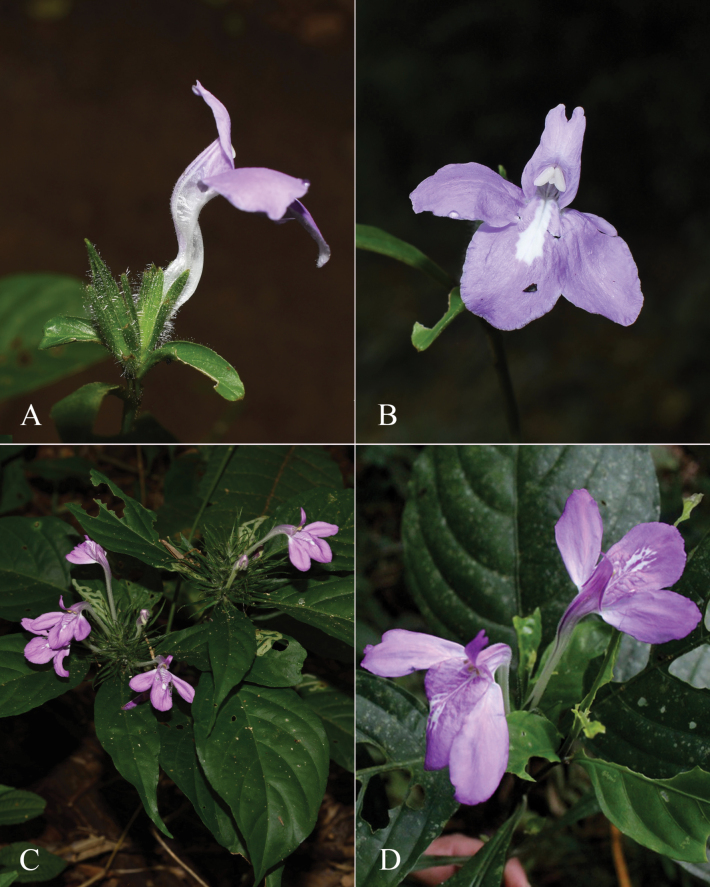
Photographs of **A, B***Justiciarusbyi* (*Villanueva* 1062) Note relatively stout, characteristically bent corolla tube **C, D***Justiciariedeliana* Note slender, straight, cylindrical corolla tube. **A**–**B** Rosa Villanueva, **C**–**D** Modesto Zarate.

##### Phenology.

Flowering from April to October.

##### Habitat.

Lowland rainforest up to c. 600 m.

##### Distribution.

Southern Amazon basin in Bolivia, Brazil, Bolivia and Peru, where it is restricted to Madre de Dios and Ucayali. Fig. 60.

##### Material examined.

**Peru** • **Madre de Dios**: Explorer´s Inn, 39 km SW. of Puerto Maldonado, near the confluence of Río Tambopata and Río La Torre, 12°50'S, 69°20'W, 8 July 1987, *S.F. Smith et al.* 911 (US, USM); • ibid., Laguna Tres Chimbadas 1 km al Río Tambopata, 12°47.24'S, 69°19.95'W, 200 m, 9 July 1998 *F.A. Michelangeli* 472 (USM). Prov. Tambopata, road to Tambopata, N. of Puerto Maldonado, 250 m, 21 April 1977, *Al. Gentry et al.* 19577 (F, US); • ibid., small tributary of Río Madre de Dios, 1 hour below Puerto Maldonado, 250 m, 22 April 1977, *Al. Gentry et al.* 19651 (US); • ibid., Tambopata Nature Reserve, 12°49'S, 69°17'W, 260 m, 7 May 1980, *P.J. Barbour* 5164 (US); • ibid., along main trail to Lago Cocacocha, 280 m, 6 Oct. 1981, *V.A. Funk* 3327 (US); • ibid., Ríos Torre y Tambopata, 12°49'S, 69°40'W, 270 m, 21 July 1984, *Al. Gentry et al.* 51062 (US, USM); • ibid., 30 km by air SSW Puerto Maldonado at effluence of Río La Torre (Río D’Orbigny), Río Tambopata, 22 May 1986, *V.A. Funk et al.* 8102 (US); • ibid., Sonene, 12°33'36.5"S, 68°42'39.0"W, 200 m, 14 May 1999, *P. Nuñez et al.* 25751 (CUZ); • ibid., Los Amigos Biological Station, Madre de Dios river, 167 m, 20 Sept. 2004, *A. Maceda* 1576 (BRIT); • ibid., Dist. Tambopata, 39 km, SW of Puerto Maldonado, 12°50'S, 69°20'W, 11 July 1987, *S.F. Smith et al*. 963 (F, K, U, US, USM); • ibid., Tambopata Nature Reserve, 12°15'S, 69°17'W, 260 m, 6 Oct. 1993, *F. Cornejo et al*. 1231(MOL); • ibid., El Castañal, 12°38'29.2’'S, 69°15'34"W, 240 m, 18 Aug. 2002, *G. Gonzales* 34 (HAG); • ibid., Isuyama, 12°37'S, 69°11'W, 220 m, 20 June 2003, *A. Colquesaña* 35 (HAG); • ibid., Dist. Las Piedras, Cuzco Amazónico, 12°29'S, 69°03'W, 14 Sept. 1991, *M Timaná & A. Rubio* 2271 (CUZ). • **Ucayali**: Prov. Coronel Portillo, Iparia, Cuenca del Río Iparia, afluente del Río Ucayali, 9°21'38"S, 74°29'23"W, 240 m, 25 July 2007, *J.G. Graham & J. Schunke* 4342 (MOL, US).

##### Lectotypifications.

Nees cited two collections as syntypes of *Simonisiariedeliana*, *Riedel* s.n from the Río Madeira and *Poeppig* s.n. from Maynas, both present in Nees’ own herbarium currently at GRAZ. Although the Poeppig specimen from Maynas is much the better of the two, Nees considered it as atypical commenting “specimen fructiferum bracteis bracteolisque maximis” contemplating treating it (in sched.) as "var. grandibractea. Consequently, it seems best to exclude this from consideration as lectotype. The cited locality (Maynas) is also odd as this species has not been rediscovered in Loreto or San Martin.

#### ﻿﻿21. Justicia sprucei

Taxon classificationPlantaeLamialesAcanthaceae

V.A.W. Graham, Kew Bull. 43(4): 606. 1988. (Graham 1988: 606)

D6E544FC-4975-5F7A-801A-212C3412C8B2

Chaetochlamys ciliata Lindau, Bull. Herb. Boissier 5(8): 677. 1897. (Lindau 1897: 677) Type. BRAZIL. Para, Santorem, *Spruce* s.n. (holotype B†, photo of holotype F0BN008898, lectotype K-000529280, designated by Wasshausen and Wood (2004:77) with second step lectotypification here as the original lectotypification did not specify sheet, isolectotypes BM-000992629, E-00132480, K, NY-00311860, OXF-00009053).

##### Type.

Based on *Chaetochlamysciliata* Lindau

##### Description.

Herb reaching c. 1 m in height, stems bifariously puberulent. Leaves shortly petiolate, lamina 6–15 × 2–4 cm, oblong, long-acuminate, base attenuate, glabrous except for the veins; petioles 0–12 mm. Inflorescence subcapitate arising from the uppermost leaf axils, the heads often restricted to a terminal pair subtended by reduced leaves; peduncles very short, 2–8 mm; bracts 12–15 × 2 mm, lanceolate, acuminate, long-ciliate with white hairs; bracteoles similar but narrower; calyx 5-lobed, lobes 8–10 × 1 mm, lanceolate, finely acuminate, ciliate; corolla c. 35 mm long, white to pale purple, puberulent, the tube c. 2.5 cm long with a slightly bulbous base, then narrowed to c. 2 mm before gradually widening after 15 mm, lower lip with rounded lobes 6–7 × 6–8 mm; anther thecae superposed, oblong, parallel, 3 × 1 mm, the lower with a short basal appendage. Capsule 15 × 3–4 mm, ellipsoid with a long sterile base; seeds glabrous, smooth.

##### Phenology.

Found in flower in May.

##### Habitat.

Lowland rain forest often near streams.

##### Distribution.

Amazonian Brazil extending southwards to Rondonia, northern Bolivia and southwestern Peru and northwards into French Guyana; apparently uncommon. A new record for Peru. Fig. 60.

##### Material examined.

**Peru** • **Madre de Dios**: Prov. Tahuamanu, km 62 carretera Iberia-Iñapari, cerca del aeropuerto, 25 May 1978, *F. Encarnación* 1152 (K, MO, US).

##### Note.

The few subcapitate, mostly terminal inflorescences with ciliate bracts are very distinctive.

#### ﻿﻿22. Justicia longibracteata

Taxon classificationPlantaeLamialesAcanthaceae

J.R.I.Wood & R.Villanueva sp. nov.

6D0C86AF-1902-521D-A6AC-87FB2CDA3B4E

urn:lsid:ipni.org:names:77363412-1

##### Type.

**Peru** • [Loreto], “Prope Yurimaguas, ad flumen Huallaga, Peruviae orientalis,” May 1855, *R. Spruce* 3868 (holotype K-000202092 ex Herb Hooker, isotype K-000202093 ex Herb. Bentham, this additionaly labelled “in sylvis praecipue recontioribus”

##### Diagnosis.

Similar to *Justiciamiguelii* V.A.W.Graham but differing as follows: leaves clearly petiolate with petioles 2–4.5 cm (not subsessile or with petioles < 10 mm); bracts linear/subulate 5.5–7 cm long, 0.5–1 mm wide at base (not c. 3 cm long and 1–3 mm wide; calyx lobes longer and narrower, c. 17–21 × 1.5 mm, drawn out gradually to a fine point (not 15–17 mm long and 3 mm wide, abruptly narrowed and ± cuspidate apically); corolla shorter 5–5.5 cm long (not up to 7 cm long), clearly exceeded by the bracts (not clearly exceeding the bracts). Also, somewhat resembling *J.riedeliana* but distinguished by the different shaped corolla with a gradually widened tube (not subcylindrical), longer bracts and calyx lobes.

##### Description.

Subshrub 0.5–1.5 m in height; stems ± terete, obscurely bifariously crisped puberulent. Leaves equal in each pair, petiolate, lamina 6–17 × 3–6 cm, apex acuminate, base attenuate and slightly decurrent, both surfaces subglabrous, adaxially with small cystoliths, abaxially the venation not very prominent with c. 6 lateral pairs, midvein puberulent when young, glabrescent; petioles 2–4.5 cm, glabrescent. Inflorescence of dense terminal spikes c. 8–12 cm long and 10 cm broad because of abundant linear bracts, the flowers imbricate; rhachis pubescent; peduncles 0–1.5 cm; bracts and bracteoles similar, 55–70 × 0.5–1 mm, linear, hirsute with long trichomes mixed with shorter trichomes; calyx 5-lobed to base, lobes erect, 17–21 × 1–1.5 mm, lanceolate, gradually narrowed to a long, fine apex, pubescent; corolla 5–5.5 cm long, pinkish-purple, pubescent in bud, tube 3 mm wide at base, gradually widened to c. 7 mm after 3.5 cm, 2-lipped, upper lip lanceolate, bifid, lower lip 3-lobed, the lobes ovate, rounded, c. 10 × 8 mm; anther thecae oblong, 1.5 × 0.5 mm, strongly superposed, glabrous, the lower with a basal appendage; ovary glabrous. Capsule and seed not seen.

##### Illustration.

Fig. 21.

**Figure 21.**
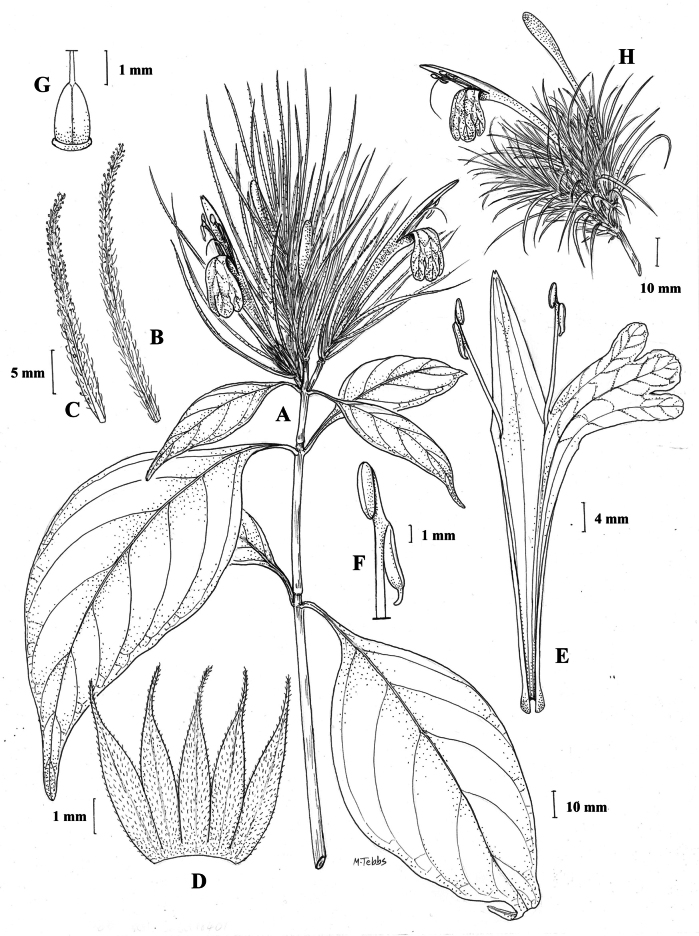
*Justicialongibracteata***A** habit **B** bract **C** bracteole **D** calyx **E** Corolla opened out to show stamens **F** anther **G** ovary. *Justiciamiguelii***H** inflorescence showing bracts and corolla. **A**–**G** drawn from *Spruce* 3868 **H** from *Wood* 18401 by Margaret Tebbs.

##### Etymology.

This species is named “*longibracteata*” because of the exceptionally long bracts, which are its most distinctive feature.

##### Phenology.

Found in flower from May to October.

##### Habitat.

Lowland rainforest, c. 150–210 m.

##### Distribution.

Endemic to Amazonian Peru and apparently restricted to the Yurimaguas area. Fig. 60.

##### Material examined.

**Peru** • **Loreto**: Prov. de Alto Amazonas, Dist. Yurimaguas, Yurimaguas, River Huallaga, the type; • ibid., Aug. 1902, *E. Ule* 6285 (K, L); • ibid. lower Río Huallaga, 155–210 m, Oct.–Nov. 1929, *Ll. Williams* 4094 (F); • ibid., 22 Oct. 1929, *Ll. Williams* 3900 (F); Prov. Requena, Dist. Requena, Requena, 200–207 m, Oct. 2008. *M. Ocrospoma* 084 (USM).

#### ﻿﻿23. Justicia rusbyi

Taxon classificationPlantaeLamialesAcanthaceae

(Lindau) V.A.W. Graham, Kew Bull. 43(4): 605. 1988. (Graham 1988: 605)

80833814-671F-50A0-B67A-07477EBDBC30

Chaetochlamys rusbyi Lindau, Bull. Herb, Boissier 3: 491.1895. (Lindau 1895: 491) Type. BOLIVIA. [La Paz, Larecaja,] prope Guanai, *H.H. Rusby* 1117 (lectotype BM-000617757, designated here, isolectotypes F-007757F, K-00529479, MO-1999310, NY-0003882, NY-00038824, NY-00049762, US-000617757, US-00137245, US-02880667).
Ruellia lanceolata Morong, Ann. New York Acad. Sci. 7: 193. 1893 (Morong and Britton 1893: 193), non Justicialanceolata (Chapman) Small. Type. PARAGUAY. Between Pirayu and Jaguaron, *T. Morong* 667 (holotype NY-00312333, isotypes F-0077595F, TEX-00373085, US-00432509).
Beloperone matthewsii Lindau, Bull. Herb. Boissier 6, app. 1: 30. 1898. (Hassler 1898: 30). Type. PARAGUAY. Cordillera de Los Altos, *E. Hassler* 1936 (lectotype G, designated by Wasshausen and Wood (2004: 76) without barcode, second step lectotypification with barcode G-00102447, designated here, isolectotypes, G-00102445, G-00102446).
Justicia matthewsii (Lindau) Rusby ex Dyer, Index Kew. 99. 1904. (Dyer 1904: 99)

##### Type.

Based on *Chaetochlamysrusbyi*.

##### Description.

Much branched subshrub commonly 30–130 cm high; young stems slightly glaucous, sulcate, bifariously pubescent. Leaves shortly petiolate, lamina 4–14 × 2.5–5 cm, oblong-elliptic, acuminate at both ends, adaxially glabrous with conspicuous cystoliths, abaxially paler with 7–10 pairs of prominent side veins, glabrous or pubescent; petioles glabrous or pubescent. Inflorescence of flowers and flower clusters subsessile in the axils of the upper leaves; bracts lanceolate, finely acute, 6–17 × 3–5 mm, thinly ciliate, bracteoles similar, but narrower; calyx 5-lobed to base, lobes 15–20 × 2–3 mm, narrowly oblong-lanceolate, finely acuminate, pubescent, sometimes bristly-glandular in fruit; corolla 2.5–5 cm long, thinly glandular hirtellous, the tube white, commonly flexuose, relatively stout, c. 20–22 × 5–8 mm, limb deep pinkish-purple, the upper lip obovate, bifid, 10 mm long, the lower lip with “herring bone” patterning, broad, spreading, deeply 3-lobed, the lobes 15 × 10 mm, ovate, rounded; pollen subprolate, 2-aperturate (Graham 1988: 568). Capsule 17–22 × 5–8 mm, clavate, glabrous, 4-seeded; seeds smooth, 4 × 4 mm.

##### Illustration.

Fig. 20A, B.

##### Phenology.

Principally from February to July, very rarely outside these months.

##### Habitat.

Forest both moderately dry and humid, 100–1200 m.

##### Distribution.

Paraguay, southern Brazil, Bolivia and Peru. In Peru frequent on the eastern Andean slopes. Fig. 60.

##### Material examined.

**Peru** • Sin loc. *McLean* s.n. (K). • **Ayacucho**: Estrella, between Huanta and Río Apurímac, 500 m, 8–14 May 1929, *E.P. Killip & A.C. Smith* 22648 (F, US). Prov. De La Mar, Dist. Ayna, between Machente and Rosario, c. 8 km E of Ayna, 1100 m, 4 June 1975, *D.C. Wasshausen & F. Encarnación* 512 (FLAS, K, MO, US); • ibid., Anco, Villa Unión, 8 km to the NNW of San Antonio, 12°52'45.9 “S 73°33'12.1"W, 938 m, 28 April 2007, *J. Roque* 5560 (USM). **Cusco**: Prov. La Convención, 20 Feb. 1940, 1200 m, *C. Vargas* 1822 (CUZ); • ibid., Dist. Echarate, Palma Real, Koribeni, 12°38'30"S, 72°49'45"W, 718 m, 16 July 2007, *G. Calatayud et al.* 4315 (MO); • ibid., Palma real a Koribeni, 750–850 m, 17 April 1966, *C. Vargas* 17324 (CUZ, US); • ibid., Río Picharí, 2 km E of Colonization Pichari, 620 m, 13 June 1975, *D.C. Wasshausen & F. Encarnación* 545 (K, US); • ibid., Cumpire-Quiteni, 740 m, 12 May 1987, *L. van der Hoogte & C. Roersch* 3142 (U); • ibid., Río Maguriari (Manguyari), Alto Urubamba, upstream to Río Manguriari, 12°47'S, 72°40'W, 750 m, 2 Feb. 1991, *P. Nuñez & G. Ortiz* 12929 (MO); • ibid., Alto Manguariari, 700 m, *G. Ortiz* 62 (CUZ-027898); • ibid., Quepashato, 12°39'47"S, 73°16'13"W, 800 m, 26 March, *L. Valenzuela et al*. 9439 (CUZ); • ibid., Dist. Quiteni, 12°38'28"S, 73°04'18"W, 600 m, 19 July 2004, *W. Galiano et al.* 6675 (CUZ). • **Huánuco**: Prov. Puerto Inca, Dist. Tournavista, Ganso Azul, Río Pachitea, 1500 ft, Oct. 1942, *C. Sandeman* 3318 (K). Prov. Leoncio Prado, Dist. Luyando, Tulumayo entre Tingo María y Divisoria, 700–750 m, 24 July 1948, *R. Ferreyra* 4336 (MO, US, USM); • ibid., Dist. Rupa-Rupa, Tingo Maria, left bank of Río Huallaga, 700–800 m, 5 April 1976, *T. Plowman* 5818 (US, USM).Prov. Pachitea, Honoria, carretera Miel de Abejas, a 2 km. del campamento de Iparia, 300–400 m, 5 May 1967, *J. Schunke* 1926 (F, K, US, USM); • ibid., Ayamiria a 7 km del campamento de Iparia, 14 March 1967, *J. Schunke* 1778 (F, US, USM); • ibid., 11 April 1967, *J. Schunke* 1841 (US). • **Junín**: Prov. Chanchamayo, río Chanchamayo valley, between San Ramón and Puente Paucartambo, 11°00'S, 75°20'W, 750 m, *D. Smith et al*. 1405 (MO, US, USM); • ibid., Dist. Chanchamayo, Río Chanchamayo, Vic. La Merced, 750 m, 23 May 1979, *D.C. Wasshausen & F. Encarnación* 1084 (K, US, USM); • ibid., La Merced, 2000 ft, 10–24 Aug. 1923, *F. Macbride* 5356 (US); • ibid., wooded valley, 700 m, 20 May–4 June 1929, *E.P. Killip & A.C. Smith* 23449 (US); • ibid., Cañón del Río Colorado, 10 km N of La Merced, 10°59'S, 75°20'W, 800 m, 3 March 1991, *Al Gentry & C. Diaz* 73304 (MO); • ibid., Dist. Perené, Río Paucartambo Valley, near Perené Bridge, 700 m, 19 June 1929, *E.P. Killip & A.C. Smith* 25381 (P, US); • ibid., Dist. San Ramón, 750 m, 28 May 1979, *D.C. Wasshausen & F. Encarnación* 1137 (K, MO, US, USM); • ibid., 11°07'17"S, 75°20'7.1"W, 772 m, 17 Aug. 2010, *Xue-Jun Ge et al.* 274 (USM); • ibid., Dist. Vitoc, cerca a Monobamba, 29 May 1983, M. Vargas & R. Fernandez 85 (US, USM). Prov. Satipo, Along road between La Merced and Satipo, vicinity of Río Negro at Ipoki steep cliffs E of Río Pichanaki, 11°10'38"S, 74°39'32"W, 1200 m, 10 June 1998, *T. Croat & M. Sizemore* 82012 (MO, US, USM). **Loreto**: Prov. Maynas, Dist. Alto Nanay, San Antonio, on Río Itaya, 110 m, 18 Sept. 1929, *E.P. Killip & A.C. Smith* 29432 (F, US); • ibid., Dist. Punchana, Iquitos, Santa Maria de Ojeal, 19 Aug. 1973, *S. McDaniel & M. Rimachi* 17886 (US); • ibid., Dist. Mazán, trail from Río Amazonas to Río Napo at Mazán, 100–150 m, 16 April 1973, *M. Rimachi* 201 (US); • ibid., Dist. Indiana, Explorama Inn, 1 km S of Indiana, Río Amazonas, 03°30'S, 73°11'W, 130 m, 16 June 1986, *Al. Gentry et al.* 54575 (US); • ibid., Dist. Belén, Padre Isla, just below Iquitos, 1230 m, 22 May 1978, *Al. Gentry et al.* 22238 (MO). Prov. Ucayali, Dist. Pampa Hermosa, P. N. Cordillera Azul, Quebrada Yanayacu, 07°02'21"S, 75°48'36"W, 257 m, 25 May 2018, *L. Valenzuela et al.* 35264 (HOXA, MO, MOL, USM); • ibid., 07°21'33.3"S, 75°59'29.1"W, 565 m., 27 May 2021, *L. Valenzuela et al.* 39807 (HOXA); • ibid., *L. Valenzuela et al.* 35275 (HOXA, HUT, MO, USM); • ibid., 07°02'20.7"S, 75°48'35.9"W, 25 May 2018, *L. Valenzuela et al*. 35278 (HOXA, MOL, USM); • ibid., Dist.Contamana, road to oriente, 160–200 m, 28 July 1970, *S. McDaniel* 14115 (US, USM). **Pasco**: Prov. Oxapampa, 3 km E of Puente Paucartambo, 900 m, 27 May 1979, *D.C. Wasshausen & F. Encarnación* 1123 (FLAS, K, US). • **San Martin**: Alto Río Huallaga, 360–900 m, Dec. 1929, *Ll. Williams* 6233 (F). Prov. Lamas, along Quebrada Vainilla, c. ½ km downstream on Río Mayo from Puente Bolivia (km 33 of Marginal W of Tarapoto), 350 m, 30 May 1986, *S. Knapp & P. Alcorn* 7417 (US, USM). Prov. San Martín, trail to Boca Toma del Shilcayo, along Río Shilcayo N of Tarapoto, 06°30'S, 76°22'W, 400 m, 20–21 May 1986, *S. Knapp & P. Alcorn* 7325 (US, USM); • ibid., near Shapaja, 400 m, 2 Oct. 1973, *R. Ferreyra et al*. 18272 (USM); • ibid., Tarapoto, 1835, *A. Mathews* 1525 (K); • ibid., Aug. 1855, *R. Spruce* s.n. (K); • ibid., 5–8 km E of Tarapoto, 520 m, 6 May 1979, *D.C. Wasshausen & F. Encarnación* 1020 (K, MO, US, USM); • ibid., Dist. San Antonio, 6°24'54"S, 76°23'07"W, 470 m, 15 Aug. 2023, *R. Villanueva et al.* 1062 (HOXA). Prov. Tocache [Mariscal Cáceres], Arriba de Tarapoto, 600–700 m, 31 Aug. 1968, *R. Ferreyra* 17433 (USM); • ibid., Dist. Uchiza, Cachiyacu de Lepuna, 450–500 m, 10 July 1974, *J. Schunke* 7304 (F, MO, USM). • **Ucayali**: Prov. Coronel Portillo, Dist. Calleria, cuenca del Río Utiquinia, cabecera de la quebrada Espejoyacu, Cerro Espajoyacu, 7°57.81'S, 73°53.98'W, 800 m, 7 March 2003, *J. Graham* 2401 (F, MOL, US); • ibid., Dist. Pucallpa, Cerca Portrero 45, IVITA, 200 m, 11 May 1973, *R. Ferreyra* 18149 (US). Prov. Purús, Río Curanja, cerca el pozo grande entre las comunidades de Alta y Columbiana, 10°04.121'S, 71°08.555'W, 325 m, 24 July 1998, *J. Graham* 657 (F, US).

##### Lectotypification.

Lindau cited *Rusby* 1117 and *Kuntze* s.n. from Santa Cruz as types of *Chaetochlamysrusbyi*. Assuming he saw these collections in Berlin, they would have been destroyed in 1943 so *Rusby* 117 at the BM has been selected as lectotype. It is not a holotype as suggested by Tropicos. Wasshausen and Wood (2004) cited Hassler 1936 (G) as lectotype of *Beloperonematthewsii*, but there are three sheets at G so a unique sheet (G-00102447) is here designated as second step lectotypification.

Species 24–27. ﻿A possibly natural group of dry country plants with 5-lobed calyx, white flowers and a tendency to have 2-seeded capsules, verruculose pollen and smooth seeds.

#### ﻿﻿24. Justicia reginaldii

Taxon classificationPlantaeLamialesAcanthaceae

Wassh., Fl. Ecuador 89: 180. 2013. (Wasshausen 2013: 180)

F43FBC57-BFA0-58CE-A2F8-BEBE197E4F7D

##### Type.

Ecuador • Camino Zaruma–Loja, 1100 m, May 1958, *R. Espinosa* 2409 (holotype US-01106126, isotype K-000544761).

##### Description.

Subshrub to 70 cm in height; stems puberulent when young. Leaves very shortly petiolate, lamina 2.5–5 × 1.5–2.5 cm, lanceolate to ovate, apex obtuse, base cuneate, pubescent; petioles. Inflorescence of terminal bracteate spikes, the flowers in very short axillary spikes, commonly reduced to subsessile opposite pairs; bracts foliose below, diminishing in size to 6 × 2 mm upwards; calyx 5-lobed to about three quarters its length, lobes oblong, 4–5 × 1 cm, glabrous or puberulent; corolla relatively large, 2.2–2.5 cm long, gaping, white with purple markings, puberulent, the tube 12–14 mm long, upper lip erect, hooded, notched, lower lip 3-lobed, the lobes c. 6 mm long, rounded; anther thecae parallel, slightly superposed, oblong, 2.5 × 0.75 mm, the lower basally apiculate; pollen prolate-perprolate 43–47 × 27–29 μm, 2-aperturate, porate, grain covered in verrucae (Fig. 50C). Capsule 7 mm long, subglobose, glabrous, 2-seeded.

##### Illustration.

Fig. 22.

**Figure 22.**
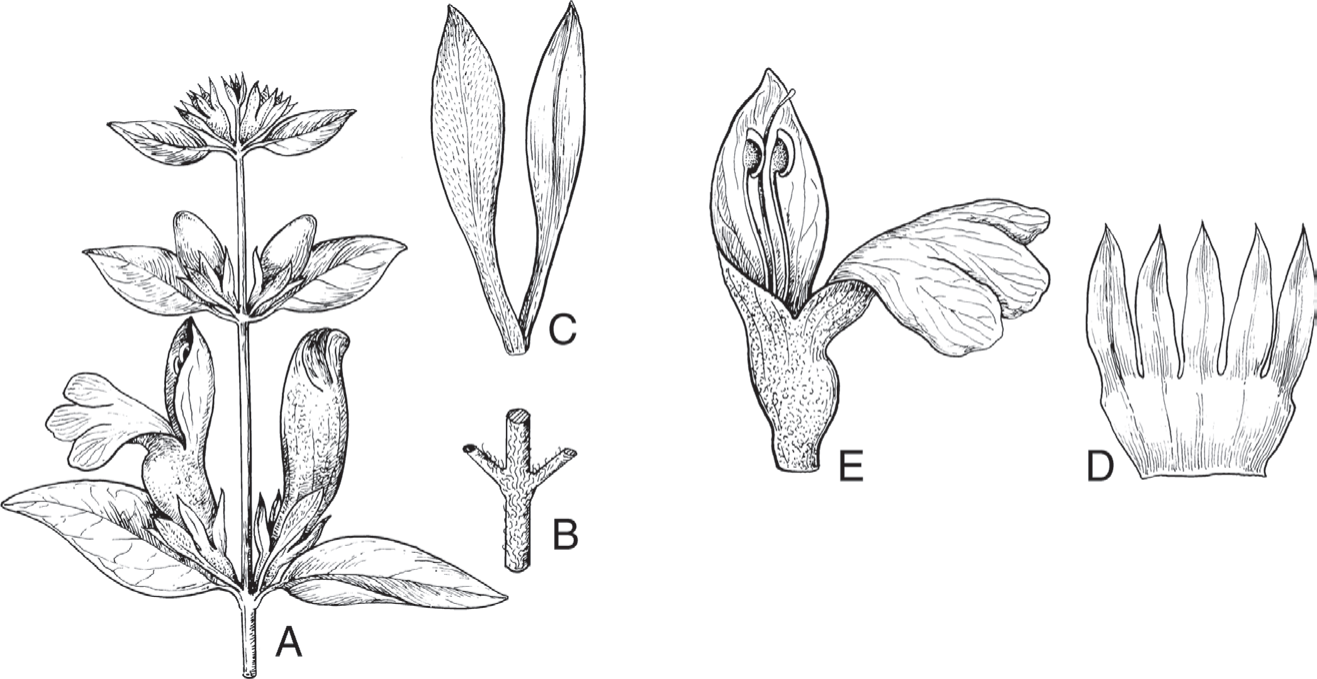
*Justiciareginaldii***A** inflorescence **B** stem, detail **C** upper bracts **D** calyx **E** corolla. Drawn from *Ferreyra* 7070 by Alice Tangerini.

##### Phenology.

March to May.

##### Habitat.

Rocky scrubby mountain sides, 1600–1700 m (1100 m in Ecuador).

##### Distribution.

A rare species restricted to southern Ecuador and northern Peru. New record for Peru. Fig. 61.

##### Material examined.

**Peru** • **Cajamarca**: Prov. Cajamarca, Dist. Matará, entre Tingo y San Miguel de Asunción, Chileto, [7°16'S, 78°16'W], 1600–1700 m, 10 April 1950, *R. Ferreyra* 7070 (K, MO, US).

##### Note.

The following specimens from dry forest dominated by *Eriotheca* Schott & Endl. at 2137–2281 m in Ancash and Huánuco differ in the smaller corolla c. 1.5 cm long, shorter capsule (4–5 mm long) and slightly smaller calyx. However they share the same terminal inflorescence with axillary flowers, the same gaping corolla and 2-seeded capsule. The leaves are similar in shape but are very variable in size. They may simply be a variant of the poorly known *Justiciareginaldii*.

##### Additional material examined.

**Peru** • **Ancash**: Prov. Sihuas, Dist. Acobamba, entre Quiches y Jocos, 0.96 km NW del Puente Isabel Gonzales Lozano, 8°20'36.5"S, 77°31'28.9"W, 2153 m, 6 March 2021, *P. González et al.* 7656 (E, USM); • ibid., 1.53 km NW del Puente Isabel Gonzales Lozano, 8°20'20.7"S, 77°31'38.7"W, 2281 m, *P. González et al.* 7634 (E, USM). **Huánuco**: Prov. Huacaybamba, Dist. Huacaybamba, right bank of Río Marañón, 9°2'9.2"S, 76°59'50.2"W, 2137 m, 25 March 2021, *P. González et al.* 9449 (E, USM).

#### ﻿﻿25. Justicia baguensis

Taxon classificationPlantaeLamialesAcanthaceae

J.R.I.Wood & R.Villanueva sp. nov.

C7E063B7-4BEF-531E-933B-CB83CA5FAA30

urn:lsid:ipni.org:names:77363413-1

##### Type.

Peru • Amazonas, Prov. Bagua, Bagua Grande–Pedro Ruiz Road, 500–1000 m, 10 March 1998, *H. van der Werff* 14604 (holotype MO-5763982, isotypes F-2236803, MOL, USM).

##### Diagnosis.

Resembling *Justiciareginaldii* in the short, stout, glabrous, usually 2-seeded capsule, white flowers in a spicate inflorescence and small, somewhat glaucous leaves but differing in the well-developed axillary spikes up to 7 cm long, (not spikes essentially terminal), much smaller corolla c. 10 mm long (not 22–25 mm), the bracts all c. 7 mm long, distinct from leaves (not foliose below) and very small, near glabrous calyx < 2.5 mm long (not 4–5 mm long).

##### Description.

Erect subshrub 30–50 cm; stems somewhat woody, branched, quadrangular, nearly glabrous, obscurely bifariously scurfy. Leaves shortly petiolate, lamina (1–) 2–5.5 (–7.5) × (0.2–) 0.4–1 cm, diminishing in size upwards oblong, tapered to an obtuse apex, base cuneate, glabrous except for a few hairs towards the base, glaucous, cystoliths prominent adaxially; pet**i**oles 2–4 mm. Inflorescence of shortly pedunculate, terminal and axillary spikes, 2–7 cm long, glabrous, flowers in opposite pairs with distinct internodes; peduncles 0–10 mm; bracts 6–7 × 1.25 mm, oblanceolate, attenuate basally; bracteoles 4–5 × 0.75 mm, linear, acute; calyx subequally 5-lobed, lobes 2–2.5 × 0.5 mm, linear-lanceolate, apiculate, glabrous but puberulent apically; corolla 9–11 mm long, white with violet “herring bone” patterning, glabrous except a few hairs on lips, tube 4 mm long, upper lip 4–5 mm long, notched, lower lip 5–6 mm long, 3-lobed, the lobes ovate, rounded, c. 2 × 2 mm; anther thecae oblong, c. 1 × 0.5 mm, oblong, glabrous, weakly superposed, maroon; pollen sub, 42 × 25 μm, 3-aperturate, colporate, 1 row of c. 6–8 distinct insulae on either side of aperture (Fig. 50D); style 7.5 mm, glabrous; ovary glabrous. Capsule 7 × 3 mm, obovoid-clavate, apiculate, glabrous; 2(–4)-seeded; seeds c. 2.5 mm diam., smooth.

##### Illustration.

Fig. 23.

**Figure 23.**
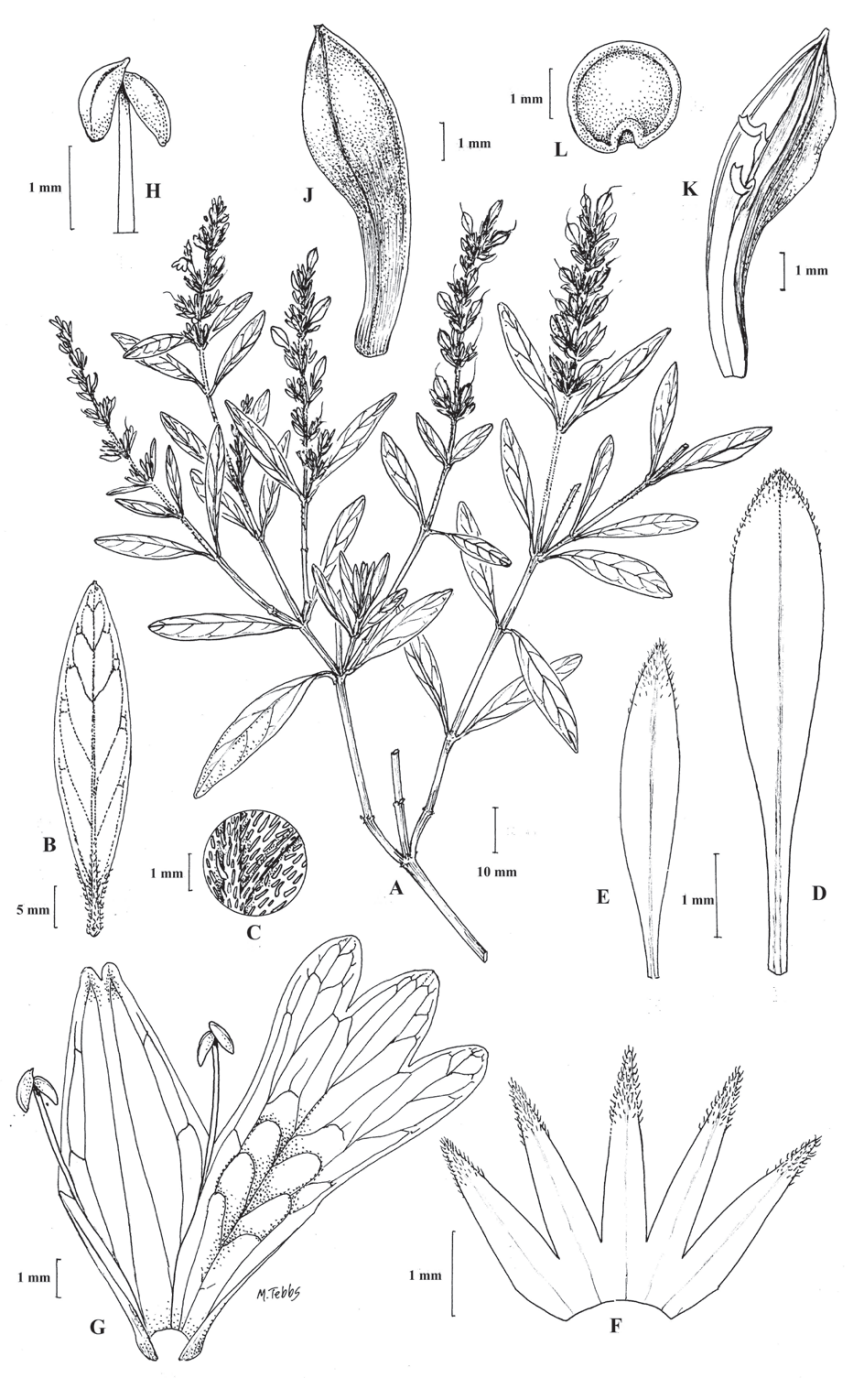
*Justiciabaguensis***A** habit **B** leaf **C** detail showing cystoliths on adaxial surface of leaf **D** bract **E** bracteole **F** calyx **G** corolla opened out to show stamens **H** anther thecae **J** exterior of capsule valve **K** interior of capsule valve **L** seed. **A, H** drawn from *Van der Werff* 14633, **J, L** from *Van der Werff* 1460 by Margaret Tebbs.

##### Etymology.

This species is named *Justiciabaguensis* as it grows mostly in Bagua Province in Amazonas region in Peru.

##### Phenology.

Flowering mainly from January to May and sporadically later.

##### Habitat.

Xerophytic, deciduous scrub/woodland with cacti at 350–600 m approximately.

##### Distribution.

Endemic to the Río Marañón valley system and restricted to Amazonas and neighbouring parts of northern Cajamarca in the north of Peru. Fig. 61.

##### Material examined.

**Peru** • **Amazonas**: Prov. Bagua, Bagua Chica to Limonyacu [5°33'S, 78°29'W], 400–450 m, 27 June 1959, *R. Ferreyra* 13646 (USM); • ibid., 10 km from Bagua Grande, 24 May 1990, *F. Borchsenius* 2617 (USM); • ibid., Bagua Chica a Bagua Grande, 700 m, 7 Aug. 1978, *J. Sánchez Vega et al.* 2288 (CPUN); • ibid., Bagua Grande–Pedro Ruiz road, 500–1000 m, 10 March 1998, *H. van der Werff* 14633 (MO, US, USM); • ibid., the type, *H. van der Werff* 14604 (F, MO, MOL, USM); • ibid. *H. van der Werff* 14611 (F, MO, MOL, USM, US); • ibid., 2 km W of Bagua Chica [5°38'S, 78°31'W], 550 m, 19 Jan. 1964, *P.C. Hutchison & J.K. Wright* 3631 (F, K, MO, US). Prov. Utcubamba Dist. El Milagro, El Valor, 5°38'S, 78°40'W, 350 m, 3 Feb. 1999, *R. Vásquez* 25904 (HUT, MO, US). • **Cajamarca**: Prov. San Ignacio, Dist. Huarango, Puerto Ciruelo–camino a Huarango, 5°17'S, 78°46'W, 550–650 m, 26 April 1996, *J. Campos & P. Diaz* 2675 (F, HUT, MO, US, USM); • ibid., Dist. Tabaconas, Tamborapa, Las Juntas, 5°22'34"S, 78°46'51"W, 600 m., 9 Dec. 2001, *R. Vásquez et al*. 27235 (HUT, MO, USM). Prov. Jaén, cerca de Jaén [5°42'S, 78°48'W], 650–700 m, April 1950, *H. Augusto* 11 (USM).

##### Note.

The capsules usually contain only two seeds, but this is not constant; some specimens develop 3 or 4 apparently viable seeds.

#### ﻿﻿26. Justicia cajamarcensis

Taxon classificationPlantaeLamialesAcanthaceae

R.Villanueva & J.R.I.Wood sp. nov.

E9763C15-FA92-53DE-98EA-34C668972ED9

urn:lsid:ipni.org:names:77363414-1

##### Type.

**Peru** • Cajamarca: Prov. San Ignacio, Dist. Huarango, Entre Puerto Tabalozo and Nueva Esperanza, 5°21'S, 78°44'W, 550–700, 18 Jan. 1996, *J. Campos & O. Diaz* 2005 (holotype US-3387858, isotypes F-2235744, MO-5297090, USM).

##### Diagnosis.

Resembling *Justiciareginaldii* and *Justiciabaguensis* in the short, stout, glabrous 2-seeded capsule and white flowers but differing in the densely imbricate (not lax and somewhat distant) flowers of the axillary spikes; from the former by the smaller corolla and from the latter by the relatively broad, oblong-ovate leaves (1–3.5 cm long), and the larger bracts, 6–9 mm long (not < 7 mm) and calyx lobes 4–5 mm long (not 2–2.5 mm long).

##### Description.

Subshrub 0.5–1.3 (–2.8) m in height; stems grey-green, bifariously pubescent when young, soon glabrescent. Leaves petiolate, lamina 3–10 × 1–3.5 cm, broadly to narrowly oblong-ovate, shortly acuminate to a very obtuse apex, base narrowly to broadly cuneate, margin undulate, both surfaces with numerous very small cystoliths, glabrous, adaxially dark green, abaxially paler, lateral veins 3–4 pairs; petioles 4–9 mm, puberulent, glabrescent. Inflorescence of shortly pedunculate axillary and terminal spikes 1–6 cm long; flowers imbricate; peduncles 3–20 mm, shortly pubescent, sometimes with gland-tipped hairs; bracts sessile, 6–9 × 1.3–2 mm, diminishing in size upwards, narrowly oblong-elliptic to oblanceolate, acute, ciliate; bracteoles linear 4–6 × 0.5–1 mm, ciliate; calyx subequally 5-lobed to 1.5 mm above base, lobes 4–5 × 1.25 mm, narrowly oblong-elliptic, acute to cuspidate, ciliolate; corolla 15 mm long, white, pubescent, basal cylindrical tube very short, c. 2 × 2 mm, upper lip hooded, notched, c. 12 mm long, lower lip 3-lobed, lobes narrowly ovate, 4–5 × 3 mm, rounded; filaments glabrous, anther thecae oblong with short basal appendage, 1.5 × 0.5 mm, parallel, superposed, glabrous; pollen subprolate, 39 × 35 μm, 3-aperturate, porate, grain covered in verrucae (Fig. 50E); ovary and style glabrous. Capsule 8–9 × 2.75–3 mm, obovoid with broad sterile base, apiculate, glabrous, 2–4-seeded; seeds c. 3 mm diam., brown, smooth.

##### Illustration.

Fig. 24.

**Figure 24.**
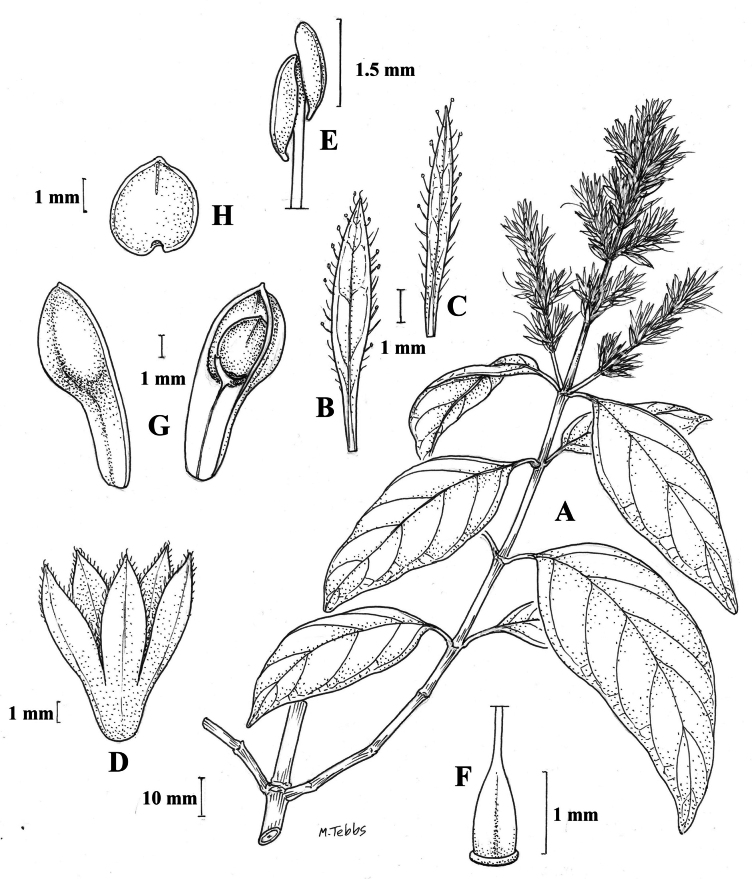
*Justiciacajamarcensis***A** habit **B** bract **C** bracteole **D** calyx **E** anther **F** ovary **G** capsule, exterior (left) and interior (right) **H** seed. Drawn from *Campos & Diaz* 2005 by Margaret Tebbs.

##### Etymology.

This species is named after the region of Cajamarca, to which it is endemic.

##### Phenology.

Flowering from December (to April).

##### Habitat.

Xerophytic scrub/woodland in northern Cajamarca, 550–900 m.

##### Distribution.

Endemic to Cajamarca in northern Peru. Fig. 61.

##### Material examined.

**Peru** • **Cajamarca**: Prov. San Ignacio, Dist. Huarango, entre Puerto Tabalozo and Nueva Esperanza, 5°21'S, 78°44'W, 550–700, 18 Jan. 1996, *J. Campos & O. Diaz* 2005 (F, MO, US, USM); • ibid., Dist. San Ignacio, entre Puerto Huaquillo y Casa Quemada, 5°15'S, 78°50'W, 600–800 m, 29 Jan. 1976, *J. Campos & O. Diaz* 2262 (F, HUT, MO, MOL, US, USM); • ibid., Dist. Chirinos, Las Juntas, 5°20'30"S, 78°46'00"W, 600–650 m, 6 Jan. 1997, *J. Campos & O. Diaz* 3263 (F, MO, MOL, USM). Prov. Jaén, Dist. Jaén sector El Huito, 5°41'17"S, 78°48'59"W, 780 m, 22 Dec. 2012, *J.L. Marcelo Peña & R. Gutiérrez* 2253 (MOL); • ibid., Dist. Pucará [6°02'S, 79°07'W], 900 m, 12 April 1960, *F. Woytkowski* 5671 (US).

##### Note.

No open corollas were present on the specimens available to us.

#### ﻿﻿27. Justicia sagasteguii

Taxon classificationPlantaeLamialesAcanthaceae

J.R.I.Wood & R.Villanueva sp. nov.

0101950A-F91B-57A5-8F1C-C4FD486EA563

urn:lsid:ipni.org:names:77363423-1

##### Type.

Peru • Cajamarca, Prov. Contumazá, Dist. Contumazá, El Platanar–Planta Eléctrica por Ruta Cascas-Contumazá, 1400 m, 31 March 1994, *A. Sagástegui et al.* 15206 (holotype US-3291670, isotypes F-2138254, HUT).

##### Diagnosis.

Superficially resembling *J.reginaldii* and similar white-flowered, winter-flowering species from xerophytic habitats but distinctive because the axillary spikes are reduced to dense flower clusters, from which radiate distinct, elongate, linear-oblanceolate bracts up to 18 mm in length. The capsule is 4-seeded.

##### Description.

Isophyllous branched subshrub of uncertain height; stems woody below, ± terete, bifariously scurfy, glabrescent when mature. Leaves shortly petiolate, lamina 1.5–11 × 0.5–5 cm, ovate to broadly oblong-ovate, less commonly oblong-lanceolate, base cuneate, margin undulate, both surfaces glabrous, abaxially paler and with numerous cystoliths; lateral veins 5–6 pairs; petioles 0.4–1 cm, glabrous to obscurely bifariously puberulent. Inflorescence of very shortly pedunculate axillary spikes, commonly reduced to axillary clusters, 1–3 cm long; peduncles 0–4 mm bifariously puberulent; bracts at base of spike foliose, narrowly oblong, subglabrous, but with some apical pubescens, mostly 10–16 × 3–4 mm; floral bracts 8–18 × 1.5–2.5 mm, narrowly oblong, finely acuminate, narrowed to a long petiole-like base, subglabrous; bracteoles 4–12 × 0.5–1 mm, linear, finely acuminate, glabrous to thinly (glandular-) puberulent; calyx subequally 5-lobed to base, lobes 4–5 × 1 mm, oblong-lanceolate, acute, puberulent; corolla c. 1.5–2.2 cm long, pale lilac to white, densely pubescent, 2-lipped, tube c. 10 mm long, slightly widened upwards, upper lip erect, shallowly bilobed, lower lip deeply 3-lobed, the central lobe broadly ovate, rounded 8 × 7 mm, the laterals oblong, 7 × 2.5 mm rounded, filaments glabrous, anthers included in upper lip, thecae 1.75 × 0.7 mm, oblong, weakly superposed, glabrous, the lower with a short basal appendage; pollen subprolate, ?2/3-aperturate, porate, grain covered in verrucae; style pubescent basally, glabrous above, 15 mm long. Capsule 8–11 × 3.5–4 mm, strongly clavate, glabrous; 4-seeded; seeds 2.5–3 mm diam., rounded, flattened, smooth.

##### Illustration.

Fig. 25.

**Figure 25.**
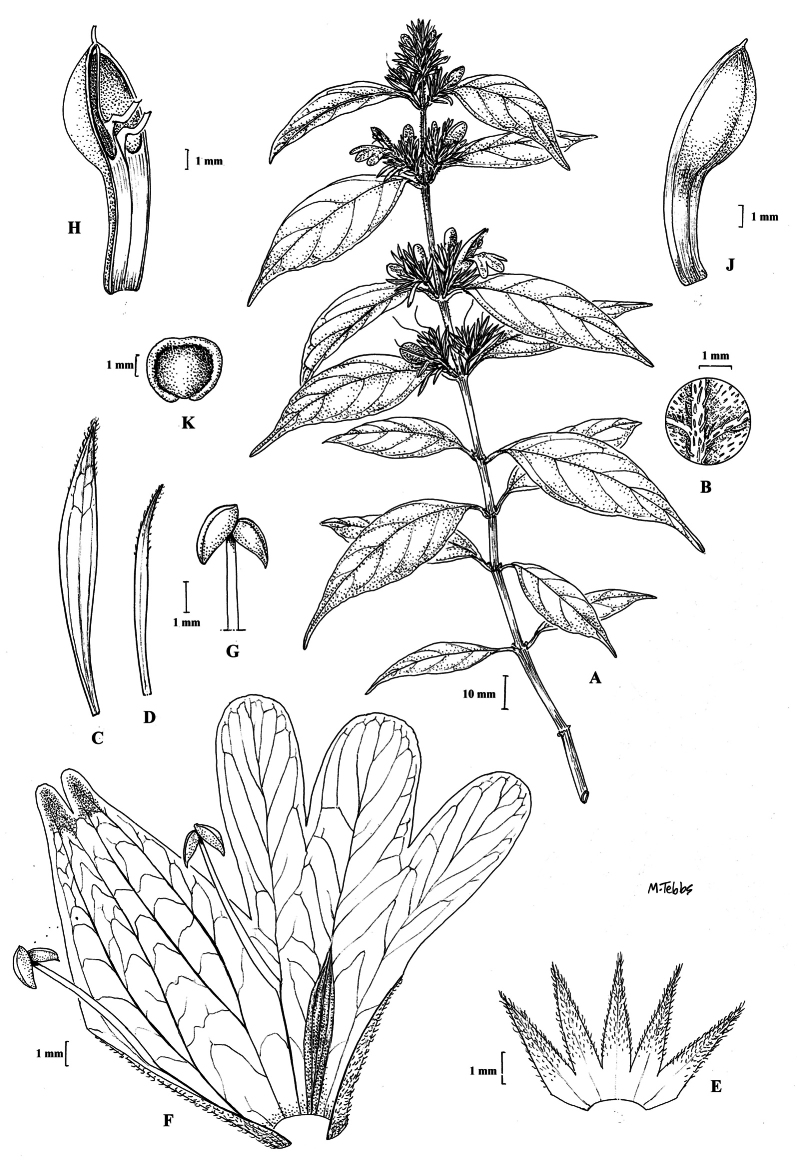
*Justiciasagasteguii*. **A** habit **B** detail of abaxial surface of leaf **C** bract **D** bracteole **E** calyx **F** corolla **G** anther **H** exterior of capsule valve **J** interior of capsule valve **K** seed. **A, G** drawn from *Sagástegui* 15206 **H, K** from *Sagástegui* 14539 by Margaret Tebbs.

##### Etymology.

This species is named for Abundio Sagástegui, collector of all cited specimens of this species.

##### Phenology.

Flowering in March and April.

##### Habitat.

Scrubby slopes and roadsides 1400–1450 m.

##### Distribution.

Endemic to Peru occurring in the Corlás area on the La Libertad/Cajamarca border area. Fig. 61.

##### Material examined.

**Peru** • **Cajamarca**: Contumazá, the type. • **La Libertad**: Prov. Gran Chimú, Corlás, (Cascas-Contumazá), 7°26'7"S, 78°48'W, 1450 m, 26 April 2002, *Sagástegui et al.* 16879 (F); • ibid., Corlás, arriba de Cascas [7°28'S, 78°49'W], 1450 m, 16 April 1992, *Sagástegui et al.* 14539 (F, US).

##### Note.

The long, thin bracts and bracteoles give the inflorescence a “whiskery” appearance. Occurs at higher altitudes than similar species.

﻿Species 28–31. These species have a branched terminal inflorescence forming a panicle of spikes, the calyx 4–5-lobed but are probably heterogenous genetically.

#### ﻿﻿28. Justica dryadum

Taxon classificationPlantaeLamialesAcanthaceae

Wassh. & J.R.I.Wood, Kew Bull. 58(4): 813. 2003. (Wasshausen and Wood 2003: 813)

ABE51221-19B1-5960-81FA-9EB350583960

##### Type.

Bolivia • La Paz, Sud Yungas, P.N. Pilon Lajas, *D.C. Wasshausen & J.R.I. Wood* 2196 (holotype US-00731139[Cat. No 3455450], isotypes K-001256339, LPB-0000410, US-02878814).

##### Description.

Perennial herb to 1 m in height, stems bifariously pilose. Leaves petiolate, lamina (2–)6–16 × (1–)2–8.5 cm, oblong-elliptic or obovate-oblong, acuminate, falcate, attenuate at the base, pilose on both surfaces, cystoliths prominent above; petioles 0.3–2 cm long. Inflorescence a large terminal panicle up to 25 × 20 cm; flowers arranged in pairs; branches repeatedly 3-forked, glandular-pilose; floral bracts 1–2.5 mm long, linear-lanceolate, shortly glandular-pilose; calyx c. 3 mm long, subequally 5-lobed to near the base, lobes linear-lanceolate, glandular; corolla c. 11 mm long, glabrous except for a few hairs on the exterior of the lips, tube pale cream c. 1.5 mm wide at base, upper lip c. 3.5 mm long, entire, lower lip c. 4 mm long, 3-lobed, the lobes c. 1.5 × 1 mm, ovate, rounded, purplish except for distinct “herring bone” patterning; anther thecae weakly superposed, violet, c. 0.5 mm long; pollen 18 × 13 μm, 2-aperturate (Wasshausen and Wood 2003: 803). Capsule 8.5–10 × 2–2.5 mm, oblong-clavate, minutely glandular-pilose; seeds c. 2 × 2 mm, ovate, lenticular, minutely pustulate.

##### Illustration.

Wasshausen and Wood 2003: 803 (pollen), 814 (habit and floral details).

##### Phenology.

Flowering in the winter dry season, principally from July to October.

##### Habitat.

Moist lowland rainforest, 200–500 m.

##### Distribution.

Northern Bolivia and southern Peru. New record for Peru. Fig. 62.

##### Material examined.

**Peru** • **Cusco**: Prov. Paucartambo, Dist. Kosñipata, near junction Río Carbon with Río Alto Madre de Dios, 6–7 Aug. 1974, *H. Brokaw, M. Brockaw & R. Foster et al.* 3045 (F). • **Madre de Dios**: Prov. Manu, P.N. del Manu, Coca Cashu Station, 350 m, 11°50'S, 71°25'W, 31 Aug. 1984, *R. Foster* 9936 (F, MO, USM); • ibid., Río Palotoa, NW of Shintuya, 500 m, 26–28 Aug. 1978, *R. Foster & J. Terborgh* 6734 (F); • ibid., zona reservada del P.N. del Manu, 350 m, 4–22 Sept. 1989, *A. Tupayachi* 1201 (CUZ). Prov. Tambopata, Dist. Las Piedras, Cusco Amazónico, Inventario Permanente, Trocha B, 12°29'S, 69°03'W, 200 m, Aug. 1991, *M. Timaná* 1991 (MO); • ibid., 10 Oct. 1991, *M. Timaná & N. Jaramillo* 2532 (MO).

##### Note.

Close to *Justiciatenuiflora* Ruiz & Pav., differing in the pubescent leaves which lack the long-attenuate leaf base of that species, by the presence of warty outgrowths on the lower part of the stem, and the shorter, lilac, not red corollas.

#### ﻿﻿29. Justicia lineolata

Taxon classificationPlantaeLamialesAcanthaceae

Ruiz & Pav., Fl. Peruv. Prodr.1: 9. 1798. (Ruiz and Pavón 1798: 9)

AFC63CD9-5702-5F20-906D-B7F5CE3E8730

Rhytiglossa lineolata (Ruiz & Pav.) Nees, Prodr. [A. P. de Candolle] 11: 341.1847. (Nees 1847b: 341)
Ecbolium lineolatum (Ruiz & Pav.) Kuntze, Revis. Gen. Pl. 2: 980. 1891. (Kuntze 1891: 980)
Justicia flavidiflora Lindau, Bull. Herb. Boissier, sér. 2, 4: 409. 1904. (Lindau 1904: 409). Type. Peru. Amazonia, Juruá Miry, *E. Ule* 5699 (presumed holotype B†, photo of holotype F0BN008828, lectotype HBG-0522729, designated here, isolectotypes CORD-00005109, G-00236288, K-000529332, MG-005628, US-02879664, fragment).

##### Type.

Peru • [Huánuco], Cochero, *Ruiz & Pavon* s.n., (lectotype MA-815525, designated here, isolectotypes BM, MA-815526, MA-815527, MPU-018337).

##### Description.

Herb 30–100 cm high; stems striate, glaucous, thinly but coarsely bifariously hirsute. Leaves shortly petiolate, lamina 7–18 × 3–10 cm, oblong-elliptic, elliptic or obovate, apex obtuse, base broadly to narrowly cuneate; coriaceous in texture, olive-green in colour, cystoliths numerous, glabrous; petioles 3–15 mm. Inflorescence a long-pedunculate terminal panicle of spikes; the branches 0–12 cm long, sometimes reduced to a cluster of flowers; peduncles 5–22 cm, pubescent with crisped white hairs; rhachis white-pubescent with some gland-tipped hairs; flowers sessile or borne on short pedicels c. 1 mm long; bracts subulate, 1.5 mm long, hispid; bracteoles similar; calyx 5-lobed, lobes lanceolate, 3 × 0.5 mm, hispid and with scattered glands; corolla 8–9 mm long, cream, puberulent, gaping, upper lip entire, lower lip with purple “herring bone” patterning, shallowly 3-lobed; thecae 1 × 0.5 mm, ellipsoid, weakly superposed, lacking basal appendages.Capsule 10 × 2 mm, clavate, glabrous, 4-seeded.

##### Illustration.

Figs 26, 27.

**Figure 26.**
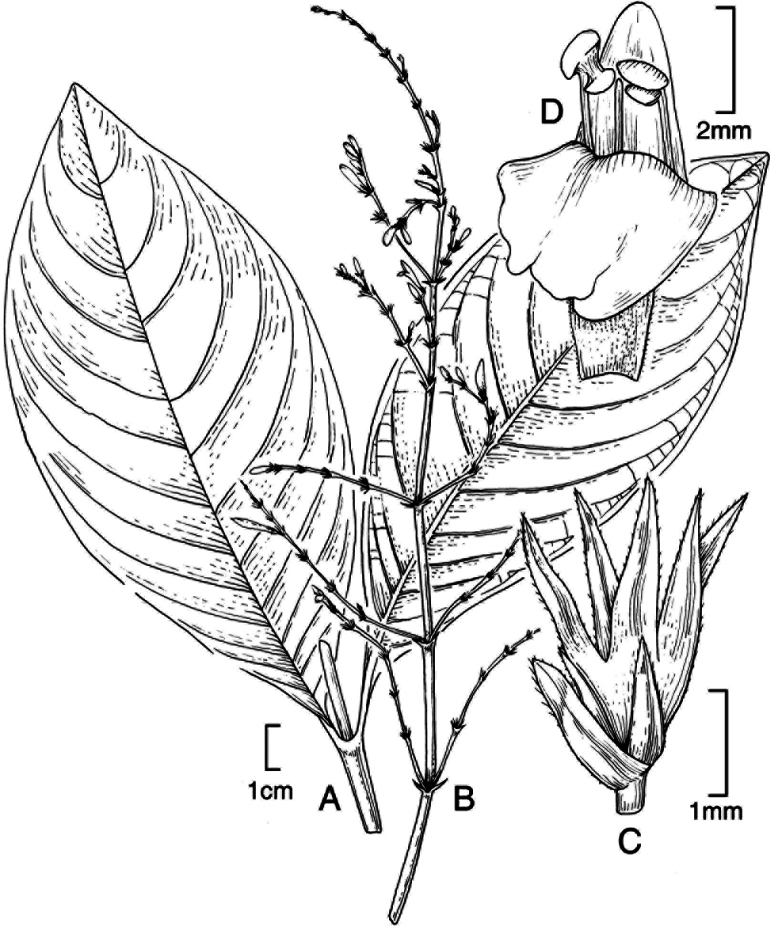
*Justicialineolata***A** leaves **B** inflorescence **C** bracts, bracteoles and calyx **D** corolla opened to show stamens. Drawn from *Wasshausen & Encarnación* 588 by Cathy Pasquale.

**Figure 27.**
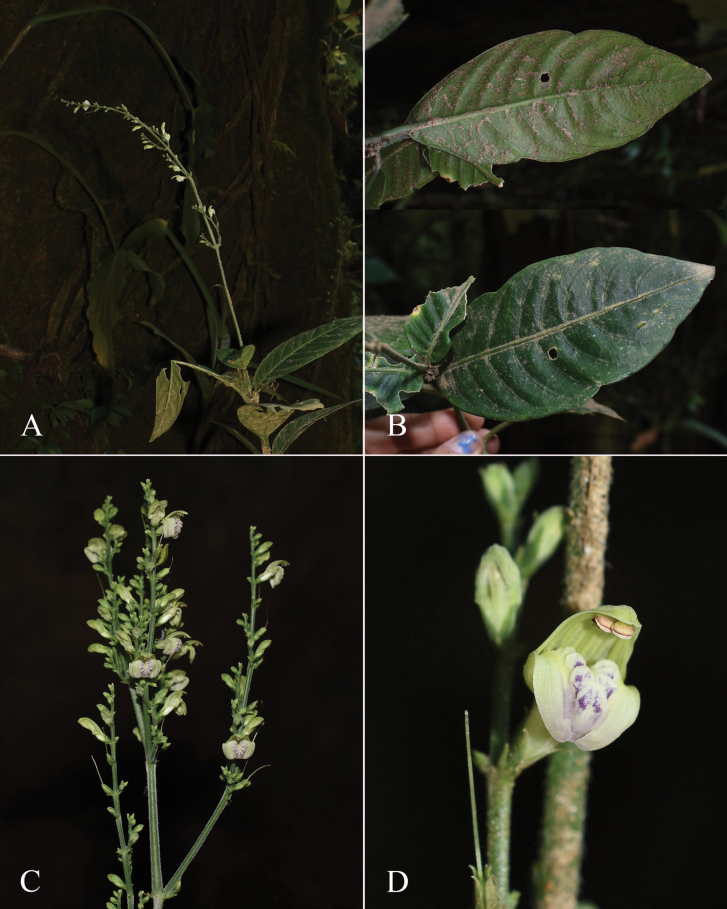
Photographs of *Justicialineolata* by Rosa Villanueva.

##### Phenology.

Flowers principally between June and October.

##### Habitat.

Primary and secondary lowland rainforest up to 960 m, often on flood plains.

##### Distribution.

Peru and Bolivia in the Amazonian lowlands and lower eastern Andean slopes from Loreto south to Amazonian Bolivia. Fig. 63.

##### Material examined.

**Peru** • Sine loc, *A. Mathews* 1838 (K). • **Ayacucho**: Prov. La Mar, along Río Catute, 2 km NW of Santa Rosa, 680 m, 3 June 1975, *D.C. Wasshausen & F. Encarnación* 509 (K, US); • ibid., along Río Catute, 2 km NW of Santa Rosa, 680 m, 8 Sept. 1976, *D.C. Wasshausen & F. Encarnación* 617 (K, MO, USM). **Cusco**: Prov. Camisea; Campamento Malvinas, 11°52'12"S, 72°56'28"W, 450 m, 23 Sept. 1997, *P. Acevedo-Rodríguez* 9889 (US, USM). Prov. La Convención, along Río Quimiri, 4 km E of San Francisco de Apurimac, 750 m, 6 June 1975, *D.C. Wasshausen & F. Encarnación* 516 (US); • ibid., Dist. Echarate, Armihuari well site, 11°51.88'S, 72°46.69'W, 535 m, 14 May 1997, *P. Nuñez V*. 20089 (US); • ibid., Armihuari–Río Camisea, 11°55'55.1"S, 72°46'38.9"W, 535 m, 11 Oct. 1998, *P. Nuñez et al*. 24253 (US, USM); • ibid., Cashiriari-3 well site, 5 km south of Camisea river, 11°52'57.1"S, 72°39'6.1"W, 700 m, 2 Oct. 1998, *P. Nuñez et al.* 23774 (US, USM); • ibid., 11°53'S, 72°39'W, 700 m, 2 Sept. 1998, *P. Nuñez et al.* 23837 (CUZ); • ibid., San Martin 3 site well, 11°46.89'S, 72°42.10'W, 400 m, 2 Nov. 1998, *H. Beltrán et al*. 3276 (US, USM) Prov. Paucartambo, Dist. Kosñipata, along lumber trail N of Pilcopata, 580 m, 27 June 1975, *D.C. Wasshausen & F. Encarnación* 588 (K, US). Prov. Quispicanchis, 3 km E of Quincemil, 960 m, 7 Oct. 1976, *D.C. Wasshausen & F. Encarnación* 727 (K, USM). • **Huánuco**: Prov. Huamalíes, Cueva de las Lechuzas, Río Monzón, 700–800 m, 18 July 1948, *R. Ferreyra* 4255 (K, MOL, US, USM). Prov. Leoncio Prado, Dist. Rupa-Rupa, Tingo María, 600–700 m, 15 Aug. 1943, *C.A. Ridoutt* s.n. (US, USM13141); ibid, Dist. Jose Crespo y Castillo, Moena, 600 m, 24 March 1954, *F. Woytkowski* 1195 (US, USM). Prov. Puerto Inca [Pachitea], Puerto Inca, 2–5 km E of town, 9°18'S, 74°57'W, 250–300 m, 11 Sept. 1982, *R. Foster* 8698 (USM). • **Junín**: Prov. Paucartambo, Dist. Perené, Río Paucartambo Valley, near Perené Bridge, 700 m, 19 June 1929, *E.P. Killip & A.C. Smith* 25288 (US); • ibid., *E.P. Killip & A.C. Smith* 25263 (US); Prov. Satipo [Chanchamayo], 550 m, 15 July 1982, *O. Tovar* 9373 (USM); • ibid., Aug. 1940, *C.A. Ridoutt* s.n. (US, USM11660). • **Madre de Dios**: Prov. Manu, Sunset Point Trail, Explorer’s Inn, near the confluence of Río Tambopata and Río La Torre, 39 km SW. of Puerto Maldonado, 12°50'S, 69°20'W, 19 Sept. 1984, *S.F. Smith* et al. 117 (US); • ibid., 12 July 1987, *S.F. Smith* 951 (US); • ibid., Río Manu: Cocha Cashu Station, 350 m, July 1978, *J. Terborgh* 6500 (F). Prov. Tambopata, Zona Reservada Tambopata, 13 Sept. 1986, *O. Phillips & P. Willein* T17 (USM); • ibid., Dist. Las Piedras, Cuzco Amazónico, 12°29'S, 69°03'W, 3 Sept. 1991, *M. Timaná & A. Rubio* 2178 (CUZ). **Pasco**: Prov. Oxapampa, La Merced–Oxapampa km 37, 800 m, 27 Aug. 2002, *A.N. Schmidt-Lebuhn* 546 (USM). • **San Martín**: Prov. San Martín: Prov. Tocache [Mariscal Cáceres], Dist. Tocache [Nuevo], Quebrada de Huaquisha (margen derecha del Río Huallaga), 17 May 1987, *J. Schunke Vigo* 3987 (F, US); • ibid., entre la Merced y Huánuco, Valle del Huallaga, 500–600 m, 2 Aug. 1948, *R. Ferreyra* 4376 (US, USM); • ibid., Vicinity of Tocache, 400–700 m, 1979, *J. Schunke* 11001 (F, MO, US); • ibid., Dist. Uchiza, Cachiyacu de Lepuna, 11 July 1974, *J. Schunke* 7306 (F, US); • ibid., Quebrada de Cañuto, 500 m, 8 Aug. 12092, *J. Schunke* 12092 (US). • **Ucayali**: Prov. Atalaya, Dist. Raimondi, Atalaya, near junction of Río Carbon with Río Alto Madre de Dios, 27 Aug. 1973, *R. Foster* 2721 (F). Prov. Coronel Portillo, Dist. Calleria, Pucallpa, 200 m, 4 Aug. 1946, *J. Soukup* 3042 (F); • ibid., Bosque Nacional Alexander von Humboldt, km 86 carretera a Pucallpa, 250–300 m, 30 July 1978, *J. Schunke Vigo*10416 (MO). Prov. Purús, Dist. Purús. Río Curanja, cerca la comunidad nativa colombiana, 10°04'S, 71°06'W, 300–350 m, 13 July 2002, *J. G. Graham & J. Schunke Vigo* 1721 (US); • ibid., cerca del pozo grande entre las comunidades de Balta y Columbiana, 10°04.121'S, 71°08.555'W, 325 m, 24 July 1998, *J. Graham* 659 (F); • ibid., Centro Poblado Balta, *K.M. Kensinger* 79 (USM).

##### Lectotypifications.

In choosing a lectotype for *Justicialineolata* Ruiz & Pav. we have simply chosen the most complete specimen at MA. Lindau cited two specimens of *Justiciaflavidiflora*, *Ule* 5699 and *Poeppig* 1813, both stored presumably at Berlin and both destroyed in 1943. *Poeppig* 1813 was collected at “Maynas prope Tocache”, so presumably from San Martin in Peru. We have been unable to trace any duplicate or image of this specimen, so have selected the Hamburg collection of *Ule* 5699 as the lectotype, this specimen already annotated (but unpublished) as lectotype by Wasshausen.

##### Notes.

This species is very well named. The cystoliths are numerous giving the plant a bluish-green appearance when dry, often facilitating the identification of poor or even sterile material.

#### ﻿﻿30. Justicia tenuiflora

Taxon classificationPlantaeLamialesAcanthaceae

Ruiz & Pav., Fl. Peruv. Prodr.1: 9. 1798. (Ruiz and Pavón 1798: 9)

69C2B830-0A18-56E2-99AB-639B973C3F09

Rhytiglossa tenuiflora (Ruiz & Pav.) Nees, Prodr. [A. P. de Candolle] 11: 340. (Nees 1847b: 340).
Ecbolium tenuiflorum (Ruiz & Pav.) Kuntze, Revis. Gen. Pl. 2: 981. 1891. (Kuntze 1891: 981).
Jacobinia tenuistachys Rusby, Mem. Torrey Bot. Club 6(1): 105. 1896. (Rusby 1896: 105) Type. BOLIVIA. La Paz, [Larecaja], Guanay–Tipuani, *M. Bang* 1441 (holotype NY-00312097, isotypes BM-000617658, BM-000617659, C-10004935, CAS-0003066, CM-2079, CORD-00005098, E, F-0047399F, GH-00094054, GH-00249355, K-000529470, LD-1688024, M-0186467, M-0186468, MICH-1104048, MIN-1001467, MO-1587290, NY-00038826, PH-00016185, PH-00016186, PH-00016187, PUL-00000304, S-05-386, US-01095038, US-01095039), W1893-0005428, W-1893-0005430).
Justicia tenuistachys (Rusby) Wassh. & J.R.I.Wood, Kew Bull. 58(4): 818. 2003. (Wasshausen and Wood 2003: 818), syn. nov.
Beloperone baenitzii H. Winkl., Repert. Spec. Nov. Regni Veg. 7: 113. 1909 (Winkler 1909: 113) Type. BOLIVIA, Larecaja, Charopampa bei Mapiri, *Buchtien* 1409 (presumed holotype B†, photo of holotype F0BN008922, isotype US-00137217).
Justicia baenitzii (H. Winkl.) C. Ezcurra, Bol. Soc. Argent. Bot. 25: 348. 1988. (Ezcurra 1988: 348).
Beloperone viridissima Rusby, Mem. New York Bot. Garden 7: 367. 1927. (Rusby 1927: 367). Type. BOLIVIA. Vic. Huachi, head of Beni River, 1800 ft, 13 Aug. 2021, *C.E.White* 550 (holotype NY-00311814, isotypes BKL-00000005, GH-00093742, K-000529469, MICH-1104018, NY-00311815, US-00137240).

##### Type.

Peru • [Huánuco], Cuchero [San Juan de Cochero], [9°30'S, 75°51'W, 1886 m], *Ruiz & Pavon* s.n. (lectotype MA815548, designated here, isolectotype MA815547).

##### Description.

Perennial subshrub to 80 cm in height. Leaves shortly petiolate, lamina mostly 8–18 × 4–8 cm, oblong-elliptic, acuminate, base cuneate, glabrous or nearly so. Inflorescence a terminal panicle c. 15–20 cm long, composed of lax branched spikes; bracts and bracteoles deltoid, 1–2 mm long; calyx subequally 5-lobed, subglabrous to densely, but shortly glandular hirtellous, lobes 4–6 × 0.25 mm, subulate; corolla 4–4.5 cm long, red, hirtellous, the tube 2–2.5 cm long, the lips 1.3–2 cm long; thecae narrowly ellipsoid, 2 × 0.5 mm, one distinctly smaller, weakly superposed, lacking basal appendages; pollen prolate 38–40 × 22–23 μm, 2-aperturate, colporate, 1 row of c. 6–7 poorly developed insulae on either side of the aperture (Fig. 50F). Capsule 12–13 × 2–3 mm, clavate, glandular-hirtellous; 4-seeded; seeds tuberculate.

##### Illustration.

Fig. 28.

**Figure 28.**
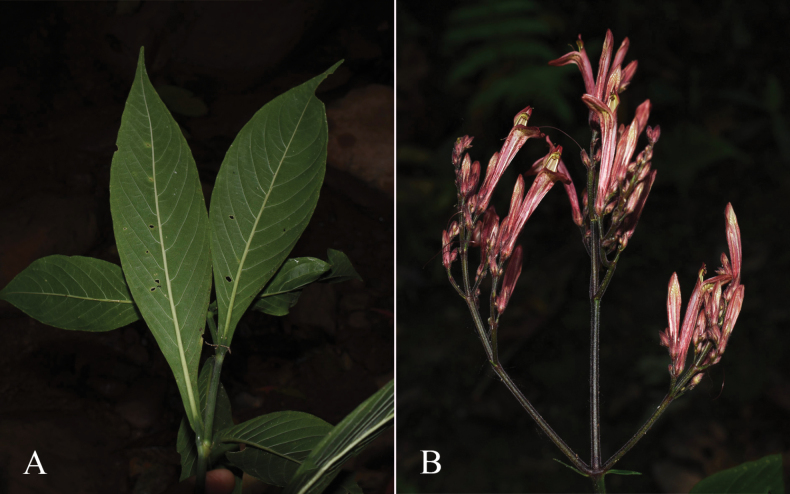
Photographs of *Justiciatenuiflora* (*Villanueva* 975) by Rosa Villanueva.

##### Phenology.

Flowering from May to October.

##### Habitat.

Tropical rainforest in the Andean foothills, very tolerant of shade, often near streams. Locally common, 600–1200 m approx.

##### Distribution.

Moist eastern slopes of the Andes from northern Argentina through Bolivia to San Martin and Loreto, but apparently much more common in Huánuco than elsewhere in Peru. Its local abundance in central Peru parallels to some extent the distribution of *Justiciawarmingii*. Fig. 62.

##### Material examined.

**Peru.** July 1854, *Lechler* 3153 (K). • **Huánuco**: [Prov. Chinchao], Pampayacu [9°33'28"S, 75°54'35"W], mouth of Río Chinchao, July 1923, *J.F. Macbride* 5102 (US). Prov. Huamalíes, Monzón, 950 m, 8 Sept. 1954, *F. Woytkowski* 1562 (MOL). Prov. Leoncio Prado, right margin of Río Monzón, near Cueva de Las Lechuzas [9°18'46.92"S, 76°00'00.71"W], 700–800 m, 18 July 1948, *R. Ferreyra* 4237 (MO, US, USM); • ibid., trail S of Puerto Cayumba [9°29'41"S, 75°57'9"W], 25 km S of Tingo María, 20 June 1982, *D.C. Wasshausen & O. Tovar* 1279 (K, US); • ibid., confluence of Monzón and Huallaga rivers, near Tingo María, 700 m, 25 Oct. 1938, *H.E. Stork & O.B. Horton* 9503 (K); • ibid., entre Tingo Maria y Monzón, 700–750 m, 20 Aug. 1967, *R. Ferreyra* 16993 (MO, US, USM); • ibid., Dist. Luyando, Hacienda Shapajilla, cerca de Tingo María, 600–700 m, 10 Aug. 1946, *R. Ferreyra* 878 (USM); • ibid., Tulumayo, near Tingo María, 650–700 m, *R. Ferreyra* 2182 (US); • ibid., Dist. Mariano Damaso, Bella Durmiente, cerca de Cueva de las Lechuzas, 700 m, 10 Oct. 1959, *R. Ferreyra* 13806 (USM); • ibid., Dist. Monzón, confluencia con el Río Huallaga, cerca Tingo María, 700 m, 23 Sept. 1954, *R. Ferreyra* 10233 (US, USM); • ibid., Dist. Rupa-Rupa, Río Bella, 7 km de Tingo María, 6 Aug. 1946, *J. Soukup* 3097 (US); • ibid., along Río Monzón on trail to Cueva de las Lechuzas, 650 m, 18 June 1982, *D.C. Wasshausen & O. Tovar* 1259 (K, MO, US); • ibid., along Río Monzón, Jacintillo [9°19'6"S, 76°0'38"W], 680–700 m, 18 July 1978, *J. Schunke Vigo* 10374 (MO, US); • ibid., Calpar Bella, cueva de los Hauriños. margen izquierda del Río Monzón, 700–900 m, 2 July 1976, *J. Schunke Vigo* 9488 (AAU, F, MO, US), *J. Schunke Vigo* 9489 (USM). Prov. Pachitea, Dist. Chaglla, rumbo a caserío Montevideo, 9°28'51"S, 75°53'13"W, 1810 m, 9 Aug. 2023, *R. Villanueva* et al. 988 (HOXA). • **Madre de dios**: Prov. Tahuamanu, cerca del Río Yaverija, a 2 km de Iñapari, 23 May 1978, *F. Encarnación* 1147 (US). • **Puno**: Prov. Carabaya, Dist. San Gabán [13°28'48"S, 70°25'12"W], 900 m, 12 June 1982, *D.C. Wasshausen & A. Salas* 1245 (K, US). Prov. Sandia, Dist. Sagrario [13°55'01.2"S, 69°40'58.8"W], 1000–1300 m, 26 May 1942, *R.D. Metcalf* 30631 p.p. (MO). • **San Martín**: Prov. Tocache [Mariscal Cáceres], Dist. Tocache Nuevo, Quebrada de Santa Rosa de Cachiyacu carretera a Progreso, [8°31'5"S, 76°25'W], 500–700 m, 19 July 1974, *J. Schunke Vigo* 7572 (MO, US, USM); • ibid., Dist. Uchiza, Cerro de Santa Cruz, al este del puente, carretera marginal, 700–800 m, 3 Aug. 1974, *J. Schunke Vigo* 8010 (F, MO, US, USM). • **Ucayali**: Prov. Coronel Portillo, Dist. Iparia, falda dentro las cuencas de los Ríos Arapo y Manegene, afluentes del Río Ucayali, 1100 m, 22 Aug. 2010, *J.G. Graham* 5952 (MOL, US, USM). Prov. Padre Abad, Dist. Padre Abad, carretera antigua a Pucallpa, 1200–1300 m, 9 May 1978, *J. Schunke Vigo* 10142 (U, US); • ibid., cumbre de la Divisoria, entre Ucayali y Huánuco, cabecera del Río Yurac, afluente del Río Aguaytía, 9°11'03"S, 75°47'47"W, 1500–1600, 7 June 2007, *J. G. Graham & J. Schunke Vigo* 4185 (MOL, US, USM); • ibid., Catarata Santa Rosa, 9°09'40"S, 75°45'39"W, 882 m, 8 Aug. 2023, *R. Villanueva et al.* 975 (HOXA); • ibid., Boquerón Padre Abad, 9°03'58"S, 75°40'45"W, 450–500 m, 5 Sept. 2019, *I. Azevedo et al.* 281 (IBSC).

##### Note.

Variable in the indumentum and length of the sepals. Sepals are narrowly lanceolate with a fine attenuate apex. In younger specimens, the sepals may be only 3–4 mm long but in more mature specimens, they can reach 7 mm. In some specimens the sepals are completely glabrous, but younger specimens are often with sessile glands, these becoming stipitate and mixed with eglandular hairs in some more mature specimens.

#### ﻿﻿31. Justicia warmingii

Taxon classificationPlantaeLamialesAcanthaceae

Hiern, Vidensk. Meddel. Naturhist. Foren. Kjøbenhavn 1877–78: 80. (Hiern 1877: 80)

68C33605-458B-5BD1-B26C-C56040FE24EA

Dianthera hirsuta Ruiz & Pav., Fl. Peruv. Prodr.1: t.13 (Ruiz and Pavón 1798: t.13) pro parte, quoad icones et spec. in Herb. Benth. (K), non descr. et non (Jacq.) J.F.Gmel. (1791).
Sarotheca elegans Nees, Flora Bras. 9(7): 113. 1847. (Nees 1847a: 113) non J.elegans P. Beauv. (1806). Type. BRAZIL. Goias, Serra de S. Felis ad fluvium Rio Trahiras, Pohl 1989 (holotype GZU-000250360, isotype W-0049983).
Justicia sarotheca V.A.W. Graham, Kew Bull. 43(4): 614. 1988. (Graham 1988: 614). Type. Based on SarothecaelegansPohl.
Sarotheca glutinosa Bremek., Proc. Kon. Ned. Akad. Wetensch., C 72: 426. 1969. (Bremekamp 1969: 426) Type. BOLIVIA. Chuquisaca, *W.M.A. Brooke* 5677 (holotype BM-000551558, isotype F-0077602F).
Justicia glutinosa (Bremek.) V.A.W. Graham, Kew Bull. 43(4): 613. 1988. (Graham 1988: 613).

##### Type.

Brazil. *Warming* 1028 (holotype C-10005029, isotype K-000529271).

##### Description.

Subshrub 0.5–2 m high, stems conspicuously pilose. Leaves petiolate, lamina 6–14 × 2–4 cm, variable in shape, commonly broadly oblong, sometimes, ovate, oblong-elliptic or oblanceolate, shortly acuminate, base attenuate and sometimes decurrent on the petiole, thinly or densely pilose on the veins, especially beneath; petioles 0.2–2.5 cm, hirsute. Inflorescence of simple or branched spikes 5–15 cm long from the upper leaf axils, these often aggregated to form a terminal panicle of spikes; rhachis and axes glandular and somewhat sticky; bracts linear-oblong, 4–5 × 1–2 mm, glandular, bracteoles similar but shorter; calyx 4-lobed to base, lobes 4–5 × 1 mm, lanceolate, glandular; corolla 9–10 (–12) mm long cream with purple “herring bone” patterning on lower lip, glandular, tube short, stout, 2.5 mm wide, lips short 4–5 mm; pollen prolate 40 × 21 μm, 2-aperturate, colporate, 1 row of indistinct insulae on either side of the aperture (Fig. 51A). Capsule 14–17 × 3 mm, clavate, constricted between seeds, covered in subsessile glands, 4-seeded; seeds 3 mm diam., tuberculate.

##### Illustration.

Figs 29, 30.

**Figure 29.**
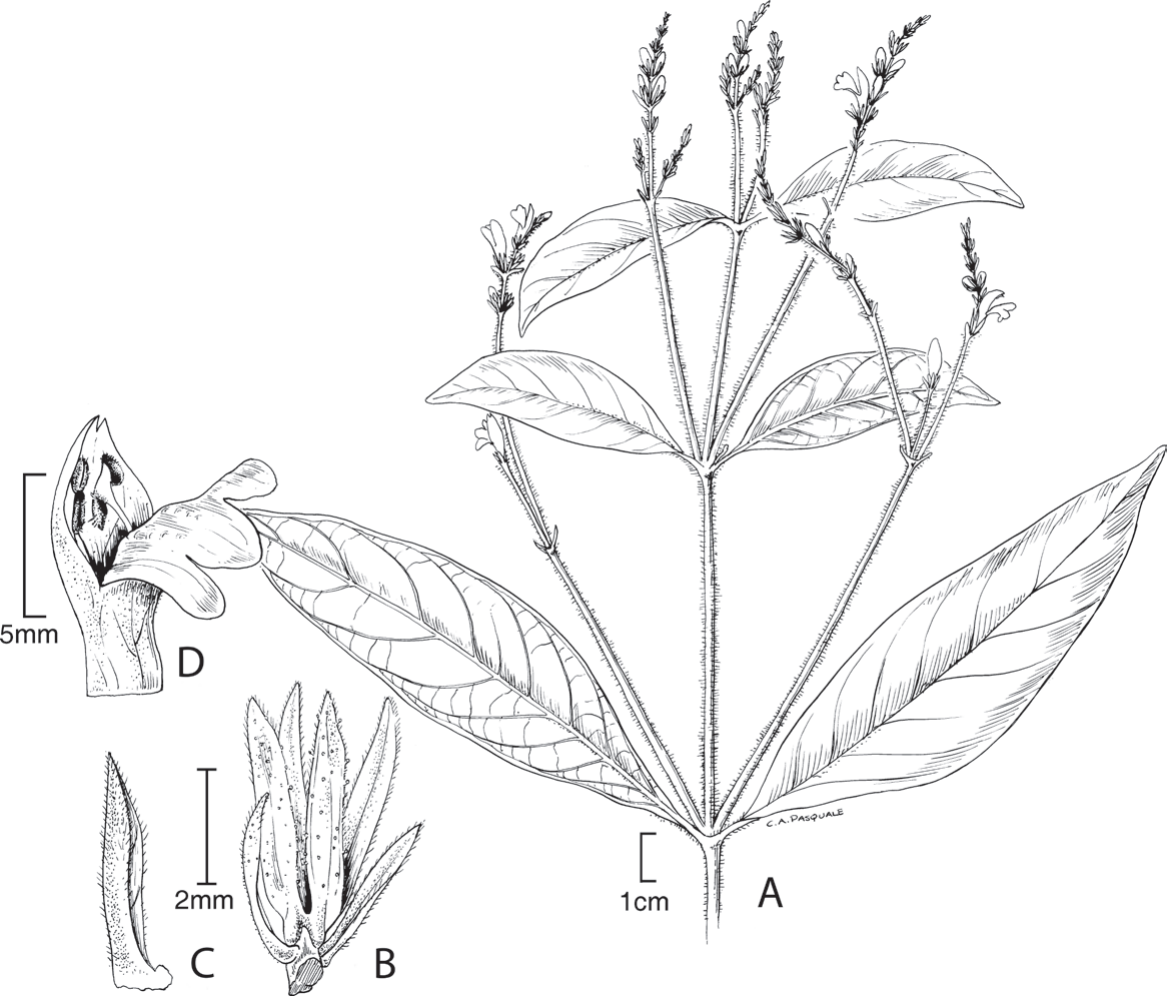
*Justiciawarmingii***A** habit **B** bract, bracteole and calyx **C** bract **D** corolla showing anthers. Drawn from *Killip & Smith* 23459 by Cathy Pasquale.

**Figure 30.**
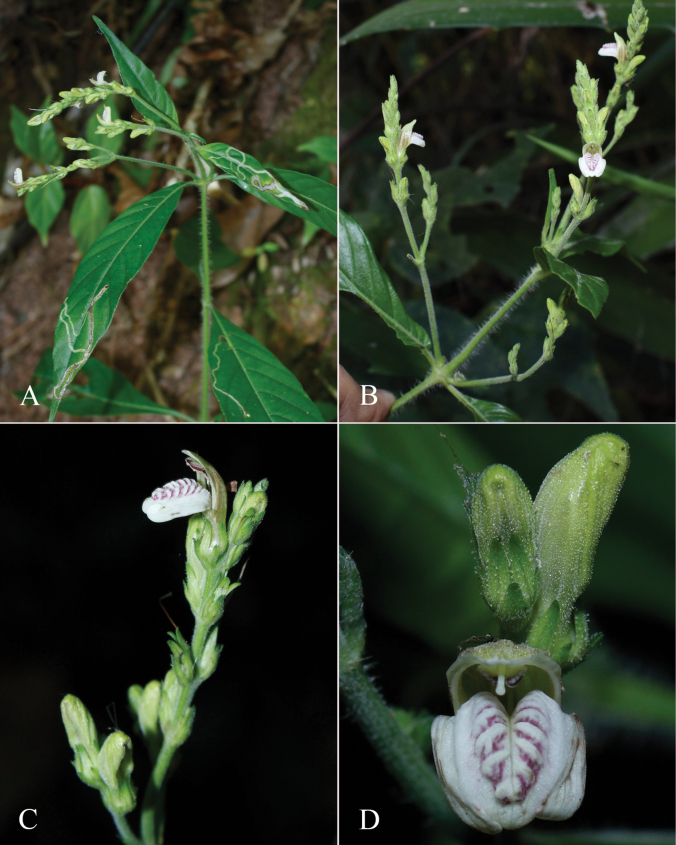
Photographs of *Justiciawarmingii* (*Villanueva* 920) by Rosa Villanueva.

##### Phenology.

Found in flower throughout most of the year, but perhaps flowering most prolifically in the June–September period.

##### Habitat.

Subtropical forest, secondary bushland, cliffs, scrubby slopes, roadsides between 500 and 1500 m.

##### Distribution.

Widely distributed in dispersed but locally extensive colonies in Peru, Argentina, Brazil and Bolivia. In Peru almost restricted to Junín in the centre of the country, where it is abundant, constituting a very curious distribution, somewhat parallelling that of *Justiciatenuiflora*, as both species are locally common further south in Bolivia. Fig. 62.

##### Material examined.

**Peru** • **Junín**: Prov. Chanchamayo, Montayaco, west of San Ramón, 900 m, 27 June 1976, *Al. Gentry & G. Prance* 16419 (F, MO, USM); • ibid., Puente Paucartambo to La Merced, Chanchamayo valley, 800 m, 30 Jan. 1983, *Al. Gentry et al.* 39826 (MO, US, USM); • ibid., Río Colorado, near junction with Río Chanchamayo, 10°58'S, 75°22'W, 500–600 m, 7 Feb. 1983, *A. Gentry &. D. Smith* 40141 (MO, US); • ibid., Pichis trail, Enenas, 1600–1900 m, June–July 1929, *E.P. Killip & A.C. Smith* 25647 (US); • ibid., San Ramón–Chanchamayo, 1360 m, 17 July 1978, *H. Ellenberg* 8880 (US); • ibid., Río Colorado, San Ramón–Puente Paucartambo road, 10°58'S, 75°30'W, 760 m, 6 Oct. 1982, *D. Smith & R. Foster* 2486 (MO, USM, US); • ibid., Chanchamayo Valley, 1200 m, July 1929, *C. Schunke* 457 (F); • ibid., *C. Schunke* 458 (F); • ibid.,Chanchamayo Valley, 1200 m, June 1929, *C. Schunke* 234 (F); • ibid., Chanchamayo Valley, 1500 m, Sept. 1924–1927, *C. Schunke* 376 (F); • ibid., San Luis, 14 Aug. 1944, *Ridoutt* s.n., USM 14558 (USM); • ibid., Dist. Chanchamayo, La Merced, 2000 ft, Aug. 1923, *J.F. MacBride* 5204 (US); • ibid.,10–24 Aug. 1923, *J. F. MacBride* 5294 (F); • ibid., 800 m, Aug. 1944, *J. Soukup* 2527 (US); • ibid., 700 m, May–June 1929, *E.P. Killip & A.C. Smith* 23454 (F); • ibid., *E.P. Killip & A.C. Smith* 23462 (US); • ibid., Río Chanchamayo, vic. La Merced, 750 m, 23 May 1979, *D.C. Wasshausen & F. Encarnación* 1080 (K, MO, US, USM); • ibid., entre Pampa Whaley y Puente Perené, 800–900 m, 23 Sept. 1955, *R. Ferreyra* 11363 (MO, US, USM); • ibid., *R. Ferreyra* 11369 (USM); • ibid., 600 m, 16–18 June 1929, *E.P. Killip & A.C. Smith* 25240 (F); • ibid., Río Perené, 19 June 1929, *E.P. Killip & A.C. Smith* 25762 (US); • ibid., Dist. Pichanaki, 750 m, 3 Sept. 1960, *G.W.H. Kunkel* 6237 (B); • ibid., Dist. Perené, Río Perené, Colonia Perené, 600 m, June 1929, *E.P. Killip & A.C. Smith* 25211 (US); • ibid., June 1929, *E.P. Killip & A.C. Smith* 24940 (US); • ibid., along Río Perené, near “Hacienda 3”, Colonia Perené, *Tovar et al.* 1536 (USM); • ibid., Dist. San Ramón, San Ramón, 11°07'17"S, 75°21'11.2"W, 772 m, 17 Aug. 2010, *Xue-Jun Ge et al*. 276 (USM); • ibid., 3000 ft, Aug. 1945, *C. Sandeman* 4938 (OXF); • ibid., along roadside, 750 m, 28 May 1979, *D.C. Wasshausen & F. Encarnación* 1133 (USM); • ibid., valle de Chanchamayo, 800–900 m, 28 June 1954, *O. Tovar* 2290 (US, USM); • ibid., Hac. Génova, 1600 m, 10 July 1962, *F. Woytkowski* 7396 (MO, US); • ibid., 11°05'26"S, 75°21'08"W, 820 m, 27 Dec. 2017, *R. Villanueva et al.* 33 (MOL); • ibid., Hac. Schunke, 1300–1700 m, 1923, *C. Schunke* A7 (US); • ibid., Lourdes de Oxabamba, 11°04'25.6"S, 75°23'24.3"W, 1227 m, 3 Aug. 2023, *R. Villanueva et al.* 920 (HOXA); • ibid., Centro Poblado Nueva Italia, 10°59'23.1"S, 75°25'7.1"W, 1245 m, 3 Aug. 2023, *R. Villanueva et al*. 935 (HOXA); • ibid., Catarata Tirol, 11°08'11.4"S, 75°20'31"W, 886 m, 4 Aug. 2023, *R. Villanueva et al. 9*42 (HOXA). **Pasco**: Prov. Oxapampa, below Oxapampa, 1500 m, 19 Oct. 1974, *P & G. Gutte* 4101 (US); • ibid., Santa Cruz, 14 Aug. 1944, *C. Ridoutt* s.n. (USM14610).

##### Typification.

As GZU-000250360 is the only specimen of *Pohl* 1989 annotated by Nees, it must be assumed to be the holotype of *Sarothecaelegans*, even though the Vienna specimen, W-0049983, is much more complete.

##### Notes.

The inflorescence can be of axillary spikes or aggregated to form a terminal panicle of spikes. The indumentum of the corolla and capsules is of subsessile glands.

﻿Species 32–35 Plants with spathulate bracts, small corollas, 5-lobed calyx and axillary spicate inflorescences.

#### ﻿﻿32. Justicia chlamydocardioides

Taxon classificationPlantaeLamialesAcanthaceae

(Mildbr.) R.Villanueva & J.R.I.Wood comb. nov.

F50C4290-14C7-5C3E-9C10-63C86FDB6222

urn:lsid:ipni.org:names:77363424-1

Tessmanniacanthus chlamydocardioides Mildbr., Notizbl. Bot. Gart. Berlin-Dahlem 9: 987. 1926. (Mildbraed 1926: 987). Type. PERU. Puerto Melendez, below Pongo de Manseriche, *Tessmann* 4788 (presumed holotype B†, photo of holotype FOBN008790, US-02869761, isotype NY-00278328).

##### Type.

Based on *Tessmanniacanthuschlamydocardioides* Mildbr.

##### Description.

Subshrub up to 2 m high. Stems dark brown with warty outgrowths. Leaves petiolate, lamina mostly 15–25 × 8–9 cm, broadly oblong elliptic, acuminate to an acute apex, base cuneate and subcordate, slightly oblique, glabrous except for the veins beneath, the venation patterning very clear abaxially. Inflorescence of shortly pedunculate spikes from the uppermost leaf axils, mostly 10–15 cm long; bracts spathulate with apiculate apex and prominent petiole, 9–11 mm long, the expended part c. 4–5 × 7 mm, thinly glandular ciliate; calyx 5-lobed to base, lobes 3 × 0.5 mm, linear-lanceolate, glabrous; corolla 12 mm long, pubescent, cream with purple lines on lower lip, thecae c. 2 × 0.5 mm, oblong, parallel, lower with a basal appendage. Capsule 10 × 4 mm, strongly clavate, glabrous (*Wasshausen* 904, *Lewis* 12985) or pubescent (*Ancuash* 1368); seeds 3.5 mm diam., rugose.

##### Illustration.

Fig. 31.

**Figure 31.**
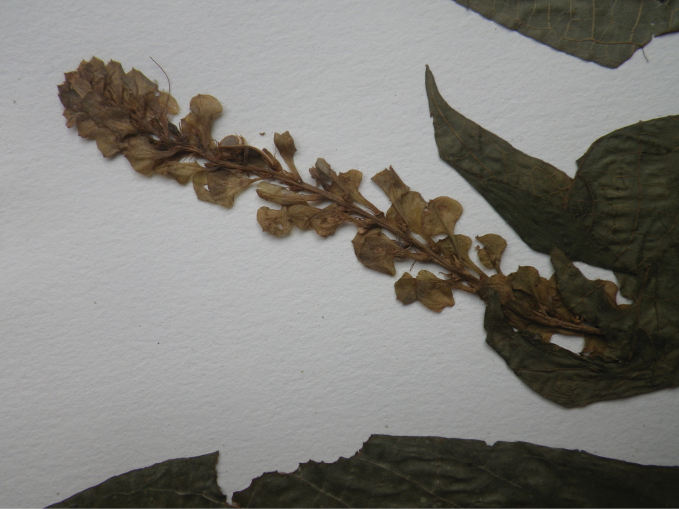
Photograph of inflorescence of *Justiciachlamydocardioides* (*Wasshausen & Encarnación* 904).

##### Phenology.

Found in flower in January to March, June to August and November.

##### Habitat.

Lowland rainforest, often near rivers 150–600 m.

##### Distribution.

Endemic to Peru in a limited area of Amazonas and Loreto near the Pongo de Manseriche. Fig. 64.

##### Material examined.

**Peru** • **Amazonas**: Prov. Condorcanqui, Dist. El Cenepa, Quebrada Huampani, 600 m, 17 July 1974, *R. Kayap* 1149 (US, USM); • ibid., 4°33'S, 78°10'W, Río Cenepa, Comunidad Tutino, 4°33'S, 78°10'W, 350 m, 21 Nov. 1993, *R. Vásquez et al*. 18416 (HUT, MOL, USM); • ibid., 300 m, 23 Nov. 1993, *R. Vásquez et al.* 18548 (HUT, US, USM); • ibid., Com. Aguaruna Agui Suwa, 4°31'35"S, 78°10'34"W, 289 m, 22 Jan. 1997, *R. Vásquez et al*. 22152 (HUT, US, USM); • ibid., vic. Huampami, 5 km E. of Chavez Valdivia, 4°30'S, 78°30'W, 200–250 m, 15 Aug. 1978, *A. Kujikat* 390 (F, MO, US); • ibid., 200 m., *E. Ancuash* 1368 (MO, US); • ibid., 15 Aug. 1978, *E. Ancuash* 1519 (US); • ibid., Com. Mamayaque, 4°03'35"S, 78°10'34"W, 260 m, 21 Feb. 1997, *E. Rodríguez et al.* 1593 (F, MO, USM). Prov. Bagua, Dist. Imaza, Com. Aguaruna de Yamayakat, 250 m, 16 July 1994, *C. Diaz et al.* 6905 (F, MO, USM); • ibid., Com. Aguaruna de Putuim, 4°55'S, 78°19'W, 480 m, 19 June 1996, *E. Rodríguez et al.* 1120 (F, HUT, MO, US, USM); • ibid., Yamayakat, Quebrada Kusu, 5°03'20"S, 78°20'23"W, 380 m, 5 Nov. 1996, *R. Vásquez et al.* 21522 (USM); • ibid., 480 m, 9 Nov. 1996, *R. Vásquez et al.* 21785 (US, USM). • **Loreto**: Prov. Alto Amazonas, Río Marañón, near Pongo de Manseriche, 200 m, 14 Feb. 1978, *D. C. Wasshausen & F. Encarnación* 904 (K, US, USM); • ibid., Dist. Pijuayil, Quebrada Tiriima, 1 km S on Río Morona, 4°22'S, 77°17'W, 150 m, 23 March 1987, *W.H. Lewis et al.* 12985 (MO); • ibid., Dist. Manseriche, Pongo de Manseriche, 4°26'01"S, 77°34'18"W, 500 m, 29 Nov. 1997, *R. Vásquez & E. Chávez* 25084 (USM).

##### Notes.

*Justiciachlamydocardioides* was described in a separate genus *Tessmanniacanthus* by Mildbraed. Mildbraed’s reasons for establishing a distinct genus are not entirely clear but seem to be based on his mistaken belief that it belonged to the Odontonemeae (i.e. *Graptophyllinae* T.Anderson) rather than the Justiciinae Nees, although he does comment that it resembles *Justiciapilosa*. He comments that it is distinguished “durch die Brakteen sehr ausgezeichnet,”, which are large relative to the flowers, broadly petiolate, the lamina broader than long, the bracteoles minute. None of these characters serve as a justification for a new genus so we have transferred this species to *Justicia*, so reducing the genus *Tessmanniacanthus* to synonymy with *Justicia*.

*Justiciachlamydocardioides* is close to *J.reniformis* J.R.I.Wood from Colombia, which is a very local endemic to the Río Claro area of Antioquia (Wood et al. 2024), so the two populations are very disjunct in distribution. *J.chlamydocardioides* differs in the larger bracts, the expanded part c. 4–5 × 7 mm (not 2–3 × 5 mm) and longer petiolar base 9–11 mm long (not 2–5 mm). In size the bracts recall another Colombian species, *J.axiologa* (Leonard) J.R.I.Wood, but the expanded part is subreniform, not suborbicular as in *J.axiologa*.

#### ﻿﻿33. Justicia schunkei

Taxon classificationPlantaeLamialesAcanthaceae

J.R.I.Wood & R.Villanueva sp. nov.

3180740F-4AAF-5931-B2C2-87E5182C0F58

urn:lsid:ipni.org:names:77363425-1

##### Type.

Peru • San Martin, Prov. “Mariscal Caceres” [Tocache], Dist. Tocache, Quebrada de Santa Rosa, Carretera a Progreso, 500–700 m, 20 July 1974, *J. Schunke Vigo* 7599 (holotype F-1882751, isotypes MO-2794266, US-2798703, USM).

##### Diagnosis.

This species can be compared with *Justiciayuyoensis* Wassh. & J.R.I.Wood but is a scandent shrub (not a herb), the bracts are < 6 mm long, mucronate and ciliate with eglandular hairs (not c. 8 mm long, ciliate with glandular hairs), the bracteoles nearly equal the bracts (not much shorter than bracts) and the calyx is c. 4 mm long, puberulent (not 2.5 mm long, glabrous).

##### Description.

Isophyllous scandent shrub 1–6 m in height; stems rounded, glabrous, greenish. Leaves petiolate, lamina 3–7.5 × 2–4 cm, ovate, acuminate to an obtuse apex, base broadly cuneate, margin irregularly undulate, both surfaces shiny, glabrous, paler beneath; petioles 0.5–0.8 cm, scurfy. Inflorescence of short axillary spikes, 1–3.5 cm long; peduncles 0–0.8 cm, puberulent; rhachis pubescent; bracts 5.5–6 × 1.5–2 mm, spathulate, apiculate, yellow-green, pilose and ciliate; bracteoles 5.5 × 1 mm, narrowly obovate, ciliate; calyx subequally 5-lobed to base, lobes 4 × 0.75 mm, narrowly ovate, finely acuminate, densely puberulent; corolla c. 7 mm long, pink with white markings, shortly pubescent on exterior in bud, tube c. 3 × 1.5 mm, upper lip 3–4 mm long, entire, lower lip 3-lobed, 3–4 mm long, lobes oblong, rounded; filaments glabrous, anther thecae weakly superposed, spreading, broadly oblong, c. 0.75 × 0.25 mm, pubescent dorsally, lower theca with a basal appendage; pollen prolate, 23 × 14–15 μm, 3-aperturate, colporate, a distinct band of sexine on either side of aperture (Fig. 51C). Capsule 9 × 3 mm, narrowly clavate, pubescent, 4-seeded; seeds 1.5–2 mm diam., rounded, flattened, ± smooth.

##### Illustration.

Fig. 32.

**Figure 32.**
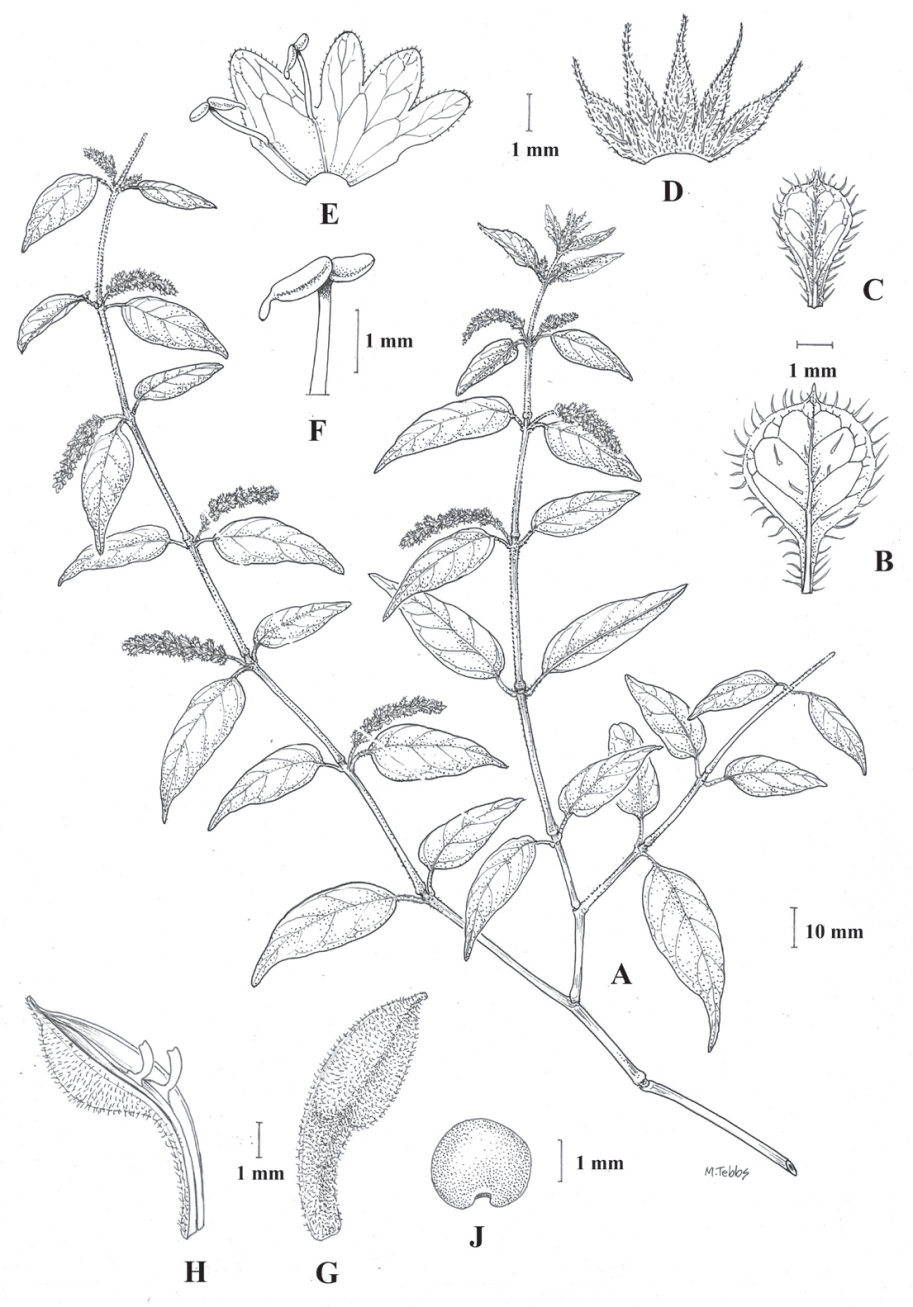
*Justiciaschunkei***A** habit **B** bract **C** bracteole **D** calyx **E** corolla **F** anther **G** exterior of capsule valve **H** interior of capsule valve **J** seed. **A, F** drawn from *Schunke Vigo* 7599, **G, J** from *Schunke Vigo* 5011 by Margaret Tebbs.

##### Etymology.

This species is named for Jose Schunke Vigo, leading Peruvian plant collector and botanist. Not only did he collect the type of *Justiciaschunkei* but some 80 other collections cited in this paper.

##### Phenology.

Flowering from July to September.

##### Habitat.

In shade of primary forest, 500–700 m.

##### Distribution.

Endemic to San Martin in Peru. Fig. 64.

##### Material examined.

**Peru** • **San Martin**: Prov. “Mariscal Cáceres” [Tocache], Dist. Tocache, the type; • ibid., desembocadura del Río Mishollo, margen derecha del Río Huallaga, 8 Sept. 1972, *J. Schunke Vigo* 5011 (F, K, US, USM).

#### ﻿﻿34. Justicia werffii

Taxon classificationPlantaeLamialesAcanthaceae

J.R.I.Wood & R.Villanueva sp. nov.

1A4354C9-B135-5217-9AC5-4CDF94E33609

urn:lsid:ipni.org:names:77363426-1

##### Type.

**Peru** • San Martin, Prov. Rioja, Carretera Rioja [6°03'S, 77°10'W]–Pedro Ruiz [5°57'S, 77°58'W], 1450 m, 2 March 1998, *H. Van der Werff* 15565 (holotype MO-5763942, isotypes US-3387908, USM).

##### Diagnosis.

A small-flowered species that resembles *J.morona-santiagoensis* Wassh. in the spathulate bracts but differs in the liana habit (not erect or ascending subshrub), short peduncles, 0.5–2 cm long (not 4–6 cm) and the ciliate bracts and bracteoles (not glabrous).

##### Description.

Liana of unknown height; stems sulcate, glabrous. Leaves petiolate, lamina 2.5–6 × 1–2.3 cm, apex acuminate, base cuneate, slightly oblique, margin entire to crenulate, subglabrous or with few hairs in the veins, numerous small cystoliths present, veins 5–6 pairs; petioles 3–7 mm, subglabrous or hirtellous. Inflorescence of solitary or paired axillary racemes 1–4 cm long, becoming aggregated apically to form a terminal thyrse, the leaves progressively reduced in size; peduncles 0.5–2 cm, glabrous; rhachis 0.5–2 cm long, scurfy; flowers on pedicels 0–2 mm long; bracts and bracteoles similar, spathulate, 4 × 1.5 mm, ciliolate; calyx 5-lobed to c. 1 mm above base, lobes 4 × 0.5 mm, lanceolate, acuminate, puberulent; corolla c. 12 mm long, white, minutely tomentellous, the tube c. 3.5 mm, the base somewhat bulbous, upper lip c. 8 mm long, minutely notched, erect, lower lip c. 6 mm, 3-lobed, lobes c. 2–2.5 × 1.5–2 mm, narrowly ovate, obtuse; anthers shortly exserted, thecae ellipsoid, c. 1.5 × 1 mm, slightly superposed, glabrous, basally acute; pollen prolate, 29–35 × 16–21 μm, 3-aperturate, colporate, 1 row of ± 7–8 distinct insulae on either side of aperture (Fig. 51B); style 12 mm, glabrous except for a few hairs at base; ovary glabrous. Capsule and seeds not seen.

##### Illustration.

Fig. 33.

**Figure 33.**
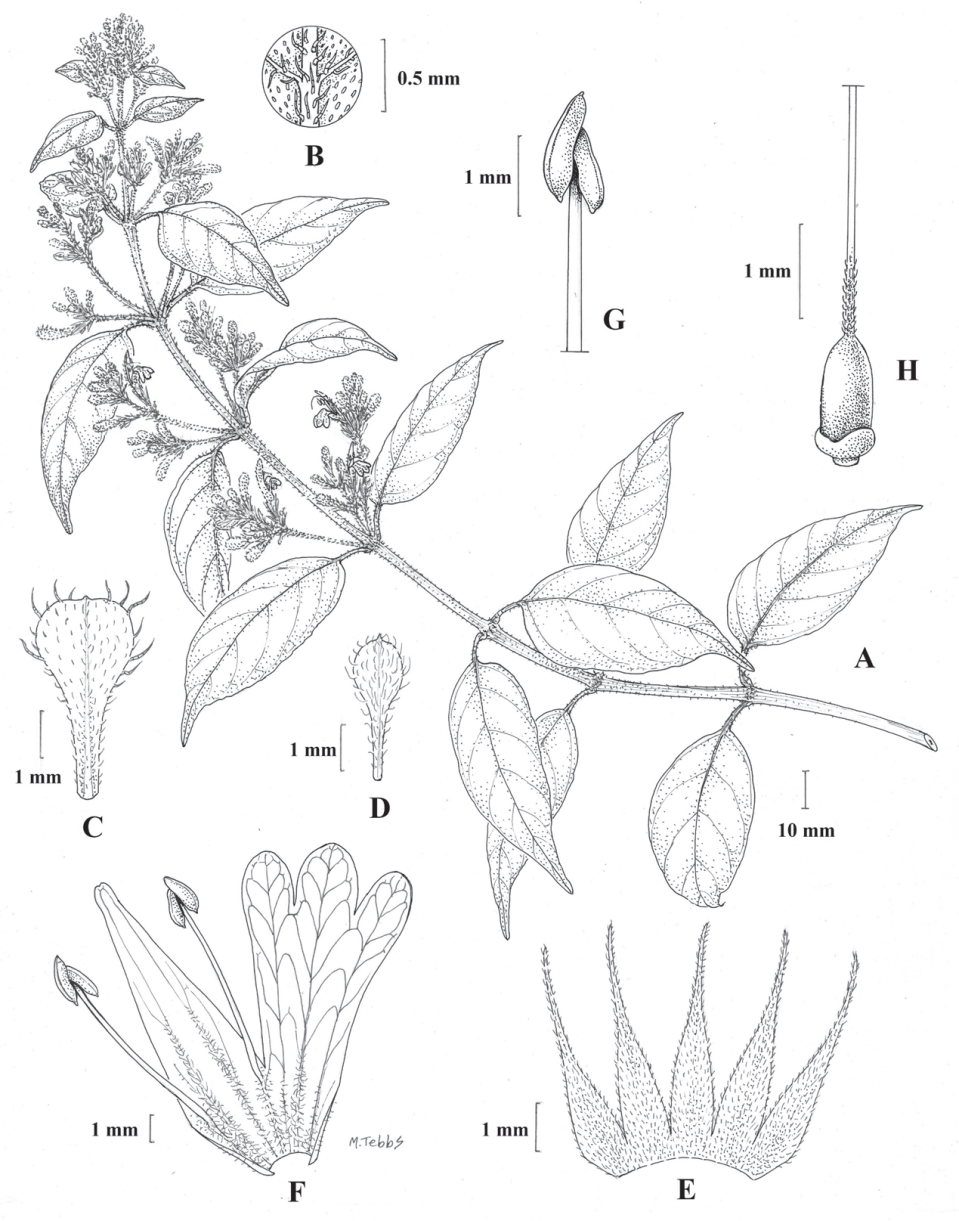
*Justiciawerffii***A** habit **B** detail of abaxial surface of leaf **C** bract **D** bracteole **E** calyx **F** corolla **G** anther **H** ovary and style. Drawn from *Van der Werff* 15565 by Margaret Tebbs.

##### Etymology.

This species is named for Henk van der Werff, who collected the type as well as many other important specimens from northern Peru.

##### Phenology.

Found in flower in March.

##### Habitat.

Montane forest on clay soil in San Martin, 1450 m.

##### Distribution.

Endemic to Peru and only known from the type, which was collected in San Martin. Fig. 64.

##### Material examined.

**Peru** • **San Martin**: type collection.

#### ﻿﻿35. Justicia spathuliformis

Taxon classificationPlantaeLamialesAcanthaceae

R.Villanueva & J.R.I Wood sp. nov.

A16EE967-DB93-5E5F-991F-F3A0B71E94B3

urn:lsid:ipni.org:names:77363427-1

##### Type.

Peru • Huánuco, Prov. Leoncio Prado, Dist. Hermilio Valdizán, Río Azul, 30 km from Tingo María, 750–850 m, 15 Oct. 1957, *R. Ferreyra* 12739 (holotype US-2267252, isotype USM).

##### Diagnosis.

Resembling *Justiciamorona-santiagoensis* Wassh. but the inflorescence is clearly terminal (not axillary), with verticillate branching, the bracts at the inflorescence branching points oblong, (not spathulate), floral bracts ciliate (not glabrous) and corolla glabrous (not with hirtellous upper lip).

##### Description.

Isophyllous shrub 0.6–1.5 m high, habit unknown but probably erect, branched; stems crisped-pubescent with multicellular hairs. Leaves petiolate, lamina 10–19 × 2–9.5 cm, narrowly oblong-elliptic, apex acuminate, base cuneate, adaxially dark green, thinly pilose with multicellular hairs, the veins pubescent, abaxially paler, sometimes reddish-purple, glabrous except for pubescent veins, lateral veins 6–8 pairs; petioles 1–5.5 cm, densely pubescent. Inflorescence terminal, noticeably shorter than the subtending leaves, consisting of a relatively stout rhachis, from which arise verticillate branches with relatively slender secondary peduncles from the upper leaf axils; primary peduncles 3.2–5.2 cm (including rhachis to 10 cm), weakly quadrangular, densely but somewhat bifariously pubescent; secondary peduncles 1.5–3.6 cm; bracts at inflorescence branching points shortly petiolate, 10–12 × 2.5 mm, oblong, foliose, glabrous, somewhat caducous, the petioles up to 2 mm long; pedicels 5–15 mm flowers clustered in short dense spikes at apex of pedicels; floral bracts 6–10 × 2.5 mm, obovate-spathulate, ciliate; calyx subequally 5-lobed to base, lobes 4 × 1.5 mm, lanceolate, acuminate, ciliolate; corolla yellow-green, minutely puberulent, tube 5 × 3 mm, stout, somewhat ventricose, mouth gaping, upper lip 4–5 mm, bidentate, lower lip purple-spotted, 3-lobed, the lobes oblong-ovate rounded; anthers included but prominent, the thecae 2.25 × 1.5 mm, oblong-elliptic, glabrous, weakly superposed, lower with a small basal appendage; pollen prolate, 27–33 × 19–22 μm, 3-aperturate, colporate, 1 row of c. 4–5 insulae on either side of the aperture (Fig. 51D). Capsule 11 × 3 mm, weakly clavate, glabrous, 4-seeded; seeds lenticular, 2 × 2 mm.

##### Illustration.

Figs 34, 35A–D.

**Figure 34.**
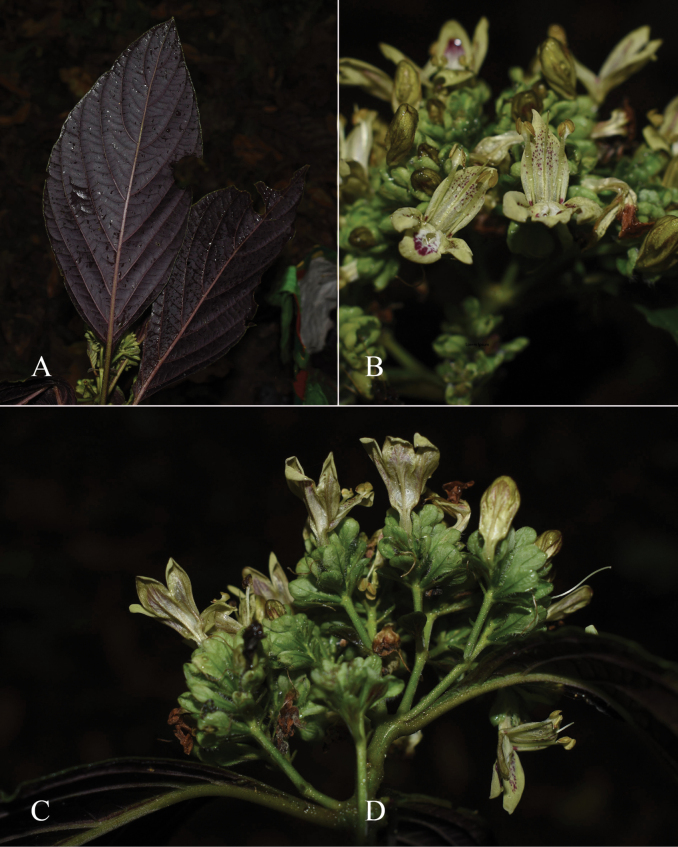
Photographs of *Justiciaspathuliformis* (*Villanueva* 1028) by Rosa Villanueva.

**Figure 35.**
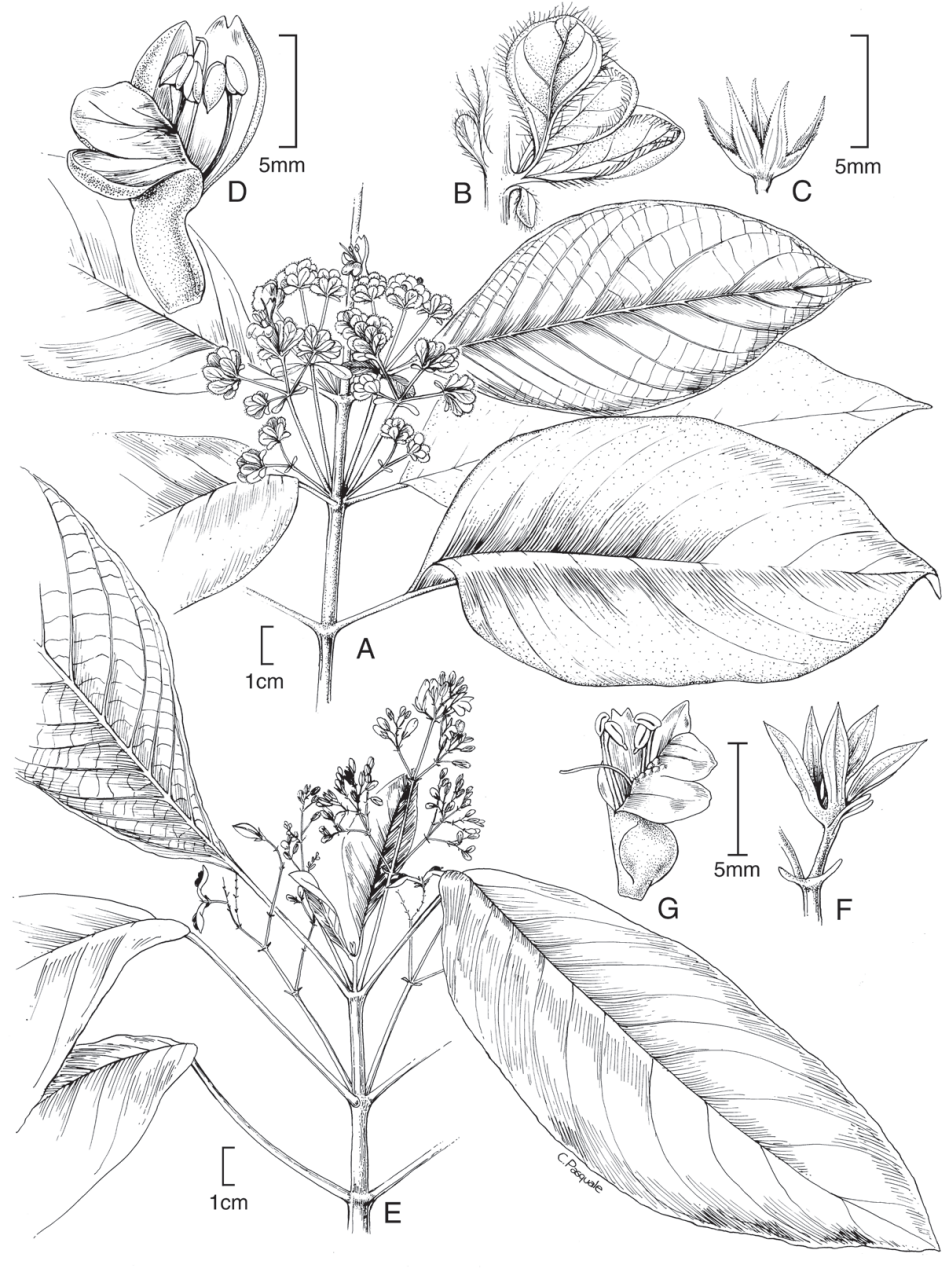
*Justiciaspathuliformis***A** habit **B** bracts **C** calyx **D** corolla. *Justiciasaccata***E** habit **F** bracts and bracteoles **G** corolla. **A, D** drawn from *Ferreyra* 12739, **E, G** from *Schunke* 3264 by Cathy Pasquale.

##### Etymology.

The epithet “*spathuliformis*” refers to the distinctive spathulate bracts, which are characteristic of this species.

##### Phenology.

Found in flower from June to October.

##### Habitat.

c. 350–800 m. Primary Forest, often near streams.

##### Distribution.

Endemic to Peru but relatively widely distributed on the eastern Andean slopes from San Martin south to Cusco. Fig. 64.

##### Material examined.

**Peru** • **Cusco**: Prov. La Convención, Dist. Echarate, Sepriato, margen derecha del Río Camisea, Reserva Nacional Nahua Kogapakori, 11°49'S, 78°33'W, 430 m, 19 July 2007, *H. Beltrán et al.* 6413 (USM). • **Junín**: Prov. Chanchamayo, Dist. Chanchamayo, La Merced, 400–500 m, 14 Aug. 1948, *R. Ferreyra* 4470 (USM). • **Huánuco**: Prov. Leoncio Prado, hills east of Tingo María [9°18'S, 75°59'W], 5 Oct. 1972, *T.B. Croat* 21176 (US); • ibid., Dist. Hermilio Valdizán, Río Azul, 30 km from Tingo María, 750–850 m, 15 Oct. 1957, *R. Ferreyra* 12739 (US, USM); • ibid., Dist. Daniel Alomias Robles, oeste del Restaurante Canabraba, cerca de Delicios, 800–900 m, 17 June 1976, *J. Schunke V*. 9296 (MEXU, MO, US); • ibid., Dist. José Crespo y Castillo, 8°40'S, 76°05'W, 673 m, 11 Aug. 2023, *R. Villanueva et al.* 1028 (HOXA, IBSC); • ibid., Dist. Rupa-Rupa, Tingo María, 625–1100 m, 1949–1950, *H.A. Allard* 20611 (US); • ibid., *H.A. Allard* 21902 (US). • **San Martin.** Prov. Tocache [Mariscal Cáceres], Dist. Tocache [Nuevo], desembocadura del Río Mishollo en Río Huallaga, 350–380 m, 25 July 1973, *J. Schunke V.* 6423 (US); • ibid., el este del puente, 500 m, 27 July 1974, *J. Schunke V*. 7816 (F, MO, US, USM). • **Ucayali**: Prov. Padre Abad, Dist. Padre Abad, Cuenca del Río Aguaytía, carretera al Río Yurac 9°04'S, 75°36'W, 350 m, 9 Oct. 2004, *J. Schunke V. & J.G. Graham 16322* (F, US, MOL).

##### Note.

This species has a gaping corolla, so giving the appearance of having strongly exserted anthers. *Schunke* 7816 has large ovate, subcordate, somewhat oblique leaves, the other cited specimens have broadly oblong-elliptic leaves with cuneate base.

﻿﻿Species 35–36. Species with axillary inflorescences, 5-lobed calyx and small flowers, the corolla < 15 mm long.

#### ﻿﻿36. Justicia saccata

Taxon classificationPlantaeLamialesAcanthaceae

R.Villanueva & J.R.I.Wood sp. nov.

195AD858-8B47-597F-B099-F25099F93899

urn:lsid:ipni.org:names:77363429-1

##### Type.

Peru. Huánuco, Prov. Leoncio Prado, Dist. Rupa-Rupa, Quebrada Las Pavas, 5 km S. of Tingo María, 720 m, 24 March 1976, *T. Plowman & H. Kennedy* 5722 (holotype US-2728351, isotype USM).

##### Diagnosis.

Resembling *Justiciachloanantha* Leonard in the large leaves, panicled inflorescence with white flowers and short calyx lobes but the corolla shorter, < 10 mm long, with a prominent ventral bulge (not corolla c. 15 mm long, lacking a ventral bulge), calyx lobes very narrowly oblong-elliptic to oblanceolate, appressed to the corolla, (not narrowly linear-lanceolate to subulate and spreading) and the capsule glabrous (not glandular-pubescent). It might be confused with *J.wallnoeferi* Wassh. and *J.cuzcoensis* Lindau but the leaves are long-petiolate and flowers not arranged in opposite pairs, the calyx 4–5 mm long (not 3 mm long) and corolla minutely glandular-puberulent with ventral bulge (not pubescent and lacking a ventral bulge).

##### Description.

Isophyllous subshrub c. 1 5 m high; stem glabrous. Leaves petiolate, lamina 12–29 × 4–12 cm, broadly oblong-elliptic, apex shortly acuminate, base broadly cuneate, both surfaces glabrous with numerous cystoliths, abaxially paler, lateral veins 12 pairs; petioles 8–10 cm long, glabrous. Inflorescence of pedunculate thyrses from the upper leaf axils, these much shorter than the leaves; peduncles 1–11 cm, glabrous, rhachis mostly 4–6 cm long, secondary peduncles 2–3 cm, tertiary peduncles 5–8 mm; bracts 2–5 mm, filiform, caducous; calyx subequally 5-lobed to base, lobes 5 × 1 mm, very narrowly oblong-elliptic to oblanceolate, shortly acuminate, minutely scabrous; corolla c. 8–9 mm long, cream with purplish veins, minutely glandular-puberulent, tube c. 4 × 2 mm, stout with prominent ventricose bulge, 2-lipped, upper lip c. 3 mm long, bidentate, lower lip with purplish “herring bone” patterning, c. 4 mm long, 3-lobed, the lobes obovate, rounded; filaments glabrous, anthers included in the upper lip, thecae superposed, c. 1 × 0.75 mm, basally muticous, glabrous; pollen prolate, 25–28 × 16–19 μm, 3-aperturate, colporate, 1 row of c. 6 insulae on either side of the aperture (Fig. 51E). Capsule 10–12 × 3–4 mm, clavate, glabrous, 4-seeded; seeds lenticular, 1.75 mm diam., dark brown, smooth.

##### Illustration.

Figs 35E–G, 36.

**Figure 36.**
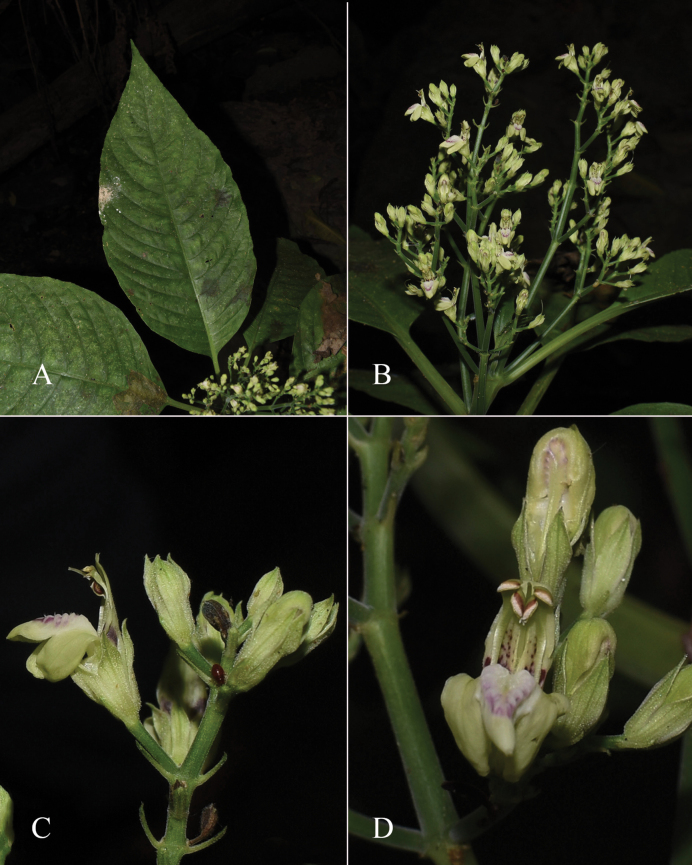
Photographs of *Justiciasaccata* (*Villanueva* 1018) by Rosa Villanueva.

##### Etymology.

The epithet “*saccata*” refers to the distinctive saccate or ventricose corolla, which is a characteristic feature of this species.

##### Phenology.

Flowering from March to September.

##### Habitat.

In forest shade along rocky river banks and river cliffs, c. 380–950 m.

##### Distribution.

Endemic to Peru, where it is known from Huánuco and Pasco. Fig. 65.

##### Material examined.

**Peru** • **Huánuco**: Prov. Leoncio Prado, Dist. Rupa-Rupa, Cueva de las Pavas, 5 km S of Tingo María, [9°19'19"S, 75°59'36"W] 672 m, 4 July 1969, *J. Schunke Vigo* 3264 (F, K, US, USM); • ibid., quebrada Las Pavas, 5 km S. of Tingo María, 720 m, 24 March 1976, *T. Plowman & H. Kennedy* 5722 (US, USM); • ibid., Cueva de las Pavas, 700–750 m, 11 July 1948, *R. Ferreyra* 4133 (USM); • ibid., Dist. Mariano Damaso Beraun, Catarata Derrepente, 09°29'57"S, 75°58'38"W, 938 m, 9 Aug. 2023, *R. Villanueva et al*. 997 (HOXA); • ibid., cerca de la Poza de la Doncella, Cueva de las pavas, 9°22'15.3"S, 75°58'28.1"W, 694 m, 10 Aug. 2023, *R. Villanueva et al.* 1018 (HOXA). • **Pasco**: Prov. Oxapampa, Dist. Palcazú, quebrada Ataz, 10°10'06"S, 75°19'23"W, 407 m, 28 May 2009, *L. Valenzuela et al.* 13027 (HOXA, MO, USM); • ibid., Ataz, camino al convento, 10°09'30"S, 75°19'34"W, 375–635 m, 28 May 2009, *L. Valenzuela et al.* 12009 (HOXA, USM); • ibid., P. N. Yanachaga-Chemillén, Est. Biológica Paujil Pozo Tigre, 10°20'16"S, 75°15'07"W, 450 m, 31 March 2006, *R. Vásquez et al.* 31357 (HOXA); • ibid., 10°19'55"S, 75°15'58"W, 400 m, 10 March 2007, *R. Vásquez et al.* 32095 (HOXA); • ibid., 10°19'31"S, 75°15'51"W, 380 m, 10 March 2009, *R. Vásquez et al.* 35533 (HOXA, HUT, MOL, USM); • ibid., Estación Paujil-camino a la quebrada Ozus, 10°18'20"S, 75°16'52"W, 479 m, 17 March 2009, *R. Vásquez et al.* 35664 (HOXA, MOL, USM); • ibid., camino a parcela permanente ubicada en el Cerro Ozus, 10°18'37.5"S, 75°17'18.8"W, 850–1010 m, 26 Sept. 2005, *Vilca* 371 (HOXA, USM).

##### Note.

*L. Valenzuela & J.L. Mateo* 13175 (MO) is close to *Justiciasaccata*. It has the same ventricose corolla but differs in the more prominently hirtellous corolla and the subsessile leaves which are basally truncate.

#### ﻿﻿37. Justicia secundiflora

Taxon classificationPlantaeLamialesAcanthaceae

(Ruiz & Pav.) Vahl, Enum. Pl. 1:159. 1804. (Vahl 1804: 158)

2013C72C-02C5-532E-AFF8-2F08EC603D6D

Dianthera secundiflora Ruiz & Pav., Fl. Peruv. Prodr.1: 11. 1798. (Ruiz and Pavón 1798: 11) Type. PERU. *Ruiz & Pavon* s.n. (lectotype MA815540, designated here, isolectotypes BC-872859, BM-014608695, G-00236348, G-00236349, G-00236350, MA-815542, OXF-00194112).
Leptostachya secundiflora (Ruiz & Pav.) Nees, Prodr. [A. P. de Candolle] 11: 378. 1847. (Nees 1847b: 378)
Leptostachya poeppigiana Nees, Flora Bras. 9: 150. 1847. (Nees 1847a: 150) Type. BRAZIL, Ega, Rio Amazon, *Poeppig* 2485β (lectotype W-0049994, designated here), syn. nov.
Ecbolium poeppigianum (Nees) Kuntze, Rev. Gen. Pl. 2: 981. 1891. (Kuntze 1891: 981)
Justicia poeppigiana (Nees) Lindau, Bot. Jahrb. Syst. 19(4), Beibl. 48: 20. 1894. (Lindau 1894: 20)

##### Type.

Based on *Diantherasecundiflora* Ruiz & Pav.

##### Description.

Subshrub 1–3 m high; stems glabrous. Leaves petiolate, lamina 10–23 × 3.5–11 cm, obovate-elliptic, apex obtuse to very shortly acuminate, base cuneate, lateral veins 7–10; petioles 2–4 cm. Inflorescences composed of axillary 1-sided spikes, up to 18 cm long these simple or branched, the branches verticillate with 1–4 secondary branches arising at each node; ultimate spikes 2–6 cm long, the flowers c. 3 mm distant, rachis pulverulent; bracts at inflorescence branching points 10 × c. 2 mm, puberulent; floral bracts deltoid 1 × 0.5 mm; bracteoles 2 × 1 mm, deltoid, both puberulent; calyx 4-lobed to near base, lobes 3 × 1 mm; corolla 8 mm long, cream mottled purple, puberulent; anthers superposed, 0.9 × 0.5 mm, subquadrangular, glabrous, lower with a basal appendage. Capsule 15–16 × 2.5 mm, oblong, puberulent, 4-seeded; seeds rugose.

##### Illustration.

Fig. 37.

**Figure 37.**
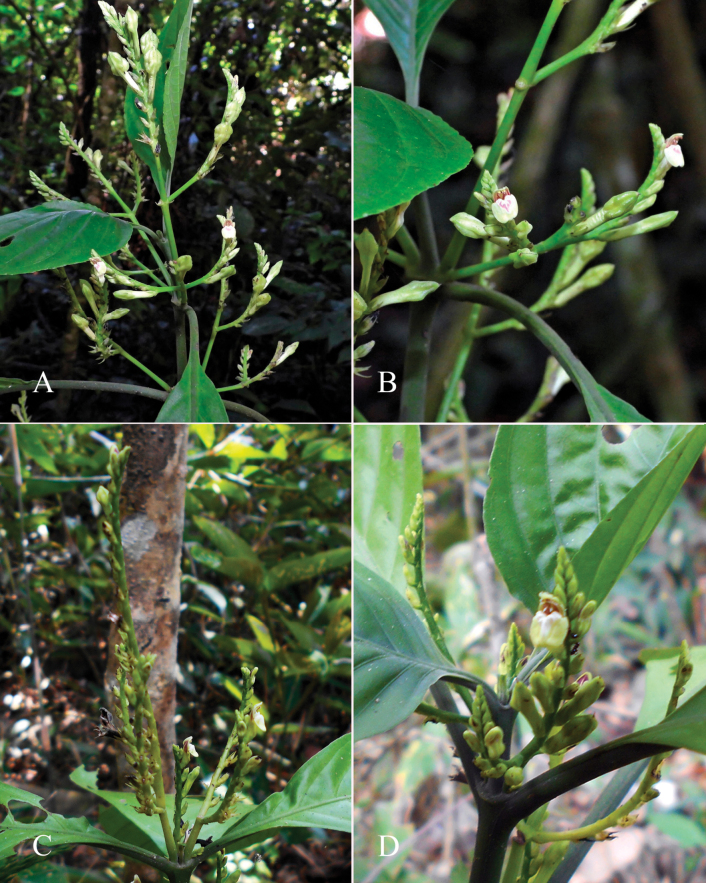
Photographs of *Justiciasecundiflora***A, B** (*Vásquez* 46813) Rodolfo Vásquez **C, D** (*Azevedo* 174) Igor Azevedo

##### Habitat.

Lowland rainforest, mostly at altitudes below 600 m, but exceptionally up to 1368 m.

##### Phenology.

Mostly flowering from June to October.

##### Distribution.

Amazonian regions of Peru, Amazonian Brazil and the south of Ecuador. Fig. 66.

##### Material examined.

**Peru** • **Amazonas**: Prov. Condorcanqui, Dist. Río Santiago, Cerro Kampankis, 3°06'37"S, 77°46'07"W, 450 m, 6 Aug. 2011, *I. Huamantupa et al.* 15412 (USM); • ibid., Dist. El Cenepa, Quebrada Satik entsa, 600 ft, 16 July 1974, *R. Kayap* 1132 (US, USM); • ibid., Comunidad de Tutino, 04°33'05"S, 78°12'54"W, 340 m, 28 July 1977, *R. Vásquez et al*. 24479 (HUT, US, USM); • ibid., 04°34'05"S, 78°11'53"W, 28 June 1997, *R. Vásquez et al.* 24289 (USM); • ibid., 04°29'30"S, 78°10'30"W, 300 m, 16 June 1997, *R. Vásquez et al.* 24052 (USM). Prov. Bagua, rainforest along Río Marañón 2–10 km above mouth of Río Santiago, 250–275 m, 14–15 Oct. 1962, *J. Wurdack* 2249 (US, USM). • **Ayacucho**: Prov. La Mar, between Santa Rosa y Hac. Luisiana, 640 m, 8 Sept. 1976, *D.C. Wasshausen & F. Encarnación* 626 (K, USM); • ibid., 585 m, 7 June 1968, *T.R. Dudley* 9088B (F). **Cusco**: Prov. La Convención, Quempiri, caserío Campa, 460–480 m, 24 July 1965, *R. Ferreyra* 16361 (USM); • ibid., Camp. Malvinas, 11°52'12"S, 72°56'28"W, 23 Sept. 1997, *P. Acevedo-Rodríguez & F. Ramírez* 9885 (F, K, MO, US, USM); • ibid., Chokoriari, 11°51'S, 72°57'W, 400 m, *P. Nuñez et al.* 20877 (CUZ, F, USM). • **Huánuco**: Prov. Puerto Inca, Dist. Codo de Pozuzo, 9°40'S, 75°25'W, 450 m, 18 Oct. 1982, *R. Foster* 9278 (USM, US); • ibid., Dist. Puerto Inca, Santa Antonio en la cocha, 200 m, 29 Aug. 2019, *I. Azevedo & R. Villanueva* 174 (HOXA); • ibid., Dist. Rupa-Rupa, Villa Isabel-Cucharas, 550 m, 24 July 1954, *F. Woytkowski* 1247 (MOL). Prov. Pachitea, western part of Sira mountains, 9°28'S, 74°48'W, 31 July 1988, *B. Wallnoefer* 115-31788 (US). • **Junín**: Prov. Satipo, Dist. Río Tambo, Com. Nativa Pichiquia, 11°22'12"S, 74°02'19"W, 1348 m, 13 July 2013, *L. Valenzuela et al.* 25073 (HOXA, MO). Prov. Chanchamayo, Dist. La Merced, entre Situlli y Cerro Santa Cruz, c. 86 km. de Tingo María, 500–600 m, 3 Aug. 1948, *R. Ferreyra* 4383 (USM, US). Prov. Datém del Marañón, Dist. Manseriche, Santa Rosa, lower Río Huallaga below Yurimaguas, 135 m, 1–5 Sept. 1929, *E. P. Killip & A. C. Smith* 28833 (US). **Loreto**: Prov. Alto Amazonas, Dist. Acapulco, 25 March 2009, *M. Chocce et al.* 4991 (USM); • ibid., Dist. Yurimaguas, Between Yurimaguas and Balsapuerto (lower Río Huallaga basin), 135–150 m, 26–31 Aug. 1929, *E. P. Killip & A. C. Smith* 28343 (F, US); • ibid., Dist. Balsapuerto (lower Río Huallaga basin), 150–350 m, 28–30 Aug. 1929, *E. P. Killip & A. C. Smith* 28635 (F, US); • ibid., *E. P. Killip & A. C. Smith* 28654 (US). Prov. Ucayali, Dist. Pampa Hermosa, Parque Nacional Cordillera Azul, Sector PV-106, 175 m, 15 Sept. 2002, *R. Vásquez et al.* 47524 (USM). • **Madre de Dios**: Prov. Manu, P.N. Manu, 5 km due north of Est. Biol. Cocha Cashu, 11°52'S, 71°22'W, 15 Aug. 1983, *Al. Gentry* 43606 (MO); • ibid., 11°40'S, 71°55'W, 400–450 m, 2 Oct. 1986, *R. B. Foster et al.* 11581 (F); • ibid., 15 Aug. 1976, *R. B. Foster & C. Augspurger* 3160 (F); • ibid., Cocha Cashu uplands, 11°45'S, 71°0'W, 400 m, 22 Aug. 1986, *P. Nuñez* 5895 (USM); • ibid., 5 Sept. 1989, *A. Tupayachi* 1196 (CUZ27808). • **Pasco**: Prov. Oxapampa, Dist. Palcazú, Iscozacin, Est. Biol. Paujil, 10°21'27"S, 75°14'50"W, 405 m, 13 Aug. 2010, *J. Perea et al.* 4501 (HOXA, USM); • ibid., 10°20'58"S, 75°16'10"W, 386 m, 17 Aug. 2010, *J. Perea et al.* 4578 (HOXA, USM); • ibid., Ataz, camino al Convento, 10°09'30"S, 75°19'34"W, 375–635 m, 9 Aug. 2008, *L. Valenzuela et al.* 11965 (HOXA); • ibid., quebrada Ataz, 10°10'06"S, 75°19'23"W, 407 m, 28 May 2009, *L. Valenzuela et al.* 13035 (HOXA, USM); • ibid., P. N. Yanachaga Chemillén, trocha Estación Biológica Paujil a Pozo Tigre, 400 m, 10°19'55"S, 75°6'00"W, 12 July 2007, *A. Monteagudo et al.* 14217 (HOXA); • ibid., Dist. Puerto Bermudez, Valle Pichis, Santa Rosa de Chivis, Río Nochos, 9 km SW of Puerto Bermúdez on new highway, 10°20'S, 74°58'W, 300–400 m, 7 Sept. 1982, *R. Foster* 8595 (MEXU, F, US, USM); • ibid., 375 m, 14–17 July 1929, *E.P. Killip & A.C. Smith* 26646 (F, MA, P, US); • ibid., Cahuapanas, on Río Pichis, 340 m, 20–21 July 1929, *E.P. Killip & A.C. Smith* 26815 (US); • ibid., Dist. Villa Rica, 0°45'28"S, 74°55'92"W 1355 m, 6 July 2003, *J. Perea & C. Mateo* 0194 (HOXA, US). • **San Martin**: Prov. San Martín, Dist. Tarapoto, 1835, *A. Mathews* 1596 (K, OXF). Prov. Tocache, Dist. Tocache, Río Huallaga, fundo Miramar, a 3 km de Tocache Nuevo, 400 m, 19 Aug. 1969, *J. Schunke Vigo* 3352 (F, MOL, US, USM); • ibid., Dist. Uchiza, Cachuyacu de Lepuna, 450–500 m, 11 July 1974, *J. Schunke Vigo* 7314 (F, US, USM). Prov. Bellavista, Dist. Alto Biavo, Sector Las Palmas, Parque Nacional Cordillera Azul, Puesto de Control 20 “Mojarra”, 7°35'S, 76°11'W, 772 m, 13 Sept. 2019, *L. Valenzuela et al.* 36857 (HOXA). • **Ucayali**: Prov. Padre Abad, Río Aguaytía, carretera a Caserío San Miguel y Mapuya, 350 m, 9°05'S, 75°26'W, *J. Schunke Vigo & J.G. Graham* 16210 (F, MOL, US).

##### Lectotypification.

Of the original material of *Diantherasecundiflora* at Madrid, MA815540 is much the best material and is accordingly chosen as the lectotype. In describing *Leptostachyapoeppigiana* Nees cited two collections by Poeppig, “In Maynas et ad Egam [Tefe]oppidum, circum flumen Amazonum”, the former from Peru and the latter from Brazil. Two specimens from Egam are annotated by Nees, one in his own herbarium (GZU-000250901) and one in Vienna (W-00499940). Neither specimen is very adequate but as GZU-000250901 is a fragment, apparently removed from W-0049994, the latter is selected as lectotype, being the original material. There is a fragment also apparently removed from the Vienna specimen at the Smithsonian (US-02880220).

##### Note.

*Acevedo-Rodriguez* 9885 from Cusco, La Convención, has lanceolate (rather than deltoid), less obviously puberulent bracts and a larger corolla and may represent a different species.

﻿﻿Species 38–45 Miscellaneous species with large corollas exceeding 2.5 cm in length and which do not fit any of the earlier groupings.

#### ﻿﻿38. Justicia sericea

Taxon classificationPlantaeLamialesAcanthaceae

Ruiz & Pav., Fl. Peruv. Prodr.1: 12. 1798 (Ruiz & Pavón 1798: 12)

A83EF6B9-0A8E-5AC1-A770-35EEF963EB17

##### Type.

Peru • [Junín], Tarma, *Ruiz & Pavon* s.n. (lectotype MA-815544, designated here, possible isolectotypes BC-872769, BM-000992567, MA-815543, MA-817208, OXF-00194108, US- 02880852).

##### Description.

Shrub 1–2 m high, stems sericeous, often with dark corky deposits on angles. Leaves subsessile, lamina 3.5–6.5 × 1–1.5 cm, lanceolate to oblong-elliptic, acute, sericeous, coriaceous, yellow-green, lateral veins c. 3 pairs. Inflorescence of few-flowered axillary and terminal spikes, 2.5–3 cm long; bracts oblong-ovate, acuminate, 15 × 4–5 mm; bracteoles linear; calyx deeply 5-lobed, lobes lanceolate, 10 × 2 mm; corolla c. 4.5 cm long, red, hirsute, upper lip 2 cm long, emarginate, lower lip with lobes ovate, 1.5 cm long; anthers purplish, the thecae 2.5–3 × 0.75 mm, held at same level, slightly unequal, parallel, glabrous, base acute. Capsule 22 × 7 mm, oblong, clavate, glabrous, 4-seeded; seeds 5 × 4 mm, wrinkled.

##### Illustration.

Fig. 38.

**Figure 38.**
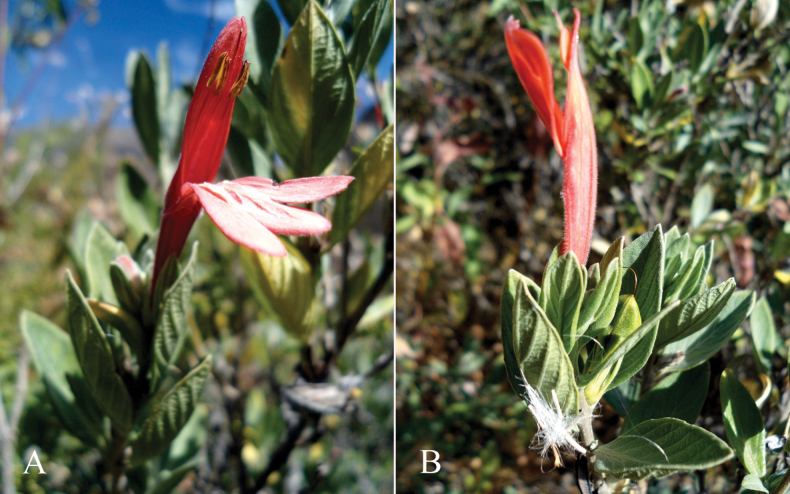
Photographs of *Justiciasericea* by Hamilton Beltrán.

##### Phenology.

Flowering mostly in the dry season from October to May.

##### Habitat.

Dry Andean Forest on stony ground. Almost restricted to 2400–3400 m, thus growing at higher altitudes than other species except *J.alpina*.

##### Distribution.

NW Peru and southern Ecuador, one of the few species of *Justicia* found on both sides of the Andean cordillera. Fig. 67.

##### Material examined.

**Peru** • sine data: *Maclean* s.n. (K); “Huanca”, *Pearce* 495 (K). • **Ancash**: Prov. Huaraz, 8000 ft, 6 Oct. 1922, *MacBride & Featherstone* 2521 (F, US); • ibid., Purchased in Huaraz market, 14 March 1964, *P. Hutchinson & J. Wright* 4376 (US, USM); • ibid., 15 km S of Huaraz, 09°41'S, 77°29'W, 3450 m, 27 Jan. 1985, *D. Smith et al.* 9386 (US, USM).Prov. Huaylas, debajo de Huaylas, 2500 m, 4 July 1988, *E. Cerrate et al.* 8984 (USM). Prov. Pallasca, Dist. Conchucos, 2900–3150 m, 25 May 2012, *A. Cano et al.* 21245 (USM). Prov. Bolognesi, Entre Chiquian y Aquia, 2900–3000 m, 18 May 1950, *R. Ferreyra* 7553 (K, MO, MOL, US, USM); • ibid., Entre Chiquian y Tallenga, 3000 m, 14 March 1903, *A. Weberbauer* 2857 (MOL, USM); • ibid., near Huasta, 10000 ft, 1970, *C. Boutin* 26975 (US); • ibid., Dist. Pacllón, Llamac, 9000ft, *M. Slesser* 6 (K); • ibid., Dist. Chiquian, road from Chiquian to Huallanca, 3220 m, 17 March 2001, *M. Weigend* 5192 (HUT). • **Ayacucho**: Prov. Huanta, NE of Huanta, 3200 m, 1–10 Feb. 1926, *A. Weberbauer* 7520 (F, US). • **Cajamarca**: Between Cajamarca and Huánuco, *Matthews* 796 (K, OXF). Prov. Cajamarca, Baños del Inca, El Chicche-Sengal, 2800 m, 10 Feb. 2001, *I. Sánchez Vega* 10371 (CPUN, F). Prov. Contumazá, Dist. Chilete, Bosque de Huertas, *L. Dávila* 847 (Laboratorio de dendrología, Cajamarca). • **Huancavelica**: Prov. Huancavelica, Cañón del Río Mantaro, north of Mejorada, 2940 m, 28 Oct. 1957, *P.C. Hutchison* 1671 (F, K, US, USM); • ibid., Dist. Izcuhaca, 8600 ft, 16 April 1960, *S.C.E. Saunders* 479 (K). • **Huánuco**: Quinna, 28 Oct. 1927. *M. Sawada* P98 (F). • **Junín**: Prov. Huancayo, Dist. Huancayo, Cerro Corona del Fraile, Jan. 1948. *J. Soukup* 3581 (US); • ibid., Dist. Colca, 3120 m, 18 Nov. 1989, *G. Yarupaitán* 49 (USM); • ibid., Dist. El Tambo, Hac. la Mejorada, 2900 m, *W. Hoffman* 159 (USM); • ibid., Cercanías de Pasco, al paso del ferrocarril a Huancavelica, 3000 m, 20 Jan. 1950, *C. Ochoa* 762 (US). Prov. Jauja, Dist. Acolla, 3600 m, 29 Oct. 1979, *C. Hastorf* 156 (USM). Prov. Tarma, Dist. Acobamba, 3021 m, 8 Dec. 1999, *M. Binder & A. Daxberger* 417 (USM); • ibid., Santuario de Muruhuay, camino San Ramón–La Merced, 2800 m, Nov. 1948, *C. Ochoa* 625 (MOL, US); • ibid., Sector 9 de Octubre, alrededores de Muruhuay, 2800 m, 25 Oct. 2007, *J.L. Marcelo Peña* 2931 (MOL, USM); • ibid., Cerca de Acobamba, 3000 m, 11 Aug. 1961, *Pizarro* s.n. (USM). Prov. Yauli, Dist. Paccha, a 20 km, sur de Huancayo, 25 April 1961, *O. Tovar* 3284 (MO, US); • ibid., *O. Tovar* 3315 (USM). • **La Libertad**: Prov. Otuzco, 2615 m, 19 June 1950, *O. Velarde* 3567 (US); • ibid., Río Pollo, 2625 m, 109 June 1950, *N. Angulo* 0930 (US, USM); • ibid., Río Huangamarca arriba, 2600 m, 26 Sept. 1997, *E. Rodríguez et al*. 1872 (HUT); • ibid., Trujillo–Otuzco road, 7 km from Otuzco, 2550 m, 13 Feb. 1983, *D.N. Smith & R. Vásquez* 3254 (US); • ibid., Dist. Agallpampa, abajo de José Balta, 2600 m, 30 Oct. 1993, *S. Leiva* 954 (F, HUT, MO, US). Prov. Trujillo, 2–3 km, arriba de Otuzco, carretera Trujillo–Otuzco, 2500 m, 15 May 1948, *R. Ferreyra* 2976 (MOL, USM). • **Lima**: Prov. Cajatambo, Dist. Cajatambo, 16 Oct. 1966, La Rosa 1805 (USM). Prov. Oyón, Dist. Oyón, Oyón, 2400 m, March 1987, *O. Cuya* 209 (USM); • ibid., Viroc, 10°41'50"S, 76°49'29"W, 3040 m, 1 June 2013, *C. Aedo & J. Molina* 20631 (USM). Prov. Canta, Dist. San Buenaventura, San Buenaventura, en el camino del Puente Verde, 2600 m, 10 April 2010, *G. Vilcapoma* 7934 (USM). Prov. Huaral, Dist. San Miguel de Acos, Huascoy, 3300 m, 5 Sept. 1974, *P. Waechter* s.n. (USM). • **Pasco**: Camino entre la Quinua y Huariaca, 24 Nov. 1927, *N. Esposto* s.n. (MOL); • ibid., debajo de Chicón, 3120 m, 8 Feb. 1984, *S. Rivas et al.* s.n. (USM); • ibid., Cerro de Pasco-Huánuco, km 40, 10°28.5'S, 76°10.8'W, 3400 m, 22 Aug. 2002, *A.N. Schmidt-Lebuhn* 525 (USM). Prov. Pasco, Dist. Huariaca, between Cerro de Pasco and Ambo, 3000 m, 24 Nov. 1945, *R.J. Seibert* 2212, 2213, 2214 (US); • ibid., Dist. Ticlacayan, Piquilhuanca, 3.3 km al sur de Huariaca, 2950 m, 10 Aug. 2001, *S. Baldeón et al.* 4942 (USM).

##### Lectotypification.

The lectotype has been selected from material at MA. MA815544 is the most complete material and has the label Tarma as specified in the protologue.

#### ﻿﻿39. Justicia lactiflora

Taxon classificationPlantaeLamialesAcanthaceae

J.R.I.Wood & R.Villanueva sp. nov.

5F98ACC2-69F5-54F2-8A00-73194064F8C6

urn:lsid:ipni.org:names:77363430-1

##### Type.

**Peru** • Ucayali, Prov. Padre Abad, Pucallpa–Tingo María road, km 139, Puente Chio, 10 Sept. 1980, *P.J.M. Maas et al.* 4554 (holotype US-2949117, isotypes K-000544763, U, USM).

##### Diagnosis.

Clambering shrub similar in habit to *Justiciascansilis* and *J.pyrrhostachya*, differing most clearly from the former by the greenish lanceolate bracts up to 3 mm wide (not ovate, reddish, 2–2.5 × 1.5–2.5 cm) and from the latter by the white or whitish flowers (not red or orange-red) and the membranous (not scarious) bracts only c. 12–15 × 3 mm (not more than 15 × 10 mm).

##### Description.

Isophyllous clambering shrub 1–8 m in height; stems woody below, bifariously hirtellous. Leaves petiolate, lamina 4–13 × 1.2–5.3 cm, ovate to oblong-ovate, acuminate, base broadly cuneate, both surfaces puberulent when young soon glabrescent, abaxially paler, minutely punctate, lateral veins 6 pairs; petioles 0.3–1.7 cm, puberulent. Inflorescence of pedunculate axillary and terminal, foliose, cymose capitula; primary peduncles to 3 cm long, bifariously puberulent; pedicels 0–0.9 mm, puberulent; bracts 12 × 3 mm, lanceolate, pubescent; bracteoles similar but only c. 1–2 mm long; calyx 5-lobed, lobes 10–11 × 2.5–3 mm, lanceolate, acuminate to a very acute apex, minutely pubescent; corolla c. 3 cm long, white, variously suffused with purple, the veins prominent, thinly pubescent, upper lip hooded c. 10 × 12 mm, ovate, emarginate, lower lip broadly ovate to suborbicular, c. 20 × 20 mm, very shallowly 3-lobed, lobes c. 1 × 2–3 mm; filaments glabrous, anther thecae 2 × 1.5 mm, superposed, parallel, pubescent, shortly oblong, lower with white basal appendage; pollen prolate, 46–47 × 29 μm, 2-aperturate, colporate, 1 row of c. 7–8 peninsulae on either side of aperture (Fig. 51F); ovary pilose, style pilose. Capsule and seeds not seen.

##### Illustration.

Fig. 39.

**Figure 39.**
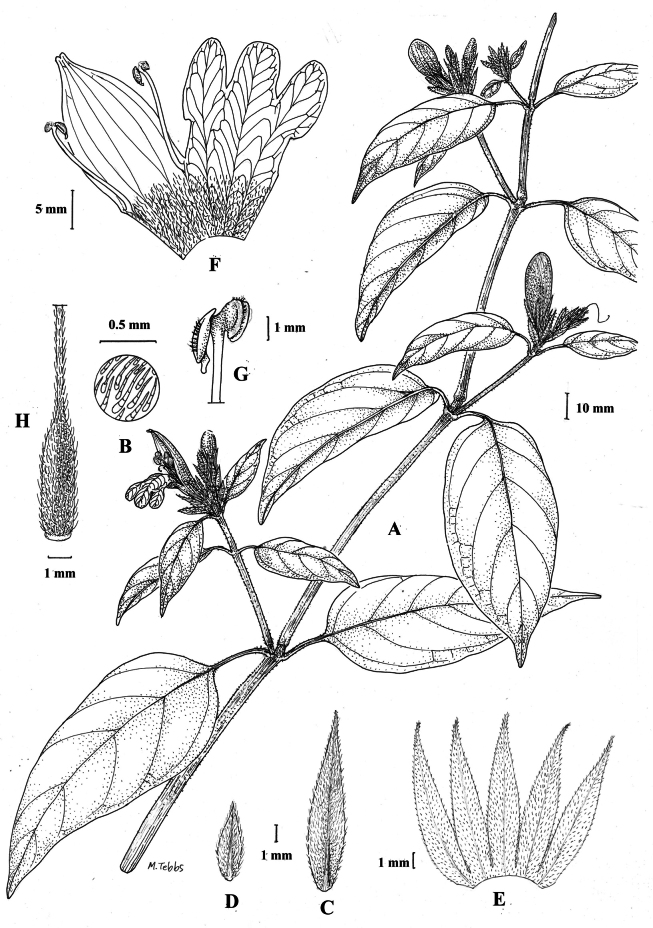
*Justicialactiflora***A** habit **B** detail of bract indumentum **C** bract **D** bracteole **E** calyx **F** corolla **G** anther **H** ovary and style base. Drawn from *J. Schunke Vigo & J.G. Graham* 16107 by Margaret Tebbs.

##### Etymology.

The epithet “*lactiflora*” refers to the distinctive milk-white corollas of this species.

##### Phenology.

Found in flower principally between August and October with a few sporadic records from other months.

##### Habitat.

Primary tropical forest on alluvial soil, often in scrub along river banks, 100–470 (–1000) m.

##### Distribution.

Endemic Amazonian Peru and restricted to Loreto and Ucayali. Fig. 68.

##### Material examined.

**Peru. Loreto**: Prov. Maynas, Punchana near Iquitos, 100 m, 7 April 1948, *R. Ferreyra & H.J. Corner* 3346 (MOL, US, USM); • ibid., Dist. Iquitos, 100 m, Aug. 1929, *E.P. Killip & A.C. Smith* 27471 (F, US); • ibid., Explorer’s Inn, near Indiana, Río Amazon below Iquitos, 130 m, 3°30'S, 73°3'W, 15 Feb. 1989, *Al. Gentry et al.* 65850 (MO). **Ucayali**: Prov. Coronel Portillo [General Portillo], entre Sinchono y Boquerón, hacia Pucallpa, 1000–1100 m, 15 Aug. 1946, *R. Ferreyra* 1107 (US, USM). Prov. Padre Abad, the type collection; • ibid., Dist. Padre Abad, Aguaytía, 180 m, 26 Aug. 1946, *F. Woytkowski* 34443 (F); • ibid., Pampa Yurac, 300 m, 9 Sept. 2004, *J. Schunke V. & J.G. Graham* 15882 (MO, US); • ibid., Aguaytía, 9°05'S, 75°32'W, 28 Sept. 2004, *J. Schunke V. & J.G. Graham* 16107 (F, MOL, US); • ibid., Previsto, Río Yurac, 420 m, 16 Oct. 1962, *F. Woytkowski* 7610 (US, MO); • ibid., Boquerón de Padre Abad, along Río Chino, 300 m, 8 Aug. 1946, *F. Woytkowski* 34339 (F, MO); • ibid., 470 m, 8 Aug. 1946, *F. Woytkowski* s.n. (USM 72643).

##### Note.

The corolla is variously reported as white or white with various types of purple markings, but it is unclear whether this is because the corolla varies in colour from plant to plant or at various stages of its development.

#### ﻿﻿40. Justicia bambusiformis

Taxon classificationPlantaeLamialesAcanthaceae

J.R.I.Wood & R, Villanueva sp. nov.

C76F894B-836C-525F-AC2C-9F3EFBF69AE9

urn:lsid:ipni.org:names:77363431-1

##### Type.

Peru • Cusco, Prov. La Convención, Dist. Echarate, Llactahuaman, N del Río Apurímac, NE of Pueblo Libre, 12°51'55.5"S, 73°30'40"W 1650 m, 14 July 1998, *S. Baldeón et al.* 3215 (holotype US-3379708, isotype USM).

##### Diagnosis.

Distinctive species because of its tall, stout, subcylindrical stems, the flowers in short elongate cymose structures, mostly 2–4 cm in length, arranged in verticels in the axis of the leaves, the bracts reduced to scales.

##### Description.

Subshrub 1.5–4 m high; stems erect or ascending, bamboo-like in form, slender, pubescent with crisped, multicellular hairs. Leaves petiolate, lamina 10–17 × 2–4.5 cm, narrowly oblong-elliptic, apex acuminate, base attenuate, dark green, adaxially glabrous to very thinly pubescent with scattered hairs, abaxially glabrous apart from puberulent midveins; petioles 5–8 mm, puberulent. Inflorescence a long leafy raceme, at least 25 cm in length, possibly much more; flowers in slender elongate cymose structures, mostly 2–4 cm in length, several arising in the axils at each node; rhachis shortly but densely pubescent; bracts scale-like, c. 0.5 mm long; bracteoles linear, 2.5 × 0.5 mm; calyx subequally 5-lobed to base, lobes 3 × 0.5 mm, ciliolate, puberulent; corolla 15–23 mm long, creamy-white/yellow, puberulent on exterior, cylindrical, 2-lipped, upper lip 8 mm long, notched, lower lip shallowly 3-lobed, the lobes c. 1 mm long, oblong, rounded; filaments glabrous, anther thecae weakly superposed, 1.25 mm long, upper apiculate at base, lower with a basal appendage; pollen prolate-perprolate, 38–40 × 19–20 μm, 3-aperturate, colporate, 1 row of c. 5–8 insulae, (some coalescing) on either side of aperture (Fig. 52A); ovary oblong, acute to rostrate, 2.5–3 mm, glabrous, style puberulent, 20 mm long. Capsule 15 × 3 mm, clavate, glabrous, 4-seeded; seeds 2 × 2 mm, glabrous, lenticular.

##### Illustration.

Fig. 40.

**Figure 40.**
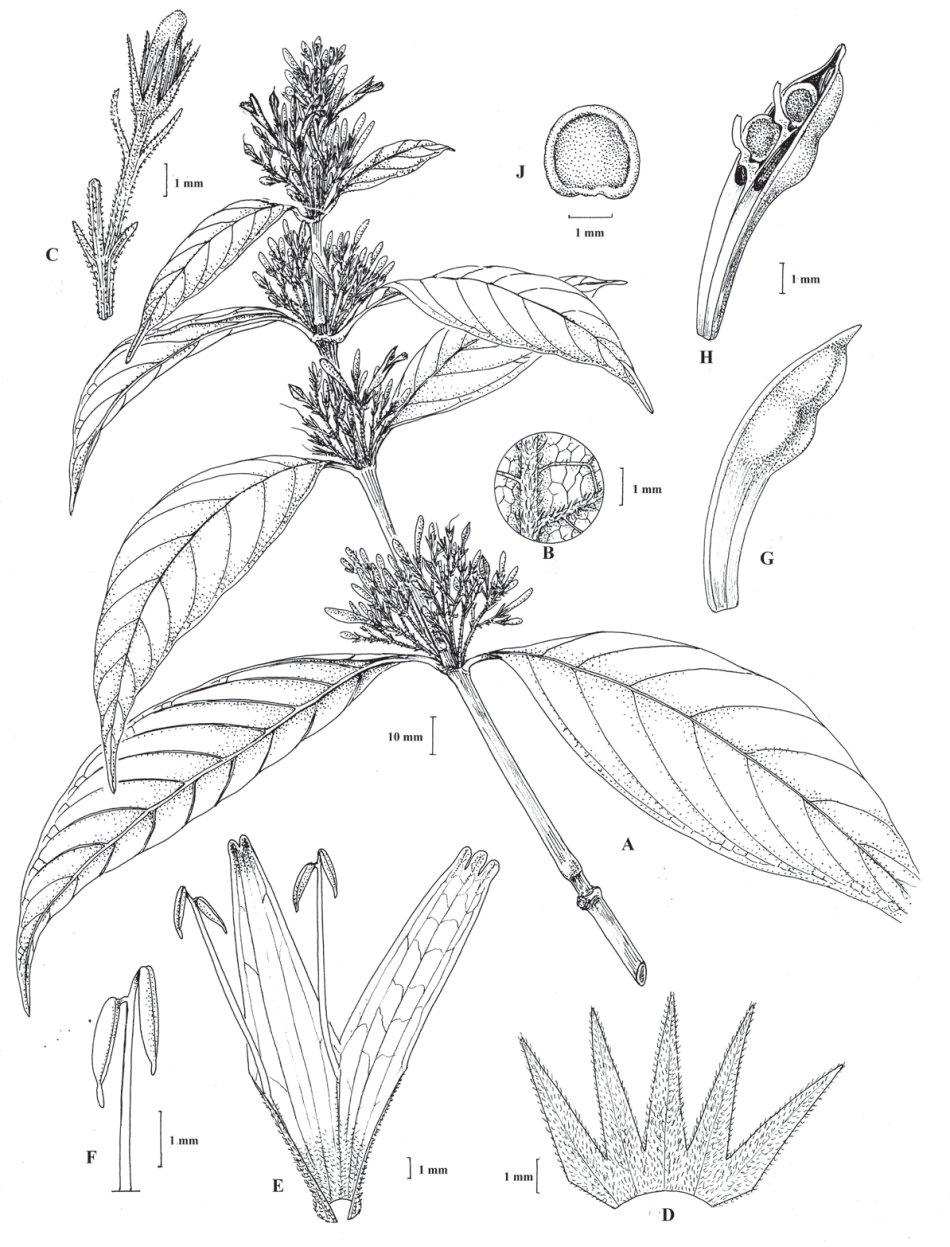
*Justiciabambusiformis***A** habit **B** detail of abaxial surface of leaf **C** portion of inflorescence showing bracteole **D** calyx **E** corolla **F** anther **G** exterior of capsule valve **H** interior of capsule valve showing seed **J** seed. **A, F** drawn from *Dudley* 10440 **G, J** from *Baldeon* 3215 by Margaret Tebbs.

##### Etymology.

The epithet “*bambusiformis*” refers to the distinctive habit of the species with its stout, subcylindrical stems and pseudoverticels of flowers in the leaf axils, recalling the habit of cloud forest bamboos of the genus *Chusquea* Kunth.

##### Phenology.

Found in flower in June and July.

##### Habitat.

Ceja de monte. Edge of bamboo thickets in cloud forest, 1650–1700 m.

##### Distribution.

Endemic to Cusco, La Convención. Fig. 69.

##### Material examined.

**Peru** • **Cusco**: La Convención, 12°38'S, 73°36'W, 1700 m, 25 June 1968, *T.R. Dudley* 10440 (NA, US); • ibid., Dist. Echarate, the type; • ibid., Echarate, Katarompanaki, 12°11'12"S, 72°28'13"W, 1800 m, *N. Salinas et al.* 6840 (USM).

#### ﻿﻿41. Justicia valenzuelae

Taxon classificationPlantaeLamialesAcanthaceae

J.R.I.Wood & R.Villanueva sp. nov.

26BB1206-28EA-5358-81B1-FADAFF8781B3

urn:lsid:ipni.org:names:77363432-1

##### Type.

Peru • Cusco, [Prov. La Convención], Dist. Vilcabamba, Oyara, Cedropata, 13°04'37"S, 72°49'09"W, 2133 m, 19 Feb. 2007, *L. Valenzuela et al.* 8722 (holotype FHO-00141081, isotype MO).

##### Diagnosis.

The inflorescence is somewhat similar to that of *Justiciacolorata* (Nees) Wassh. from Ecuador but the new species is distinguished by the yellow or cream corolla 2.3–2.7 cm long (not red or purple, 3.7–4.5 cm long) and shorter calyx up to 10 mm long (not 12–15 mm long).

##### Description.

Subshrub 0.4–1 m high, stems woody below, sulcate, bifariously pubescent with crisped, dark reddish hairs. Leaves petiolate, lamina somewhat oblique, 2.5–17 × 1–7.5 cm, quite variable in size on the same branch, narrowly elliptic, acuminate at apex and base, margin crenulate to subentire, somewhat hirsute with reddish multicellular hairs when young, especially on the margins and abaxial veins, ± glabrescent, lateral veins relatively prominent, 7–10 pairs; petioles 0.5–5.5 cm, bifariously hirsute, glabrescent. Inflorescence of lax, branched, cymose structures from the upper leaf axils, these up to 12 cm long; peduncles (1–)2.6–5 cm; bracts at inflorescence branching points resembling minute leaves, 0.5–1(–2.7) cm long, somewhat deciduous; secondary peduncles 0.5–3 cm; rhachis 0–3 (–6) cm long; flowers mostly alternate, borne on short persistent pedicels 0.5–1 mm long; floral bracts narrowly oblanceolate, 3 × 1 mm, hirtellous; bracteoles similar, 2 × 1 mm, linear, hirtellous, caducous; calyx subequally 5-lobed to base, lobes 7–10 × 1 mm, lanceolate, acuminate, hirtellous; corolla 2.3–2.7 cm long, yellow, hirsute in bud, tube c. 1.2 cm long, upper lip c. 13 mm, notched, lower lip c. 1.4 cm long, shallowly 3-lobed; filaments glabrous, yellow; anthers purple, thecae c. 2 × 0.75 mm, linear, parallel, glabrous, very weakly superposed, lower with a small basal appendage; pollen prolate-perprolate, 46–50 × 22–25 μm, 3-aperturate, colporate, 1 row of c. 7–9 distinct insulae on either side of aperture (Fig. 52B); ovary and style glabrous. Capsule and seed not seen.

##### Illustration.

Fig. 41.

**Figure 41.**
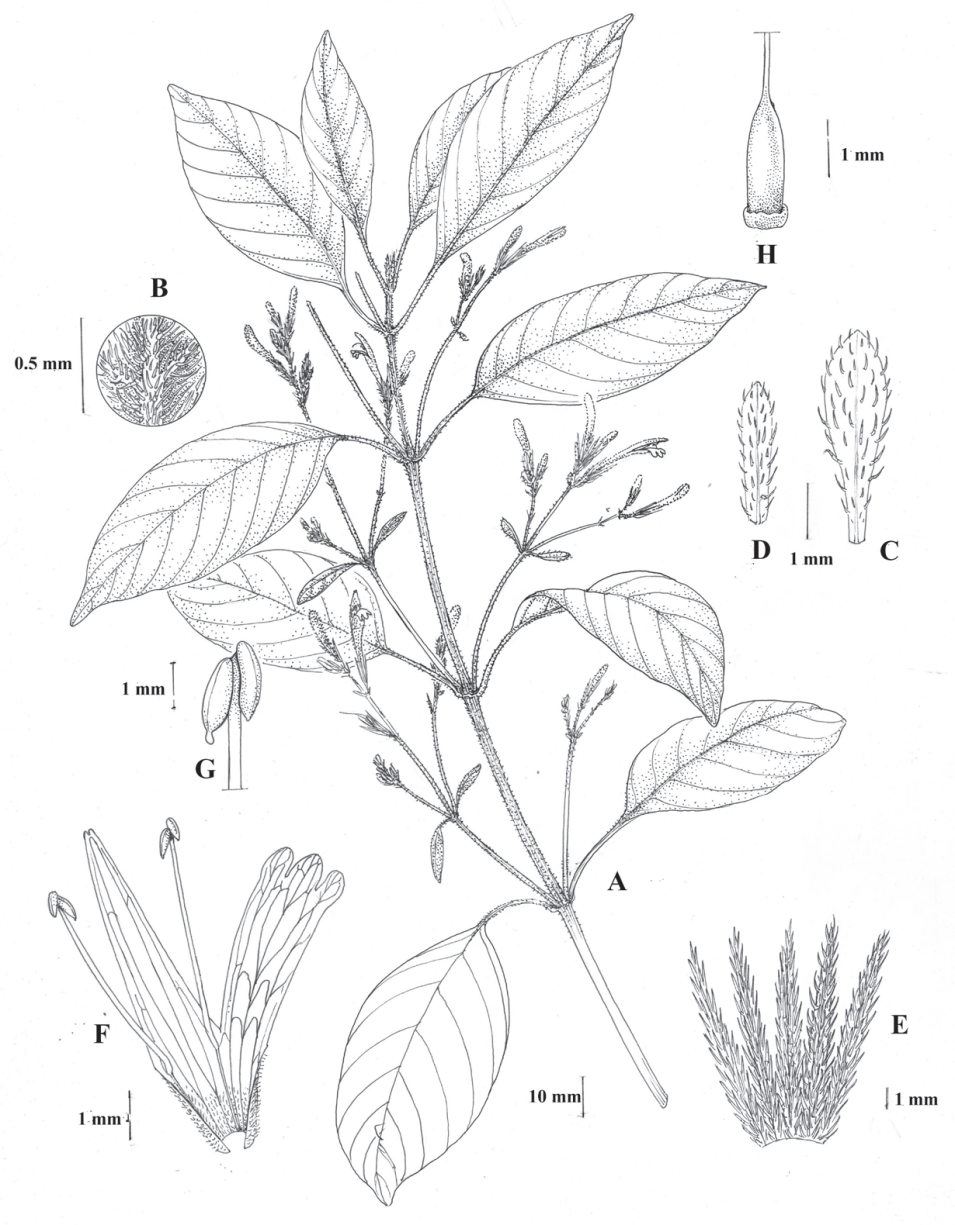
*Justiciavalenzuelae***A** habit **B** detail of indumentum of abaxial leaf surface **C** bract **D** bracteole **E** calyx **F** corolla **G** anther **H** ovary and style base. Drawn from *Valenzuela* 8722 by Margaret Tebbs

##### Etymology.

The epithet “*valenzuelae*” commemorates Luis Valenzuela, important Peruvian botanist and plant collector, who made nearly all collections of this species.

##### Phenology.

Found in flower from October to April.

##### Habitat.

Humid montane subtropical forest, 2133–2950(–3300) m.

##### Distribution.

Endemic to southern Peru. Fig. 69.

##### Material examined.

**Peru** • **Cusco**: Prov. La Convención, Dist., Santa Teresa, Yanatile, 13°05'S, 72°23'W, 2950 m, 16 April 2005, *L. Valenzuela et al.* 5371 (FHO, MO); • ibid., Dist. Vilcabamba, the type; • ibid., Dist. Huayopata, Pistipata drainage, 10 km SW of Incatambo, 2380 m, 5 Oct. 1982, *B. Peyton & S. King* 1417 (MO, US); • ibid., Incatambo, quebrada Curcur, 13°04'07"S, 72°27'05"W, 2477 m, 23 Nov. 2007, *L. Valenzuela et al.* 4521 (CUZ); • ibid., 13°04'07"S, 72°26'49"W, 2630 m, 24 April 2007, *L. Valenzuela et al.* 9554 (CUZ); • ibid., Espiritupampa, 13°03'S, 73°05'W, 3300 m, 15 Oct 2003, *E. Suclli et al.* 1318 (US); ibid, Dist. Santa Ana, Poromate, 12°56'57"S, 72°47'20"W, 2302 m, 17 June 2003, *G. Calatayud et al.* 1504 (CUZ); • ibid., Dist. Santa Ana, Tunquimayo, 12°54'47"S, 72°49'34"W, 2367 m, 25 Sept. 2004, *G. Calatayud et al.* 2890 (FHO, MO); • ibid., Dist. Maranura, 12°52'01"S, 72°32'46"W, 2200 m, 15 April 2004, *G. Calatayud et al.* 2182 (CUZ); • ibid., Dist. Santa Teresa, subida a Yerbabuenayoc, Rocotol, 13°04'S, 72°22'W, 2420 m, *I. Huamantupa et al.* 0712 (FHO, MO); • ibid., Balconpata, 12°52'01"S, 72°32'46"W, 2200 m 15 April 2004, *G. Calatayud et al.* 2182 (FHO, MO). Prov. Urubamba, Dist. Machu Picchu, Alcamayo, 13°09'59"S, 72°30'57"W, 2200–2500 m, 23 Feb. 2003, *L. Valenzuela et al.* 1482 (CUZ); • ibid., 13°09'25"S, 72°30'58"W, 2600 m, 15 May 2003, *I. Huamantupa et al.* 3085 (CUZ).

##### Note.

Specimens of this species have been identified as *Stenostephanus* sp. but the anthers are bithecous. The flowers are variously reported as yellow or white (*Valenzuela et al.* 3014 as red). The leaves may be entire or crenate.

#### ﻿﻿42. Justicia huallagensis

Taxon classificationPlantaeLamialesAcanthaceae

R.Villanueva & J.R.I.Wood sp. nov.

D71EA267-1158-5F8A-B8A1-11596FF62B36

urn:lsid:ipni.org:names:77363433-1

##### Type.

Peru • San Martin, Prov. San Martin, 15 km E of Shapaja on road to Chazuta, Quebrada Chumia, near Mal Paso, Chumia on Río Huallaga, 6°36'S, 76°10'W, 250–300 m, 4 Aug. 1986, *S. Knapp* 7879 (holotype MO-3518887, isotypes F-1992309, US-3082657).

##### Diagnosis.

A new species resembling *Justiciamalacophylla* Leonard in having the inflorescence formed of long axillary and terminal spikes, mostly > 8 cm long, and in the persistent linear or lanceolate bracts, but differing in the glabrous leaves (not abaxially pubescent), longer, linear bracts 12–22 mm long with recurved tips (not 5–7 mm long, lanceolate with erect tips) and a white corolla 5–5.5 cm long (not red, 3.5–4 cm long).

##### Description.

Shrub to 1.5 m high; stems somewhat sulcate, glabrous. Leaves petiolate, equal or unequal in each pair, lamina 6–27 × 2.5–10 cm, broadly oblong-elliptic, apex acuminate, base attenuate, both surfaces glabrous, lateral veins 13–15 pairs, adaxially with abundant small cystoliths, abaxially paler; petioles 0.4–5 cm, glabrous. Inflorescence of terminal and axillary, spikes arising from the upper leaf axils, the terminal spikes up to 14 cm long, the axillary spikes shorter, the lowermost apparently infertile, flowers up to 15 mm apart, mostly in opposite pairs; peduncles 1.5–3.4 cm, glabrous; bracts at base of terminal inflorescence, foliose, petiolate, c. 1.5 × 0.5 cm; rachis glabrous; floral bracts linear, acute, 15–20 × 1 mm, puberulent; bracteoles similar but shorter and narrower, c. 12–14 × 0.5–1 mm; calyx 5-lobed to near base, lobes 14–16 × 1 mm, linear, acuminate, minutely puberulent; corolla 5–5.5 cm long, white, pubescent on the exterior, 2-lipped, tube 40 × 3 mm; upper lip c. 15 mm long, notched, lower lip slightly longer, shallowly 3-lobed, lobes c. 1.5 mm long, the laterals ovate, the central rounded; filaments with a few scattered hairs, anthers included, thecae c. 2.5 × 0.75 mm, oblong, both with a short basal appendage, parallel, superposed, glabrous; pollen prolate, 33–35 × 23–25 μm, 2-aperturate, colporate, 1–2 rows of indistinct insulae either side of the aperture (Fig. 52C); style glabrous. Capsule 17–18 × 2.5 cm, weakly clavate, puberulent, 4-seeded; seeds rounded, c. 2.25 mm dia., rugose.

##### Illustration.

Fig. 42.

**Figure 42.**
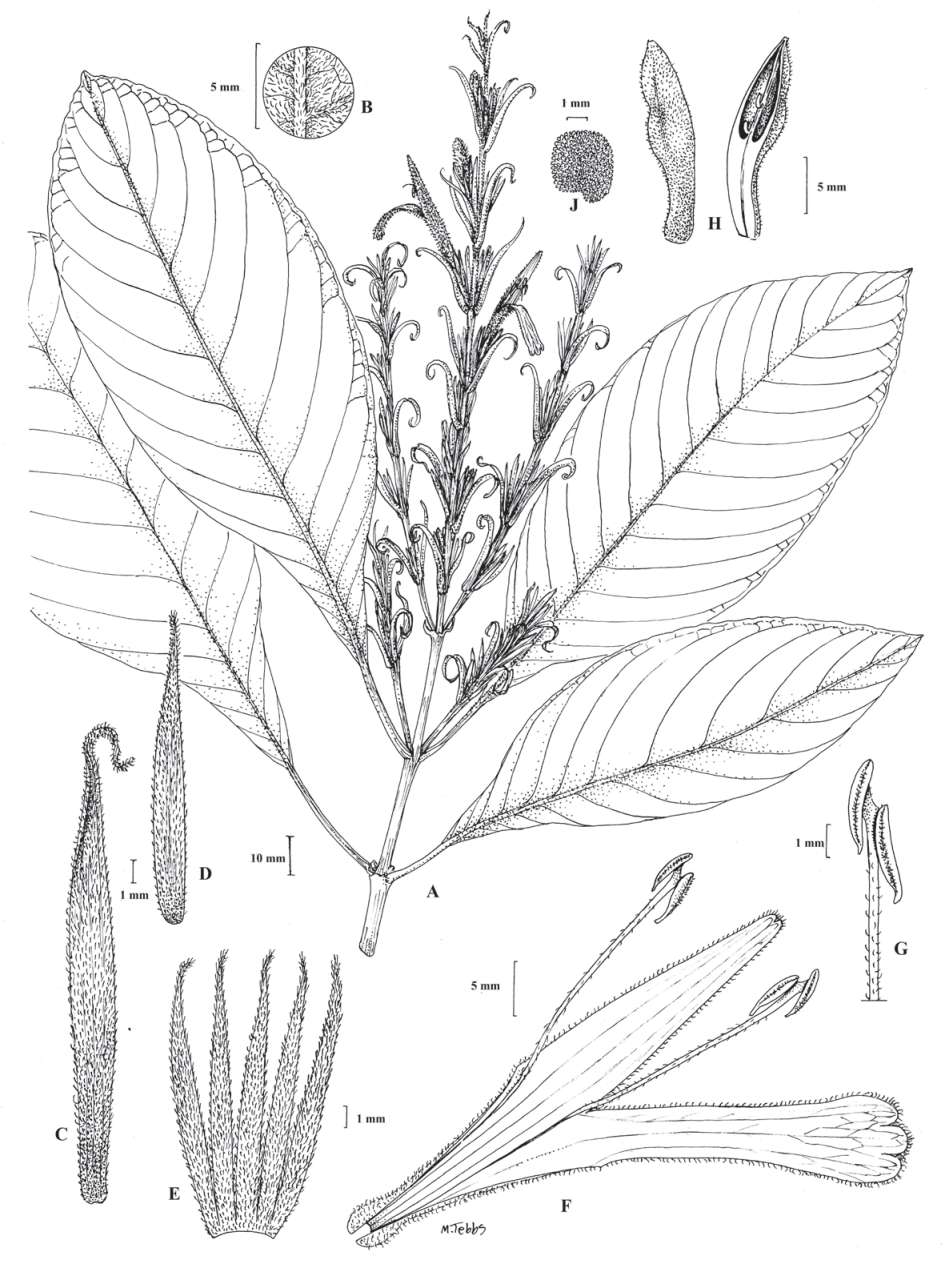
*Justiciahuallagensis***A** habit **B** detail of abaxial surface of the leaf **C** bract **D** bracteole **E** calyx **F** corolla opened out to show stamens **G** anther **H** capsule, exterior (left), interior (right), **J** seed. Drawn from *Knapp* 7879 by Margaret Tebbs

##### Etymology.

The epithet “*huallagensis*” refers to the Río Huallaga, whose valley runs through San Martin and is the site of many endemic species, including several species of *Justicia*.

##### Phenology.

Found in flower at the beginning of August.

##### Habitat.

Humid lowland tropical forest along a stream, 300 m.

##### Distribution.

Endemic to Peru and only known from the type locality in San Martin. Fig. 68.

##### Material examined.

**Peru** • **San Martin**: Type collection.

##### Note.

The bracts are longer than the calyx and bracteoles and often become twisted, giving the inflorescence an untidy, “whiskery” appearance. The white corolla is also notably long. The relationships of this species are not obvious.

#### ﻿﻿43. Justicia oxapampensis

Taxon classificationPlantaeLamialesAcanthaceae

R.Villanueva & J.R.I.Wood, sp nov.

9B47CD42-A97F-5A4D-BD3D-EF72129F0067

urn:lsid:ipni.org:names:77363434-1

##### Type.

Peru • Pasco, Prov. Oxapampa, Dist. Palcazú, P.N. Yanachaga-Chemillén, Estación Biológica Paujil, 10°19'26"S, 75°15'50"W, 399 m, 10 Aug. 2014, *R. Vásquez* 39073 (holotype MO-6720846, isotype USM).

##### Diagnosis.

A new species with no obvious affinity to other Peruvian species but superficially resembling *Justiciaholgueri* Wassh. and *J.balslevii* Wassh. from Ecuador in the lax subterminal spikes with near white corollas, but distinguished from both by the larger corollas, c. 19 mm long (not 11–12 mm) and the distinctive broadly oblong-elliptic, slightly oblique, pilose leaves up to 8 cm wide (not subglabrous, < 4 cm wide).

##### Description.

Erect herb 50–60 cm high; stems hirsute with patent white, large-celled hairs mixed with shorter curled gland-tipped hairs. Leaves petiolate, lamina 6–16 × 3–8 cm, broadly oblong-elliptic, apex acuminate, base somewhat oblique, broadly to narrowly cuneate and decurrent on the petiole, margin entire to obscurely crenulate, cystoliths prominent, adaxially thinly pilose, abaxially pilose, more densely so on veins, lateral veins 7–8 pairs; petioles 2.5–5 cm, pilose. Inflorescence of lax pedunculate spikes 4–7 cm long, terminal or arising from the upper leaf axils, pilose with gland-tipped hairs; peduncles 1–6 cm, pilose; flowers in opposite pairs, 3–10 mm apart; bracts at base of spike 2–4 cm long, resembling reduced leaves, floral bracts 9–10 × 1.3 mm, narrowly oblong or oblanceolate, glandular-pubescent; bracteoles 4–5 × 0.75 mm; calyx subequally 5-lobed, lobes 7 × 1 mm, lanceolate, pilose with gland-tipped hairs; corolla c. 1.9 cm long, white with pale blue lower lip, glandular-puberulent, tube 10 mm long, scarcely widened upwards to 2 mm, upper lip 5 mm long, ovate, obtuse, minutely notched, lower lip with pale blue “herring bone” patterning, 3-lobed, central lobe suborbicular, rounded, laterals oblong; anther thecae oblong 1.25 × 0.5 mm, superposed, the connective stout, lower theca with a white appendage; pollen c. 32 × 18 μm, 2-aperturate, colporate, 1 row of c. 7 indistinct insulae on either side of aperture (Fig. 52D). Capsule 16 × 4 mm, clavate, pilose with gland-tipped hairs, 4-seeded; seeds lenticular, 2.5 × 2 mm, brown with pale margin, scabrous.

##### Illustration.

Figs 43, 44.

**Figure 43.**
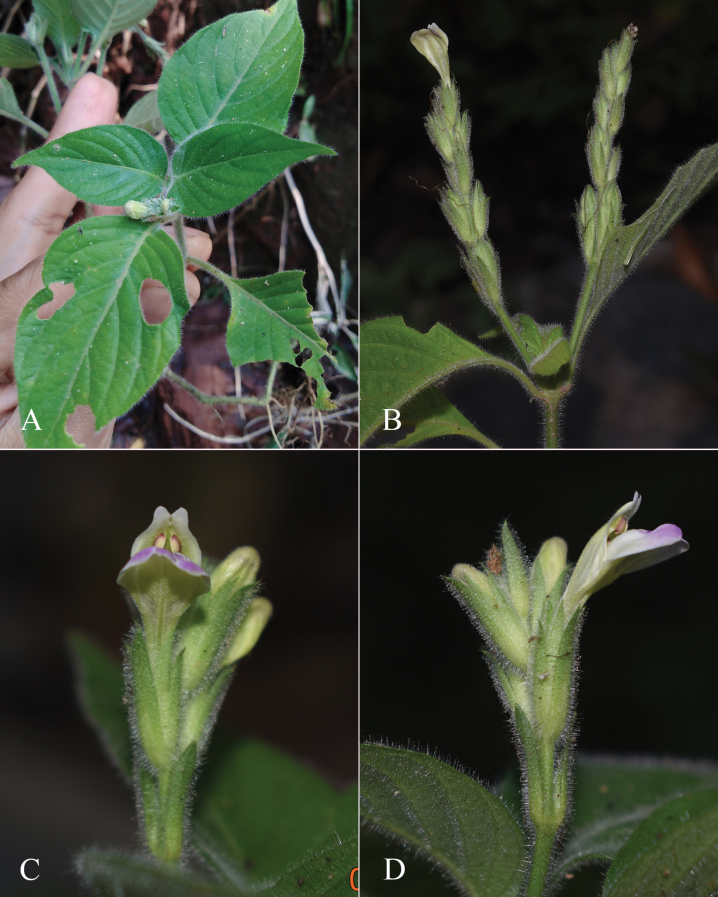
Photographs of *Justiciaoxapampensis***A** Igor Azevedo **B, D** (*Villanueva* 963) Rosa Villanueva

**Figure 44.**
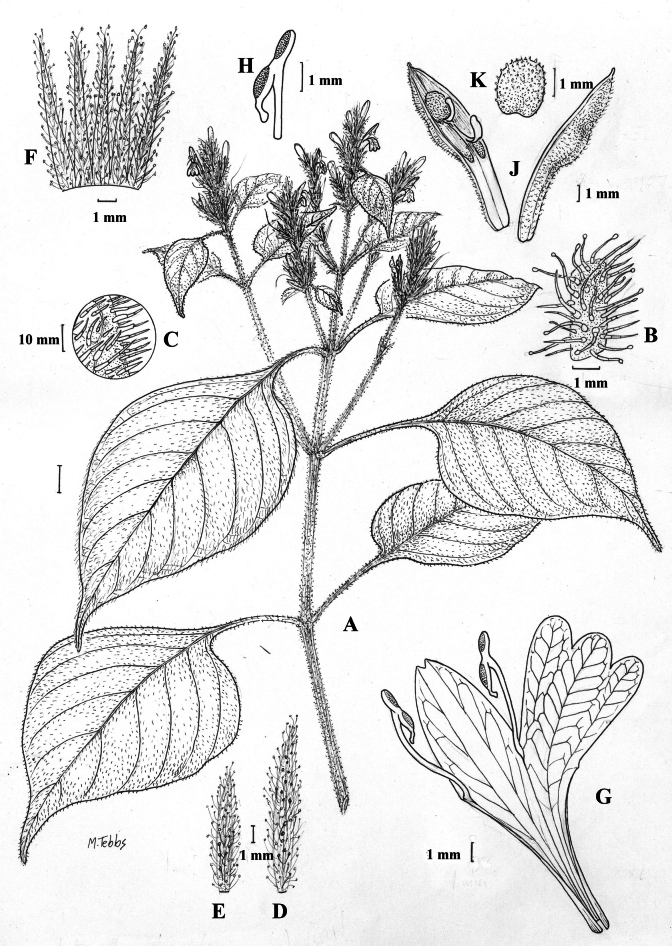
*Justiciaoxapampensis***A** habit **B** cross section of stem showing trichomes **C** leaf margin showing trichomes **D** bract **E** bracteole **F** calyx **G** corolla opened out showing androecium **H** anther **I** capsule valve (interior) **J** capsule valve (exterior) **K** seed. **A, H** drawn from *Van der Werff* 18236 **I, K** from *Vásquez* 39073 by Margaret Tebbs.

##### Etymology.

This species is one of several new taxa described from the Oxapampa region of Pasco in central Peru. Oxapampa is commemorated in this name because it seems to be a hotspot for endemic species of *Justicia*.

##### Phenology.

Flowers mainly in the winter dry season from May to August, with one isolated record from January.

##### Habitat.

Forested slopes, 380–1600 m.

##### Distribution.

Endemic to the Oxapampa area in Junín, Peru. Fig. 68.

##### Material examined.

**Peru** • **Pasco/Junín**: Pichis trail, Yapas [10°19'53"S, 74°53'28"W approx.], 1350–1600 m, 28–29 June 1929, *E.P. Killip & A.C. Smith* 25571 (US). • **Pasco**: Prov. Oxapampa, along road Chatarra–Cacazu, 10°32'S, 75°04'W, 890 m, 10 July 2003, *H. Van der Werff* 18236 (HOXA, MO, US); • ibid., Dist. Oxapampa, Comunidad Nativa Alto Lagarto-Convento (Reserva Comunal Yanesha), 10°08'04"S, 75°22'06"W, 500 m, 30 June 2014, *R. Rojas & G. Ortiz* 9280 (HOXA); • ibid., Dist. Palcazú, Iscozacin, 10°12'S, 75°15'W, 380 m, 11 Jan. 1984, *R. Foster et al.* 7872 (US, MO, USM); • ibid., Com Nativa Santa Rosa de Chuchurras, 10°08'24"S, 75°13'05"W, 27 Aug. 2007, *L. Hernan* 0300 (USM); • ibid., P.N. Yanachaga-Chemillén, Est. Biol. Paujil 10°19'26"S, 75°15'50"W, 399 m, 10 Aug. 2014, *R. Vásquez* 39073 (MO, USM); ibid, Dist. Villa Rica, camino Bella Esperanza–Sares, 10°32'23"S, 75°04'19"W 696 m, 26 July 2010, *R. Vásquez et al.* 36722 (HOXA, USM). • **Huánuco**: Prov. Puerto Inca, Dist. Codo de Pozuzo, Pozuzo–Codo road, 9°40'27"S, 75°27'43"W, 395 m, 7 Aug. 2023, *R. Villanueva et al.* 963 (HOXA); • ibid., 28 Aug. 2019, *I. Azevedo & R. Villanueva* 156 (HOXA).

##### Note.

Once noticed the leaf shape and unusual leaf base combined with the relatively long petioles are very distinctive.

#### ﻿﻿44. Justicia falcifolia

Taxon classificationPlantaeLamialesAcanthaceae

J.R.I.Wood & R.Villanueva sp. nov.

E4F23771-E2CE-5CE0-B7B9-DA7F0EAA0FCA

urn:lsid:ipni.org:names:77363435-1

##### Type.

Peru • Pasco, Prov. Oxapampa, Dist. Villa Rica, Antiguo camino hacia Cacazú, Centro Poblado Palma, zona de Amortiguamiento, P. N. Yanachaga-Chemillén, 10°39'57"S, 75°10'41"W, 1760–1850 m, 29 May 2005, *E.M. Ortiz* 660 (holotype MO-6409873, isotypes MOL, HOXA, US, USM).

##### Diagnosis.

Bears a superficial resemblance to *Justiciasquarrosa* Griseb. in the size and shape of the leaves and the size, shape and colour of the corolla, but leaves reticulate beneath, the inflorescence elongate, clearly spicate (not subcapitulate) and the bracts and bracteoles linear-lanceolate, 8–9 mm long, straight (not filiform, mostly 10–20 mm long, and curled).

##### Description.

Liana to 5 m in height; stems roughly hirsute, glabrescent when old and woody. Leaves shortly petiolate, lamina somewhat coriaceous, 3–9.5 × 1–3 cm, ovate, apex long-acuminate and strongly falcate, base rounded, somewhat oblique, margin obscurely crenulate, both surfaces roughly pilose with scattered hairs, adaxially weakly bullate, with cystoliths, abaxially somewhat reticulate with raised venation, lateral veins 6–7 pairs; petioles 0.3–1 cm, pubescent. Inflorescence of dense, shortly pedunculate axillary and terminal spikes 2–6 cm long; flowers imbricate; peduncles 0.4–1 cm, villous; bracts 8–9 (–12) × 1–1.25 mm, linear-lanceolate, pilose; bracteoles similar but smaller, 7 × 1 mm; calyx subequally 5-lobed, lobes 9–11 × 0.75 mm, very narrowly linear-lanceolate, pilose with gland-tipped hairs; corolla c. 2.5–2.7 cm long, deep pink, glandular-pilose on the exterior, basal tube 12–15 mm long, slightly widened upwards from c. 1 mm at base, upper lip c. 12 mm long, notched, lower lip, shallowly 3-lobed, lobes ovate, rounded, 1–2 mm long and wide; filaments 10–11 mm long, glabrous, anther thecae elliptic, glabrous, superposed, the upper theca 1 × 0.75 mm, the lower half the size; pollen prolate, 33–34 × 21–22 μm, 2-aperturate, colporate, 1 row of c. 7–8 distinct insulae on either side of aperture (Fig. 52E); style glabrous, c. 2.5 cm long. Capsule 9 × 2 mm, clavate, pubescent, 4-seeded; seeds 2 × 1.5 mm rugose.

##### Illustration.

Fig. 45.

**Figure 45.**
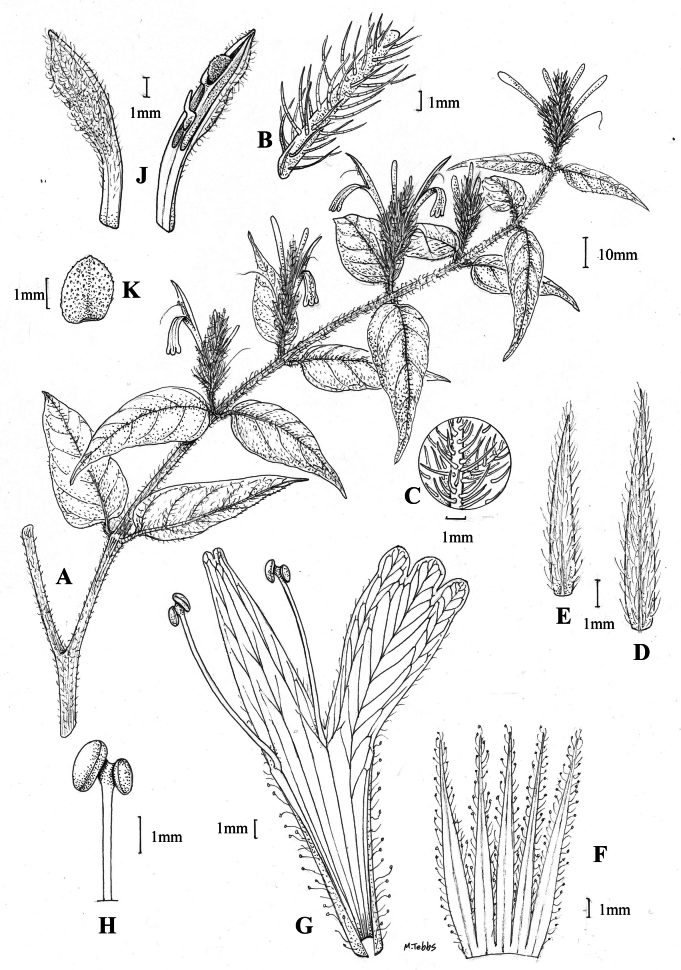
*Justiciafalcifolia***A** habit **B** detail of stem indumentum **C** abaxial surface of leaf showing indumentum **D** bract **E** bracteole **F** calyx **G** corolla opened out to show lips and androecium **H** anther **J** capsule valves, exterior (left), interior (right) K. seed. **A, H** drawn from *Ortiz* 660, **J, K** from *Killip & Smith* 25936 by Margaret Tebbs.

##### Etymology.

The epithet “*falcifolia*” refers to the distinctive falcate leaves, which are characteristic of this species.

##### Phenology.

Found in flower from March to July.

##### Habitat.

Montane forest from approximately 1500 and 2300 m. Fig. 69.

##### Distribution.

Endemic to Peru in the zone of the Parque Nacional Yanachaga-Chemillén.

##### Material examined.

• **Pasco**: Prov. Oxapampa, Dist. Villa Rica, the type collection; • ibid., Comunidad Centro Bocaz, Zona de amortiguamiento del Parque Yanachaga, 10°39'16.8"S, 75°10'39.2"W, 1300–1700 m, 12 April 2006, *S. Vilca et al.* 664 (HOXA); • ibid., Dist. Oxapampa, sector Santa Cruz, Fundo Pocras, 10°39'06"S, 75 20'36"W, 2306 m, March 2010, *Y. Ojeda & J. Vargas* 1433 (USM). • **Junín/ Pasco**: Pichis trail, Porvenir [11°36'S, 74°03'W approx], 1500–1900 m, 3–4 July 1929, *E.P. Killip & A.C. Smith* 25936 (F, US).

#### ﻿﻿45. Justicia hyalina

Taxon classificationPlantaeLamialesAcanthaceae

J.R.I.Wood & R.Villanueva sp. nov.

836B8F31-66AD-5B68-AAE0-F28C36834630

urn:lsid:ipni.org:names:77363436-1

##### Type.

Peru • Cusco, Prov. Quispicanchis, Dist. Camanti, Quincemil–Camanti, Río Tunquimao, 13°15'76"S, 70°80'78"W, 801 m, 7 Feb. 2011, *J.D. Wells, P. Centeno & M. Hammett* 1258 (holotype USM, isotype BRIT).

##### Diagnosis.

An apparently isolated species, not obviously comparable with other species of *Justicia* but distinguished by the very large oblong-elliptic leaves, by the short, rather delicate, usually paired inflorescences arising in each leaf axil, the large, pale yellow corollas and especially the linear-oblanceolate hyaline floral bracts.

##### Description.

Shrub 1–5 m high; stems bifariously scurfy-hirtellous. Leaves petiolate, lamina 13–25 × 2.8–7.3 cm, narrowly oblong-elliptic, apex acuminate to a fine point, base cuneate, margin entire, sparsely to densely ciliolate with multicellular hairs; adaxially glabrous apart from scurfy-pubescent veins towards the base, abaxially paler, the veins puberulent, sometimes when young thinly pubescent with multicellular hairs, venation somewhat prominent, the laterals 9–10 pairs; petioles 1.8–4.5 cm, scurfy-hirtellous. Inflorescence terminal, with 2–3 cymes arising from each of the uppermost leaf axils, the whole inflorescence dense and fragile in texture; cymes 5–6 cm long; primary peduncles 1–2.3 cm, crisped-pubescent; secondary peduncles and pedicels shorter but similar in indumentum; bracts linear-oblanceolate to spathulate, 8–9 × 1–2 mm, dotted with minute glands, subglabrous to sparsely ciliolate especially apically, thin and hyaline in texture; bracteoles linear, 5 × 0.75 mm; calyx lobes 13–14 × 2–3 mm, lanceolate, tapering to a fine point, subglabrous to ciliolate apically; corolla 3.5–4.5 cm long, cream to yellowish, in bud minutely puberulent on the exterior towards base, strongly 2-lipped, tube c. 1.2 cm long, upper lip 1.8 cm long, bilobed, lower lip 1.8 cm long, 3-lobed, lobes c. 3 mm long, the laterals c. 2 mm wide, the central lobe c. 3 mm wide; anther thecae superposed, narrowly oblong, c. 2 × 0.25 mm, both with a white basal appendages c. 0.5 mm long; pollen prolate-perprolate, 59–63 × 25–26 μm, 3-aperturate, colporate, insulae in 1 row on each side of the aperture (Fig. 52F); ovary oblong, glabrous, c. 1.5 × 0.5 mm; style glabrous above, strigose below. Capsule and seeds not seen.

##### Illustration.

Figs 46, 47.

**Figure 46.**
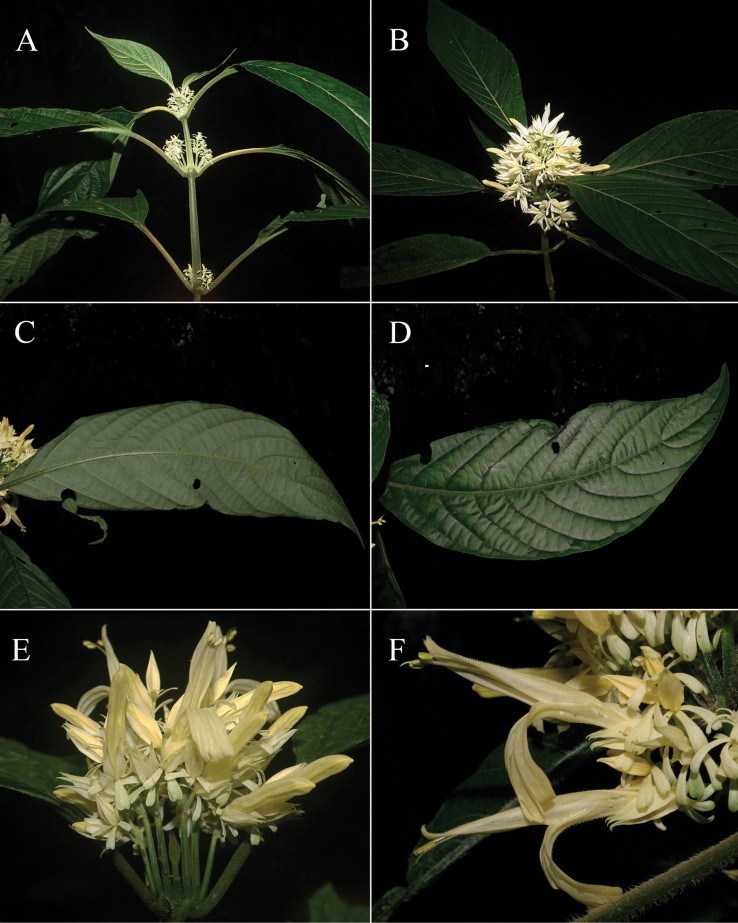
Photographs of *Justiciahyalina***A, B** (*Chambi* 79), **E** (*Chambi* 924) by B. Chambi, **C, D**, **F** (*Janovec* 3246) **A, B, E** B. Chambi; **C, D, F** J. Janovec.

**Figure 47.**
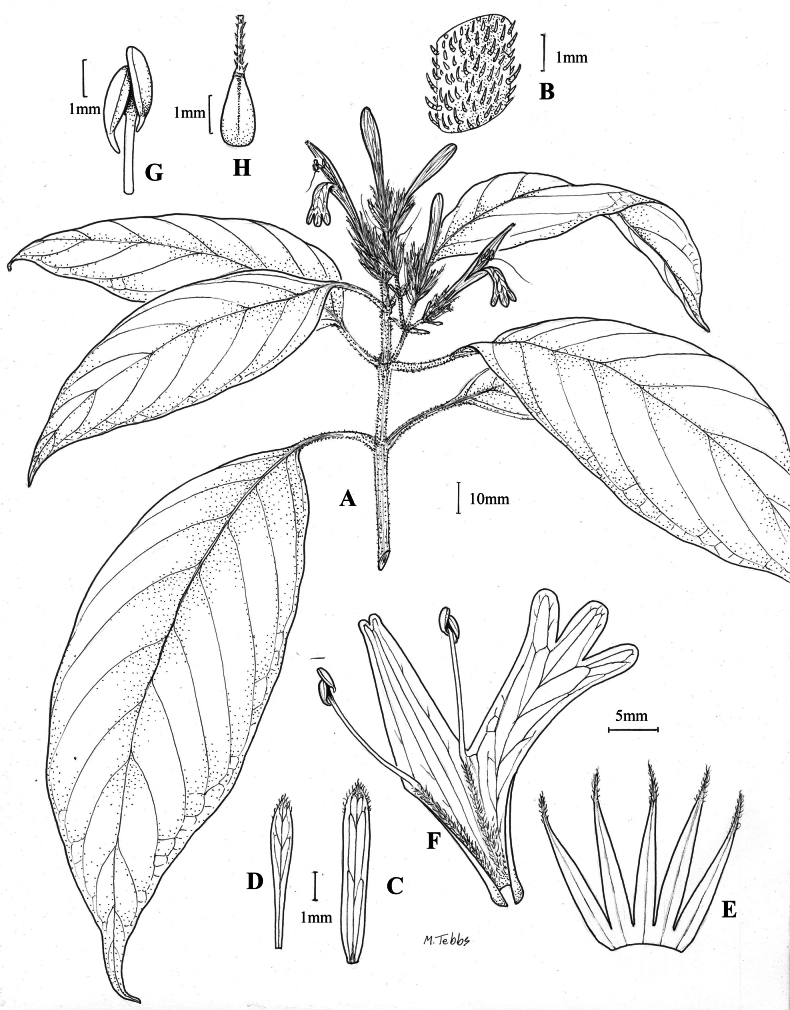
*Justiciahyalina***A** habit **B** cross section of stem **C** bract **D** bracteole **E** calyx **F** corolla opened out to show androecium **G** anther **H** ovary and base of style. **A**, **C, H** drawn from *Chambi*, *P. Centeno & J.P. Janovec* 1310 **B** from *Scolnik* 909 by Margaret Tebbs.

##### Etymology.

The epithet “*hyalina*” refers to the distinctive hyaline bracts, which are characteristic of this species.

##### Phenology.

Found in flower in February and May to August.

##### Habitat.

Low altitude humid cloud forest, 800 to 950 m.

##### Distribution.

Endemic to Peru and only known from a few locations in Cusco. Fig. 69.

##### Material examined.

**Peru** • **Cusco**: Prov. Paucartambo, Keros–Valle de Kosñipata [13°31'30"S, 71°58'20"W], ca. 950 m., 23–31 June 1948, *R. Scolnik* 909 (MO); • ibid., Dist. Kosñipata, Patria, 900 m, 5 Aug. 1951, *C. Vargas* 10235 (CUZ); • ibid., Consuelo, aguada de Asunción, 850 m, 24–30 July 1948, *C. Vargas* 07355 (CUZ). Prov. Quispicanchis, Dist. Camanti, Propiedad del sr. Bustamante, 13°13'52.32"S, 70°46'45.12"W, 600–1700 m, 19 March 2008, *B. Chambi* 797 (CAS); • ibid., propiedad privada, 13°14'09.96"S, 70°47'47.4"W, 600–1700 m, 19 March 2008, *B. Chambi* 924 (CAS); • ibid., Quincemil–Camanti, the type; • ibid., cima de la Montaña de Camanti, [13°15'28"S, 70°46'52"W], 800 m, 28 May 2008, *B.R. Chambi et al.* 1310 (BRIT); • ibid., trail to mining camp at Río Yanaurco, SE of Quincemil, km 234 Interoceanica-Transamazonica highway, 13°14'37"S, 70°48'45"W, 840–900 m, 18 May 2010, *J.L Clark et al.* 11525 (MO, US); • ibid., 1–2 km west of Quincemil, 872 m, 12 May 2010, *J.L. Clark* 11368 (CAS). • **Madre de Dios**: Prov. Manu, Dist. Madre de Dios, Pantiacolla Ridge, around and up mountain from Pantiacolla Lodge, 18 April 2006, *J.P. Janovec* 3246 (BRIT).

**Figure 48.**
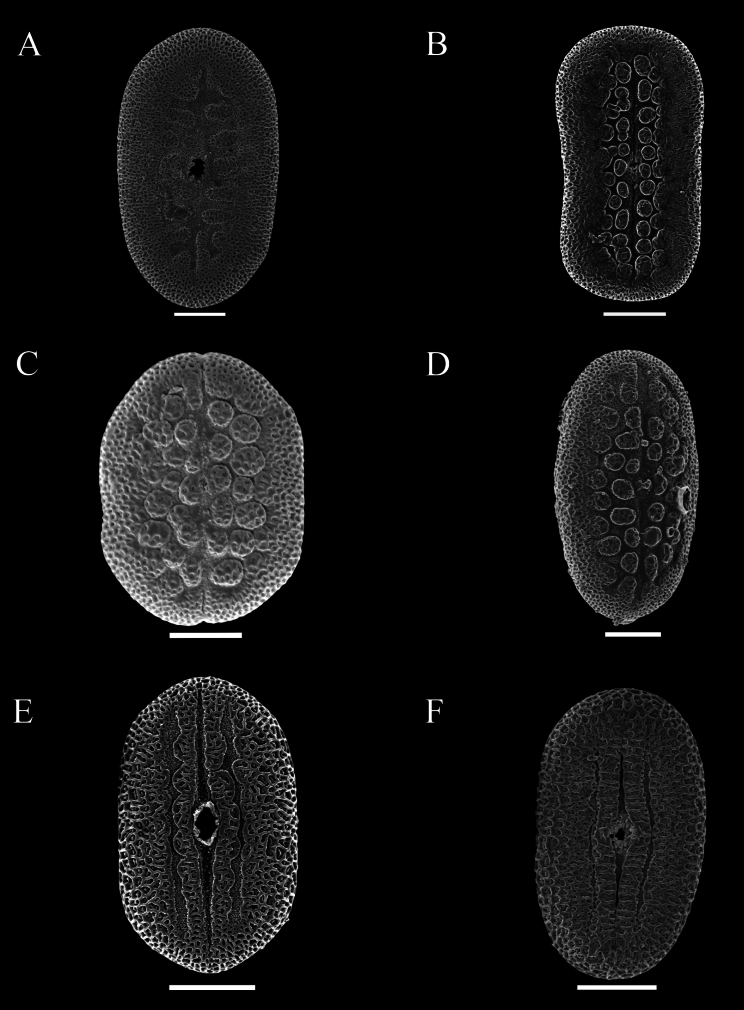
Pollen images **A**Justiciaalpinasubsp.machupicchuensis (*Stafford* 1222) **B***J.rojasiae* (*Vásquez et al*. 28272) **C***J.pozuzoensis* (*Villanueva* 925) **D***J.oppositiflora* (*Smith & Pretel* 4189) **E**J.discolorsubsp.discolor (*Woytkowski* 5824) **F**J.discolorsubsp.filisepala (*Beltrán* 5429). Scale bars: 10 μm.

**Figure 49.**
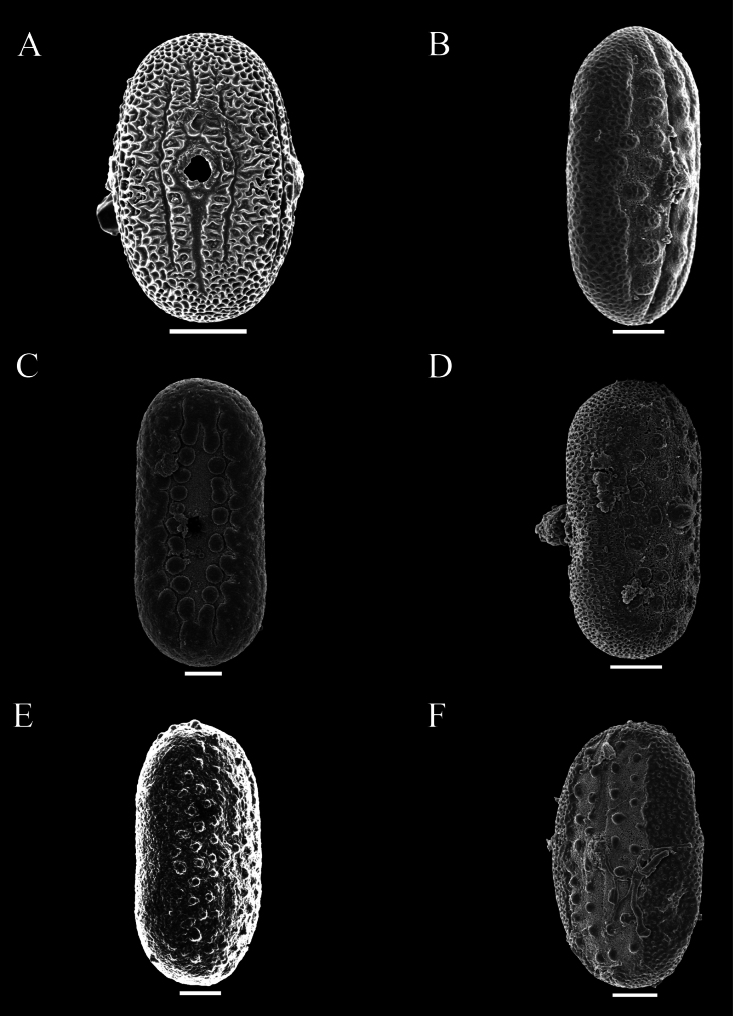
Pollen images **A***Justiciachamaecaulis* (*Encarnación* 927) **B***J.appendiculata* (*Villanueva* 916) **C***J.pelianthia* (*McDaniel* 21499) **D***J.tumbesiana* (*Diaz* 5081) **E***J.aphelandroides* (*Wurdack* 2245) **F***J.sanchezioides* (*Schunke* 12458). Scale bars: 10 μm.

**Figure 50.**
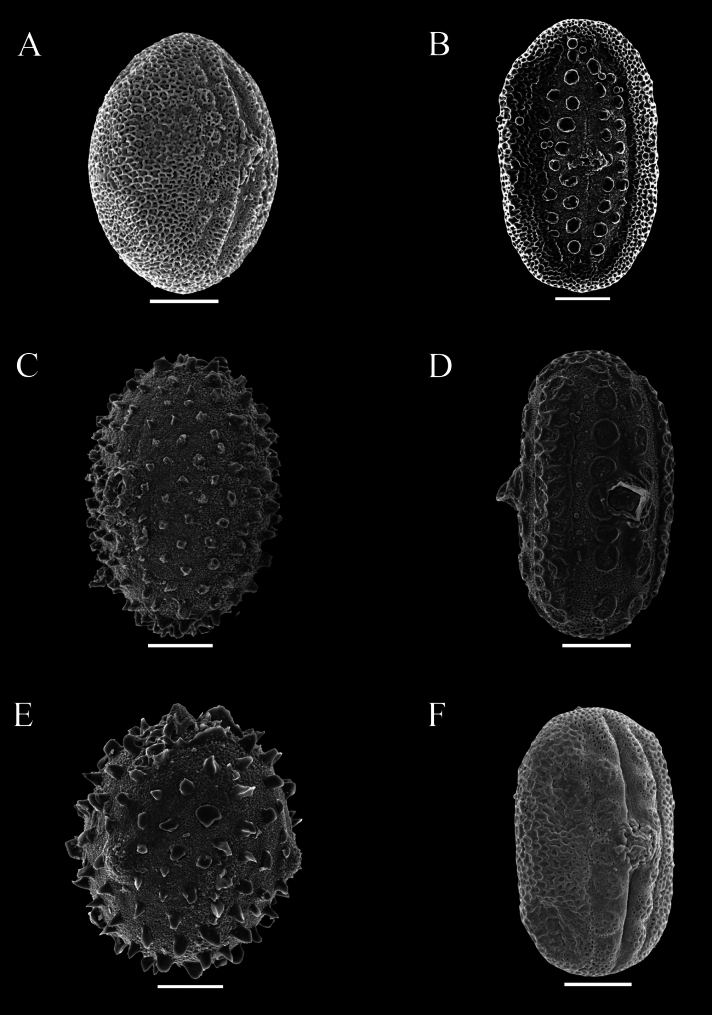
Pollen images **A***Justiciaradicans* (*Azevedo* 147) **B***J.angustituba* (*Campos & Diaz* 2380) **C***J.reginaldii* (*Ferreyra* 7070) **D***J.baguensis* (*Van der Werff* 14633) **E***J.cajamarcensis* (*Woytkowski* 5671) **F***J.tenuiflora* (*Azevedo* 281). Scale bars: 10 μm.

**Figure 51.**
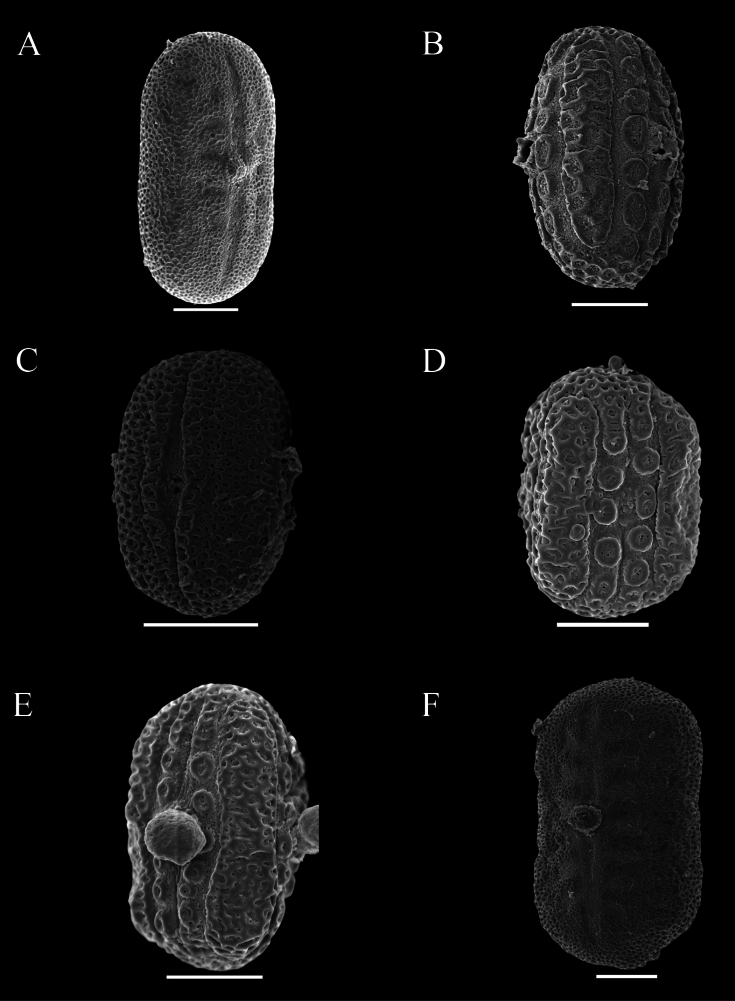
Pollen images **A***Justiciawarmingii* (*Villanueva* 920) **B***J.werffii* (*Van der Werff* 15565) **C***J.schunkei* (*Schunke* 7599) **D***J.spathuliformis* (*Villanueva* 1028) **E***J.saccata* (*Villanueva* 997) **F***J.lactiflora* (*Woytkowski* 34339). Scale bars: 10 μm.

**Figure 52.**
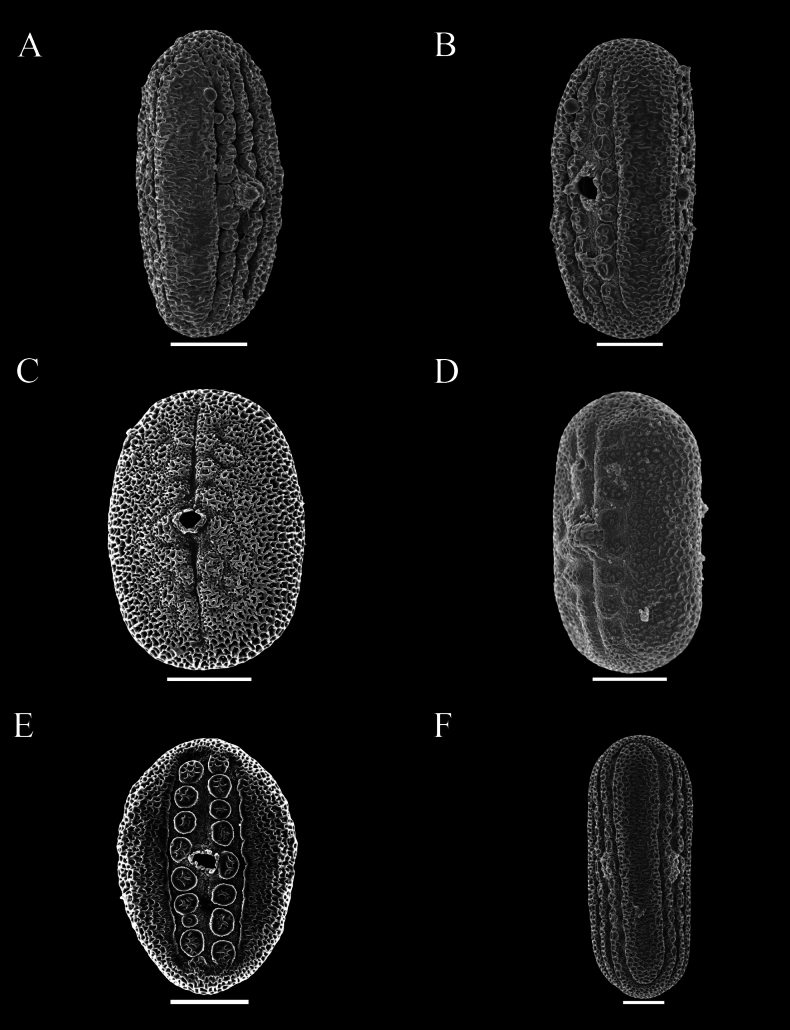
Pollen images **A***Justiciabambusiformis* (*Dudley* 10440) **B***J.valenzuelae* (*Valenzuela* 8722) **C***J.huallagensis* (*Knapp* 7879) **D***J.oxapampensis* (*Villanueva* 963) **E***J.falcifolia* (*Ortiz* 660) **F***J.hyalina* (*Scolnik* 909). Scale bars: 10 μm.

**Figure 53.**
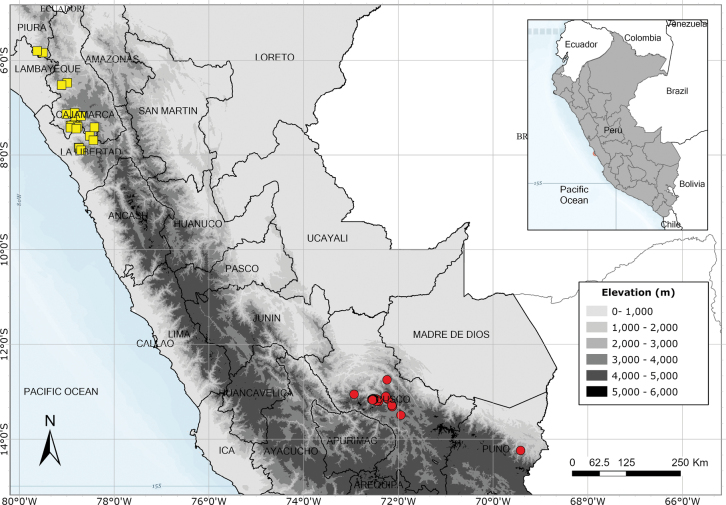
Distribution of Justiciaalpinasubsp.alpina (yellow squares) and J.alpinasubsp.machupicchuensis (red circles) in Peru.

**Figure 54.**
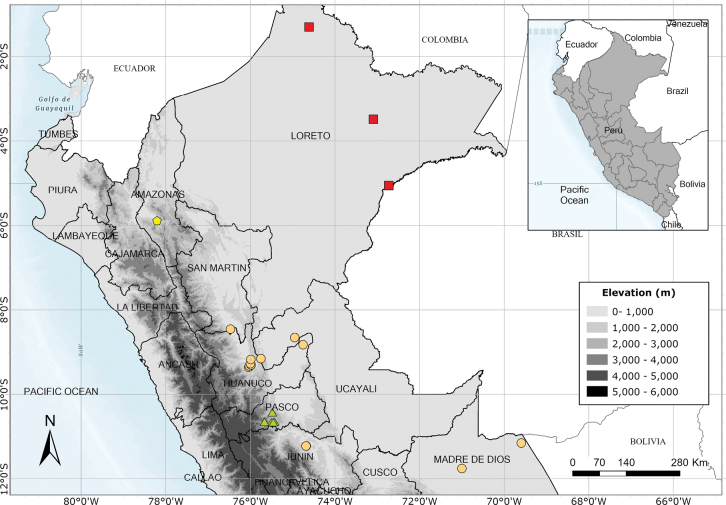
Distribution of *Justiciacuspidulata* (yellow polygon), J.discolorsubsp.discolor (orange circles), J.discolorsubsp.filisepala (red squares) and *J.rojasiae* (green triangles) in Peru.

**Figure 55.**
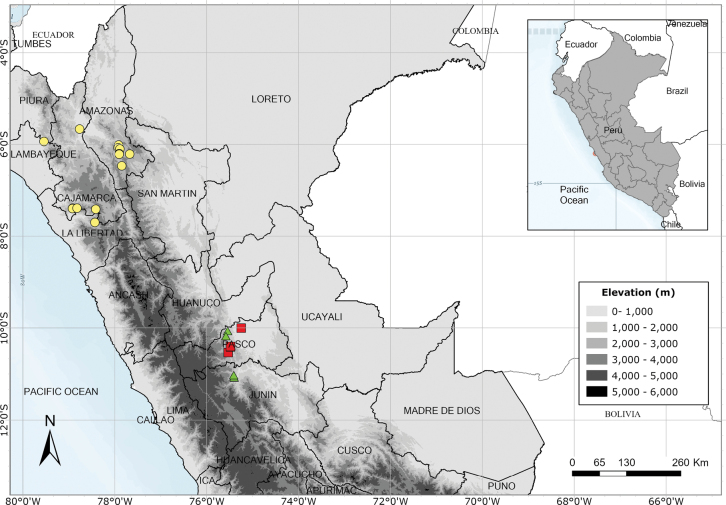
Distribution of *Justiciachimboracensis* (yellow circles), *J.pozuzoensis* (green triangles) and *J.oppositiflora* (red squares) in Peru.

**Figure 56.**
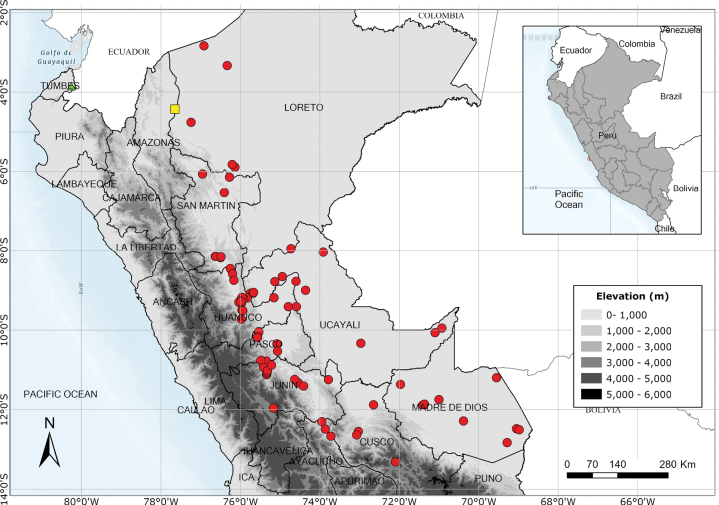
Distribution of *Justiciaappendiculata* (red circles), *J.aphelandroides* (yellow square) and *J.tumbesiana* (green triangle) in Peru.

**Figure 57.**
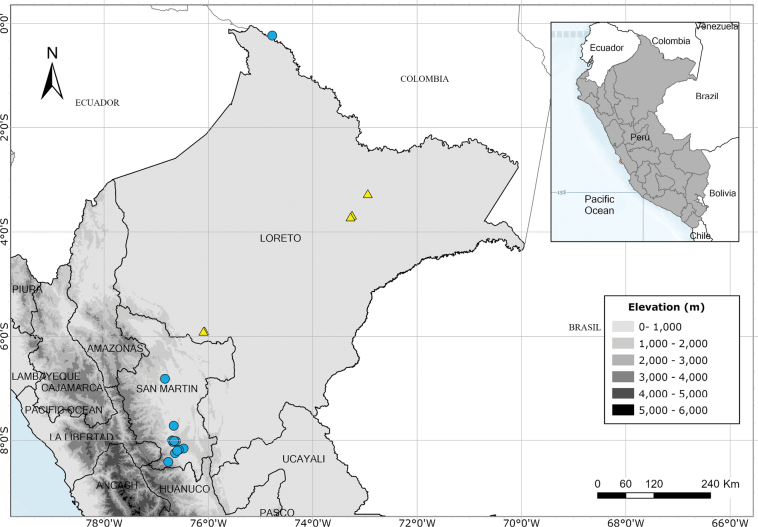
Distribution of *Justiciapelianthia* (yellow triangles) and *J.sanchezioides* (blue circles) in Peru.

**Figure 58.**
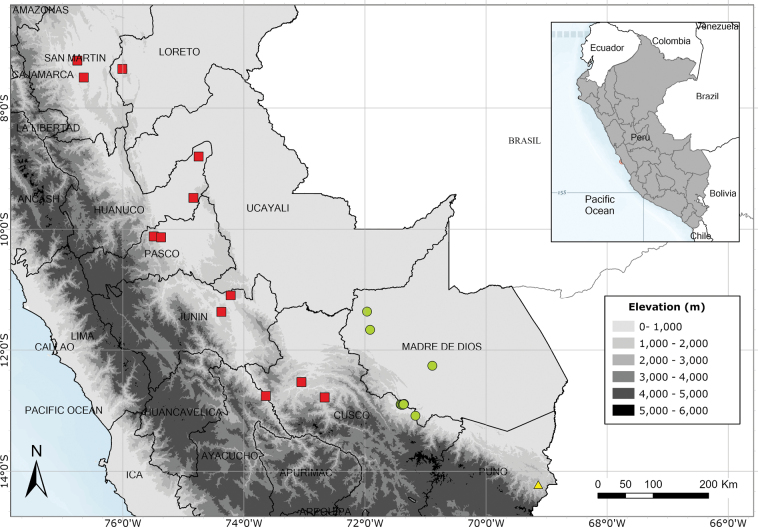
Distribution of *Justiciabeckii* (yellow triangles), *J.rauhii* (green circles) and *J.siraensis* (red squares) in Peru.

**Figure 59.**
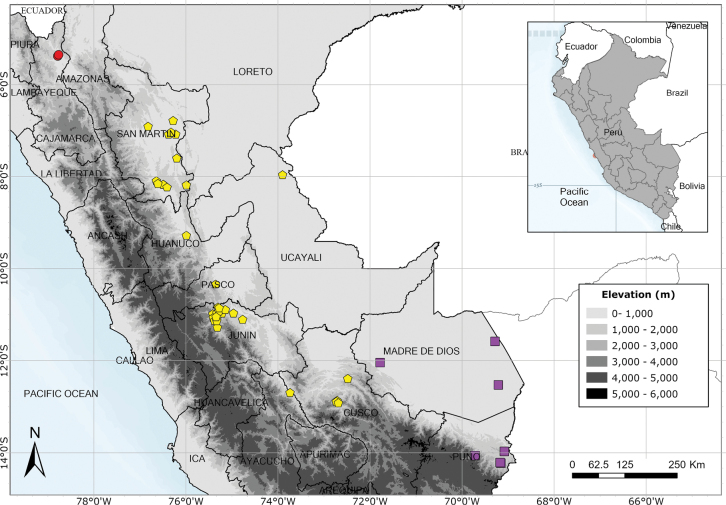
Distribution of *Justiciaramulosa* (purple squares), *J.radicans* (yellow polygons) and *J.angustituba* (red circle) in Peru.

**Figure 60.**
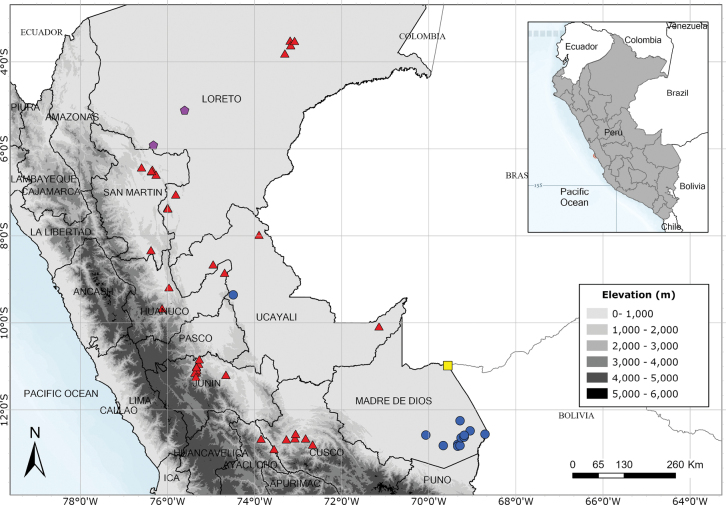
Distribution of *Justiciariedeliana* (blue circles), *J.sprucei* (yellow square), *J.longibracteata* (purple polygons) and *J.rusbyi* (red triangles) in Peru.

**Figure 61.**
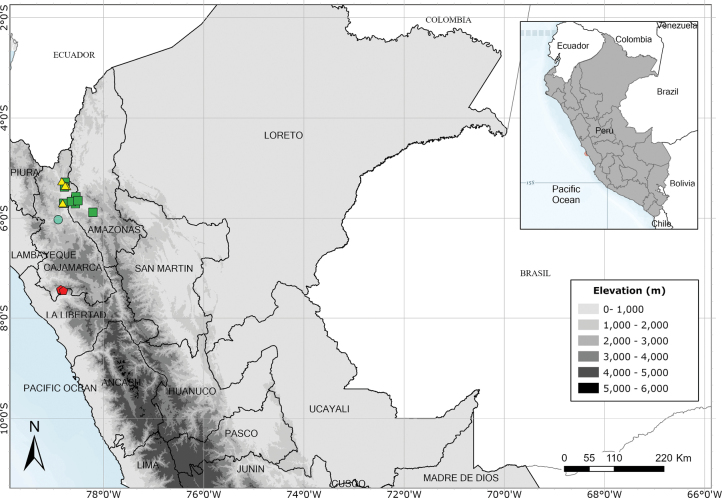
Distribution of *Justiciareginaldii* (light blue circle), *J.baguensis* (green squares), *J.cajamarcensis* (yellow triangles) and *J.sagasteguii* (red polygons) in Peru.

**Figure 62.**
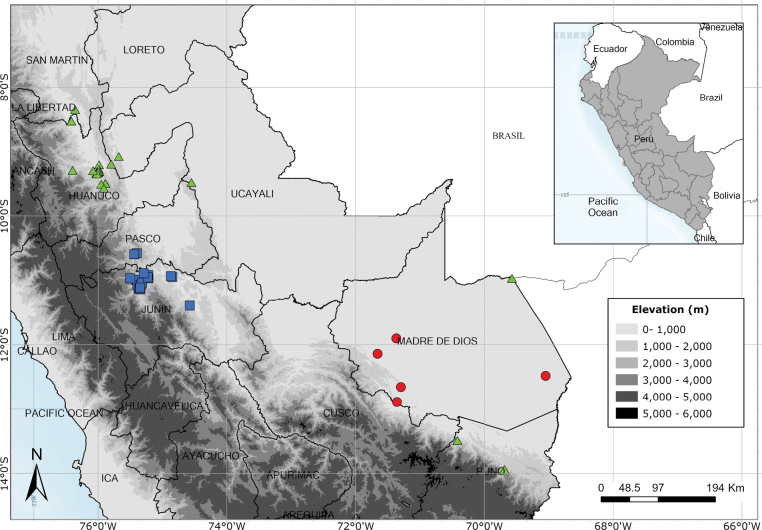
Distribution of *Justiciadryadum* (red circles), *J.tenuiflora* (green triangles) and *J.warmingii* (blue squares) in Peru.

**Figure 63.**
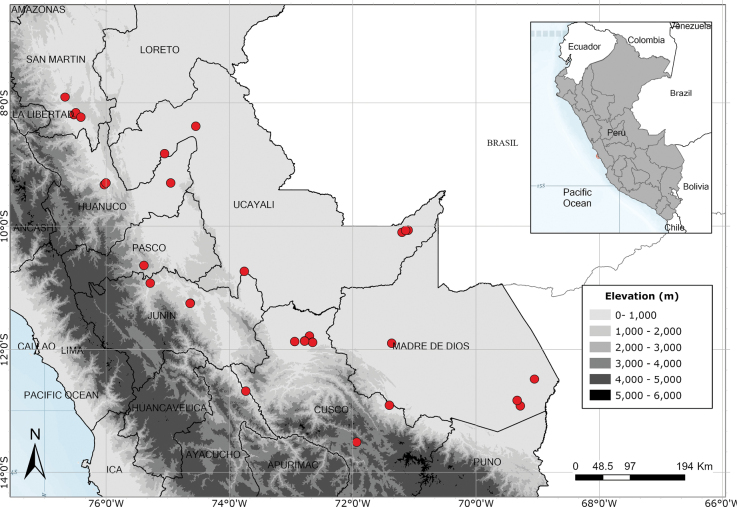
Distribution of *Justicialineolata* in Peru.

**Figure 64.**
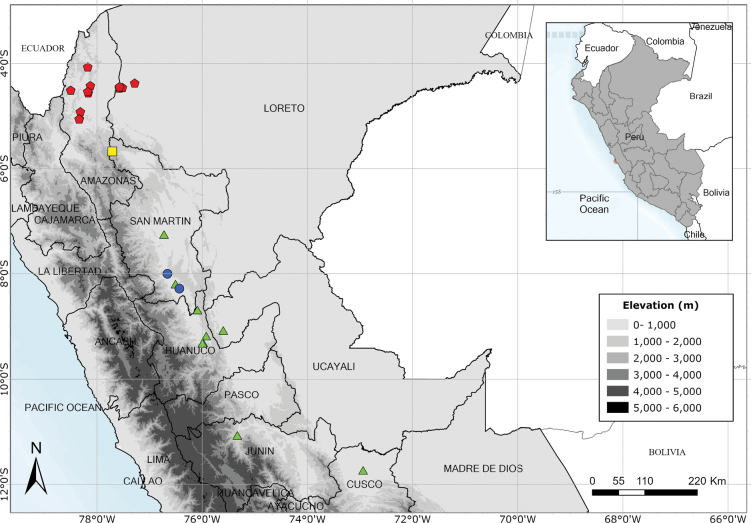
Distribution of *Justiciachlamydocardioides* (red polygons), *J.schunkei* (blue circles), *J.werffii* (yellow square) and *J.spathuliformis* (green triangles) in Peru.

**Figure 65.**
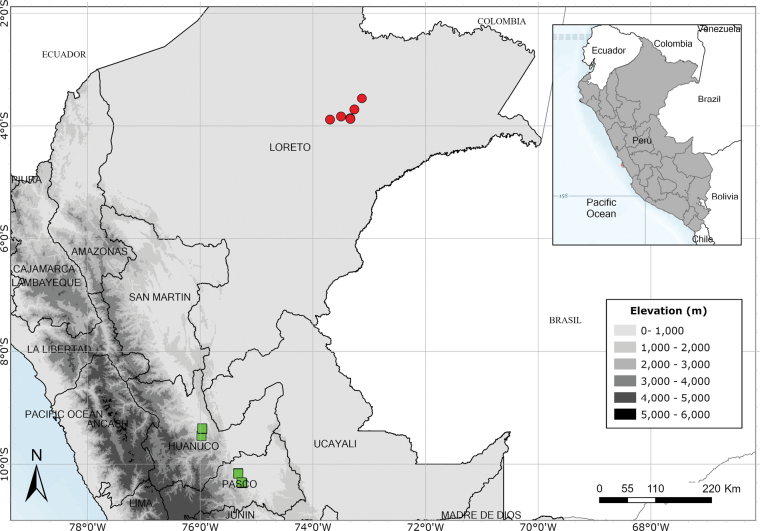
Distribution of *Justiciasaccata* (green squares) and *J.chamaecaulis* (red circles) in Peru.

**Figure 66.**
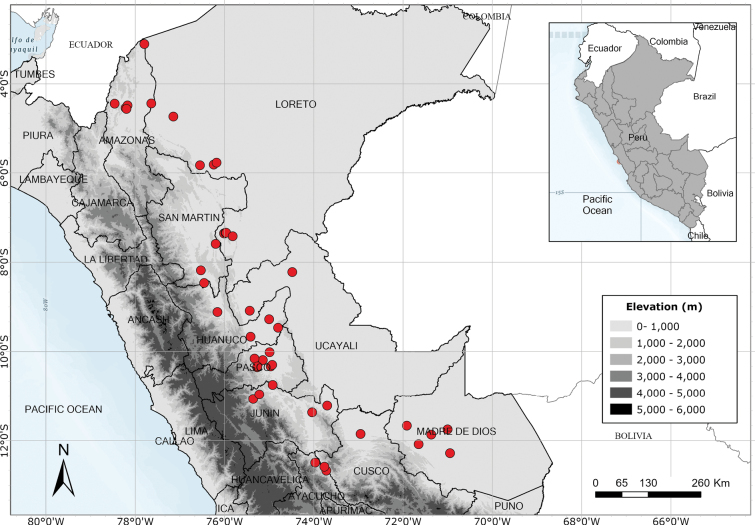
Distribution of *Justiciasecundiflora* in Peru

**Figure 67.**
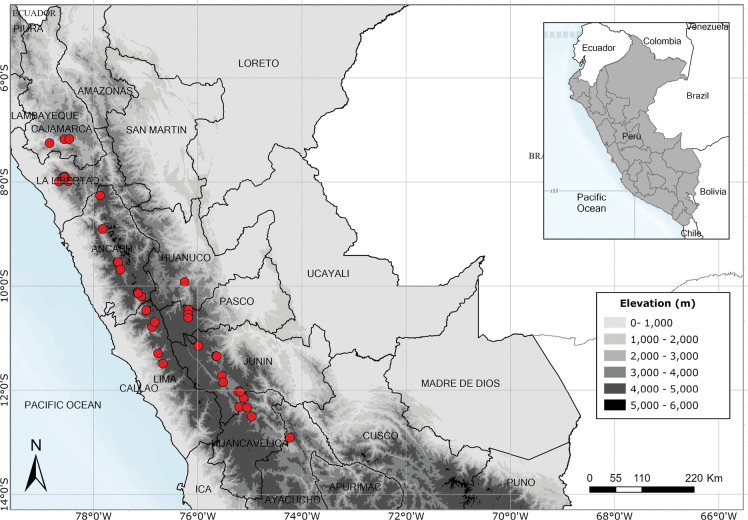
Distribution of *Justiciasericea* in Peru.

**Figure 68.**
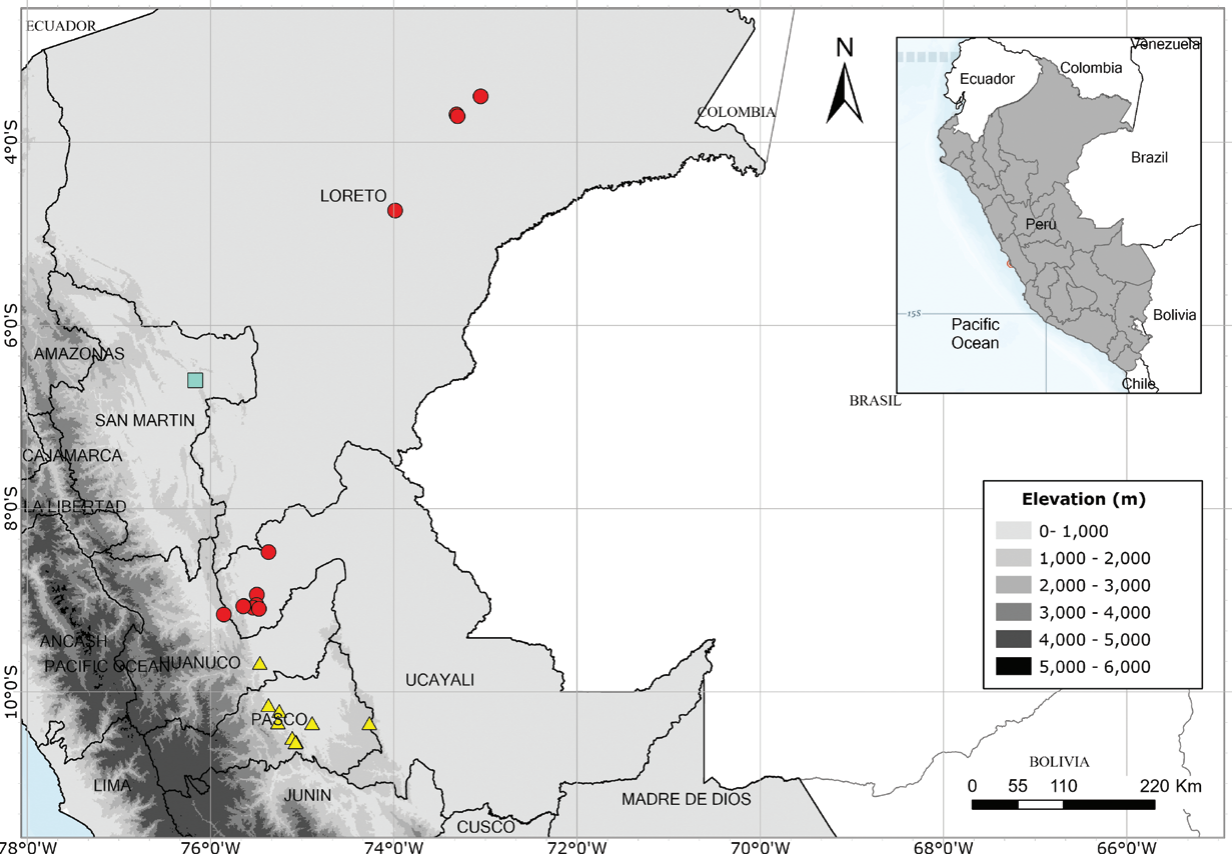
Distribution of *Justiciaoxapampensis* (yellow triangles), *J.lactiflora* (red circles) and *J.huallagensis* (light blue square) in Peru.

**Figure 69.**
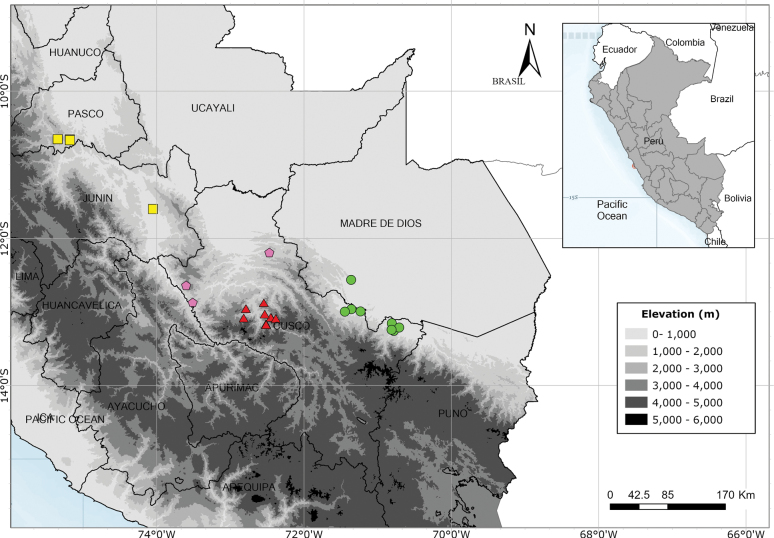
Distribution of *Justiciabambusiformis* (pink polygons), *J.valenzuelae* (red triangles), *J.falcifolia* (yellow squares) and *J.hyalina* (green circles) in Peru.

## Supplementary Material

XML Treatment for Justicia alpina

XML Treatment for Justicia alpina subsp. alpina

XML Treatment for Justicia alpina subsp. machupicchuensis

XML Treatment for Justicia cuspidulata

XML Treatment for Justicia chimboracensis

XML Treatment for Justicia rojasiae

XML Treatment for Justicia pozuzoensis

XML Treatment for Justicia oppositiflora

XML Treatment for Justicia discolor

XML Treatment for Justicia discolor subsp. discolor

XML Treatment for Justicia discolor subsp. filisepala

XML Treatment for Justicia chamaecaulis

XML Treatment for Justicia appendiculata

XML Treatment for Justicia tumbesiana

XML Treatment for Justicia pelianthia

XML Treatment for Justicia sanchezioides

XML Treatment for Justicia aphelandroides

XML Treatment for Justicia rauhii

XML Treatment for Justicia siraensis

XML Treatment for Justicia beckii

XML Treatment for Justicia radicans

XML Treatment for Justicia ramulosa

XML Treatment for Justicia angustituba

XML Treatment for Justicia riedeliana

XML Treatment for Justicia sprucei

XML Treatment for Justicia longibracteata

XML Treatment for Justicia rusbyi

XML Treatment for Justicia reginaldii

XML Treatment for Justicia baguensis

XML Treatment for Justicia cajamarcensis

XML Treatment for Justicia sagasteguii

XML Treatment for Justica dryadum

XML Treatment for Justicia lineolata

XML Treatment for Justicia tenuiflora

XML Treatment for Justicia warmingii

XML Treatment for Justicia chlamydocardioides

XML Treatment for Justicia schunkei

XML Treatment for Justicia werffii

XML Treatment for Justicia spathuliformis

XML Treatment for Justicia saccata

XML Treatment for Justicia secundiflora

XML Treatment for Justicia sericea

XML Treatment for Justicia lactiflora

XML Treatment for Justicia bambusiformis

XML Treatment for Justicia valenzuelae

XML Treatment for Justicia huallagensis

XML Treatment for Justicia oxapampensis

XML Treatment for Justicia falcifolia

XML Treatment for Justicia hyalina

## ﻿﻿Additional information

### Author ORCIDs

Rosa Villanueva-Espinoza https://orcid.org/0000-0003-2989-8160

Yunfei Deng https://orcid.org/0000-0002-0876-3286

Robert Scotland https://orcid.org/0000-0002-6371-2238

John R. I. Wood https://orcid.org/0000-0001-5102-3729

### Data availability

All of the data that support the findings of this study are available in the main text.
